# Using coding and non-coding rare variants to target candidate genes in patients with severe tinnitus

**DOI:** 10.1038/s41525-022-00341-w

**Published:** 2022-11-30

**Authors:** Alvaro Gallego-Martinez, Alba Escalera-Balsera, Natalia Trpchevska, Paula Robles-Bolivar, Pablo Roman-Naranjo, Lidia Frejo, Patricia Perez-Carpena, Jan Bulla, Silvano Gallus, Barbara Canlon, Christopher R. Cederroth, Jose A. Lopez-Escamez

**Affiliations:** 1 Otology & Neurotology Group CTS495, Department of Genomic Medicine, GENYO, Centre for Genomics and Oncological Research: Pfizer/University of Granada/Andalusian Regional Government, PTS Granada, Avenida de la Ilustración, 114, 18016 Granada, Spain; 2 Department of Otolaryngology, Instituto de Investigación Biosanitaria, ibs.Granada, Hospital Universitario Virgen de las Nieves, 18014 Granada, Spain; 3 Sensorineural Pathology Programme, Centro de Investigación Biomédica en Red en Enfermedades Raras, CIBERER, 28029 Madrid, Spain; 4 Section of Experimental Audiology, Department of Physiology and Pharmacology, Karolinska Institutet, 171 77 Stockholm, Sweden; 5 Department of Surgery, Division of Otolaryngology, University of Granada, 18016 Granada, Spain; 6 Department of Mathematics, University of Bergen, 5020 Bergen, Norway; 7 Department of Psychiatry and Psychotherapy, University of Regensburg, 93053 Regensburg, Germany; 8 Department of Environmental Health Sciences, Istituto di Ricerche Farmacologiche Mario Negri IRCCS, 20156 Milan, Italy; 9 National Institute for Health Research (NIHR) Nottingham Biomedical Research Centre, Nottingham University Hospitals NHS Trust, Ropewalk House, Nottingham, NG1 5DU UK; 10 Hearing Sciences, Division of Clinical Neuroscience, School of Medicine, University of Nottingham, Nottingham, NG7 2UH UK

**Keywords:** Genetics research, Diagnostic markers

## Abstract

Tinnitus is the phantom percept of an internal non-verbal set of noises and tones. It is reported by 15% of the population and it is usually associated with hearing and/or brain disorders. The role of structural variants (SVs) in coding and non-coding regions has not been investigated in patients with severe tinnitus. In this study, we performed whole-genome sequencing in 97 unrelated Swedish individuals with chronic tinnitus (TIGER cohort). Rare single nucleotide variants (SNV), large structural variants (LSV), and copy number variations (CNV) were retrieved to perform a gene enrichment analysis in TIGER and in a subgroup of patients with severe tinnitus (SEVTIN, *n* = 34), according to the tinnitus handicap inventory (THI) scores. An independent exome sequencing dataset of 147 Swedish tinnitus patients was used as a replication cohort (JAGUAR cohort) and population-specific datasets from Sweden (SweGen) and Non-Finish Europeans (NFE) from gnomAD were used as control groups. SEVTIN patients showed a higher prevalence of hyperacusis, hearing loss, and anxiety when they were compared to individuals in the TIGER cohort. We found an enrichment of rare missense variants in 6 and 8 high-constraint genes in SEVTIN and TIGER cohorts, respectively. Of note, an enrichment of missense variants was found in the *CACNA1E* gene in both SEVTIN and TIGER. We replicated the burden of missense variants in 9 high-constrained genes in the JAGUAR cohort, including the gene *NAV2*, when data were compared with NFE. Moreover, LSVs in constrained regions overlapping *CACNA1E*, *NAV2*, and *TMEM132D* genes were observed in TIGER and SEVTIN.

## Background

Tinnitus is the phantom percept of an internal non-verbal set of noises and tones reported by more than 15% of the population and it is usually associated with hearing and/or brain disorders^1^. Severe tinnitus is considered a disorder in around 1% of the population, and it is associated with emotional distress, cognitive dysfunction, and/or autonomic arousal, leading to behavioral changes and functional disability^2^.

Evidence from epidemiological studies in twins, adoptees, and familial aggregation supports a genetic contribution to tinnitus that may help distinguish environmental effects from heritability^3–6^. This heritability seems to be higher in women reporting severe tinnitus, which is strongly associated with hyperacusis^7^. Two genome-wide association studies (GWAS) have recently identified several common variants associated with tinnitus in non-coding regions using UK Biobank data obtained from patients with self-reported tinnitus^8,9^.

Amanat et al. used an alternative approach by selecting patients with Meniere’s disease (MD), an inner ear disorder characterized by episodes of vertigo associated with sensorineural hearing loss (SNHL), that also presented an extreme tinnitus phenotype^10^. They reported a burden of rare missense variants in 24 synaptic genes including *ANK2*, *TSC2*, and *AKAP9* identified using exome sequencing. These findings were replicated in an independent cohort of tinnitus patients without MD, but not in a large cohort of patients with generalized epilepsy, confirming the specificity of these genes to severe tinnitus. Together, exome sequencing and GWAS data suggest an additive polygenic model of inheritance consisting of common and rare SNVs that might shape the phenotype. However, the genetic architecture contributing to tinnitus disorder is not well understood, and the role of structural variants (SVs) in coding and non-coding regions has not been investigated in patients with severe tinnitus. Previous genomic studies in brain disorders such schizophrenia or Alzheimer have shown the contribution of SVs in complex phenotypes^11,12^.

Therefore, this study aims to explore the association of rare single-nucleotide variants (SNVs), large structural variations (LSVs), and copy number variants (CNVs) in the genome of Swedish patients with severe tinnitus.

## Results

### Clinical features

Most individuals with tinnitus reported hearing loss and hyperacusis in TIGER (64% hearing loss, 69% hyperacusis) and SEVTIN (79% hearing loss, 94% hyperacusis) cohorts (Table 1). However, we found that hyperacusis (*p* = 0.002), hearing loss (*p* = 0.040) and anxiety (*p* = 0.013) were significantly more common in the SEVTIN than in the TIGER cohort. Supplementary Table 1 displays the sociodemographic data from the chronic constant tinnitus groups and non-tinnitus controls. Hearing loss, headache and hyperacusis were more frequent in JAGUAR constant tinnitus subjects than in controls. However, depression and anxiety did not differ between groups (Supplementary Table 1).

**Table 1:** Clinical features in Swedish patients with tinnitus for all (TIGER) and severe tinnitus (SEVTIN) cohorts.

	TIGER	SEVTIN	*p* value
	*N* = 97	*N* = 34	
Age (mean ± SD)	46.02 ± 12.86	48.84 ± 13.48	
Sex (% women)	54 (56%)	16 (47%)	
Hearing loss, *n* (%)	62 (64%)	27 (79%)	0.040
Hyperacusis, *n* (%)	67 (69%)	32 (94%)	0.002
Headaches, *n* (%)	35 (36%)	11 (32%)	0.459
HADS-Anxiety >8, *n* (%)	54 (56%)	25 (74%)	0.013
HADS-Depression >8, *n* (%)	30 (31%)	13 (38%)	0.163
Tinnitus duration			
6 months–3 years, *n* (%)	16 (16%)	8 (24%)	0.080
3 years to 10 years, *n* (%)	21 (22%)	5 (15%)	0.047
10 years to 20 years, *n* (%)	33 (34%)	13 (38%)	0.428
more than 20 years, *n* (%)	17 (18%)	5 (15%)	0.410
No information, *n* (%)	4 (4%)	3 (9%)	0.039

Patients in the SEVTIN cohort were selected if Tinnitus Handicap Inventory (THI) score ≥58.

*HADS* Hospital Anxiety and Depression Scale, *THI* tinnitus handicap inventory.

### Single-nucleotide variation

#### Enrichment of SNV and short indels in patients with tinnitus

A gene burden analysis (GBA) was performed for all rare LoF and missense variants (MAF < 0.01) found in the coding regions in the TIGER and SEVTIN cohorts using the allelic frequencies reported in SweGen and gnomAD as references (Supplementary Table 2A, C). We found 8 genes with a burden of LoF variants in the SEVTIN cohort (*TUT4*, *FAM135A*, *KIAA1109*, *DNAH7*, *TMEM232*, *TMEM41A*, *ATP7B*, *DYNLT2B*). Of note, four of them were considered mutation-intolerant genes (pLI > 0.9, LOEUF < 0.5). *TUT4* OR = 1.67 (1.11–2.24, *p* = 1.82^−06^), *FAM135A* OR = 3.43 (1.81–5.04, *p* = 8.88^−03^), *KIAA1109* OR = 3.14 (1.62–4.65, *p* = 1.33^−02^), *DNAH7* OR = 3.81 (2.01–5.61, *p* = 8.60^−03^) (Supplementary Table 3).

**Fig. 1:**
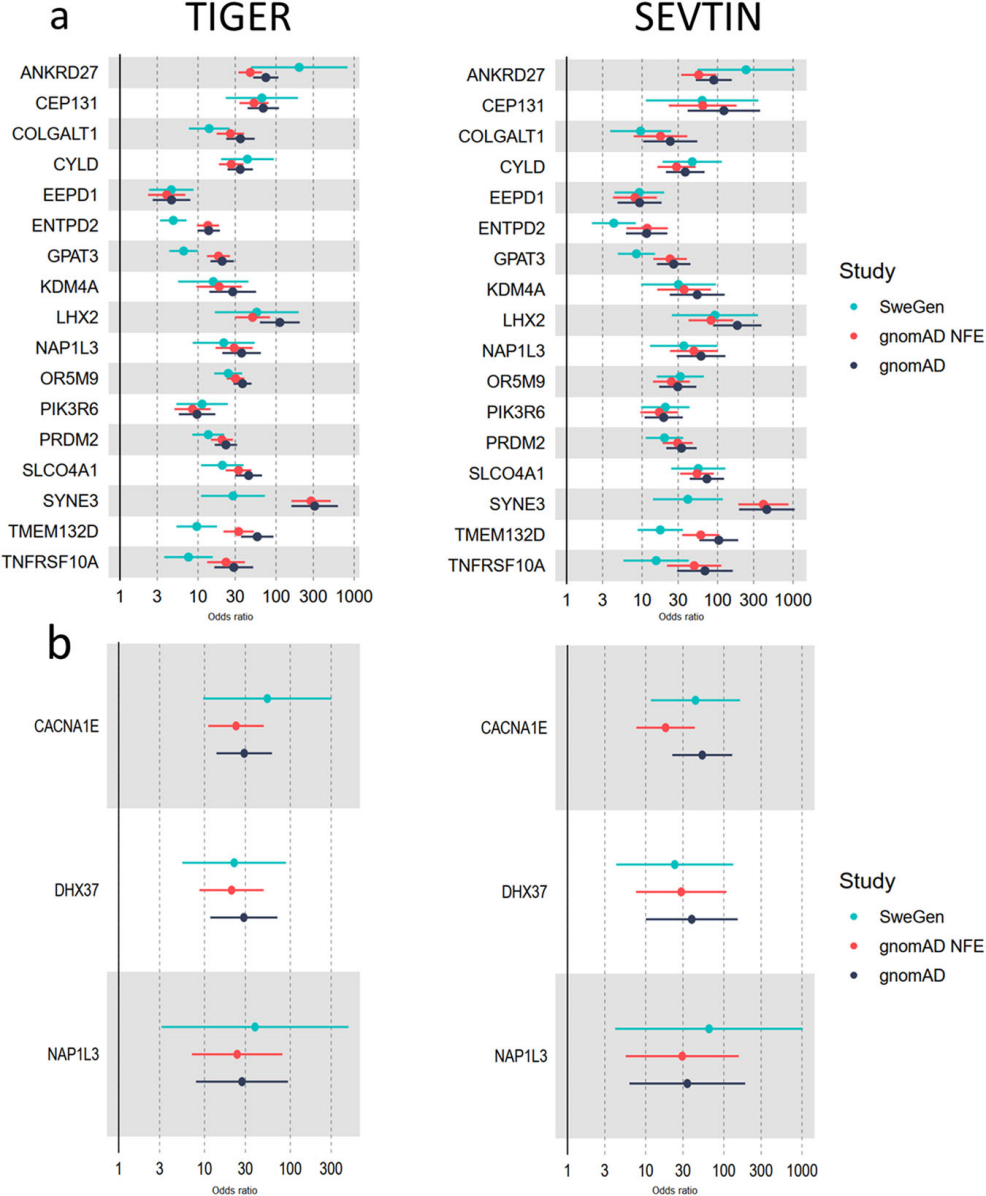
Odds ratios for loss of function (LoF) and missense variants found in enriched genes in patients with tinnitus. **a** Genes with a burden of LoF variants in TIGER (left) and SEVTIN (right) cohorts; **b** genes with a burden of missense variants in TIGER (left) and SEVTIN (right).

When we compared genes with variants significantly enriched in both TIGER and SEVTIN, a total of 17 genes were shared between both cohorts (Supplementary Table 4). The top-ranked mutation-intolerant genes were *KDM4A* OR = 3.41 (2.27–4.55, *p* = 1.25^−06^), *CYLD* OR = 3.83 (2.93–4.73, *p* < 1.00^−16^), *LHX2* OR = 4.52 (3.21–5.84, *p* = 1.08^−07^), *PRDM2* OR = 2.98 (2.41–3.56, *p* < 10^−16^) and *TMEM132D* OR = 2.86 (2.17–3.55, *p* = 6.30^−11^). To search for associations of rare variants in the same gene, we retrieved the individuals reporting at least two variants in the same gene showing enrichment of LoF SNVs (Fig. 1a and Supplementary Table 4). An enrichment of missense variants was found in 3 mutation-intolerant genes in both TIGER and SEVTIN cohorts: *CACNA1E* OR = 3.99 (3.11–4.88, *p* = 2.10^−15^), *DHX37* OR = 3.10 (2.39–3.82, *p* = 6.12^−14^) and *NAP1L3* OR = 3.66 (2.38–4.94, *p* = 7.24^−05^) (Fig. 1b).

We also found some rare variants previously reported in genes associated with tinnitus such as *ANK2, AKAP9* and *TSC2* (Supplementary Table 5). Eight missense variants in *ANK2* were found in 6 individuals of the TIGER cohort and 2 of them were also found in 3 patients of the SEVTIN cohort (rs141191319 and rs764914059). Moreover, no SNVs were found associated with either hyperacusis or hearing loss in the TIGER cohort.

#### WES replication cohort

Replication was performed in the JAGUAR cohort following the same bioinformatics pipeline used in TIGER. We found several genes that were replicated when they were compared with Non-Finnish European (NFE) from gnomAD, including *NAV2* (OR = 3.40 (2.15–4.64)) (Supplementary Table 2B, D). For LoF-enriched genes, we replicated a burden of LoF variants in three genes: *PTCH2*, *RAB25* and *TNFRSF10A*. However, the three genes found had a LOEUF > 0.5, so they were considered as loss-of-function mutation-tolerant genes. Also, they were found not enriched when compared with Swedish control dataset (Supplementary Table 6A).

We also found 24 genes with a burden of missense variants in at least one reference dataset using TIGER or SEVTIN, which were replicated in JAGUAR. Among them, we found 9 genes (6 of them in SEVTIN and 8 of them in TIGER) with low LOEUF values (LOEUF < 0.5) (Supplementary Table 6B). *NAV2* was only found enriched in SEVTIN, while the other three were found enriched in both TIGER and SEVTIN for at least one reference comparison.

### Structural variations

#### Large structural variants

We found a total of 6603 LSVs (duplications, deletions) with a length between 1 Kb and 1 Mb in the TIGER cohort. After quality filtering, removing high signaling and low mappable calls, we retrieved a total of 4630 LSVs in TIGER that were previously annotated in SweGen. Among them, 241 LSVs were classified as variants of unknown significance and 37 LSVs were annotated as likely pathogenic or pathogenic, according to AnnotSV and ACMG guidelines. Importantly, 4 LSVs classified as likely pathogenic overlapped a gene in a highly constrained region (gnomAD pLI ≥0.9, LOEUF bin score <2) (Supplementary Table 7).

#### Burden of rare LSV in constrained regions

A total of 189 LSVs were in overlapping genes and regions with a high constrain reported in gnomAD (gnomAD pLI >0.9, LOEUF bin score <2). For burden analysis, we considered LSVs those classified as pathogenic, likely-pathogenic and of unknown significance. We found that 84 of these 189 LSVs were ultra-rare in TIGER; these were selected for a second burden test (Supplementary Table 8). We found ultra-rare LSVs in constrained regions in the TIGER cohort, including a deletion (11:19617912–19620833) overlapping the mutation-intolerant gene *NAV2* that also showed an enrichment of missense variants in the TIGER and SEVTIN cohorts. Moreover, the deletion 12:129087937–129089566 involving the gene *TMEM132D* was more frequently observed in TIGER and SEVTIN than in SweGen (Supplementary Table 9). This burden effect was replicated in the SEVTIN cohort for ultra-rare LSVs in constrained regions. Differences between subgroups (hyperacusis, hearing loss) were not significant when we selected only ultra-rare LSVs and compared them with the SweGen reference dataset. Finally, there was no difference in the frequency of LSVs when we performed a sex-specific analysis in either men or women.

#### Copy number variation

CNV calls were analyzed along the genome in the TIGER and SEVTIN cohorts. We compared the gain and loss for common CNVs in both TIGER and SweGen cohorts. A total of 1501 CNVs in both sets were retrieved. Among them, 36 CNVs were not found in the SweGen cohort, the remaining CNV were observed at least once in SweGen.

Next, we evaluated the CNV retrieved in the TIGER cohort according to its predicted pathogenicity using the ACMG guidelines for LSV. CNV reported were categorized as likely pathogenic or pathogenic. However, none of the CNV overlapped a candidate gene showing enrichment of SNV (Supplementary Table 10).

#### LSV and CNV in candidate genes for severe tinnitus

Individuals with missense variants in known genes described for severe tinnitus such as *ANK2, TSC2, and AKAP9* were targeted for specific LSVs overlapping these genes. We found a copy gain variant close to the region where the *ANK2* gene is located. A similar finding is also reported in one individual in the Database for Genomic Variants (DGV, accession number *nsv1006992)*. Furthermore, a small deletion of 76 bp was detected in the intron 2 of the *ANK2* gene 4:113136968–113137044, but this variant was also reported in one individual in SweGen. This indel overlaps with an AluSz repetition region in this intron, but it was discarded as the region has a high rate of benign mutations due to short repetitions.

On the other hand, we found few LSVs located on the same loci that included enriched genes for missense and LoF variants in the SEVTIN or TIGER cohorts. All these LSVs were classified as of unknown significance according to ACMG guidelines for LSV (Table 2).

**Table 2:** SVs overlapping genes enriched in LoF and missense variants in SEVTIN and TIGER cohorts.

Overlapping candidate gene	Structural variant coordinates (ChrStartEnd)	Type of SV	TIGER (*n* = 97)	SEVTIN (*n* = 34)	SweGen (*n* = 1000)	*p* value
NAV2	11:19617912–19620833	DEL	1 (0.010)	1 (0.029)	1 (0.001)	0.098
TMEM132D	12:129087937–129089566	DEL	21 (0.216)	8 (0.235)	17 (0.017)	<0.001
CACNA1E	1:65590243–213271783	DUP	1 (0.010)	1 (0.029)	5 (0.005)	0.070
	1:65590258–213271483	DEL	3 (0.030)	3 (0.088)	26 (0.026)	0.349
	1:114479837–212725029	DEL	5 (0.051)	3 (0.088)	39 (0.039)	0.063

*p* values are calculated using Chi-square (*χ*^2^) test between observed frequencies in TIGER and expected frequencies found in SweGen.

#### Brain visualization of candidate gene expression

**Fig. 2:**
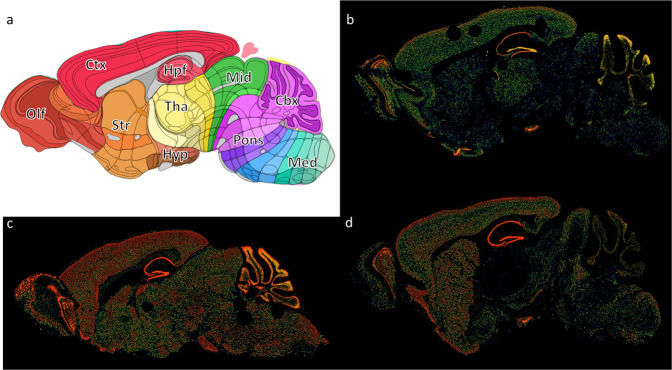
Expression data for adult mouse sagittal brain sections in Allen Brain Atlas for three candidate genes. **a** Anatomical annotations overlap layer of mouse brain areas in a sagittal overview from the Allen Mouse Brain Atlas and Allen Reference Atlas - Mouse Brain, at the same slice position as **b** and **c** and **d**. **b** NAV2 expression layer data (mouse.brain-map.org/experiment/show/69443367). **c** ANK2 expression layer data (mouse.brain-map.org/experiment/show/68844707). **d** CACNA1E expression layer data (mouse.brain-map.org/experiment/show/69236897). Each expression cube is color-coded by average expression intensity ranging from blue (low intensity) through green to red (high intensity). Ctx cortex, Hpf hippocampal formation, Tha thalamus, Hyp hypothalamus, Str striatum, Olf olfactory bulb, Mid midbrain, Pons pons, Med medulla, Cbx cerebellum. Allen Mouse Brain Atlas, mouse.brain-map.org and atlas.brain-map.org.

By using ISH expression data from adult mouse brain sagittal sections (Allen Brain Atlas)^13^, we found a strong signal in several regions including the cortex, hippocampal region, cerebellum and olfactory bulbs for *NAV2* and *CACNA1E* (Fig. 2b–d). The spatial distribution of *CACNA1E* gene expression found in the adult mouse brain was comparable to the expression profile found in *ANK2* (Fig. 2c). We also found this spatial expression profile in human brain^14^. Hence, we extracted their microarray data for six human brains to assess the correlation between the different probes found for each candidate gene. We found a significant co-expression for at least one probe for the genes *NAV2*, *CACNA1E* and *ANK2* (Supplementary Fig. 1). The human brain regions showing significant co-expression for the three genes include temporal lobe (superior temporal gyrus, middle temporal gyrus, inferior temporal gyrus, fusiform gyrus), frontal lobe (superior frontal gyrus), insula, hippocampal formation (dentate gyrus), and limbic system (cingulate gyrus) (Supplementary Fig. 2).

## Discussion

The present study illustrates that the application of burden tests on LoF, missense and, particularly, LSV in the human genome across different cohorts can improve the identification of candidate genes in complex disorders. By using an extreme phenotype approach and burden tests, we have found three new candidate genes for tinnitus were identified: *NAV2, TMEM132D* and *CACNA1E*. First, we found a burden of missense variants in the mutation-intolerant gene *NAV2* in the SEVTIN and JAGUAR cohorts, and a large deletion in the same gene the in one patient. Second, we also observed an enrichment of LoF variants in TIGER and SEVTIN and a large deletion involving the *TMEM132D* gene. Finally, the gene *CACNA1E* showed a burden of missense variants in the TIGER and SEVTIN cohort and several deletions were also observed in TIGER patients. Our results indicate that the identification of rare LSV in constrained regions can facilitate a better understanding of genetic variation in complex disorders such tinnitus.

The THI was used to estimate tinnitus-related distress and to select patients with a severe tinnitus phenotype (SEVTIN cohort). This strategy has been previously used in complex disorders to target candidate genes with a burden of rare missense variations^10,15^. Following this approach, Amanat et al.^10^ reported a burden of rare missense variants in patients with severe tinnitus in several genes including *ANK2, AKAP9* and *TSC2*. Our results confirm that severe tinnitus is associated with hyperacusis, hearing loss and anxiety, according to the HADS and THI scores in both sexes, consistent with large epidemiological studies^7,16^. Here, these findings were partially replicated for *ANK2* and 2 variants (rs141191319—NM_001148.6:c.7132G>A, Exon 38, VUS) and rs764914059—NM_001148.6:c.8242C>A, Exon 38—likely benign), which were found in 3/34 patients in the SEVTIN cohort. *ANK2* is a member of the ankyrin family with an important role in the organization of plasma membranes, linking integral proteins to the spectrin-actin cytoskeleton. Two major ankyrin-2 proteins have been described, being one of them an isoform especially expressed in the brain, particularly in postcentral gyrus in cortex^17^. Also, exon 38, where both described variants were found, is brain-specific.

There was a burden of LoF and missense variants in some genes shared in the TIGER and SEVTIN cohorts. These variants should be considered as susceptibility variants for tinnitus as a “symptom”, which is a common condition in the general population, since this enrichment was found in both, patients with and without a severe tinnitus phenotype. We have found several genes selectively enriched in LoF variants in SEVTIN that were not significantly enriched in the TIGER cohort. The difference may be related to the heterogeneity of individuals in TIGER, but also to the selection of the severe phenotype in SEVTIN.

Seventeen genes enriched in LoF variants were found in both cohorts with a higher odds ratio in SEVTIN than TIGER. Among them, at least 5 genes were annotated as mutation-intolerant genes: *KDM4A*, *CYLD*, *LHX2*, *PRDM2* and *TMEM132D*. The most interesting candidate gene is *TMEM132D*, a transmembrane protein only known for its capacity to act as a cell-surface marker for oligodendrocyte differentiation and neuronal morphogenesis^18^. Polymorphisms in *TMEM132D* have been associated with anxiety^19^, depression^20^, and panic disorder^21^. This gene is mainly expressed in the brain, particularly in the frontal cortex.

The burden analysis of missense variants found 3 mutation-intolerant genes shared between both cohorts, TIGER and SEVTIN: *CACNA1E*, *DHX37* and *NAP1L3*. *CACNA1E* encodes the alpha-1E subunit of the R-type calcium channels, which belongs to the ‘high-voltage activated’ channel involved in the firing patterns modulation of neurons important for information processing^22^. These channels mediate the entry of calcium ions into excitable cells and are also involved in a variety of calcium-dependent processes, including muscle contraction, hormone or neurotransmitter release, gene expression, cell motility, cell division, and cell death. This gene has been associated with epileptic encephalopathy^23^ among other neurodevelopmental disorders with epilepsy^24,25^. *CACNA1E* is highly expressed in brain (particularly in the *nucleus accumbens*) and neural tissue in general, according to GTEx. *DHX37* is a helicase involved in several cellular processes implicated in alterations of RNA secondary structure^26^. This helicase is an important protein highly expressed in every tissue in the organism. However, it has also been associated with developmental and epileptic encephalopathy and Neurodevelopmental Disorder with Brain Anomalies and with or Without Vertebral or Cardiac Anomalies (NEDBAVC)^27,28^. Lastly, *NAP1L3* is an intronless gene involved in the nucleosome assembly as a histone chaperone^29^. Although *NAP1L3* function is not well-known, it is expressed in neural tissues, especially cortex, hypothalamus, and cerebellum.

The *NAV2* gene, a mutation-intolerant gene was enriched for missense variants in both SEVTIN and JAGUAR cohorts when comparing with NFE-gnomAD, being a potential candidate gene for tinnitus disorder. The gene *NAV2* is involved in neuronal development and different sensory organs development^30,31^ and it has been associated with neuroblastoma^32^.

Some ultra-rare LSVs found in TIGER and SEVTIN, particularly in highly constrained regions, were not found in the SweGen reference cohort. These LSVs may increase the susceptibility to tinnitus when they are found in regions with a significant burden of protein-coding short variants. Some studies have observed that the deletion of elements in highly constrained regions can lead to different neurological diseases such as Schizophrenia^8^. Some of these highly constrained regions, such as topologically-associating domain (TAD) boundaries, are important in the chromatin structure as well as the access to regulatory elements needed for gene expression^33–35^. The enrichment of LSVs has also been reported in other diseases such autism^36^ and epilepsy^37^, and their role in a tinnitus disorder remains difficult to interpret. Genes overlapping these highly constrained regions include genes that have been associated with neuropsychiatric disorders such as schizophrenia^38^.

Four potential likely-pathogenic LSVs overlapping highly constrained regions deserve further analyses. Among the rest of the observed variants, 58 LSVs had a frequency of <1% in the SweGen cohort, but only 6 of them were found in >1 individual in the TIGER cohort. Some of the LSVs overlapped with several genes including *TMEM132D*, *DLGAP2*, *TSHZ1*, and *COL4A1*. However, the functional impact of these LSVs remains unknown^39^.

Some duplications and deletions were overlapping genes such as *TMEM132D*, *NAV2* and *CACNA1E*, which were also enriched for LoF and missense variants in both cohorts. Interestingly, the *NAV2* gene was found enriched in missense variants in patients with severe tinnitus, and this enrichment was successfully replicated in the JAGUAR cohort. *NAV2* encodes the protein neuron navigator 2 (or NAV2, unc53h2), which is related to neurite outgrowth and axonal elongation^31^. The inducible knockdown of NAV2 in SH-SY5Y cells alters all-*trans* retinoic acid-stimulated neurite outgrowth. Also, a mutant mouse lacking the full-length NAV2 protein (a hypomorphic mutant) display problems in several sensory systems and show resistance to pain. Nav2/unc53H2 mutant embryos show lower nerve fiber density as well as cranial nerves IX and X sometimes fused or poorly connected to the brain^30^. We have observed how *NAV2* and *CACNA1E* are co-expressed in the same areas of the brain that *ANK2*, including the hippocampal formation, which seems to be involved in severe tinnitus^40^.

The most widely accepted model to explain the generation of tinnitus is the amplification of spontaneous activity in the auditory pathway^41–43^. The intrinsic neuronal excitability after sensory deprivation could occur at the axon initial segments, as well as stochastic increase in axonal connectivity may provide novel substrates for additional neurological consequences^17^. Since we also reported a burden of missense variants in the *ANK2* gene in patients with severe tinnitus, we propose a potential role of axonal elongation and branching in tinnitus disorder^10^.

Finally, we have found some limitations in our study. First, our study was performed in the Swedish population and considered the entire cohort of SweGen individuals as controls. Nevertheless, according to the prevalence of severe tinnitus in Sweden^5^, it is probable that some of the individuals included in the SweGen cohort may also experience severe tinnitus. However, misclassification of SweGen subjects with tinnitus as controls is unlikely to affect the results or the power of the study. A second limitation is the self-reported nature of the tinnitus phenotype. As tinnitus is a subjective percept, it has traditionally been assessed by means of questionnaires^44^. Such findings, together with emerging evidence of additional electrophysiological^45^ and neuroimaging signatures for tinnitus^46–48^ suggest that objective diagnostic methods for the assessment of tinnitus are underway and may improve patient selection. Third, despite the known impact of sex on tinnitus severity^49,50^ and the increased genetic liability in women with severe tinnitus^5^, our study was insufficiently powered to perform a sex-stratified analysis. Finally, given the high prevalence of hearing loss and hyperacusis in our sample, disentangling the genetic contribution of the identified targets on each co-morbidity has proven challenging. However, while this may appear as a limitation, the lack of previous association of the identified targets with hearing loss suggests that these are strong candidates for severe tinnitus. Consequently, larger studies will be required to further provide additional evidence in supporting these claims.

As conclusion, we extract the extreme phenotype approach and the integration of missense, LoF, and LSV in the burden analysis in constraint regions can improve the identification of candidate genes in complex disorders. In this study, we report a burden of missense and LSV in constraint regions and support *CACNA1E*, *NAV2*, and *TMEM132D* as new candidate genes that contribute to severe tinnitus.

## Methods

### Participants and ethics

The project was approved by the local ethics committee “Regionala etikprövningsnämnden” in Stockholm (2015/2129-31/1). Written informed consent was obtained from all subjects.

Adult participants (>18 years old) from LifeGene^51^ were recruited to the Swedish Tinnitus Outreach Project (STOP) and registered their interest on the STOP website (https://stop.ki.se), after which they received additional information and a consent form by mail. After consenting, 5671 participants answered several online questionnaires, translated and validated in Swedish^52^, between June 2016 and January 2020. In brief, the online survey consisted of the Tinnitus Sample Case History Questionnaire (TSCHQ), the Tinnitus Handicap Inventory (THI), the Tinnitus Functional Index (TFI), the Tinnitus Catastrophizing Scale (TCS), the Fear of Tinnitus Questionnaire (FTQ), the Hospital Anxiety and Depression Scale (HADS), the Perceived Stress Questionnaire (PSQ-30), the hyperacusis questionnaire (HQ) and four domains of the World Health Organization Quality of Life Scale (WHOQoL-BREF). The ‘European School for Interdisciplinary Tinnitus Research Screening Questionnaire’ (ESIT-SQ) was developed with specific attention to questions about potential risk factors for tinnitus (including demographics, lifestyle, general medical and otological histories), and tinnitus characteristics (including perceptual characteristics, modulating factors, and associations with comorbidities). The ESIT-SQ^53,54^ was added to the platform in November 2018 and was answered by a subset of 4590 participants (80.9%).

A first case–control study was previously designed including 97 patients with chronic and constant tinnitus (>6 months and with a TFI score ≥ 48, meaning “tinnitus is a big problem”^55^), referred hereafter as the TIGER or discovery cohort^10^. DNA samples from these cases were obtained through LifeGene and subjected to WGS in November 2019. Sequencing data from healthy subjects were obtained from the SweGen project^56^, a population-based high-quality genetic variant dataset from the Swedish population. The SweGen cohort includes a total of 1000 individuals, of which 942 individuals were selected from The Swedish Twin Registry (STR) and 58 individuals from The Northern Swedish Population Health Study (NSPHS). Details on this database have been previously described^56^. The allelic frequencies from the Non-Finish European population in gnomAD was also used as external controls^57^. The following clinical variables were retrieved from TIGER: age, sex, age of onset of tinnitus, hyperacusis, headache, and hearing loss using survey questions from the TSCHQ (items 26, 28, and 30). The tinnitus handicap inventory (THI) was used to assess the impact of tinnitus on health-related quality of life as previously described^5,7,58,59^. We used the THI score to define patients with severe tinnitus (>58; SEVTIN cohort), that show greater tinnitus distress than those with a TFI ≥ 48.

A case-control study design was also chosen for the replication cohort (JAGUAR). Five hundred and forty-eight individuals with constant tinnitus were selected and their age and sex-matched non-tinnitus controls were subsequently identified. To refine the selection for the whole-exome sequencing (WES), we excluded individuals who either did not report constant tinnitus two times in a row or did not report “no tinnitus” two times in a row (using the Intro3 question from the TSCHQ and the ESITSQ A17 item). Among these 548 cases with robust assessment of tinnitus over time, we selected 147 individuals with chronic and constant tinnitus from the STOP cohort with the same ancestry as the TIGER cohort. These tinnitus cases (JAGUAR cohort) were compared with population-specific dataset from Sweden (SweGen). Sociodemographic data and comorbidities are reported in Supplementary Table 1.

### Genome sequencing

Genomic libraries were prepared from ~1 μg DNA using Illumina TruSeq PCR-free DNA sample preparation kits targeting an insert size of 350 bp. Concentration was measured by fluorometry using Quant-iT dsDNA HS assay kit (Thermofisher Scientific). Also, the quality of the library was assessed by capillary electopheresis using TapeStation (Agilent). Libraries were sequenced by the NovaSeq 6000 sequencing platform according to manufacturer protocols at 2 × 150 bases read length.

### Bioinformatics analyses

#### Alignment and variant calling

The BAM files were handled in a server in the SNIC UPPMAX HPC systems (Uppsala, Sweden). We classified variant datasets into short variants (SNV/short indels), LSVs (DUP, DEL), and CNVs. The main calling protocol was streamlined using Nextflow pipeline Sarek v.2.6.1^60^ to ensure both portability and reproducibility. Software dependencies for the entire pipelines are encapsulated in Docker and Singularity containers accessible from Docker Hub. The pipeline is separated in two main steps: preprocessing (including alignment, deduplication and base recalibration) and calling. Aligment was performed using BWA mem and Hg38 reference genome from GATK resource bundle. Following preprocessing steps included marking duplicates and Base Quality Score Recalibration (BQSR) using both GATK tools MarkDuplicates and BaseRecalibrator/ApplyBQSR respectively. We used known polymorphic sites from GATK resource bundle to build recalibration tables for variants in samples using BaseRecalibrator. Recalibration tables were streamlined to ApplyBQSR to correct for systematic bias that affect the assignment of base quality scores by the sequencer. Preprocessing quality control stats were calculated and annotated using samtools stats. We generated and processed all cases and healthy controls BAM files together, and performed a joint genotyping of short variants across all samples using GATK (v4.1)^61–64^.

**Fig. 3:**
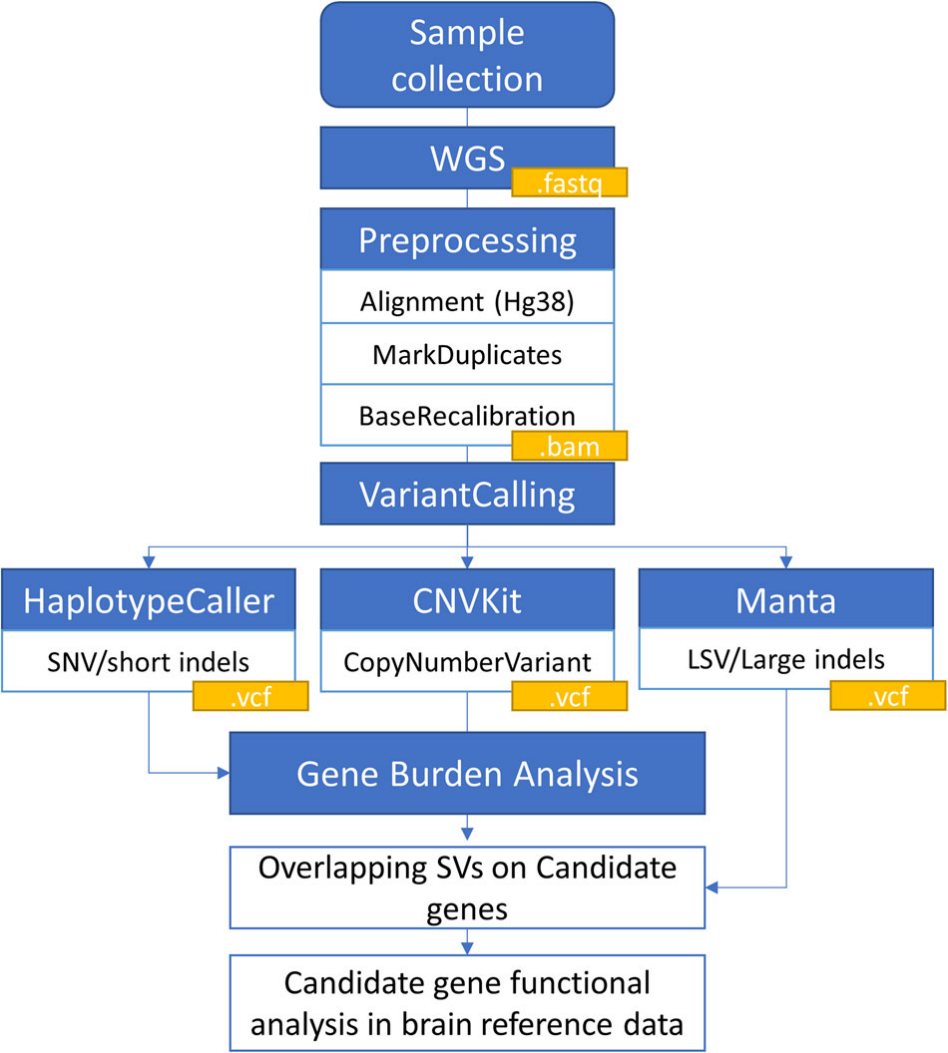
Bioinformatic pipeline for analyses of genome sequencing datasets according to the type of variant (SNV/short indels, copy number variant or structural variants/ large indels). In detail, output format for each step file is described in yellow boxes, processing steps in blue boxes, and tools or methods used in white boxes.

Variant calling was performed using HaplotypeCaller for SNV and short indels using default parameters recommended by GATK. HaplotypeCaller generated unfiltered gVCF files. To further select high-quality genetic variants, the GATK Variant Quality Score Recalibration (VQSR) filtering was executed on SNVs and indels separately using GATK Variant Recalibrator and Apply Recalibration walkers. GATK VQSR pipeline was used to filter variants as recommended^63^. The SNV VQSR model was trained using SNP sites from HapMap3.3, 1000 Genomes Project (1000GP), Illumina Omni 2.5M SNP arrays, 1000GP Phase 1 high-confidence SNPs, and dbSNP (v138). A 90.0% sensitivity threshold was applied to filter variants resulting in a Ti/Tv (transition to transversion) ratio of 2.195. The indel VQSR model was trained using high confidence indel sites from 1000GP and dbSNP (v138) and a 90% sensitivity threshold. We kept only the “PASS” variants based on the results of VQSR filtering. A summary workflow is represented in Fig. 3.

#### Structural variants calling

We applied Manta, a complementary algorithm from Illumina for the discovery and genotyping of LSVs^65^. Manta calls LSVs from mapped paired-end sequencing reads. We used Manta with default parameters for LSV calling on the entire set. As a second tool to measure specifically CNVs, we used CNVkit (0.9.8)^66^ following recommended batch standard procedure for germline variants. Structural variants greater than 1 Mb underwent additional filtering. Called LSVs found in SweGen or gnomAD with an overlap of 99% were kept as true positive LSVs, filtering novel LSVs not reported in any database as part of the LSV quality filtering.

#### Variant annotation and prioritization

We used Variant Effect Predictor VEP (v106)^67^, vcfanno (v0.2.9)^68^, and AnnotSV (v3.0.6)^69^ for variant annotations. The short variant allelic frequencies were annotated by using population allele frequencies obtained from gnomAD v3 genomes for GRCh38. LSVs were annotated using population allele frequencies from 1000GP and Database of Genomic Variants (DGV)^70^ as well as gnomAD v2 SV database liftover to GRCh38^57^. We used the default settings in AnnotSV. LSVs were quality-filtered if they overlapped by more than 66% with large genome gaps (e.g., centromeres), segmental duplications, or regions subject to somatic V(D)J recombination in white blood cells, arguing that these variant calls are likely artefactual. Also, following American College Medical Genetics (ACMG) adapted recommendations^71–73^, we filtered out benign and likely benign overlapping areas according to different databases annotated through AnnotSV. Finally, we extracted variants with MAF ≥ 0.01 for common variant association analysis and the remaining for rare variant analysis.

To assess constrained sequences in humans and the tolerance of each gene to loss-of-function (LoF) variants, we annotated variants using 2 scoring systems: pLI (*probability of being loss-of-function intolerant*) and LOEUF (*loss-of-function observed/expected upper bound fraction*) scores from the ExAC/gnomAD database for coding regions. Both values estimate the tolerance to protein-truncating variants for each gene^57^. Following ExAC annotations, we also considered pLI ≥0.9 as an extremely intolerant set of transcripts, so variants in those transcripts were used to generate a high-constraint variant dataset and define mutation-intolerant genes. Following gnomAD notations, we also used LOEUF value <0.5 for high-constraint genes. For LSVs covering high-constraint regions, we used LOEUF bin score (minimal “decile bin of LOEUF” for given transcripts of a gene) <2, whose values have been precalculated for GRCh38 through AnnotSV^69^.

For transcript-level annotations, we annotated variants with VEP using Ensembl transcripts from GENCODE. For SNVs/short indels, we further annotated the variants using the annotation database dbNSFP 4.1_a. Exonic SNVs/short indels were classified according to the effect on the protein sequence: synonymous, missense non-damaging, missense damaging, and loss-of-function (stop-gain, stop loss, start loss, frameshift, or splice donor/acceptor). Additionally, we used the CADD database (Combined Annotation Dependent Depletion)^74^ to annotate the damaging score for each coding variant and the CADD Indel score to annotate the potentially damaging effects on indels.

For the CNVkit output, annotation was addressed by overlapping positions of the called CNVs on the refFlat gene names database from UCSC^75^. Scatter plots were used to represent log2(ratio) for each interesting CNV call. We also retrieved and annotated Swedish variant frequencies using the SweGen database as the reference population.

#### Variant interpretation

Candidate variant analysis was performed to search for SNVs that could segregate the tinnitus phenotype. First, we filtered the variants in the TIGER cohort (MAF < 0.01) according to the frequencies observed in gnomAD and SweGen annotations. Second, we targeted those coding variants with a major damaging score. We used the CADD phred score, annotated from CADD (>20), a value that is standardized as likely pathogenic by the database. All potential candidate variants were assessed and evaluated following the guidelines provided by the American College of Medical Genetics and Genomics (ACMG) and the Association for Molecular Pathology (AMP)^72,76^. For LSVs, we followed the modifications proposed by Riggs et al.^71^ and annotated by AnnotSV^69^. Variants scored from 3 to 5 according to the ACMG guidelines and ClinGen were considered as unknown significance, likely-pathogenic and pathogenic and used for posterior analysis.

### Statistical analysis

The duration of tinnitus and the relative frequencies of the main comorbidities (anxiety, hearing loss, hyperacusis, headache) were compared by *t* test and chi-squared (or *χ*^2^) test, respectively. Normality for each variable was tested using Shapiro–Wilk normality test.

We performed a GBA to search for rare variants associated with each dataset. For each comparison, we addressed population frequencies from the different databases mentioned above. Clustering was used to reduce granularity in the dataset to search for specific variants enriched for each trait, before comparing each subgroup to Swedish controls.

The GBA was performed in the coding regions for SNVs/short indels (less than 50 bp). We extracted rare coding variants in the TIGER and SEVTIN cohorts and compared their allelic frequencies with the observed frequencies in the different control datasets. For this, we collapsed rare variation along each gene using the Wald chi-squared test, to compare cases and control^77^. As frequency threshold for each cohort, we used minor allele frequency for the number of samples in each cohort. For each enriched gene in TIGER and SEVTIN, we retrieved the VUS, likely-pathogenic and pathogenic variants according to Riggs et al.^71^.

For LSV analyses, we annotated those variants overlapping high-constraint regions. We used LOEUF bin scores <2 as a threshold to select variants in highly constrained genome regions, following AnnotSV notations. Afterward, we compared the observed frequencies in cases and control for LSVs overlapping high constraint boundaries. Finally, we also performed a burden analysis with the LSV found at least in one individual in the TIGER cohort, clustered by type of LSV (duplications or deletions), frequency and constraint.

### Visualization and brain expression of candidate genes

We used Allen Brain Atlas^13,14,78,79^ (ABA) data to assess the gene expression of candidate genes on different areas of the human and mouse brain. We used ISH data from Mouse and Human Brain Atlas to locate which areas of the brain express the candidate genes. Therefore, if genes of interest were expressed in brain, we retrieved microarray expression data from ABA. We extracted expression data for different probes of each gene of interest and calculated the correlation coefficient between probe expression. We selected those probes with significant correlation between them to check co-expression between different regions of the brain. We also used *ANK2* mouse expression data as previously reported (Amanat et al.^10^).

### Reporting summary

Further information on research design is available in the Nature Research Reporting Summary linked to this article.

## Supplementary information

Supplemental material

Nature Reporting Summary

## Data Availability

The datasets used and/or analyzed during the current study are available from the corresponding author on reasonable request. Individual data will be made accessible upon request to C.R.C., with qualified investigators whose proposal of data use has been approved for tinnitus research by an independent review committee. A data transfer agreement will have to be established with the Karolinska Institutet. Aggregated data of variants found in TIGER, SEVTIN and JAGUAR can be found and downloaded on Zenodo, under 10.5281/zenodo.7304956. SweGen data can be obtained from the Swedish Frequency resource for genomics (SweFreq) on https://swefreq.nbis.se/. Non-Finnish European data from gnomAD can be downloaded from https://gnomad.broadinstitute.org/downloads. Allen Brain Atlas microarray expression data can be consulted in https://human.brain-map.org/. Allen Brain Atlas mouse brain data can be accessed through mouse.brain-map.org and atlas.brain-map.org.
