# Supplementary material for: Using coding and non-coding rare variants to target candidate genes in patients with severe tinnitus
Source: NPJ Genom Med. 2022 Nov 30;7:70. doi: 10.1038/s41525-022-00341-w (PMC9712652; doi:10.1038/s41525-022-00341-w)

Supplementary Data

Index

Supplementary table 1	2
Supplementary table 2a	3
Supplementary table 2b	12
Supplementary table 2c	44
Supplementary table 2d	47
Supplementary table 3	57
Supplementary table 4	58
Supplementary table 5	59
Supplementary table 6	60
Supplementary table 7	63
Supplementary table 8	64
Supplementary table 9	67
Supplementary table 10	68
Supplementary figure 1	69
Supplementary figure 2	70

Supplementary table 1. Clinical features in constant and non-constant replication WES cohort. Information regarding the selection of these two cohorts can be found in Methods.

	CONTROLS (N=155)	JAGUAR (N=143)	p value
**Age**			
- Mean (SD)	46.394 (10.945)	47.951 (11.824)	
**Sex**			
- Female	84 (54.2%)	68 (47.6%)	
- Male	71 (45.8%)	75 (52.4%)	
**Hearing Loss**			< 0.001
- No	104 (67.1%)	45 (31.5%)	
- Yes	32 (20.6%)	79 (55.2%)	
**Hyperacusis**			0.031
- No	23 (14.8%)	10 (7.0%)	
- Yes	132 (85.2%)	133 (93.0%)	
**Headache**			< 0.001
- No	0 (0.0%)	113 (79.0%)	
- Yes	0 (0.0%)	27 (18.9%)	
**HADS Anxiety**			0.518
- No	132 (85.2%)	117 (82.4%)	
- Yes, > 8	23 (14.8%)	25 (17.6%)	
**HADS Depression**			0.311
- No	150 (96.8%)	134 (94.4%)	
- Yes, > 8	5 (3.2%)	8 (5.6%)	
**Tinnitus Onset**			< 0.001
- No information	155 (100.0%)	0 (0.0%)	
- 0 to 6 months	0 (0.0%)	2 (1.4%)	
- 10 to 20 years	0 (0.0%)	49 (34.3%)	
- 3 to 10 years	0 (0.0%)	28 (19.6%)	
- 6 months to 3 years	0 (0.0%)	20 (14.0%)	
- More than 20 years	0 (0.0%)	40 (28.0%)	

Supplementary table 2a. Swedish discovery cohort. Genes with significant enrichment in LoF variants in TIGER (N= 97) and SEVTIN (N=34) cohorts. Odds ratio (OR) were calculated for the allelic frequencies reported in Non-Finish European (NFE) from gnomAD, all individuals from gnomAD (ALL) and SweGen. If no LoF variants was reported in NFE or SweGen cohorts, OR cannot be calculated and cells are empty. Multiple hypothesis testing correction for each pvalue have been addressed by False Discovery Rate (FDR) approach for the total number of genes and total number of variants for each comparison.

Gene	N Variants	logOR (CI) NFE	pvalue	pvalue corrected	logOR (CI) ALL	pvalue	pvalue corrected	logOR (CI) Swedish	pvalue	pvalue corrected
ABCF1	2	5.81 (3.85-7.77)	6.45E-09	3.88E-06	6.20 (4.41-8.00)	1.16E-11	7.31E-09			
ACOX2	2	5.81 (3.85-7.77)	6.47E-09	3.89E-06	5.69 (4.05-7.33)	1.12E-11	7.02E-09			
ACSF2	2	4.24 (2.71-5.78)	6.01E-08	3.61E-05	4.24 (2.78-5.69)	1.19E-08	7.49E-06			
ADGRF3	2	4.11 (2.60-5.63)	9.97E-08	5.99E-05	4.82 (3.32-6.33)	3.23E-10	2.03E-07			
AGR3	2	3.68 (2.21-5.15)	9.76E-07	5.87E-04	4.01 (2.57-5.45)	5.11E-08	3.21E-05	0.55 (-0.96-2.05)	4.77E-01	1.00E+00
ANKRD27	36	4.30 (3.93-4.67)	0.00E+00	0.00E+00	3.84 (3.50-4.19)	0.00E+00	0.00E+00	5.29 (3.86-6.71)	3.77E-13	1.50E-10
ARPC5	2				4.80 (3.30-6.30)	3.54E-10	2.23E-07			
ATP2A1	13	4.80 (4.15-5.45)	0.00E+00	0.00E+00	4.25 (3.67-4.83)	0.00E+00	0.00E+00			
BCO2	2	5.81 (3.85-7.78)	6.43E-09	3.86E-06	6.61 (4.65-8.57)	4.08E-11	2.57E-08			
BEST4	5	2.69 (1.79-3.60)	4.82E-09	2.90E-06	2.40 (1.51-3.28)	1.22E-07	7.64E-05			
BFSP1	6	2.19 (1.38-3.01)	1.39E-07	8.37E-05	2.21 (1.40-3.02)	8.62E-08	5.42E-05	1.74 (0.74-2.73)	6.57E-04	2.62E-01
CAMK2A	2	5.12 (3.42-6.82)	3.79E-09	2.28E-06	5.22 (3.67-6.78)	4.69E-11	2.95E-08			
CAMSAP2	13	3.44 (2.86-4.02)	0.00E+00	0.00E+00	3.28 (2.72-3.84)	0.00E+00	0.00E+00	3.53 (2.41-4.66)	7.18E-10	2.87E-07
CANX	17	3.83 (3.32-4.35)	0.00E+00	0.00E+00	3.78 (3.29-4.28)	0.00E+00	0.00E+00	2.88 (2.10-3.67)	5.79E-13	2.31E-10
CBX6	4	6.45 (4.75-8.15)	9.67E-14	5.81E-11	5.53 (4.38-6.67)	0.00E+00	0.00E+00			
CCDC63	4	2.17 (1.17-3.16)	2.07E-05	1.25E-02	2.70 (1.71-3.70)	1.02E-07	6.43E-05	1.94 (0.67-3.21)	2.79E-03	1.00E+00
CCDC7	6	1.82 (1.00-2.63)	1.18E-05	7.11E-03	2.27 (1.46-3.08)	4.30E-08	2.70E-05	1.43 (0.48-2.38)	3.29E-03	1.00E+00
CD36	2	4.90 (3.25-6.54)	5.19E-09	3.12E-06	4.82 (3.32-6.32)	3.10E-10	1.95E-07			
CDC25B	45	3.22 (2.90-3.54)	0.00E+00	0.00E+00	2.88 (2.57-3.19)	0.00E+00	0.00E+00	2.78 (2.32-3.25)	0.00E+00	0.00E+00
CEP131	24	4.23 (3.77-4.69)	0.00E+00	0.00E+00	3.96 (3.52-4.39)	0.00E+00	0.00E+00	4.19 (3.12-5.25)	1.21E-14	4.85E-12
CFAP251	2	5.12 (3.42-6.82)	3.62E-09	2.17E-06	5.51 (3.91-7.11)	1.63E-11	1.03E-08			
CHRNA3	2				6.59 (4.63-8.55)	4.67E-11	2.94E-08			
CNGB1	9	4.06 (3.33-4.79)	0.00E+00	0.00E+00	3.63 (2.94-4.31)	0.00E+00	0.00E+00	2.49 (1.53-3.46)	3.97E-07	1.59E-04
COLGALT1	25	3.55 (3.13-3.97)	0.00E+00	0.00E+00	3.26 (2.85-3.66)	0.00E+00	0.00E+00	2.63 (2.03-3.23)	0.00E+00	3.40E-15

CSMD2	8	3.92 (3.17-4.68)	0.00E+00	0.00E+00	3.56 (2.85-4.28)	0.00E+00	0.00E+00			
CSMD3	6	2.43 (1.61-3.25)	5.91E-09	3.55E-06	1.94 (1.13-2.75)	2.44E-06	1.53E-03	2.74 (1.48-4.01)	2.18E-05	8.70E-03
CTC1	2	3.73 (2.26-5.21)	6.84E-07	4.11E-04	4.04 (2.60-5.49)	3.83E-08	2.41E-05			
CYLD	32	3.55 (3.17-3.92)	0.00E+00	0.00E+00	3.28 (2.92-3.64)	0.00E+00	0.00E+00	3.76 (2.98-4.54)	0.00E+00	0.00E+00
DGKG	6	3.92 (3.06-4.79)	0.00E+00	4.00E-16	3.53 (2.70-4.35)	1.00E-16	3.45E-14			
DHX37	42	3.16 (2.83-3.50)	0.00E+00	0.00E+00	2.93 (2.61-3.26)	0.00E+00	0.00E+00	2.96 (2.45-3.47)	0.00E+00	0.00E+00
DHX38	13	2.88 (2.31-3.45)	0.00E+00	0.00E+00	2.66 (2.10-3.22)	0.00E+00	0.00E+00	1.67 (0.99-2.34)	1.25E-06	4.98E-04
DNAJB8	10	6.49 (5.41-7.57)	0.00E+00	0.00E+00	6.11 (5.32-6.91)	0.00E+00	0.00E+00			
DSC2	19	3.37 (2.90-3.85)	0.00E+00	0.00E+00	3.14 (2.67-3.60)	0.00E+00	0.00E+00			
DST	27	5.24 (4.75-5.72)	0.00E+00	0.00E+00	4.81 (4.39-5.23)	0.00E+00	0.00E+00			
DYNLT2B	10	2.58 (1.93-3.22)	4.00E-15	2.38E-12	2.15 (1.52-2.79)	2.48E-11	1.56E-08	1.44 (0.70-2.18)	1.44E-04	5.77E-02
EEPD1	13	1.52 (0.97-2.08)	7.64E-08	4.59E-05	1.38 (0.83-1.93)	9.54E-07	6.00E-04	1.51 (0.86-2.17)	6.14E-06	2.45E-03
ENTPD2	38	2.62 (2.29-2.95)	0.00E+00	0.00E+00	2.59 (2.27-2.92)	0.00E+00	0.00E+00	1.57 (1.19-1.96)	1.80E-15	7.34E-13
EPPK1	8	1.88 (1.17-2.59)	2.39E-07	1.44E-04	2.00 (1.30-2.71)	2.59E-08	1.63E-05	2.81 (1.69-3.93)	8.52E-07	3.40E-04
ETNPPL	2	3.68 (2.21-5.15)	9.73E-07	5.85E-04	4.36 (2.90-5.83)	5.15E-09	3.24E-06	3.04 (0.63-5.44)	1.34E-02	1.00E+00
EVPL	26	5.86 (5.30-6.42)	0.00E+00	0.00E+00	5.00 (4.57-5.44)	0.00E+00	0.00E+00			
F10	4	2.66 (1.65-3.67)	2.42E-07	1.45E-04	2.61 (1.61-3.60)	2.84E-07	1.79E-04	3.73 (1.54-5.92)	8.62E-04	3.44E-01
FAM91A1	2	4.82 (3.17-6.46)	9.58E-09	5.76E-06	4.74 (3.24-6.24)	6.49E-10	4.08E-07			
FGFR3	4	2.86 (1.84-3.87)	3.25E-08	1.95E-05	2.90 (1.91-3.90)	1.15E-08	7.25E-06	2.63 (1.13-4.13)	5.94E-04	2.37E-01
FOXK2	5	4.15 (3.19-5.12)	0.00E+00	1.99E-14	3.58 (2.67-4.48)	9.00E-15	5.66E-12			
FRAS1	4	2.89 (1.88-3.90)	2.13E-08	1.28E-05	2.51 (1.51-3.50)	7.45E-07	4.68E-04			
GDF11	6	6.44 (5.05-7.83)	0.00E+00	1.00E-16	5.43 (4.49-6.36)	0.00E+00	0.00E+00			
GPAT3	36	3.02 (2.67-3.36)	0.00E+00	0.00E+00	2.90 (2.57-3.24)	0.00E+00	0.00E+00	1.88 (1.46-2.30)	0.00E+00	9.00E-16
GPC2	8	3.79 (3.04-4.54)	0.00E+00	0.00E+00	3.49 (2.76-4.21)	0.00E+00	0.00E+00	1.96 (1.06-2.87)	2.21E-05	8.81E-03
GPR176	4	2.56 (1.55-3.57)	6.25E-07	3.76E-04	2.41 (1.41-3.40)	2.07E-06	1.30E-03			
GPRC5D	5	1.50 (0.61-2.39)	9.32E-04	5.60E-01	2.08 (1.20-2.97)	4.19E-06	2.64E-03	1.55 (0.49-2.62)	4.12E-03	1.00E+00
GREB1L	11	4.14 (3.48-4.80)	0.00E+00	0.00E+00	3.76 (3.15-4.38)	0.00E+00	0.00E+00	3.37 (2.22-4.52)	8.85E-09	3.53E-06
GRIA3	5	4.64 (3.60-5.69)	0.00E+00	1.90E-15	4.49 (3.54-5.44)	0.00E+00	0.00E+00			
GRIN3B	14	6.63 (5.67-7.59)	0.00E+00	0.00E+00	5.72 (5.09-6.35)	0.00E+00	0.00E+00	2.92 (2.04-3.79)	5.89E-11	2.35E-08
H1-10	12	2.83 (2.24-3.42)	0.00E+00	0.00E+00	2.65 (2.06-3.23)	0.00E+00	3.00E-16	1.95 (1.22-2.69)	2.10E-07	8.38E-05
HK3	2	4.53 (2.96-6.11)	1.71E-08	1.03E-05	4.78 (3.28-6.28)	4.13E-10	2.60E-07			

HLA-DQA1	4	3.00 (1.96-4.04)	1.70E-08	1.02E-05	3.40 (2.38-4.43)	7.96E-11	5.01E-08	0.90 (-0.20-2.00)	1.09E-01	1.00E+00
HTR3B	12	3.20 (2.61-3.79)	0.00E+00	0.00E+00	2.82 (2.24-3.39)	0.00E+00	0.00E+00	1.17 (0.51-1.82)	4.54E-04	1.81E-01
IFI44	3	4.94 (3.58-6.29)	9.30E-13	5.59E-10	4.18 (2.99-5.36)	4.88E-12	3.07E-09			
IGFN1	8	3.82 (3.08-4.57)	0.00E+00	0.00E+00	3.60 (2.89-4.32)	0.00E+00	0.00E+00	1.65 (0.80-2.50)	1.49E-04	5.96E-02
ITGA10	3	3.27 (2.10-4.45)	5.17E-08	3.11E-05	3.36 (2.21-4.52)	1.18E-08	7.43E-06			
ITSN1	12	2.08 (1.50-2.66)	2.17E-12	1.31E-09	1.91 (1.33-2.48)	7.97E-11	5.01E-08	1.95 (1.21-2.68)	2.17E-07	8.65E-05
JPH2	8	2.94 (2.22-3.67)	1.50E-15	9.20E-13	2.90 (2.19-3.61)	1.20E-15	7.61E-13	1.09 (0.30-1.89)	6.83E-03	1.00E+00
KCNT2	2	4.52 (2.94-6.09)	1.86E-08	1.12E-05	4.04 (2.60-5.48)	4.12E-08	2.59E-05			
KDM4A	9	3.33 (2.64-4.01)	0.00E+00	0.00E+00	2.93 (2.26-3.59)	0.00E+00	5.40E-15	2.75 (1.72-3.79)	1.93E-07	7.71E-05
KIAA1109	5	3.11 (2.19-4.04)	4.25E-11	2.55E-08	3.02 (2.12-3.93)	5.42E-11	3.41E-08	2.58 (1.26-3.90)	1.32E-04	5.28E-02
KIR2DL3	4	2.78 (1.77-3.79)	7.22E-08	4.34E-05	2.06 (1.07-3.05)	4.40E-05	2.77E-02	0.39 (-0.66-1.44)	4.68E-01	1.00E+00
KLHL36	4	4.45 (3.34-5.56)	3.00E-15	1.80E-12	4.04 (3.02-5.06)	8.40E-15	5.27E-12			
LFNG	6	5.32 (4.30-6.34)	0.00E+00	0.00E+00	4.95 (4.07-5.84)	0.00E+00	0.00E+00			
LHX2	16	4.72 (4.13-5.30)	0.00E+00	0.00E+00	3.91 (3.40-4.43)	0.00E+00	0.00E+00	4.03 (2.80-5.27)	1.59E-10	6.33E-08
LYG1	2	2.26 (0.85-3.67)	1.70E-03	1.02E+00	2.88 (1.47-4.29)	6.32E-05	3.97E-02	1.09 (-0.49-2.67)	1.77E-01	1.00E+00
MANBA	6	5.05 (4.08-6.03)	0.00E+00	0.00E+00	4.73 (3.86-5.60)	0.00E+00	0.00E+00			
MAPK3	9	4.67 (3.91-5.42)	0.00E+00	0.00E+00	4.40 (3.70-5.09)	0.00E+00	0.00E+00			
MAPKAPK3	6	5.81 (4.67-6.94)	0.00E+00	0.00E+00	5.45 (4.52-6.37)	0.00E+00	0.00E+00			
MAS1L	8	3.52 (2.78-4.26)	0.00E+00	0.00E+00	3.36 (2.64-4.07)	0.00E+00	0.00E+00			
MEPE	2	2.43 (1.02-3.85)	7.62E-04	4.58E-01	2.85 (1.44-4.26)	7.25E-05	4.56E-02	0.95 (-0.60-2.51)	2.30E-01	1.00E+00
MERTK	38	2.95 (2.62-3.28)	0.00E+00	0.00E+00	2.89 (2.57-3.22)	0.00E+00	0.00E+00			
MS4A12	4	1.60 (0.61-2.59)	1.58E-03	9.47E-01	2.09 (1.10-3.08)	3.40E-05	2.14E-02	2.34 (0.95-3.73)	9.56E-04	3.81E-01
MUC16	2	6.51 (4.10-8.91)	1.11E-07	6.70E-05	3.14 (1.73-4.55)	1.27E-05	7.96E-03	2.34 (0.37-4.30)	1.96E-02	1.00E+00
MUC5AC	7	2.77 (2.00-3.54)	1.70E-12	1.02E-09	2.57 (1.82-3.33)	2.41E-11	1.51E-08			
NAP1L3	14	3.59 (3.02-4.16)	0.00E+00	0.00E+00	3.37 (2.82-3.92)	0.00E+00	0.00E+00	3.06 (2.15-3.97)	5.09E-11	2.03E-08
NINL	2	5.81 (3.85-7.78)	6.42E-09	3.86E-06	6.61 (4.65-8.57)	4.07E-11	2.56E-08			
NOL4	4	5.24 (4.01-6.47)	1.00E-16	4.28E-14	4.94 (3.86-6.01)	0.00E+00	1.00E-16			
NRXN2	2	5.78 (3.82-7.74)	7.88E-09	4.74E-06	6.17 (4.37-7.96)	1.55E-11	9.74E-09			
NT5C1A	21	4.37 (3.88-4.86)	0.00E+00	0.00E+00	3.79 (3.34-4.24)	0.00E+00	0.00E+00	1.91 (1.36-2.47)	1.32E-11	5.27E-09
OGFOD3	3	3.21 (2.03-4.39)	1.01E-07	6.09E-05	2.94 (1.78-4.09)	6.05E-07	3.80E-04	3.45 (1.18-5.71)	2.90E-03	1.00E+00
OLA1	7	3.68 (2.89-4.47)	0.00E+00	0.00E+00	3.72 (2.95-4.48)	0.00E+00	0.00E+00			

OR14A2	25	3.92 (3.49-4.35)	0.00E+00	0.00E+00	3.56 (3.15-3.97)	0.00E+00	0.00E+00	2.94 (2.29-3.60)	0.00E+00	8.00E-16
OR4D1	9	2.56 (1.89-3.24)	8.38E-14	5.04E-11	2.36 (1.70-3.03)	3.01E-12	1.89E-09	3.45 (2.14-4.76)	2.48E-07	9.90E-05
OR4F6	12	4.92 (4.24-5.59)	0.00E+00	0.00E+00	4.63 (4.02-5.23)	0.00E+00	0.00E+00			
OR5M9	72	3.62 (3.35-3.88)	0.00E+00	0.00E+00	3.41 (3.15-3.67)	0.00E+00	0.00E+00	3.19 (2.78-3.61)	0.00E+00	0.00E+00
OR8G1	2	6.27 (3.87-8.68)	3.20E-07	1.92E-04	5.88 (4.08-7.67)	1.36E-10	8.56E-08	1.94 (0.14-3.73)	3.45E-02	1.00E+00
PAK5	19	5.11 (4.55-5.68)	0.00E+00	0.00E+00	4.79 (4.30-5.29)	0.00E+00	0.00E+00			
PCSK4	2				5.23 (3.67-6.78)	4.50E-11	2.83E-08			
PCSK6	10	3.04 (2.39-3.69)	0.00E+00	0.00E+00	2.78 (2.14-3.41)	0.00E+00	5.60E-15			
PHACTR4	19	2.73 (2.26-3.20)	0.00E+00	0.00E+00	2.58 (2.12-3.05)	0.00E+00	0.00E+00	2.01 (1.42-2.60)	3.10E-11	1.24E-08
PHOX2B	14	2.71 (2.17-3.26)	0.00E+00	0.00E+00	2.60 (2.06-3.14)	0.00E+00	0.00E+00	2.01 (1.32-2.70)	1.24E-08	4.94E-06
PIK3R6	14	2.28 (1.74-2.81)	1.00E-16	6.40E-14	2.14 (1.61-2.67)	3.00E-15	1.91E-12	2.42 (1.67-3.18)	3.79E-10	1.51E-07
PKD2	10	3.81 (3.14-4.48)	0.00E+00	0.00E+00	3.75 (3.11-4.40)	0.00E+00	0.00E+00			
PLA2R1	2	3.17 (1.74-4.61)	1.52E-05	9.16E-03	3.50 (2.08-4.92)	1.40E-06	8.81E-04	1.64 (-0.06-3.34)	5.81E-02	1.00E+00
PLD2	3	3.96 (2.73-5.19)	2.87E-10	1.72E-07	3.42 (2.25-4.58)	8.91E-09	5.61E-06			
POTEA	4	2.04 (1.05-3.04)	5.80E-05	3.48E-02	2.09 (1.10-3.08)	3.64E-05	2.29E-02	0.95 (-0.15-2.05)	8.97E-02	1.00E+00
PRDM2	40	3.13 (2.80-3.46)	0.00E+00	0.00E+00	3.01 (2.68-3.33)	0.00E+00	0.00E+00	2.61 (2.13-3.08)	0.00E+00	0.00E+00
PRLR	12	2.62 (2.03-3.20)	0.00E+00	9.00E-16	2.70 (2.13-3.28)	0.00E+00	0.00E+00	3.04 (2.06-4.02)	1.32E-09	5.28E-07
PROM2	4	4.32 (3.22-5.41)	9.20E-15	5.52E-12	3.90 (2.88-4.91)	5.72E-14	3.60E-11			
PTCH2	2	5.81 (3.85-7.78)	6.43E-09	3.87E-06	6.61 (4.65-8.57)	4.07E-11	2.56E-08	1.93 (0.14-3.72)	3.46E-02	1.00E+00
RAB25	2	2.87 (1.44-4.30)	8.14E-05	4.89E-02	3.46 (2.04-4.88)	1.89E-06	1.19E-03	1.42 (-0.22-3.07)	8.99E-02	1.00E+00
RAB2A	8	3.53 (2.79-4.27)	0.00E+00	0.00E+00	3.35 (2.64-4.07)	0.00E+00	0.00E+00	2.64 (1.58-3.70)	1.15E-06	4.58E-04
RNF111	47	4.18 (3.85-4.51)	0.00E+00	0.00E+00	3.50 (3.20-3.81)	0.00E+00	0.00E+00	2.37 (1.96-2.78)	0.00E+00	0.00E+00
RNF123	2				6.59 (4.63-8.56)	4.55E-11	2.86E-08			
RNF168	3	5.53 (4.03-7.04)	5.41E-13	3.25E-10	3.03 (1.87-4.18)	2.86E-07	1.80E-04	3.45 (1.18-5.71)	2.90E-03	1.00E+00
ROS1	2	2.64 (1.22-4.06)	2.74E-04	1.65E-01	3.26 (1.85-4.68)	6.31E-06	3.97E-03	1.65 (-0.06-3.35)	5.80E-02	1.00E+00
RP1	2	2.68 (1.26-4.10)	2.19E-04	1.31E-01	3.35 (1.93-4.77)	3.51E-06	2.21E-03	3.03 (0.63-5.43)	1.34E-02	1.00E+00
RP1L1	3	2.82 (1.66-3.98)	2.06E-06	1.24E-03	2.46 (1.32-3.61)	2.48E-05	1.56E-02	1.24 (-0.07-2.55)	6.37E-02	1.00E+00
RPGRIP1L	2	6.49 (4.09-8.90)	1.18E-07	7.08E-05	5.68 (4.03-7.32)	1.26E-11	7.92E-09			
RPUSD2	32	1.74 (1.38-2.09)	0.00E+00	0.00E+00	1.70 (1.35-2.06)	0.00E+00	0.00E+00	2.44 (1.93-2.94)	0.00E+00	0.00E+00
SDK2	10	3.74 (3.07-4.40)	0.00E+00	0.00E+00	3.66 (3.01-4.30)	0.00E+00	0.00E+00			
SH3D21	2	3.01 (1.58-4.45)	3.81E-05	2.29E-02	3.59 (2.17-5.02)	7.88E-07	4.95E-04	3.04 (0.63-5.44)	1.34E-02	1.00E+00

SH3TC1	9	2.04 (1.38-2.70)	1.71E-09	1.03E-06	2.42 (1.75-3.08)	8.14E-13	5.12E-10	2.46 (1.50-3.41)	4.44E-07	1.77E-04
SIPA1L2	8	3.70 (2.96-4.44)	0.00E+00	0.00E+00	3.52 (2.80-4.23)	0.00E+00	0.00E+00	2.35 (1.37-3.34)	2.91E-06	1.16E-03
SLC12A9	2				3.84 (2.41-5.27)	1.50E-07	9.46E-05			
SLC22A11	2	5.81 (3.85-7.78)	6.43E-09	3.87E-06	6.61 (4.65-8.57)	4.08E-11	2.56E-08	1.64 (-0.06-3.34)	5.81E-02	1.00E+00
SLC5A9	32	2.38 (2.01-2.75)	0.00E+00	0.00E+00	2.30 (1.93-2.66)	0.00E+00	0.00E+00	2.66 (2.13-3.19)	0.00E+00	0.00E+00
SLC9A3	4	4.93 (3.75-6.11)	2.00E-16	1.50E-13	4.52 (3.48-5.57)	0.00E+00	1.40E-14	0.32 (-0.73-1.37)	5.49E-01	1.00E+00
SLCO4A1	29	3.80 (3.40-4.19)	0.00E+00	0.00E+00	3.50 (3.12-3.88)	0.00E+00	0.00E+00	3.02 (2.39-3.65)	0.00E+00	0.00E+00
SMPD1	2	5.64 (3.68-7.60)	1.80E-08	1.08E-05	6.45 (4.48-8.41)	1.21E-10	7.59E-08	2.34 (0.37-4.30)	1.96E-02	1.00E+00
SMYD4	2	3.33 (1.88-4.77)	6.46E-06	3.88E-03	4.01 (2.57-5.45)	4.90E-08	3.08E-05	2.34 (0.37-4.30)	1.96E-02	1.00E+00
SPTB	8	5.32 (4.42-6.22)	0.00E+00	0.00E+00	4.23 (3.49-4.96)	0.00E+00	0.00E+00			
STRN3	9	5.04 (4.23-5.84)	0.00E+00	0.00E+00	4.23 (3.54-4.93)	0.00E+00	0.00E+00	1.04 (0.30-1.79)	5.83E-03	1.00E+00
SYCP1	20	2.99 (2.53-3.45)	0.00E+00	0.00E+00	2.78 (2.33-3.23)	0.00E+00	0.00E+00			
SYNE1	33	3.12 (2.76-3.48)	0.00E+00	0.00E+00	3.09 (2.74-3.44)	0.00E+00	0.00E+00	3.15 (2.54-3.77)	0.00E+00	0.00E+00
SYNE2	6	4.96 (4.00-5.91)	0.00E+00	0.00E+00	4.44 (3.59-5.29)	0.00E+00	0.00E+00	1.64 (0.66-2.63)	1.03E-03	4.11E-01
SYNE3	16	5.74 (5.06-6.43)	0.00E+00	0.00E+00	5.64 (5.06-6.22)	0.00E+00	0.00E+00	3.33 (2.39-4.28)	3.85E-12	1.54E-09
TANC2	6	3.76 (2.90-4.62)	0.00E+00	6.70E-15	3.32 (2.50-4.15)	2.50E-15	1.59E-12			
TAS1R3	4	4.44 (3.34-5.55)	3.30E-15	1.98E-12	2.63 (1.63-3.62)	2.59E-07	1.63E-04	2.13 (0.81-3.45)	1.62E-03	6.46E-01
TECPR1	8	3.09 (2.36-3.81)	1.00E-16	4.57E-14	2.85 (2.14-3.56)	3.80E-15	2.38E-12			
TEX38	3	4.62 (3.32-5.92)	3.06E-12	1.84E-09	5.32 (4.04-6.61)	5.00E-16	2.94E-13	3.45 (1.18-5.71)	2.90E-03	1.16E+00
THAP9	2	6.51 (4.10-8.91)	1.11E-07	6.69E-05	7.30 (4.90-9.71)	2.56E-09	1.61E-06	3.03 (0.63-5.43)	1.34E-02	1.00E+00
TMC8	2	5.41 (3.61-7.20)	3.35E-09	2.01E-06	3.13 (1.72-4.54)	1.39E-05	8.72E-03	3.03 (0.63-5.43)	1.34E-02	1.00E+00
TMEM132D	21	4.05 (3.57-4.53)	0.00E+00	0.00E+00	3.50 (3.06-3.95)	0.00E+00	0.00E+00	2.27 (1.67-2.86)	9.28E-14	3.70E-11
TMEM232	4	4.12 (3.05-5.20)	5.59E-14	3.36E-11	3.52 (2.51-4.53)	8.98E-12	5.65E-09	2.64 (1.14-4.14)	5.82E-04	2.32E-01
TNFRSF10A	13	3.36 (2.79-3.93)	0.00E+00	0.00E+00	3.13 (2.57-3.69)	0.00E+00	0.00E+00	2.02 (1.31-2.74)	3.27E-08	1.31E-05
TRANK1	4	2.48 (1.48-3.49)	1.24E-06	7.47E-04	2.20 (1.21-3.19)	1.35E-05	8.50E-03	1.42 (0.26-2.59)	1.65E-02	1.00E+00
TRIM26	4	4.59 (3.47-5.72)	1.10E-15	6.47E-13	4.06 (3.04-5.08)	6.90E-15	4.35E-12			
TRPM4	3	2.20 (1.05-3.35)	1.75E-04	1.05E-01	2.69 (1.54-3.84)	4.19E-06	2.63E-03	1.64 (0.26-3.03)	2.03E-02	1.00E+00
TTPA	6	1.94 (1.12-2.75)	3.13E-06	1.88E-03	1.79 (0.99-2.60)	1.36E-05	8.57E-03	1.42 (0.47-2.38)	3.31E-03	1.00E+00
TYRO3	2				6.61 (4.65-8.58)	4.28E-11	2.69E-08			
UNC13C	44	3.27 (2.95-3.59)	0.00E+00	0.00E+00	3.16 (2.84-3.47)	0.00E+00	0.00E+00	2.53 (2.09-2.97)	0.00E+00	0.00E+00
VSIG8	10	4.10 (3.42-4.79)	0.00E+00	0.00E+00	3.66 (3.01-4.30)	0.00E+00	0.00E+00			

WFIKKN2	2	6.41 (4.00-8.81)	1.77E-07	1.07E-04	4.80 (3.29-6.31)	5.12E-10	3.22E-07
WNK2	65	3.38 (3.11-3.65)	0.00E+00	0.00E+00	3.16 (2.90-3.43)	0.00E+00	0.00E+00
XPNPEP2	8	3.54 (2.80-4.29)	0.00E+00	0.00E+00	3.57 (2.84-4.29)	0.00E+00	0.00E+00
ZBTB10	5	3.16 (2.25-4.08)	1.18E-11	7.08E-09	3.10 (2.20-3.99)	1.16E-11	7.32E-09
ZNF132	2	3.37 (1.92-4.83)	5.46E-06	3.28E-03	3.45 (2.03-4.87)	1.95E-06	1.23E-03
ZPR1	9	5.49 (4.62-6.36)	0.00E+00	0.00E+00	5.40 (4.65-6.15)	0.00E+00	0.00E+00
ZSCAN5A	2	5.77 (3.81-7.73)	8.20E-09	4.93E-06	5.31 (3.74-6.88)	3.87E-11	2.43E-08
ZZEF1	20	4.65 (4.14-5.16)	0.00E+00	0.00E+00	4.24 (3.77-4.70)	0.00E+00	0.00E+00

Gene	N Variants	logOR (CI) NFE	pvalue	pvalue corrected	logOR (CI) ALL	pvalue	pvalue corrected	logOR (CI) Swedish	pvalue	pvalue corrected
ACSF2	2	5.30 (3.76-6.84)	1.56E-11	5.66E-09	5.30 (3.83-6.76)	1.31E-12	4.97E-10			
ANKRD27	15	4.49 (3.94-5.04)	0.00E+00	0.00E+00	4.03 (3.50-4.56)	0.00E+00	0.00E+00	5.47 (3.99-6.95)	4.66E-13	1.2213E-10
ARHGAP40	2	3.00 (1.58-4.42)	3.43E-05	1.25E-02	3.50 (2.08-4.92)	1.29E-06	4.90E-04	2.15 (0.56-3.75)	7.92E-03	1
ATP7B	17	3.73 (3.22-4.24)	0.00E+00	0.00E+00	3.34 (2.84-3.84)	0.00E+00	0.00E+00	3.41 (2.73-4.09)	0.00E+00	0
BCL7C	2	4.26 (2.81-5.71)	8.39E-09	3.05E-06	3.88 (2.47-5.29)	7.39E-08	2.80E-05			
CCDC66	4	1.73 (0.74-2.72)	6.34E-04	2.30E-01	2.16 (1.17-3.15)	1.92E-05	7.27E-03	1.23 (0.18-2.27)	2.13E-02	1
CEP131	4	4.80 (3.69-5.90)	0.00E+00	6.80E-15	4.15 (3.12-5.19)	3.60E-15	1.37E-12	4.13 (2.42-5.85)	2.32E-06	0.00060821
CFAP251	2	6.18 (4.47-7.88)	1.27E-12	4.61E-10	6.57 (4.96-8.18)	1.20E-15	4.73E-13			
CNTLN	3	2.28 (1.13-3.43)	9.99E-05	3.63E-02	2.66 (1.51-3.81)	5.46E-06	2.07E-03	2.01 (0.74-3.29)	1.99E-03	0.52088511
COLGALT1	6	3.16 (2.34-3.99)	5.17E-14	1.88E-11	2.86 (2.05-3.68)	6.09E-12	2.31E-09	2.26 (1.33-3.18)	1.94E-06	0.00050742
CRYBA4	3	2.00 (0.83-3.16)	7.55E-04	2.74E-01	2.55 (1.39-3.71)	1.68E-05	6.37E-03	2.91 (1.46-4.37)	8.46E-05	0.0221698
CSMD2	6	4.71 (3.84-5.57)	0.00E+00	0.00E+00	4.35 (3.52-5.18)	0.00E+00	0.00E+00			
CYLD	12	3.62 (3.03-4.21)	0.00E+00	0.00E+00	3.35 (2.77-3.94)	0.00E+00	0.00E+00	3.83 (2.93-4.73)	1.00E-16	2.42E-14
DACT2	6	3.07 (2.24-3.90)	3.77E-13	1.37E-10	2.77 (1.94-3.59)	4.30E-11	1.63E-08	1.42 (0.55-2.29)	1.36E-03	0.35588518
DGKG	2	4.23 (2.78-5.69)	1.17E-08	4.23E-06	3.90 (2.48-5.33)	7.44E-08	2.82E-05			
DMBT1	2	4.52 (3.06-5.98)	1.34E-09	4.87E-07	3.60 (2.20-5.01)	5.26E-07	2.00E-04	1.69 (0.17-3.21)	2.91E-02	1
DNAH1	9	2.12 (1.45-2.79)	4.96E-10	1.80E-07	1.94 (1.27-2.60)	1.17E-08	4.45E-06	1.17 (0.47-1.87)	1.02E-03	0.2659361
DNAH7	3	2.68 (1.53-3.84)	5.07E-06	1.84E-03	2.84 (1.69-3.98)	1.31E-06	4.95E-04	3.81 (2.01-5.61)	3.28E-05	0.00860224
DNAJB8	6	7.05 (5.85-8.24)	0.00E+00	0.00E+00	6.67 (5.72-7.62)	0.00E+00	0.00E+00			
DST	7	4.78 (3.99-5.58)	0.00E+00	0.00E+00	4.38 (3.62-5.15)	0.00E+00	0.00E+00			
DYNLT2B	6	3.13 (2.30-3.96)	1.37E-13	4.98E-11	2.71 (1.89-3.53)	1.02E-10	3.88E-08	1.99 (1.09-2.90)	1.69E-05	0.00442687
EEPD1	9	2.23 (1.55-2.90)	8.86E-11	3.22E-08	2.08 (1.41-2.75)	1.10E-09	4.17E-07	2.22 (1.46-2.98)	1.00E-08	2.6219E-06
ENTPD2	10	2.44 (1.81-3.07)	3.57E-14	1.30E-11	2.46 (1.83-3.09)	2.04E-14	7.73E-12	1.44 (0.77-2.11)	2.48E-05	0.00651061
FAM135A	3	2.79 (1.62-3.96)	2.82E-06	1.02E-03	2.75 (1.59-3.91)	3.56E-06	1.35E-03	3.43 (1.81-5.04)	3.39E-05	0.00887722
FAT2	4	8.27 (6.07-10.47)	1.64E-13	5.95E-11	7.68 (6.29-9.08)	0.00E+00	0.00E+00			
GCKR	2	2.96 (1.55-4.37)	3.92E-05	1.42E-02	3.23 (1.83-4.63)	6.53E-06	2.47E-03			
GDF11	6	7.51 (6.11-8.90)	0.00E+00	0.00E+00	6.09 (5.19-6.99)	0.00E+00	0.00E+00			
GPAT3	16	3.27 (2.75-3.78)	0.00E+00	0.00E+00	3.16 (2.65-3.66)	0.00E+00	0.00E+00	2.13 (1.56-2.70)	1.70E-13	4.4422E-11
GRIA3	5	5.70 (4.65-6.75)	0.00E+00	0.00E+00	5.55 (4.59-6.50)	0.00E+00	0.00E+00			
HLA-DQA1	3	3.81 (2.62-5.00)	3.32E-10	1.21E-07	4.17 (2.99-5.34)	3.45E-12	1.31E-09	1.61 (0.37-2.84)	1.07E-02	1

HTR3B	4	3.15 (2.15-4.15)	6.82E-10	2.48E-07	2.76 (1.77-3.76)	4.64E-08	1.76E-05	1.11 (0.08-2.15)	3.51E-02	1
HTT	10	1.44 (0.82-2.07)	6.13E-06	2.22E-03	1.55 (0.93-2.18)	1.16E-06	4.40E-04	1.05 (0.40-1.70)	1.65E-03	0.43232262
IGLL1	2	2.34 (0.93-3.74)	1.10E-03	3.98E-01	2.82 (1.42-4.22)	7.92E-05	3.00E-02	1.69 (0.17-3.21)	2.91E-02	7.61472545
ITGA10	2	3.95 (2.52-5.39)	6.43E-08	2.33E-05	4.04 (2.63-5.46)	2.12E-08	8.03E-06			
KDM4A	6	3.98 (3.15-4.82)	0.00E+00	0.00E+00	3.58 (2.76-4.40)	0.00E+00	4.00E-15	3.41 (2.27-4.55)	4.60E-09	1.2052E-06
KIAA1109	3	3.67 (2.48-4.85)	1.34E-09	4.85E-07	3.58 (2.41-4.75)	1.96E-09	7.43E-07	3.14 (1.62-4.65)	5.07E-05	0.01328945
LAMC2	2	3.55 (2.13-4.97)	1.02E-06	3.69E-04	3.39 (1.98-4.80)	2.30E-06	8.71E-04			
LGR4	2	7.54 (5.14-9.95)	8.10E-10	2.94E-07	7.64 (5.68-9.61)	2.64E-14	1.00E-11			
LHX2	9	5.21 (4.47-5.94)	0.00E+00	0.00E+00	4.40 (3.72-5.09)	0.00E+00	0.00E+00	4.52 (3.21-5.84)	1.51E-11	3.958E-09
MAPK3	9	5.74 (4.98-6.51)	0.00E+00	0.00E+00	5.48 (4.77-6.18)	0.00E+00	0.00E+00			
MERTK	6	2.69 (1.87-3.51)	1.20E-10	4.37E-08	2.63 (1.82-3.45)	2.49E-10	9.45E-08			
MPHOSPH10	5	4.34 (3.40-5.28)	0.00E+00	1.00E-16	4.11 (3.19-5.04)	0.00E+00	9.00E-16			
NAP1L3	8	4.10 (3.36-4.84)	0.00E+00	0.00E+00	3.88 (3.16-4.61)	0.00E+00	0.00E+00	3.57 (2.54-4.60)	1.02E-11	2.6734E-09
NINL	2	6.87 (4.90-8.84)	7.62E-12	2.77E-09	7.67 (5.70-9.64)	2.18E-14	8.25E-12			
NRXN2	2	6.84 (4.87-8.80)	9.69E-12	3.52E-09	7.22 (5.43-9.02)	3.30E-15	1.26E-12			
NT5C1A	2	3.64 (2.21-5.07)	6.14E-07	2.23E-04	2.93 (1.53-4.34)	4.17E-05	1.58E-02	0.99 (-0.46-2.45)	1.82E-01	1
OLA1	4	4.72 (3.67-5.77)	0.00E+00	3.00E-16	4.70 (3.68-5.71)	0.00E+00	0.00E+00			
OR4F21	3	2.68 (1.46-3.91)	1.80E-05	6.52E-03	2.72 (1.55-3.90)	5.75E-06	2.18E-03	1.47 (0.24-2.70)	1.96E-02	1
OR5M9	16	3.39 (2.82-3.96)	0.00E+00	0.00E+00	3.20 (2.63-3.76)	0.00E+00	0.00E+00	3.47 (2.75-4.19)	0.00E+00	0
OTOP1	2	3.57 (2.15-5.00)	8.29E-07	3.01E-04	4.17 (2.75-5.59)	7.98E-09	3.03E-06	4.09 (1.68-6.50)	8.68E-04	0.22738027
PAK5	7	4.82 (4.02-5.62)	0.00E+00	0.00E+00	4.53 (3.76-5.29)	0.00E+00	0.00E+00			
PASK	2	3.39 (1.97-4.81)	2.77E-06	1.01E-03	3.75 (2.34-5.16)	1.91E-07	7.24E-05	2.70 (1.00-4.41)	1.91E-03	0.49996214
PCDH10	4	3.69 (2.66-4.71)	1.57E-12	5.70E-10	3.39 (2.39-4.39)	3.65E-11	1.38E-08			
PIK3R6	12	2.96 (2.38-3.54)	0.00E+00	0.00E+00	2.83 (2.25-3.41)	0.00E+00	0.00E+00	3.01 (2.27-3.75)	1.40E-15	3.628E-13
PLIN4	4	4.56 (3.51-5.61)	0.00E+00	7.70E-15	2.99 (1.99-3.99)	4.75E-09	1.80E-06			
PRDM2	20	3.50 (3.04-3.97)	0.00E+00	0.00E+00	3.39 (2.93-3.85)	0.00E+00	0.00E+00	2.98 (2.41-3.56)	0.00E+00	0
PROM2	4	5.37 (4.28-6.47)	0.00E+00	0.00E+00	4.95 (3.93-5.98)	0.00E+00	0.00E+00			
QTRT2	2	6.89 (4.91-8.86)	8.25E-12	3.00E-09	6.77 (5.11-8.42)	1.20E-15	4.67E-13	2.49 (0.83-4.15)	3.22E-03	0.84261163
RHOB	4	6.30 (5.06-7.54)	0.00E+00	0.00E+00	5.99 (4.91-7.08)	0.00E+00	0.00E+00			
SCN5A	4				7.65 (6.26-9.04)	0.00E+00	0.00E+00			
SDK2	6	4.29 (3.45-5.14)	0.00E+00	0.00E+00	4.22 (3.38-5.05)	0.00E+00	0.00E+00			

SH3TC1	3	2.08 (0.91-3.24)	4.64E-04	1.68E-01	2.47 (1.31-3.63)	2.93E-05	1.11E-02	2.44 (1.09-3.79)	3.92E-04	0.102603
SHANK1	4	6.78 (5.39-8.17)	0.00E+00	0.00E+00	6.25 (5.14-7.36)	0.00E+00	0.00E+00			
SLC12A9	2				4.90 (3.46-6.33)	2.56E-11	9.71E-09			
SLC49A3	3	5.04 (3.80-6.29)	2.30E-15	8.21E-13	4.59 (3.42-5.76)	1.63E-14	6.18E-12			
SLCO4A1	16	4.28 (3.75-4.80)	0.00E+00	0.00E+00	3.98 (3.47-4.49)	0.00E+00	0.00E+00	4.02 (3.19-4.84)	0.00E+00	0
SPTB	2	4.98 (3.47-6.48)	9.53E-11	3.46E-08	3.88 (2.47-5.30)	7.18E-08	2.72E-05			
SYCP1	8	3.19 (2.48-3.91)	0.00E+00	7.00E-16	3.00 (2.29-3.70)	1.00E-16	4.02E-14			
SYNE3	8	6.11 (5.26-6.96)	0.00E+00	0.00E+00	6.00 (5.24-6.77)	0.00E+00	0.00E+00	3.70 (2.63-4.76)	1.03E-11	2.6991E-09
TAF3	5	4.20 (3.29-5.12)	0.00E+00	1.00E-16	3.93 (3.03-4.82)	0.00E+00	2.90E-15			
TANC2	2	3.71 (2.28-5.14)	3.55E-07	1.29E-04	3.27 (1.87-4.68)	5.10E-06	1.93E-03			
TAS1R3	2	4.81 (3.32-6.30)	2.56E-10	9.31E-08	2.99 (1.58-4.40)	3.36E-05	1.27E-02	2.49 (0.83-4.15)	3.22E-03	0.84261163
TMEM132D	13	4.64 (4.05-5.23)	0.00E+00	0.00E+00	4.09 (3.53-4.66)	0.00E+00	0.00E+00	2.86 (2.17-3.55)	5.00E-16	1.434E-13
TMEM232	3	4.91 (3.68-6.14)	5.40E-15	1.98E-12	4.31 (3.13-5.48)	7.06E-13	2.68E-10	3.43 (1.81-5.04)	3.39E-05	0.00887722
TMEM41A	8	3.44 (2.71-4.17)	0.00E+00	0.00E+00	3.17 (2.45-3.89)	0.00E+00	2.00E-15	1.73 (0.95-2.50)	1.22E-05	0.00320478
TNFRSF10A	6	4.22 (3.37-5.07)	0.00E+00	0.00E+00	3.89 (3.06-4.72)	0.00E+00	0.00E+00	2.73 (1.73-3.73)	7.62E-08	1.9955E-05
TOGARAM2	3	2.01 (0.86-3.16)	6.02E-04	2.19E-01	2.41 (1.27-3.56)	3.75E-05	1.42E-02	1.55 (0.32-2.78)	1.33E-02	1
TRIM26	2	5.73 (4.12-7.33)	3.11E-12	1.13E-09	5.22 (3.76-6.68)	2.24E-12	8.48E-10			
TUT4	15	2.91 (2.38-3.45)	0.00E+00	0.00E+00	2.89 (2.36-3.42)	0.00E+00	0.00E+00	1.67 (1.11-2.24)	6.94E-09	1.8176E-06
VSIG8	2	3.53 (2.11-4.95)	1.16E-06	4.21E-04	3.08 (1.68-4.49)	1.66E-05	6.30E-03			
WDHD1	3	2.91 (1.74-4.08)	1.07E-06	3.90E-04	2.80 (1.63-3.96)	2.45E-06	9.29E-04	2.44 (1.09-3.79)	3.92E-04	0.102603
WHRN	3				6.97 (5.58-8.36)	0.00E+00	0.00E+00	4.49 (2.23-6.76)	1.02E-04	0.02681021
WNK2	9	3.91 (3.19-4.63)	0.00E+00	0.00E+00	3.54 (2.83-4.24)	0.00E+00	0.00E+00			
ZBTB10	2	3.73 (2.30-5.15)	3.10E-07	1.13E-04	3.65 (2.24-5.06)	3.77E-07	1.43E-04			
ZNF577	2	3.67 (2.23-5.10)	5.40E-07	1.96E-04	3.41 (2.00-4.83)	2.34E-06	8.88E-04	1.80 (0.26-3.33)	2.20E-02	1
ZPR1	4	5.73 (4.59-6.88)	0.00E+00	0.00E+00	5.64 (4.58-6.70)	0.00E+00	0.00E+00			

Supplementary table 2b. Genes with enrichment in missense variants in TIGER (columns A-K) and SEVTIN (columns M-W) cohorts. Odds ratio (OR) were calculated for the allelic frequencies reported in Non-Finish European (NFE) from gnomAD, all individuals form gnomAD and Swegen. If no LoF variants was reported in NFE or Swegen cohorts, OR cannot be calculated and cells are empty. Multiple hypothesis testing correction for each pvalue have been addressed by False Discovery Rate (FDR) approach for the total number of genes and total number of variants for each comparison.

Gene	N Variants	logOR (CI) NFE	pvalue	pvalue corrected	logOR (CI) ALL	pvalue	pvalue corrected	logOR (CI) Swedish	pvalue	pvalue corrected
AANAT	2	2.82 (1.39-4.25)	1.07E-04	4.80E-01	3.34 (1.92-4.76)	4.03E-06	1.87E-02	1.42 (-0.22-3.07)	8.99E-02	1.00E+00
AATK	6	1.70 (0.89-2.51)	3.88E-05	1.74E-01	2.13 (1.32-2.93)	2.41E-07	1.12E-03	2.05 (0.99-3.11)	1.51E-04	5.11E-01
ABCA3	2	3.73 (2.26-5.21)	6.84E-07	3.07E-03	4.41 (2.95-5.88)	3.50E-09	1.63E-05	3.03 (0.63-5.43)	1.34E-02	1.00E+00
ACSBG2	2	2.89 (1.47-4.32)	6.96E-05	3.13E-01	3.21 (1.80-4.62)	8.50E-06	3.95E-02	3.03 (0.63-5.43)	1.34E-02	1.00E+00
ACTR5	4	2.06 (1.06-3.06)	5.60E-05	2.51E-01	2.66 (1.66-3.66)	1.78E-07	8.28E-04	1.79 (0.55-3.03)	4.56E-03	1.00E+00
ADAM28	4	5.41 (4.14-6.68)	1.00E-16	2.79E-13	4.60 (3.55-5.64)	0.00E+00	3.50E-14	2.12 (0.80-3.43)	1.65E-03	1.00E+00
ADAMTS17	2	3.67 (2.20-5.14)	9.80E-07	4.40E-03	3.97 (2.53-5.40)	6.47E-08	3.01E-04			
ADAMTSL4	2	3.94 (2.45-5.43)	2.26E-07	1.02E-03	4.12 (2.68-5.57)	2.25E-08	1.05E-04			
ADGRV1	14	0.83 (0.31-1.36)	1.98E-03	1.00E+00	1.19 (0.66-1.71)	1.03E-05	4.79E-02	0.93 (0.35-1.52)	1.80E-03	1.00E+00
AEBP2	4	2.95 (1.94-3.96)	1.07E-08	4.79E-05	2.92 (1.92-3.92)	8.97E-09	4.17E-05	1.78 (0.55-3.01)	4.63E-03	1.00E+00
AFAP1	2	5.81 (3.85-7.77)	6.45E-09	2.90E-05	6.20 (4.41-8.00)	1.16E-11	5.41E-08			
AHNAK	6	2.77 (1.95-3.59)	4.10E-11	1.84E-07	3.14 (2.32-3.96)	4.77E-14	2.22E-10	1.57 (0.60-2.54)	1.55E-03	1.00E+00
AKAP12	9	1.29 (0.63-1.95)	1.31E-04	5.90E-01	1.67 (1.01-2.33)	6.54E-07	3.04E-03	1.40 (0.63-2.18)	3.80E-04	1.00E+00
AKAP13	3	3.15 (1.98-4.32)	1.43E-07	6.41E-04	3.35 (2.19-4.51)	1.34E-08	6.22E-05			
ALKBH1	2	4.71 (3.11-6.32)	8.29E-09	3.73E-05	5.00 (3.48-6.52)	1.18E-10	5.50E-07			
AMER1	3	3.24 (2.05-4.42)	9.71E-08	4.36E-04	3.83 (2.65-5.01)	2.14E-10	9.96E-07	2.05 (0.55-3.55)	7.36E-03	1.00E+00
AMOTL2	3	3.62 (2.42-4.82)	3.14E-09	1.41E-05	4.31 (3.12-5.50)	1.31E-12	6.11E-09	3.44 (1.17-5.70)	2.93E-03	1.00E+00
ANKS1A	2	3.87 (2.38-5.35)	3.34E-07	1.50E-03	4.26 (2.80-5.71)	9.47E-09	4.40E-05	3.03 (0.63-5.43)	1.34E-02	1.00E+00
ANLN	3	2.82 (1.65-3.98)	2.04E-06	9.16E-03	3.25 (2.10-4.41)	3.28E-08	1.52E-04	2.74 (0.95-4.53)	2.68E-03	1.00E+00
ANO9	2	5.41 (3.61-7.20)	3.37E-09	1.51E-05	5.91 (4.21-7.61)	9.34E-12	4.34E-08			
AOC2	2				5.92 (4.22-7.62)	9.10E-12	4.23E-08			
AOX1	2	4.56 (2.99-6.13)	1.38E-08	6.21E-05	4.90 (3.39-6.41)	1.93E-10	8.99E-07			
APBA3	2	4.90 (3.25-6.54)	5.19E-09	2.33E-05	5.51 (3.91-7.11)	1.63E-11	7.57E-08	3.03 (0.63-5.43)	1.34E-02	1.00E+00
APBB1IP	2				6.19 (4.40-7.99)	1.24E-11	5.77E-08			

ARHGEF17	5	1.68 (0.80-2.57)	1.99E-04	8.94E-01	2.20 (1.32-3.09)	1.02E-06	4.74E-03	2.34 (1.10-3.58)	2.23E-04	7.57E-01
ARHGEF4	2	2.89 (1.47-4.32)	6.95E-05	3.12E-01	3.23 (1.81-4.64)	7.64E-06	3.55E-02	1.08 (-0.49-2.66)	1.77E-01	1.00E+00
ARHGEF9	2	5.56 (3.60-7.53)	2.74E-08	1.23E-04	6.30 (4.34-8.26)	3.11E-10	1.44E-06			
ARID1A	3	2.43 (1.27-3.58)	3.69E-05	1.66E-01	2.64 (1.49-3.78)	6.30E-06	2.93E-02	1.49 (0.13-2.84)	3.12E-02	1.00E+00
ARID4A	3	2.49 (1.34-3.65)	2.34E-05	1.05E-01	3.06 (1.91-4.21)	1.82E-07	8.45E-04	1.24 (-0.07-2.55)	6.39E-02	1.00E+00
ARSH	5	1.58 (0.68-2.47)	5.59E-04	1.00E+00	2.10 (1.21-2.99)	4.18E-06	1.94E-02	1.57 (0.50-2.63)	4.06E-03	1.00E+00
ATG2A	2	3.62 (2.15-5.08)	1.30E-06	5.85E-03	4.36 (2.90-5.82)	4.93E-09	2.29E-05			
ATIC	2	5.12 (3.42-6.82)	3.60E-09	1.62E-05	5.11 (3.57-6.64)	7.14E-11	3.32E-07			
ATP13A5	2	6.51 (4.10-8.91)	1.11E-07	4.99E-04	5.92 (4.22-7.62)	9.11E-12	4.23E-08			
ATP2A1	7	4.32 (3.50-5.15)	0.00E+00	0.00E+00	3.99 (3.22-4.77)	0.00E+00	0.00E+00	4.29 (2.19-6.39)	6.06E-05	2.05E-01
ATP2C1	2	6.51 (4.11-8.92)	1.12E-07	5.01E-04	7.31 (4.90-9.71)	2.59E-09	1.20E-05	3.04 (0.63-5.44)	1.34E-02	1.00E+00
ATRN	2	4.90 (3.25-6.54)	5.21E-09	2.34E-05	4.74 (3.24-6.23)	4.92E-10	2.29E-06			
AXDND1	4	2.54 (1.54-3.54)	7.00E-07	3.14E-03	2.69 (1.70-3.69)	1.13E-07	5.27E-04	3.04 (1.34-4.74)	4.67E-04	1.00E+00
BARHL2	2	2.60 (1.18-4.02)	3.37E-04	1.00E+00	3.20 (1.78-4.61)	9.57E-06	4.45E-02	3.04 (0.63-5.44)	1.34E-02	1.00E+00
BAZ2B	2	5.81 (3.85-7.78)	6.44E-09	2.89E-05	3.87 (2.44-5.30)	1.22E-07	5.69E-04	3.03 (0.63-5.43)	1.34E-02	1.00E+00
BBOX1	5	1.45 (0.56-2.35)	1.43E-03	1.00E+00	2.02 (1.13-2.91)	9.12E-06	4.24E-02	1.66 (0.58-2.74)	2.67E-03	1.00E+00
BCAS3	2	5.41 (3.62-7.20)	3.34E-09	1.50E-05	6.20 (4.41-8.00)	1.15E-11	5.36E-08	3.03 (0.63-5.43)	1.34E-02	1.00E+00
BCL3	3	4.43 (3.16-5.70)	7.99E-12	3.59E-08	5.07 (3.82-6.32)	1.90E-15	9.00E-12	1.83 (0.39-3.26)	1.25E-02	1.00E+00
BCL7C	5	6.00 (4.68-7.33)	0.00E+00	2.60E-15	5.62 (4.57-6.66)	0.00E+00	0.00E+00			
BEST4	6	2.28 (1.47-3.10)	4.20E-08	1.89E-04	2.34 (1.54-3.15)	1.35E-08	6.28E-05	2.19 (1.09-3.28)	8.86E-05	3.00E-01
BFSP1	4	3.60 (2.56-4.64)	1.25E-11	5.61E-08	3.36 (2.36-4.37)	5.57E-11	2.59E-07			
BICD2	5	5.03 (3.97-6.09)	0.00E+00	1.00E-16	4.48 (3.55-5.41)	0.00E+00	0.00E+00	2.56 (1.25-3.88)	1.37E-04	4.64E-01
BIRC6	3	2.75 (1.59-3.91)	3.36E-06	1.51E-02	2.95 (1.80-4.10)	5.00E-07	2.33E-03	2.34 (0.74-3.94)	4.25E-03	1.00E+00
BMPR2	2	6.51 (4.10-8.91)	1.11E-07	4.99E-04	3.26 (1.85-4.67)	6.19E-06	2.88E-02			
BMX	3	2.14 (0.98-3.30)	2.99E-04	1.00E+00	2.70 (1.54-3.85)	4.83E-06	2.25E-02	1.84 (0.40-3.27)	1.24E-02	1.00E+00
BRD1	3	2.78 (1.62-3.94)	2.71E-06	1.22E-02	3.08 (1.93-4.23)	1.60E-07	7.43E-04	3.44 (1.17-5.70)	2.94E-03	1.00E+00
BRINP2	3	4.14 (2.90-5.37)	5.24E-11	2.35E-07	4.21 (3.03-5.40)	3.20E-12	1.49E-08	1.36 (0.03-2.69)	4.56E-02	1.00E+00
BTBD1	4	2.29 (1.28-3.29)	8.18E-06	3.67E-02	2.66 (1.66-3.66)	1.76E-07	8.17E-04	1.09 (-0.03-2.22)	5.58E-02	1.00E+00
BTBD7	2	3.47 (2.01-4.92)	3.13E-06	1.41E-02	3.91 (2.47-5.35)	1.02E-07	4.73E-04	1.65 (-0.06-3.35)	5.80E-02	1.00E+00
C10orf53	3	6.91 (4.65-9.18)	2.21E-09	9.93E-06	6.61 (5.01-8.21)	6.00E-16	2.91E-12	1.83 (0.39-3.26)	1.25E-02	1.00E+00
C10orf90	4	2.08 (1.08-3.08)	4.34E-05	1.95E-01	2.24 (1.25-3.23)	9.60E-06	4.46E-02	1.65 (0.44-2.85)	7.33E-03	1.00E+00

C6orf132	2	3.01 (1.58-4.44)	3.84E-05	1.72E-01	3.55 (2.13-4.98)	9.93E-07	4.62E-03	1.64 (-0.06-3.34)	5.81E-02	1.00E+00
CABIN1	3	2.34 (1.18-3.49)	7.07E-05	3.18E-01	2.63 (1.48-3.77)	6.97E-06	3.24E-02	3.44 (1.17-5.70)	2.94E-03	1.00E+00
CACNA1E	30	3.37 (2.99-3.75)	0.00E+00	0.00E+00	3.15 (2.77-3.52)	0.00E+00	0.00E+00	3.99 (3.11-4.88)	0.00E+00	2.10E-15
CACNA1S	2	4.11 (2.60-5.62)	9.74E-08	4.37E-04	4.53 (3.06-6.00)	1.69E-09	7.86E-06			
CAMK2A	2	4.90 (3.25-6.54)	5.44E-09	2.44E-05	5.00 (3.48-6.53)	1.28E-10	5.97E-07			
CARMIL3	2	5.41 (3.61-7.20)	3.34E-09	1.50E-05	3.31 (1.90-4.73)	4.44E-06	2.07E-02			
CARNMT1	2	5.81 (3.85-7.78)	6.43E-09	2.89E-05	6.61 (4.65-8.57)	4.07E-11	1.89E-07	0.73 (-0.80-2.25)	3.50E-01	1.00E+00
CARNS1	2				6.61 (4.65-8.57)	4.07E-11	1.89E-07			
CASKIN1	2	3.94 (2.45-5.43)	2.26E-07	1.02E-03	4.26 (2.80-5.71)	9.40E-09	4.37E-05			
CASZ1	2	5.41 (3.61-7.20)	3.38E-09	1.52E-05	3.51 (2.09-4.94)	1.24E-06	5.77E-03	3.03 (0.63-5.43)	1.34E-02	1.00E+00
CATSPER1	2	3.33 (1.88-4.77)	6.46E-06	2.90E-02	3.94 (2.50-5.37)	7.85E-08	3.65E-04	2.34 (0.37-4.30)	1.96E-02	1.00E+00
CCDC33	2	3.51 (2.05-4.98)	2.39E-06	1.08E-02	3.18 (1.77-4.60)	1.06E-05	4.93E-02	1.42 (-0.22-3.07)	8.99E-02	1.00E+00
CCDC73	3	3.44 (2.26-4.63)	1.26E-08	5.65E-05	3.84 (2.67-5.01)	1.27E-10	5.92E-07			
CCP110	2	6.51 (4.10-8.91)	1.11E-07	4.99E-04	7.30 (4.90-9.71)	2.56E-09	1.19E-05	3.03 (0.63-5.43)	1.34E-02	1.00E+00
CDC25B	15	3.02 (2.47-3.57)	0.00E+00	0.00E+00	2.65 (2.11-3.18)	0.00E+00	0.00E+00	2.72 (1.93-3.51)	1.83E-11	6.20E-08
CDH1	3	4.03 (2.80-5.26)	1.40E-10	6.29E-07	4.25 (3.06-5.44)	2.48E-12	1.15E-08			
CDH16	2	4.20 (2.68-5.72)	6.14E-08	2.76E-04	4.82 (3.32-6.32)	3.09E-10	1.44E-06			
CDK13	4	2.44 (1.45-3.44)	1.62E-06	7.29E-03	2.52 (1.53-3.51)	6.01E-07	2.80E-03	3.72 (1.53-5.92)	8.71E-04	1.00E+00
CEBPB	3	5.11 (3.72-6.50)	5.43E-13	2.44E-09	4.29 (3.11-5.48)	1.48E-12	6.89E-09	2.05 (0.55-3.55)	7.37E-03	1.00E+00
CEP131	2	5.39 (3.60-7.18)	3.69E-09	1.66E-05	4.80 (3.30-6.30)	3.53E-10	1.64E-06	3.03 (0.63-5.43)	1.34E-02	1.00E+00
CFAP251	10	1.07 (0.44-1.70)	8.73E-04	1.00E+00	1.61 (0.98-2.24)	5.79E-07	2.69E-03	1.32 (0.59-2.05)	3.82E-04	1.00E+00
CFAP45	3	5.30 (3.87-6.74)	4.22E-13	1.89E-09	5.92 (4.53-7.31)	1.00E-16	3.09E-13			
CFAP46	13	1.18 (0.63-1.73)	2.57E-05	1.16E-01	1.59 (1.04-2.14)	1.23E-08	5.70E-05	1.41 (0.76-2.05)	1.83E-05	6.20E-02
CFAP54	3	2.87 (1.70-4.03)	1.37E-06	6.14E-03	3.43 (2.27-4.59)	6.28E-09	2.92E-05	2.34 (0.74-3.94)	4.25E-03	1.00E+00
CFAP57	3	2.40 (1.25-3.56)	4.52E-05	2.03E-01	2.95 (1.80-4.10)	5.07E-07	2.36E-03	1.36 (0.03-2.69)	4.55E-02	1.00E+00
CHKA	4	2.52 (1.51-3.52)	8.69E-07	3.90E-03	2.50 (1.51-3.50)	7.87E-07	3.66E-03	1.65 (0.44-2.85)	7.33E-03	1.00E+00
CHPT1	2	6.50 (4.10-8.91)	1.12E-07	5.02E-04	6.61 (4.65-8.57)	4.13E-11	1.92E-07			
CHRNA3	2				6.60 (4.63-8.56)	4.47E-11	2.08E-07			
CILP2	4	2.09 (1.09-3.09)	4.40E-05	1.97E-01	2.44 (1.44-3.44)	1.67E-06	7.75E-03	2.13 (0.81-3.45)	1.62E-03	1.00E+00
CLSTN2	2	4.90 (3.25-6.54)	5.17E-09	2.32E-05	4.66 (3.18-6.15)	7.46E-10	3.47E-06			
CMIP	2				7.30 (4.90-9.70)	2.57E-09	1.19E-05			

CNGB1	10	3.25 (2.60-3.90)	0.00E+00	0.00E+00	3.16 (2.53-3.80)	0.00E+00	0.00E+00	2.45 (1.55-3.36)	1.05E-07	3.57E-04
COG5	2				5.92 (4.21-7.62)	9.20E-12	4.28E-08			
COL4A4	2	6.51 (4.10-8.91)	1.11E-07	5.00E-04	6.20 (4.41-8.00)	1.16E-11	5.41E-08			
COL5A3	2	3.62 (2.15-5.08)	1.31E-06	5.87E-03	4.12 (2.68-5.57)	2.27E-08	1.05E-04			
COL6A3	10	1.61 (0.98-2.24)	4.75E-07	2.13E-03	1.68 (1.06-2.31)	1.31E-07	6.08E-04	1.51 (0.76-2.25)	7.36E-05	2.49E-01
COL6A6	3	4.27 (3.02-5.52)	2.07E-11	9.31E-08	4.57 (3.37-5.78)	1.07E-13	4.96E-10	3.44 (1.17-5.70)	2.94E-03	1.00E+00
COPG1	2	6.51 (4.10-8.91)	1.11E-07	4.99E-04	5.36 (3.78-6.93)	2.60E-11	1.21E-07	2.34 (0.37-4.30)	1.96E-02	1.00E+00
CORO1A	6	1.71 (0.89-2.52)	3.87E-05	1.74E-01	2.05 (1.24-2.86)	6.84E-07	3.18E-03	1.09 (0.18-2.00)	1.93E-02	1.00E+00
CRACR2A	2	4.71 (3.11-6.32)	8.33E-09	3.74E-05	5.36 (3.78-6.93)	2.61E-11	1.21E-07			
CRB2	3	4.83 (3.50-6.16)	1.06E-12	4.74E-09	3.99 (2.82-5.17)	2.70E-11	1.25E-07	3.44 (1.17-5.70)	2.94E-03	1.00E+00
CSF1	2	5.81 (3.85-7.78)	6.44E-09	2.89E-05	6.61 (4.65-8.57)	4.09E-11	1.90E-07			
CSMD1	5	3.40 (2.48-4.31)	3.92E-13	1.76E-09	3.86 (2.95-4.77)	1.00E-16	3.25E-13	2.00 (0.85-3.15)	6.45E-04	1.00E+00
CSMD2	5	3.10 (2.19-4.02)	2.36E-11	1.06E-07	3.19 (2.29-4.08)	3.12E-12	1.45E-08			
CSMD3	2	4.29 (2.75-5.82)	4.46E-08	2.00E-04	4.71 (3.22-6.21)	5.89E-10	2.74E-06			
CTNNBL1	2	2.46 (1.05-3.88)	6.39E-04	1.00E+00	3.19 (1.78-4.60)	9.40E-06	4.37E-02	1.93 (0.14-3.72)	3.46E-02	1.00E+00
CWC25	3	3.33 (2.15-4.51)	3.36E-08	1.51E-04	2.75 (1.61-3.90)	2.57E-06	1.19E-02	3.44 (1.17-5.70)	2.93E-03	1.00E+00
CYLD	8	4.00 (3.24-4.76)	0.00E+00	0.00E+00	3.60 (2.88-4.33)	0.00E+00	0.00E+00	3.76 (2.20-5.32)	2.20E-06	7.44E-03
CYP2J2	3	2.94 (1.77-4.11)	7.69E-07	3.45E-03	2.87 (1.72-4.02)	9.42E-07	4.38E-03	2.74 (0.95-4.53)	2.68E-03	1.00E+00
DACT2	11	2.79 (2.17-3.40)	0.00E+00	2.10E-15	2.66 (2.06-3.27)	0.00E+00	2.24E-14	1.57 (0.85-2.29)	1.91E-05	6.46E-02
DCBLD1	2	4.11 (2.60-5.62)	9.69E-08	4.35E-04	4.47 (3.00-5.94)	2.44E-09	1.13E-05	2.34 (0.37-4.30)	1.96E-02	1.00E+00
DCDC1	9	1.42 (0.76-2.08)	2.54E-05	1.14E-01	1.60 (0.94-2.26)	1.93E-06	8.98E-03	2.34 (1.42-3.27)	7.21E-07	2.44E-03
DCHS1	3	2.54 (1.39-3.70)	1.63E-05	7.33E-02	2.70 (1.56-3.85)	3.73E-06	1.73E-02	1.13 (-0.16-2.43)	8.60E-02	1.00E+00
DCTN1	3	4.27 (3.02-5.52)	2.07E-11	9.29E-08	4.27 (3.09-5.46)	1.75E-12	8.15E-09	2.34 (0.74-3.94)	4.25E-03	1.00E+00
DDIAS	3	2.32 (1.17-3.47)	8.12E-05	3.64E-01	2.91 (1.76-4.06)	7.24E-07	3.37E-03	2.75 (0.95-4.54)	2.68E-03	1.00E+00
DDX31	2	5.81 (3.85-7.78)	6.43E-09	2.89E-05	6.61 (4.65-8.57)	4.08E-11	1.89E-07			
DENND1A	3	6.22 (4.43-8.01)	1.03E-11	4.61E-08	6.61 (5.01-8.21)	6.00E-16	2.96E-12	2.05 (0.55-3.55)	7.37E-03	1.00E+00
DESI1	2	3.87 (2.38-5.36)	3.43E-07	1.54E-03	4.42 (2.95-5.88)	3.68E-09	1.71E-05	3.04 (0.63-5.44)	1.34E-02	1.00E+00
DGKG	4	4.10 (3.03-5.17)	5.59E-14	2.51E-10	3.50 (2.49-4.51)	9.38E-12	4.36E-08			
DHRS7C	3	2.83 (1.66-3.99)	2.11E-06	9.48E-03	3.16 (2.01-4.32)	8.53E-08	3.96E-04	1.25 (-0.07-2.56)	6.34E-02	1.00E+00
DHX37	23	3.36 (2.90-3.82)	0.00E+00	0.00E+00	3.03 (2.59-3.47)	0.00E+00	0.00E+00	3.10 (2.39-3.82)	0.00E+00	6.12E-14
DHX38	7	2.06 (1.30-2.81)	1.04E-07	4.69E-04	2.14 (1.39-2.90)	2.36E-08	1.10E-04	1.65 (0.74-2.57)	3.84E-04	1.00E+00

DIAPH3	3	2.18 (1.03-3.34)	2.11E-04	9.47E-01	2.75 (1.60-3.91)	2.79E-06	1.30E-02	1.25 (-0.07-2.56)	6.34E-02	1.00E+00
DIDO1	2	3.67 (2.20-5.14)	9.51E-07	4.27E-03	4.21 (2.76-5.66)	1.27E-08	5.91E-05			
DIPK1C	2	2.63 (1.22-4.05)	2.72E-04	1.00E+00	3.28 (1.86-4.69)	5.56E-06	2.59E-02	3.03 (0.63-5.43)	1.34E-02	1.00E+00
DNAH14	12	1.04 (0.47-1.61)	3.31E-04	1.00E+00	1.48 (0.91-2.05)	3.37E-07	1.57E-03	1.02 (0.38-1.66)	1.82E-03	1.00E+00
DNAH17	9	2.20 (1.53-2.86)	9.12E-11	4.10E-07	2.18 (1.52-2.84)	8.83E-11	4.11E-07	1.49 (0.71-2.27)	1.89E-04	6.41E-01
DNAH7	7	1.65 (0.90-2.40)	1.67E-05	7.50E-02	1.68 (0.93-2.43)	1.06E-05	4.93E-02	1.34 (0.47-2.21)	2.51E-03	1.00E+00
DNAJB8	5	7.39 (5.23-9.54)	1.72E-11	7.73E-08	6.56 (5.32-7.81)	0.00E+00	0.00E+00			
DOCK5	2	5.12 (3.42-6.83)	3.71E-09	1.66E-05	5.23 (3.67-6.78)	4.55E-11	2.12E-07	2.34 (0.38-4.31)	1.95E-02	1.00E+00
DOK7	6	1.74 (0.93-2.55)	2.74E-05	1.23E-01	1.88 (1.07-2.69)	5.25E-06	2.44E-02	2.19 (1.10-3.29)	8.74E-05	2.96E-01
DPT	4	2.26 (1.25-3.26)	1.05E-05	4.73E-02	2.70 (1.70-3.70)	1.18E-07	5.50E-04	1.54 (0.35-2.73)	1.11E-02	1.00E+00
DST	16	2.63 (2.12-3.13)	0.00E+00	0.00E+00	1.80 (1.31-2.30)	9.69E-13	4.51E-09	2.34 (1.65-3.04)	3.91E-11	1.32E-07
DYNLT2B	11	2.95 (2.33-3.57)	0.00E+00	1.00E-16	2.42 (1.82-3.03)	4.30E-15	2.01E-11	1.50 (0.78-2.21)	3.94E-05	1.34E-01
DZIP3	2	4.11 (2.60-5.62)	9.76E-08	4.38E-04	4.66 (3.18-6.15)	7.61E-10	3.54E-06	3.03 (0.63-5.43)	1.34E-02	1.00E+00
EFCAB5	5	1.96 (1.07-2.85)	1.56E-05	7.02E-02	2.44 (1.56-3.33)	6.65E-08	3.09E-04	0.95 (-0.03-1.94)	5.78E-02	1.00E+00
EHD2	3	2.91 (1.74-4.07)	1.04E-06	4.67E-03	3.52 (2.36-4.68)	2.80E-09	1.30E-05	2.75 (0.95-4.54)	2.68E-03	1.00E+00
EML6	3	2.14 (0.99-3.29)	2.61E-04	1.00E+00	2.75 (1.61-3.90)	2.55E-06	1.18E-02	1.49 (0.13-2.84)	3.12E-02	1.00E+00
ENPP6	2	3.51 (2.05-4.97)	2.34E-06	1.05E-02	4.31 (2.85-5.76)	6.86E-09	3.19E-05			
EPHA7	2	5.12 (3.42-6.82)	3.62E-09	1.63E-05	5.51 (3.91-7.11)	1.64E-11	7.61E-08			
EPHX3	2	5.32 (3.53-7.11)	5.83E-09	2.62E-05	5.27 (3.69-6.84)	5.44E-11	2.53E-07			
ESF1	3	4.08 (2.85-5.31)	8.17E-11	3.67E-07	3.50 (2.34-4.66)	3.19E-09	1.48E-05	2.74 (0.95-4.53)	2.68E-03	1.00E+00
ETV5	2	2.90 (1.47-4.33)	7.12E-05	3.20E-01	3.36 (1.93-4.78)	3.76E-06	1.75E-02	3.04 (0.63-5.44)	1.34E-02	1.00E+00
EVPL	34	5.16 (4.75-5.58)	0.00E+00	0.00E+00	4.55 (4.18-4.91)	0.00E+00	0.00E+00	4.51 (3.47-5.55)	0.00E+00	5.83E-14
EXD3	2	6.51 (4.10-8.91)	1.11E-07	5.00E-04	7.30 (4.90-9.71)	2.56E-09	1.19E-05	3.03 (0.63-5.43)	1.34E-02	1.00E+00
EXOC3L4	3	2.94 (1.77-4.11)	7.68E-07	3.45E-03	3.52 (2.36-4.68)	2.74E-09	1.27E-05	1.64 (0.26-3.03)	2.03E-02	1.00E+00
EXTL3	2	6.51 (4.11-8.92)	1.11E-07	5.00E-04	6.62 (4.65-8.58)	4.17E-11	1.94E-07			
F13B	2	5.41 (3.61-7.20)	3.37E-09	1.51E-05	6.20 (4.41-7.99)	1.17E-11	5.46E-08			
F7	2	5.41 (3.62-7.20)	3.33E-09	1.50E-05	5.51 (3.91-7.11)	1.62E-11	7.51E-08	3.03 (0.63-5.43)	1.34E-02	1.00E+00
FADS6	2	4.86 (3.22-6.50)	6.72E-09	3.02E-05	5.31 (3.74-6.89)	3.71E-11	1.72E-07	1.42 (-0.22-3.06)	9.02E-02	1.00E+00
FAM135A	2	3.01 (1.58-4.45)	3.81E-05	1.71E-01	3.38 (1.96-4.80)	3.16E-06	1.47E-02	1.42 (-0.22-3.07)	8.99E-02	1.00E+00
FAM170A	2	3.10 (1.67-4.54)	2.23E-05	1.00E-01	3.72 (2.29-5.15)	3.26E-07	1.52E-03			
FAM186A	4	5.25 (4.02-6.48)	1.00E-16	2.91E-13	4.90 (3.83-5.97)	0.00E+00	1.20E-15	2.63 (1.13-4.13)	5.94E-04	1.00E+00

FAM89A	5	2.37 (1.48-3.27)	2.10E-07	9.45E-04	2.59 (1.70-3.48)	1.15E-08	5.37E-05	3.96 (1.81-6.10)	3.11E-04	1.00E+00
FBLN2	2				7.30 (4.90-9.71)	2.56E-09	1.19E-05			
FBXO7	2				6.20 (4.41-8.00)	1.16E-11	5.37E-08			
FOXK2	7	1.48 (0.72-2.23)	1.17E-04	5.24E-01	1.79 (1.05-2.54)	2.61E-06	1.21E-02	1.52 (0.62-2.41)	8.70E-04	1.00E+00
FOXP2	2	2.94 (1.51-4.37)	5.52E-05	2.48E-01	3.41 (2.00-4.83)	2.36E-06	1.10E-02	2.34 (0.37-4.30)	1.96E-02	1.00E+00
FRY	8	3.56 (2.82-4.31)	0.00E+00	0.00E+00	3.31 (2.59-4.03)	0.00E+00	9.00E-16	3.76 (2.20-5.32)	2.20E-06	7.44E-03
FSTL5	2	3.01 (1.58-4.44)	3.76E-05	1.69E-01	3.56 (2.14-4.99)	8.97E-07	4.17E-03	1.93 (0.14-3.72)	3.46E-02	1.00E+00
GBX1	2	2.92 (1.49-4.35)	6.02E-05	2.70E-01	3.31 (1.90-4.73)	4.43E-06	2.06E-02	1.42 (-0.22-3.06)	9.02E-02	1.00E+00
GDF11	5				5.93 (4.85-7.01)	0.00E+00	0.00E+00			
GJB6	6	1.45 (0.63-2.27)	5.02E-04	1.00E+00	1.97 (1.15-2.79)	2.22E-06	1.03E-02	1.26 (0.32-2.19)	8.49E-03	1.00E+00
GPAT3	2	5.78 (3.82-7.74)	7.90E-09	3.55E-05	5.88 (4.18-7.58)	1.23E-11	5.71E-08			
GPC1	2	5.41 (3.62-7.20)	3.34E-09	1.50E-05	4.12 (2.68-5.57)	2.26E-08	1.05E-04	3.03 (0.63-5.43)	1.34E-02	1.00E+00
GPR83	3	2.23 (1.07-3.38)	1.57E-04	7.05E-01	2.82 (1.67-3.97)	1.63E-06	7.59E-03	1.36 (0.03-2.70)	4.53E-02	1.00E+00
GRB7	3	3.00 (1.83-4.17)	4.83E-07	2.17E-03	3.58 (2.42-4.74)	1.51E-09	7.04E-06	2.74 (0.95-4.53)	2.68E-03	1.00E+00
GRK1	2	3.46 (2.01-4.91)	3.07E-06	1.38E-02	3.64 (2.21-5.06)	5.51E-07	2.56E-03	1.64 (-0.06-3.34)	5.81E-02	1.00E+00
H1-10	12	2.41 (1.83-3.00)	6.00E-16	2.64E-12	2.31 (1.74-2.89)	4.70E-15	2.19E-11	1.80 (1.08-2.52)	8.72E-07	2.95E-03
H6PD	2	4.90 (3.25-6.54)	5.17E-09	2.32E-05	4.82 (3.32-6.32)	3.08E-10	1.43E-06	3.03 (0.63-5.43)	1.34E-02	1.00E+00
HDAC4	2	4.31 (2.77-5.84)	3.81E-08	1.71E-04	4.74 (3.25-6.23)	4.85E-10	2.26E-06	3.03 (0.63-5.43)	1.34E-02	1.00E+00
HEATR5B	2	4.43 (2.87-5.98)	2.30E-08	1.03E-04	4.53 (3.06-6.00)	1.68E-09	7.79E-06	1.64 (-0.06-3.34)	5.81E-02	1.00E+00
HECA	4	2.27 (1.27-3.27)	8.30E-06	3.73E-02	2.76 (1.77-3.76)	5.11E-08	2.38E-04	1.33 (0.18-2.47)	2.35E-02	1.00E+00
HELZ2	5	3.84 (2.90-4.78)	1.00E-15	4.58E-12	3.13 (2.23-4.02)	6.56E-12	3.05E-08	2.56 (1.24-3.88)	1.37E-04	4.65E-01
HLA-DQA1	4	3.49 (2.42-4.55)	1.35E-10	6.04E-07	3.97 (2.93-5.02)	9.85E-14	4.58E-10	2.13 (0.81-3.45)	1.62E-03	1.00E+00
HMCN2	12	1.53 (0.96-2.11)	1.41E-07	6.35E-04	1.67 (1.10-2.24)	8.56E-09	3.98E-05	1.60 (0.91-2.29)	5.20E-06	1.76E-02
HOXA7	2	2.96 (1.52-4.39)	5.24E-05	2.35E-01	3.64 (2.22-5.07)	5.68E-07	2.64E-03	3.04 (0.63-5.44)	1.34E-02	1.00E+00
HSD17B2	2	3.01 (1.58-4.44)	3.75E-05	1.68E-01	3.50 (2.08-4.92)	1.39E-06	6.47E-03			
HSPG2	5	3.40 (2.48-4.31)	3.87E-13	1.74E-09	3.93 (3.02-4.84)	0.00E+00	1.06E-13	3.25 (1.61-4.90)	1.02E-04	3.45E-01
HTATSF1	2	5.57 (3.60-7.53)	2.70E-08	1.21E-04	5.90 (4.11-7.70)	1.07E-10	4.96E-07	1.93 (0.14-3.72)	3.46E-02	1.00E+00
HTR1F	2	2.70 (1.28-4.12)	1.95E-04	8.74E-01	3.24 (1.83-4.66)	6.89E-06	3.20E-02	2.34 (0.37-4.30)	1.96E-02	1.00E+00
HTR3E	2	3.51 (2.05-4.97)	2.34E-06	1.05E-02	4.17 (2.72-5.62)	1.71E-08	7.95E-05	1.24 (-0.37-2.84)	1.30E-01	1.00E+00
IDUA	6	1.73 (0.91-2.54)	3.18E-05	1.43E-01	2.32 (1.51-3.13)	2.16E-08	1.00E-04	1.50 (0.54-2.46)	2.28E-03	1.00E+00

IL12RB1	7	1.28 (0.53-2.03)	8.20E-04	1.00E+00	1.72 (0.97-2.46)	6.96E-06	3.24E-02	1.80 (0.87-2.74)	1.58E-04	5.37E-01
INSR	2	4.90 (3.25-6.54)	5.20E-09	2.34E-05	4.90 (3.39-6.41)	1.96E-10	9.09E-07			
ITGAD	2	2.98 (1.55-4.41)	4.44E-05	2.00E-01	3.59 (2.16-5.01)	7.75E-07	3.60E-03			
ITGAE	2	6.51 (4.10-8.91)	1.11E-07	4.99E-04	5.92 (4.22-7.62)	9.09E-12	4.23E-08			
ITPRID2	2	4.71 (3.11-6.32)	8.29E-09	3.72E-05	5.22 (3.67-6.78)	4.28E-11	1.99E-07			
ITSN1	4	2.53 (1.52-3.54)	8.51E-07	3.82E-03	2.32 (1.32-3.31)	5.23E-06	2.43E-02	1.95 (0.67-3.22)	2.76E-03	1.00E+00
IZUMO3	2	3.17 (1.74-4.61)	1.53E-05	6.86E-02	3.87 (2.43-5.30)	1.23E-07	5.72E-04	0.73 (-0.80-2.25)	3.50E-01	1.00E+00
JPH2	8	3.14 (2.41-3.87)	0.00E+00	1.40E-13	3.07 (2.36-3.78)	0.00E+00	1.39E-13	1.09 (0.30-1.89)	6.83E-03	1.00E+00
KCNA5	2	6.51 (4.10-8.91)	1.11E-07	5.00E-04	5.69 (4.05-7.34)	1.10E-11	5.12E-08			
KCNG4	2	4.20 (2.68-5.72)	6.14E-08	2.76E-04	3.57 (2.14-4.99)	8.92E-07	4.15E-03			
KDM4B	3	5.12 (3.73-6.51)	4.95E-13	2.22E-09	5.31 (4.03-6.59)	4.00E-16	1.90E-12			
KDM5A	2	4.11 (2.60-5.62)	9.71E-08	4.36E-04	3.37 (1.95-4.79)	3.10E-06	1.44E-02			
KIAA1109	2	3.66 (2.19-5.13)	1.03E-06	4.62E-03	4.46 (2.99-5.93)	2.70E-09	1.25E-05			
KIDINS220	2	5.41 (3.62-7.20)	3.34E-09	1.50E-05	5.92 (4.22-7.62)	9.12E-12	4.24E-08			
KIF21B	2	4.31 (2.77-5.84)	3.80E-08	1.71E-04	4.31 (2.85-5.76)	6.89E-09	3.21E-05			
KIF26B	5	3.19 (2.28-4.10)	6.58E-12	2.95E-08	3.61 (2.71-4.51)	3.60E-15	1.69E-11	3.95 (1.80-6.10)	3.16E-04	1.00E+00
KLHDC2	2				6.61 (4.65-8.57)	4.09E-11	1.90E-07			
KLHL1	2	2.96 (1.52-4.39)	5.23E-05	2.35E-01	3.20 (1.78-4.61)	9.55E-06	4.44E-02			
KLHL34	3	5.02 (3.59-6.45)	6.76E-12	3.04E-08	4.00 (2.81-5.19)	4.87E-11	2.27E-07			
KMT2B	5	2.95 (2.04-3.85)	1.69E-10	7.58E-07	3.26 (2.36-4.15)	9.53E-13	4.43E-09	3.95 (1.80-6.10)	3.15E-04	1.00E+00
KMT2C	7	2.08 (1.31-2.84)	9.76E-08	4.38E-04	2.49 (1.73-3.25)	1.41E-10	6.53E-07	2.01 (1.03-2.99)	5.66E-05	1.92E-01
KRI1	3	4.97 (3.61-6.32)	6.87E-13	3.08E-09	5.22 (3.95-6.49)	7.00E-16	3.16E-12	2.74 (0.95-4.53)	2.68E-03	1.00E+00
LAMA2	4	2.60 (1.60-3.61)	3.59E-07	1.61E-03	3.14 (2.15-4.14)	6.75E-10	3.14E-06	2.12 (0.80-3.43)	1.65E-03	1.00E+00
LAMB2	2				5.92 (4.22-7.62)	9.09E-12	4.23E-08			
LAMC3	2	4.02 (2.52-5.52)	1.50E-07	6.72E-04	4.31 (2.85-5.76)	6.85E-09	3.19E-05	1.24 (-0.37-2.84)	1.30E-01	1.00E+00
LIPG	2	3.38 (1.92-4.83)	5.21E-06	2.34E-02	3.94 (2.50-5.38)	8.17E-08	3.80E-04	1.94 (0.14-3.73)	3.45E-02	1.00E+00
LRBA	7	2.01 (1.26-2.77)	1.60E-07	7.20E-04	2.56 (1.81-3.31)	2.36E-11	1.10E-07	1.89 (0.94-2.84)	9.78E-05	3.31E-01
LRIF1	2	3.14 (1.70-4.58)	1.88E-05	8.45E-02	3.64 (2.22-5.07)	5.73E-07	2.66E-03	3.04 (0.63-5.44)	1.34E-02	1.00E+00
LRP4	4	2.66 (1.65-3.67)	2.17E-07	9.72E-04	3.21 (2.21-4.21)	3.17E-10	1.47E-06	2.12 (0.80-3.44)	1.64E-03	1.00E+00
LRRC53	9	1.07 (0.41-1.72)	1.53E-03	1.00E+00	1.50 (0.84-2.16)	7.86E-06	3.66E-02	1.07 (0.33-1.81)	4.71E-03	1.00E+00
LRRC74A	2	3.73 (2.26-5.21)	6.84E-07	3.07E-03	4.36 (2.90-5.82)	4.96E-09	2.30E-05			

LRRFIP2	2	3.80 (2.32-5.29)	4.94E-07	2.22E-03	3.62 (2.19-5.05)	6.69E-07	3.11E-03	1.65 (-0.06-3.35)	5.80E-02	1.00E+00
LRWD1	2	4.72 (3.11-6.33)	8.55E-09	3.84E-05	5.23 (3.67-6.78)	4.50E-11	2.09E-07	1.09 (-0.49-2.67)	1.77E-01	1.00E+00
LTBP4	2	4.90 (3.25-6.54)	5.19E-09	2.33E-05	5.36 (3.78-6.93)	2.60E-11	1.21E-07	1.24 (-0.37-2.84)	1.30E-01	1.00E+00
LYSMD4	2	2.93 (1.50-4.36)	6.08E-05	2.73E-01	3.28 (1.87-4.70)	5.65E-06	2.63E-02			
MACIR	3	3.18 (2.00-4.35)	1.20E-07	5.38E-04	3.63 (2.47-4.80)	9.77E-10	4.54E-06	1.13 (-0.16-2.43)	8.59E-02	1.00E+00
MAD1L1	3	5.82 (4.21-7.42)	1.17E-12	5.26E-09	6.33 (4.82-7.83)	1.00E-16	6.59E-13	2.34 (0.74-3.94)	4.24E-03	1.00E+00
MADD	4	1.81 (0.82-2.80)	3.61E-04	1.00E+00	2.38 (1.39-3.38)	2.49E-06	1.16E-02	1.65 (0.44-2.85)	7.33E-03	1.00E+00
MAP1S	2	6.51 (4.10-8.91)	1.11E-07	5.00E-04	6.61 (4.65-8.57)	4.07E-11	1.89E-07	3.03 (0.63-5.43)	1.34E-02	1.00E+00
MAS1L	9	2.71 (2.04-3.38)	3.10E-15	1.41E-11	2.94 (2.28-3.61)	0.00E+00	2.46E-14	1.77 (0.95-2.59)	2.38E-05	8.05E-02
MAST2	3	5.30 (3.87-6.74)	4.23E-13	1.90E-09	4.62 (3.41-5.83)	7.17E-14	3.33E-10			
MAST4	2	5.41 (3.62-7.20)	3.33E-09	1.50E-05	4.82 (3.32-6.32)	3.08E-10	1.43E-06	2.34 (0.37-4.30)	1.96E-02	1.00E+00
MCCC1	2	4.71 (3.11-6.32)	8.29E-09	3.72E-05	3.69 (2.27-5.12)	3.91E-07	1.82E-03	2.34 (0.37-4.30)	1.96E-02	1.00E+00
MCHR2	2	5.81 (3.85-7.78)	6.44E-09	2.89E-05	6.61 (4.65-8.57)	4.08E-11	1.90E-07	3.03 (0.63-5.43)	1.34E-02	1.00E+00
MCUR1	2				7.30 (4.90-9.70)	2.58E-09	1.20E-05			
MED23	2	3.29 (1.84-4.73)	8.12E-06	3.65E-02	3.94 (2.50-5.37)	7.89E-08	3.67E-04			
MERTK	9	3.00 (2.32-3.68)	0.00E+00	2.73E-14	2.93 (2.26-3.60)	0.00E+00	5.19E-14			
MFHAS1	4	1.95 (0.96-2.95)	1.22E-04	5.49E-01	2.34 (1.35-3.33)	3.73E-06	1.73E-02	1.65 (0.44-2.85)	7.33E-03	1.00E+00
MGAM	4	3.96 (2.90-5.01)	2.39E-13	1.07E-09	4.58 (3.53-5.62)	0.00E+00	4.78E-14			
MICAL3	3	2.85 (1.69-4.01)	1.57E-06	7.06E-03	2.67 (1.53-3.82)	4.89E-06	2.27E-02	1.83 (0.39-3.26)	1.25E-02	1.00E+00
MMP25	3	2.69 (1.53-3.85)	5.38E-06	2.42E-02	3.18 (2.02-4.33)	6.78E-08	3.15E-04	1.36 (0.03-2.69)	4.55E-02	1.00E+00
MMS22L	2	3.87 (2.38-5.36)	3.43E-07	1.54E-03	3.87 (2.44-5.31)	1.28E-07	5.93E-04	0.95 (-0.60-2.51)	2.30E-01	1.00E+00
MORC2	4	3.35 (2.32-4.38)	1.87E-10	8.39E-07	3.29 (2.28-4.30)	1.60E-10	7.43E-07	1.79 (0.55-3.03)	4.56E-03	1.00E+00
MRGPRF	2	6.51 (4.11-8.92)	1.11E-07	5.00E-04	7.31 (4.90-9.71)	2.57E-09	1.20E-05			
MRM3	2	3.07 (1.64-4.51)	2.67E-05	1.20E-01	3.75 (2.32-5.18)	2.72E-07	1.26E-03			
MROH1	4	3.33 (2.30-4.35)	1.84E-10	8.25E-07	3.05 (2.06-4.05)	1.94E-09	9.03E-06			
MROH2B	3	3.13 (1.96-4.30)	1.74E-07	7.82E-04	3.08 (1.93-4.24)	1.55E-07	7.21E-04	1.49 (0.14-2.85)	3.11E-02	1.00E+00
MTUS2	7	2.66 (1.90-3.43)	7.73E-12	3.47E-08	3.27 (2.51-4.03)	0.00E+00	1.66E-13	1.99 (1.02-2.96)	5.85E-05	1.98E-01
MUC16	23	1.43 (1.01-1.84)	1.27E-11	5.72E-08	1.38 (0.97-1.79)	5.26E-11	2.45E-07	1.41 (0.93-1.90)	1.06E-08	3.58E-05
MUC4	28	2.20 (1.82-2.58)	0.00E+00	0.00E+00	2.35 (1.98-2.73)	0.00E+00	0.00E+00	1.21 (0.78-1.64)	3.18E-08	1.08E-04
MUC5AC	11	2.83 (2.22-3.44)	0.00E+00	5.00E-16	2.90 (2.30-3.51)	0.00E+00	0.00E+00	3.36 (2.21-4.51)	9.47E-09	3.21E-05
MUC5B	3	3.12 (1.95-4.29)	1.80E-07	8.09E-04	3.64 (2.48-4.80)	8.51E-10	3.96E-06	2.74 (0.95-4.53)	2.68E-03	1.00E+00

MUC6	6	1.53 (0.72-2.34)	2.14E-04	9.61E-01	1.85 (1.04-2.66)	6.99E-06	3.25E-02	1.30 (0.36-2.23)	6.44E-03	1.00E+00
MX1	2				5.90 (4.20-7.60)	1.02E-11	4.72E-08			
MYBBP1A	6	1.47 (0.66-2.28)	3.70E-04	1.00E+00	1.95 (1.15-2.76)	2.10E-06	9.78E-03	2.05 (0.99-3.11)	1.50E-04	5.09E-01
MYH14	3	3.41 (2.23-4.60)	1.63E-08	7.32E-05	3.88 (2.71-5.05)	8.48E-11	3.94E-07	3.44 (1.17-5.70)	2.94E-03	1.00E+00
MYH4	4	2.24 (1.23-3.24)	1.26E-05	5.67E-02	2.85 (1.84-3.85)	2.54E-08	1.18E-04	1.79 (0.55-3.03)	4.56E-03	1.00E+00
MYH7B	7	1.49 (0.74-2.24)	1.03E-04	4.62E-01	1.94 (1.20-2.69)	3.61E-07	1.68E-03	1.29 (0.43-2.16)	3.42E-03	1.00E+00
MYO16	3	2.05 (0.90-3.20)	4.61E-04	1.00E+00	2.58 (1.43-3.72)	1.01E-05	4.71E-02	1.83 (0.39-3.26)	1.25E-02	1.00E+00
MYO18A	3	2.68 (1.52-3.84)	6.00E-06	2.70E-02	3.04 (1.89-4.19)	2.34E-07	1.09E-03	2.34 (0.74-3.94)	4.24E-03	1.00E+00
MYO18B	3	6.91 (4.65-9.18)	2.21E-09	9.93E-06	2.90 (1.75-4.04)	7.69E-07	3.58E-03			
MYO19	2	5.81 (3.85-7.78)	6.43E-09	2.89E-05	5.92 (4.22-7.62)	9.11E-12	4.24E-08			
MYO1C	2	6.51 (4.10-8.91)	1.11E-07	5.00E-04	5.51 (3.91-7.11)	1.64E-11	7.60E-08			
MYOM3	2	5.41 (3.62-7.20)	3.34E-09	1.50E-05	5.92 (4.22-7.62)	9.10E-12	4.23E-08	2.34 (0.37-4.30)	1.96E-02	1.00E+00
NAAA	2	4.56 (2.99-6.13)	1.38E-08	6.19E-05	5.11 (3.57-6.64)	7.13E-11	3.32E-07	3.03 (0.63-5.43)	1.34E-02	1.00E+00
NAGA	3	3.97 (2.75-5.19)	1.92E-10	8.62E-07	4.67 (3.45-5.88)	4.91E-14	2.28E-10	3.44 (1.17-5.70)	2.93E-03	1.00E+00
NAP1L3	11	3.31 (2.68-3.95)	0.00E+00	0.00E+00	3.18 (2.56-3.80)	0.00E+00	0.00E+00	3.66 (2.38-4.94)	2.14E-08	7.24E-05
NBEAL2	3	2.14 (0.99-3.29)	2.61E-04	1.00E+00	2.59 (1.45-3.73)	9.21E-06	4.28E-02	0.66 (-0.57-1.90)	2.94E-01	1.00E+00
NCOA6	2	4.90 (3.25-6.54)	5.17E-09	2.32E-05	5.11 (3.57-6.64)	7.15E-11	3.33E-07			
NDST1	2	4.03 (2.52-5.53)	1.54E-07	6.91E-04	4.54 (3.06-6.01)	1.75E-09	8.12E-06	3.04 (0.63-5.44)	1.34E-02	1.00E+00
NEB	8	2.33 (1.63-3.04)	8.88E-11	3.99E-07	2.54 (1.84-3.24)	1.18E-12	5.48E-09	2.02 (1.11-2.93)	1.43E-05	4.86E-02
NFASC	2	5.12 (3.42-6.82)	3.61E-09	1.62E-05	4.59 (3.12-6.07)	1.13E-09	5.27E-06	2.34 (0.37-4.30)	1.96E-02	1.00E+00
NFATC4	2	5.41 (3.62-7.20)	3.34E-09	1.50E-05	3.47 (2.06-4.89)	1.60E-06	7.44E-03			
NFE2L3	2	4.20 (2.68-5.72)	6.13E-08	2.75E-04	4.01 (2.57-5.45)	4.90E-08	2.28E-04	3.03 (0.63-5.43)	1.34E-02	1.00E+00
NFX1	2	2.84 (1.42-4.27)	9.22E-05	4.14E-01	3.30 (1.88-4.71)	4.96E-06	2.30E-02	1.42 (-0.22-3.06)	9.02E-02	1.00E+00
NINL	7	2.02 (1.27-2.77)	1.39E-07	6.24E-04	2.56 (1.81-3.31)	2.28E-11	1.06E-07	1.15 (0.30-2.00)	7.99E-03	1.00E+00
NKAIN2	2	6.51 (4.10-8.91)	1.11E-07	4.99E-04	6.61 (4.65-8.57)	4.09E-11	1.90E-07			
NLRC5	5	1.92 (1.03-2.81)	2.33E-05	1.05E-01	2.44 (1.56-3.33)	6.59E-08	3.06E-04	1.55 (0.49-2.61)	4.15E-03	1.00E+00
NPHS1	4	3.77 (2.72-4.81)	1.57E-12	7.06E-09	2.59 (1.59-3.58)	3.25E-07	1.51E-03	2.34 (0.95-3.73)	9.60E-04	1.00E+00
NPTXR	6	1.50 (0.69-2.31)	2.88E-04	1.00E+00	2.06 (1.25-2.87)	6.56E-07	3.05E-03	1.43 (0.48-2.38)	3.29E-03	1.00E+00
NUMA1	9	2.34 (1.67-3.01)	5.90E-12	2.65E-08	2.86 (2.19-3.52)	0.00E+00	1.54E-13	1.90 (1.06-2.74)	9.37E-06	3.17E-02
NUP188	8	1.67 (0.97-2.37)	3.17E-06	1.42E-02	2.01 (1.31-2.71)	1.95E-08	9.09E-05	1.53 (0.70-2.37)	3.32E-04	1.00E+00
NUP205	2	4.90 (3.25-6.54)	5.18E-09	2.32E-05	4.31 (2.85-5.76)	6.87E-09	3.19E-05			

NUP214	2	4.72 (3.11-6.33)	8.54E-09	3.84E-05	5.01 (3.48-6.53)	1.24E-10	5.78E-07	2.34 (0.38-4.31)	1.95E-02	1.00E+00
OBSCN	5	3.28 (2.37-4.19)	1.94E-12	8.69E-09	3.71 (2.81-4.61)	8.00E-16	3.78E-12	1.55 (0.49-2.61)	4.16E-03	1.00E+00
OBSL1	4	1.85 (0.86-2.85)	2.54E-04	1.00E+00	2.28 (1.29-3.27)	6.33E-06	2.94E-02	1.08 (-0.03-2.20)	5.64E-02	1.00E+00
OGFOD1	2	3.41 (1.96-4.87)	3.98E-06	1.79E-02	4.12 (2.68-5.57)	2.26E-08	1.05E-04			
OGN	2	3.80 (2.32-5.28)	4.82E-07	2.16E-03	3.87 (2.44-5.30)	1.22E-07	5.68E-04			
OPA1	4	2.12 (1.12-3.11)	3.20E-05	1.44E-01	2.58 (1.59-3.57)	3.59E-07	1.67E-03	1.33 (0.18-2.48)	2.34E-02	1.00E+00
OR4F6	3	5.82 (4.21-7.42)	1.27E-12	5.68E-09	5.01 (3.76-6.25)	3.70E-15	1.71E-11			
OR5M9	36	3.44 (3.06-3.82)	0.00E+00	0.00E+00	3.19 (2.82-3.56)	0.00E+00	0.00E+00	3.28 (2.68-3.88)	0.00E+00	0.00E+00
OR5P2	2	5.81 (3.85-7.77)	6.55E-09	2.94E-05	6.58 (4.61-8.54)	5.10E-11	2.37E-07			
OR8B8	6	2.36 (1.54-3.18)	1.59E-08	7.13E-05	2.73 (1.92-3.55)	4.98E-11	2.32E-07	1.14 (0.22-2.06)	1.50E-02	1.00E+00
OTOF	5	2.80 (1.90-3.70)	1.13E-09	5.09E-06	2.95 (2.06-3.84)	8.19E-11	3.81E-07	3.95 (1.80-6.10)	3.15E-04	1.00E+00
OTOG	5	1.47 (0.59-2.35)	1.12E-03	1.00E+00	2.06 (1.17-2.94)	5.11E-06	2.38E-02	2.00 (0.85-3.15)	6.45E-04	1.00E+00
OVOL1	3	3.41 (2.23-4.60)	1.61E-08	7.23E-05	3.11 (1.96-4.26)	1.16E-07	5.41E-04	2.74 (0.95-4.53)	2.68E-03	1.00E+00
PAAF1	10	2.97 (2.32-3.63)	0.00E+00	3.30E-15	2.94 (2.30-3.59)	0.00E+00	2.00E-15	2.38 (1.49-3.27)	1.55E-07	5.25E-04
PACS1	2	4.20 (2.68-5.72)	6.26E-08	2.81E-04	4.12 (2.68-5.57)	2.30E-08	1.07E-04			
PADI2	3	3.15 (1.97-4.32)	1.45E-07	6.53E-04	3.13 (1.98-4.28)	1.03E-07	4.78E-04			
PAK5	7	5.58 (4.55-6.60)	0.00E+00	0.00E+00	4.92 (4.09-5.75)	0.00E+00	0.00E+00			
PANK1	2	5.81 (3.85-7.78)	6.44E-09	2.89E-05	6.20 (4.41-8.00)	1.15E-11	5.37E-08			
PANX2	2	3.33 (1.88-4.77)	6.48E-06	2.91E-02	3.84 (2.40-5.27)	1.51E-07	7.02E-04	3.03 (0.63-5.43)	1.34E-02	1.00E+00
PARVG	2	4.90 (3.25-6.54)	5.16E-09	2.32E-05	4.05 (2.60-5.49)	3.81E-08	1.77E-04			
PCDH12	2	4.71 (3.11-6.32)	8.44E-09	3.79E-05	4.53 (3.06-6.00)	1.70E-09	7.90E-06			
PCDH15	2	5.81 (3.85-7.77)	6.46E-09	2.90E-05	6.20 (4.41-7.99)	1.17E-11	5.43E-08			
PCNX3	3	4.02 (2.80-5.25)	1.25E-10	5.62E-07	4.49 (3.29-5.69)	2.29E-13	1.07E-09	3.44 (1.17-5.70)	2.94E-03	1.00E+00
PCSK6	11	1.93 (1.33-2.53)	2.99E-10	1.34E-06	2.30 (1.70-2.90)	4.96E-14	2.30E-10	2.66 (1.75-3.58)	1.10E-08	3.74E-05
PDZD2	4	1.71 (0.71-2.70)	7.49E-04	1.00E+00	2.25 (1.26-3.25)	8.26E-06	3.84E-02	2.12 (0.80-3.44)	1.64E-03	1.00E+00
PGBD2	2	2.98 (1.55-4.42)	4.48E-05	2.01E-01	3.28 (1.86-4.70)	5.68E-06	2.64E-02	2.34 (0.38-4.31)	1.95E-02	1.00E+00
PGR	2	5.81 (3.85-7.77)	6.46E-09	2.90E-05	6.61 (4.65-8.57)	4.10E-11	1.91E-07			
PHACTR4	18	3.45 (2.95-3.95)	0.00E+00	0.00E+00	3.13 (2.65-3.61)	0.00E+00	0.00E+00	1.90 (1.30-2.49)	5.02E-10	1.70E-06
PHF3	3	6.22 (4.43-8.01)	1.01E-11	4.56E-08	7.01 (5.22-8.81)	1.64E-14	7.62E-11			
PHLDA1	3	3.58 (2.39-4.78)	4.24E-09	1.91E-05	3.95 (2.77-5.12)	4.43E-11	2.06E-07			
PHOX2B	15	1.96 (1.44-2.48)	1.23E-13	5.52E-10	2.18 (1.66-2.69)	1.00E-16	6.88E-13	1.73 (1.10-2.36)	8.44E-08	2.86E-04

PHRF1	2	6.51 (4.10-8.91)	1.11E-07	5.00E-04	7.30 (4.90-9.71)	2.56E-09	1.19E-05	2.34 (0.37-4.30)	1.96E-02	1.00E+00
PIDD1	6	3.06 (2.23-3.89)	5.34E-13	2.40E-09	3.28 (2.46-4.10)	5.00E-15	2.34E-11	1.50 (0.54-2.46)	2.28E-03	1.00E+00
PIEZO2	2	5.81 (3.85-7.78)	6.44E-09	2.89E-05	6.61 (4.65-8.57)	4.09E-11	1.90E-07	3.03 (0.63-5.43)	1.34E-02	1.00E+00
PIGR	2	3.52 (2.05-4.98)	2.39E-06	1.07E-02	4.01 (2.57-5.46)	5.08E-08	2.36E-04	1.94 (0.14-3.73)	3.45E-02	1.00E+00
PIWIL3	3	2.87 (1.70-4.03)	1.37E-06	6.13E-03	3.49 (2.33-4.65)	3.66E-09	1.70E-05			
PKD1L1	8	1.31 (0.61-2.01)	2.53E-04	1.00E+00	1.67 (0.97-2.37)	2.78E-06	1.29E-02	1.13 (0.33-1.92)	5.42E-03	1.00E+00
PKHD1L1	7	1.50 (0.75-2.25)	8.63E-05	3.88E-01	1.83 (1.09-2.58)	1.49E-06	6.91E-03	2.09 (1.10-3.08)	3.57E-05	1.21E-01
PLA2G4E	4	2.19 (1.20-3.19)	1.56E-05	6.99E-02	2.81 (1.82-3.81)	2.83E-08	1.32E-04	1.64 (0.44-2.85)	7.37E-03	1.00E+00
PLEKHA6	2	3.87 (2.38-5.35)	3.33E-07	1.49E-03	3.97 (2.53-5.41)	6.21E-08	2.89E-04			
PLEKHG5	2	5.81 (3.85-7.77)	6.46E-09	2.90E-05	6.61 (4.65-8.57)	4.10E-11	1.91E-07			
PLXNA3	2	3.12 (1.67-4.57)	2.42E-05	1.09E-01	3.60 (2.16-5.03)	9.04E-07	4.20E-03	3.03 (0.63-5.43)	1.34E-02	1.00E+00
PLXNB1	2	3.51 (2.05-4.97)	2.34E-06	1.05E-02	3.97 (2.53-5.41)	6.24E-08	2.90E-04			
PNPLA7	3	5.81 (4.21-7.41)	1.17E-12	5.23E-09	5.92 (4.53-7.30)	1.00E-16	3.11E-13			
POLN	3	3.45 (2.26-4.63)	1.25E-08	5.61E-05	3.03 (1.88-4.18)	2.50E-07	1.16E-03	3.44 (1.17-5.70)	2.94E-03	1.00E+00
POLR3B	2	3.80 (2.32-5.28)	4.81E-07	2.16E-03	4.47 (3.00-5.94)	2.43E-09	1.13E-05	1.93 (0.14-3.72)	3.46E-02	1.00E+00
POLR3E	2	4.11 (2.60-5.62)	9.75E-08	4.38E-04	4.66 (3.18-6.15)	7.51E-10	3.49E-06	3.03 (0.63-5.43)	1.34E-02	1.00E+00
PPAN	3	2.92 (1.76-4.09)	8.96E-07	4.02E-03	3.22 (2.07-4.37)	4.50E-08	2.09E-04	2.34 (0.74-3.94)	4.25E-03	1.00E+00
PPP1R26	2	5.12 (3.42-6.82)	3.61E-09	1.62E-05	5.92 (4.22-7.62)	9.09E-12	4.23E-08			
PRKAR1B	2				7.30 (4.90-9.71)	2.56E-09	1.19E-05			
PRMT8	2	6.51 (4.11-8.92)	1.11E-07	5.00E-04	7.31 (4.90-9.71)	2.57E-09	1.20E-05			
PROM2	8	1.32 (0.62-2.01)	2.26E-04	1.00E+00	1.84 (1.14-2.54)	2.49E-07	1.16E-03	1.53 (0.69-2.36)	3.37E-04	1.00E+00
PRR12	4	5.39 (4.12-6.66)	1.00E-16	3.37E-13	4.54 (3.49-5.58)	0.00E+00	7.35E-14	3.03 (1.33-4.73)	4.71E-04	1.00E+00
PRR14L	3	4.51 (3.23-5.79)	4.67E-12	2.10E-08	2.73 (1.59-3.88)	3.00E-06	1.40E-02	2.34 (0.74-3.94)	4.25E-03	1.00E+00
PRRC2A	2	3.33 (1.88-4.77)	6.47E-06	2.91E-02	3.87 (2.44-5.30)	1.22E-07	5.70E-04			
PRSS36	2	3.51 (2.05-4.97)	2.34E-06	1.05E-02	4.12 (2.68-5.57)	2.25E-08	1.05E-04			
PSMB2	5	2.30 (1.40-3.21)	5.29E-07	2.38E-03	2.63 (1.74-3.53)	8.42E-09	3.92E-05	1.57 (0.50-2.63)	4.06E-03	1.00E+00
PSMD8	8	2.56 (1.85-3.27)	2.03E-12	9.13E-09	2.35 (1.64-3.05)	6.89E-11	3.20E-07	0.99 (0.21-1.78)	1.32E-02	1.00E+00
PSMF1	4	1.94 (0.94-2.93)	1.33E-04	5.98E-01	2.40 (1.41-3.39)	2.02E-06	9.39E-03	2.12 (0.80-3.43)	1.65E-03	1.00E+00
PTCH1	5	2.57 (1.67-3.47)	1.89E-08	8.47E-05	2.29 (1.41-3.18)	3.82E-07	1.78E-03	1.31 (0.28-2.33)	1.23E-02	1.00E+00
PTPDC1	2	3.80 (2.32-5.28)	4.81E-07	2.16E-03	3.72 (2.29-5.15)	3.27E-07	1.52E-03			
PTPN13	3	5.30 (3.87-6.74)	4.23E-13	1.90E-09	4.76 (3.54-5.98)	2.01E-14	9.34E-11	1.64 (0.26-3.03)	2.03E-02	1.00E+00

PTPN23	4	1.94 (0.95-2.93)	1.28E-04	5.75E-01	2.31 (1.32-3.30)	4.83E-06	2.25E-02	3.72 (1.53-5.92)	8.71E-04	1.00E+00
PTPRJ	3	3.17 (2.00-4.35)	1.18E-07	5.29E-04	3.34 (2.18-4.49)	1.51E-08	7.02E-05	3.44 (1.17-5.70)	2.94E-03	1.00E+00
PTPRK	2	5.12 (3.42-6.82)	3.63E-09	1.63E-05	5.69 (4.05-7.33)	1.12E-11	5.20E-08			
PTPRT	2				5.36 (3.78-6.94)	2.72E-11	1.27E-07	1.94 (0.14-3.73)	3.45E-02	1.00E+00
PTPRU	2	2.95 (1.52-4.38)	5.17E-05	2.32E-01	3.69 (2.27-5.12)	3.92E-07	1.82E-03	3.03 (0.63-5.43)	1.34E-02	1.00E+00
PYGM	7	1.50 (0.75-2.25)	8.60E-05	3.86E-01	1.69 (0.94-2.44)	9.69E-06	4.51E-02	0.85 (0.03-1.68)	4.29E-02	1.00E+00
RAB2A	4	4.49 (3.38-5.60)	2.50E-15	1.12E-11	4.26 (3.22-5.30)	7.00E-16	3.42E-12	2.64 (1.14-4.14)	5.82E-04	1.00E+00
RAD50	2				5.22 (3.67-6.78)	4.30E-11	2.00E-07	1.93 (0.14-3.72)	3.46E-02	1.00E+00
RAI14	2	5.41 (3.61-7.20)	3.34E-09	1.50E-05	5.92 (4.22-7.62)	9.14E-12	4.25E-08			
RBBP6	2	2.92 (1.49-4.35)	6.04E-05	2.71E-01	3.69 (2.26-5.12)	3.96E-07	1.84E-03	3.03 (0.63-5.43)	1.34E-02	1.00E+00
RBP3	2	5.81 (3.85-7.78)	6.43E-09	2.89E-05	5.69 (4.05-7.34)	1.10E-11	5.12E-08			
RCC1	3	5.30 (3.87-6.74)	4.25E-13	1.91E-09	5.92 (4.53-7.30)	1.00E-16	3.14E-13			
REV1	4	2.75 (1.74-3.75)	8.76E-08	3.93E-04	2.92 (1.93-3.92)	8.77E-09	4.08E-05	2.34 (0.95-3.73)	9.60E-04	1.00E+00
RFLNA	3	2.46 (1.30-3.61)	3.03E-05	1.36E-01	2.80 (1.65-3.95)	1.79E-06	8.31E-03	2.05 (0.55-3.55)	7.36E-03	1.00E+00
RFLNB	11	1.21 (0.61-1.81)	7.59E-05	3.41E-01	1.36 (0.76-1.95)	8.34E-06	3.88E-02	1.34 (0.65-2.04)	1.56E-04	5.30E-01
RGL4	2	2.74 (1.32-4.17)	1.54E-04	6.92E-01	3.41 (1.99-4.83)	2.40E-06	1.12E-02	1.08 (-0.49-2.66)	1.77E-01	1.00E+00
RGS7	2	3.62 (2.15-5.09)	1.34E-06	6.00E-03	4.22 (2.76-5.67)	1.34E-08	6.23E-05			
RIPOR1	2	4.11 (2.60-5.62)	9.71E-08	4.36E-04	4.12 (2.68-5.57)	2.26E-08	1.05E-04	3.03 (0.63-5.43)	1.34E-02	1.00E+00
RNPEP	4	2.46 (1.45-3.47)	1.69E-06	7.58E-03	2.70 (1.70-3.70)	1.24E-07	5.76E-04	1.34 (0.18-2.49)	2.32E-02	1.00E+00
ROBO2	3	4.97 (3.61-6.32)	6.87E-13	3.08E-09	4.71 (3.50-5.93)	3.13E-14	1.46E-10	1.83 (0.39-3.26)	1.25E-02	1.00E+00
SCART1	3	4.61 (3.32-5.90)	2.81E-12	1.26E-08	4.93 (3.70-6.17)	5.00E-15	2.34E-11	3.44 (1.17-5.70)	2.94E-03	1.00E+00
SCIN	2	4.90 (3.25-6.54)	5.18E-09	2.33E-05	5.51 (3.91-7.11)	1.62E-11	7.55E-08			
SCNN1G	2	2.98 (1.55-4.42)	4.48E-05	2.01E-01	3.44 (2.02-4.86)	2.16E-06	1.00E-02	1.94 (0.14-3.73)	3.45E-02	1.00E+00
SDK2	7	3.61 (2.82-4.40)	0.00E+00	1.40E-15	3.88 (3.11-4.65)	0.00E+00	0.00E+00			
SEC14L1	2	3.67 (2.20-5.14)	9.51E-07	4.27E-03	4.41 (2.95-5.88)	3.50E-09	1.63E-05			
SEMA4A	2	4.02 (2.52-5.52)	1.50E-07	6.74E-04	4.82 (3.32-6.32)	3.11E-10	1.45E-06			
SEMA4D	4	2.93 (1.92-3.94)	1.43E-08	6.41E-05	2.44 (1.45-3.44)	1.40E-06	6.50E-03	3.73 (1.54-5.92)	8.62E-04	1.00E+00
SETD1A	5	1.69 (0.80-2.57)	1.93E-04	8.68E-01	2.22 (1.33-3.10)	9.02E-07	4.19E-03	2.56 (1.25-3.88)	1.37E-04	4.64E-01
SFPQ	2	3.37 (1.92-4.82)	5.16E-06	2.32E-02	3.93 (2.50-5.37)	8.00E-08	3.72E-04			
SFRP1	2	3.80 (2.32-5.27)	4.90E-07	2.20E-03	3.66 (2.23-5.08)	4.91E-07	2.28E-03			
SHOC1	2	3.51 (2.05-4.97)	2.34E-06	1.05E-02	4.01 (2.57-5.45)	4.91E-08	2.29E-04			

SKI	2	4.42 (2.87-5.98)	2.34E-08	1.05E-04	5.10 (3.57-6.64)	7.30E-11	3.39E-07			
SKIDA1	3	2.71 (1.55-3.88)	4.75E-06	2.13E-02	3.04 (1.89-4.19)	2.33E-07	1.09E-03			
SLC16A5	6	1.76 (0.94-2.58)	2.59E-05	1.16E-01	2.23 (1.41-3.05)	8.99E-08	4.18E-04	1.15 (0.23-2.07)	1.47E-02	1.00E+00
SLC22A5	2	2.64 (1.22-4.06)	2.74E-04	1.00E+00	3.30 (1.88-4.72)	5.06E-06	2.36E-02	2.34 (0.38-4.31)	1.95E-02	1.00E+00
SLC25A23	2				4.31 (2.85-5.76)	6.87E-09	3.20E-05			
SLC39A6	2	4.43 (2.87-5.98)	2.30E-08	1.03E-04	3.31 (1.90-4.73)	4.41E-06	2.05E-02			
SLC4A11	2				7.30 (4.90-9.71)	2.56E-09	1.19E-05			
SLC4A5	2	4.53 (2.95-6.10)	1.74E-08	7.82E-05	4.23 (2.77-5.68)	1.20E-08	5.58E-05			
SLC5A9	16	2.81 (2.30-3.33)	0.00E+00	0.00E+00	2.70 (2.20-3.21)	0.00E+00	0.00E+00	2.66 (1.90-3.42)	5.35E-12	1.81E-08
SLC9A1	2	6.51 (4.10-8.91)	1.11E-07	5.00E-04	7.30 (4.90-9.71)	2.56E-09	1.19E-05			
SLC9A3R1	2	5.81 (3.85-7.78)	6.42E-09	2.89E-05	5.92 (4.22-7.62)	9.06E-12	4.21E-08			
SLIT2	2	6.51 (4.11-8.92)	1.11E-07	5.00E-04	6.62 (4.65-8.58)	4.17E-11	1.94E-07	3.04 (0.63-5.44)	1.34E-02	1.00E+00
SMIM32	4	3.89 (2.79-5.00)	4.89E-12	2.20E-08	3.55 (2.52-4.58)	1.41E-11	6.55E-08			
SNAPC4	4	2.11 (1.12-3.11)	3.19E-05	1.43E-01	2.35 (1.36-3.34)	3.23E-06	1.50E-02	2.34 (0.95-3.73)	9.62E-04	1.00E+00
SORL1	2				6.61 (4.65-8.57)	4.09E-11	1.90E-07			
SOX8	37	4.43 (4.06-4.80)	0.00E+00	0.00E+00	4.15 (3.81-4.49)	0.00E+00	0.00E+00			
SPATA13	3	2.60 (1.44-3.75)	1.12E-05	5.05E-02	2.80 (1.66-3.95)	1.70E-06	7.89E-03	0.95 (-0.32-2.22)	1.42E-01	1.00E+00
SPEG	4	2.18 (1.19-3.18)	1.76E-05	7.88E-02	2.58 (1.58-3.57)	3.56E-07	1.65E-03	1.65 (0.44-2.85)	7.36E-03	1.00E+00
SPPL2B	2	5.81 (3.85-7.77)	6.44E-09	2.89E-05	4.82 (3.32-6.32)	3.09E-10	1.44E-06			
SPTB	9	2.87 (2.19-3.54)	1.00E-16	3.73E-13	3.11 (2.44-3.78)	0.00E+00	3.00E-16	3.45 (2.14-4.76)	2.48E-07	8.41E-04
SRGAP3	17	5.43 (4.81-6.06)	0.00E+00	0.00E+00	5.31 (4.76-5.85)	0.00E+00	0.00E+00			
SRL	2	6.51 (4.10-8.91)	1.11E-07	5.00E-04	6.20 (4.41-8.00)	1.16E-11	5.39E-08			
SUGP2	3	6.22 (4.43-8.01)	1.01E-11	4.55E-08	6.10 (4.66-7.53)	1.00E-16	3.53E-13			
SUPT20H	2				4.31 (2.85-5.76)	6.90E-09	3.21E-05			
SYNE1	11	1.86 (1.26-2.46)	1.20E-09	5.37E-06	2.13 (1.53-2.73)	2.73E-12	1.27E-08	1.30 (0.61-1.99)	2.15E-04	7.28E-01
SYNE2	7	3.95 (3.15-4.75)	0.00E+00	0.00E+00	3.50 (2.75-4.26)	0.00E+00	7.00E-16	2.49 (1.40-3.58)	7.70E-06	2.61E-02
SYNE3	5	5.62 (4.43-6.81)	0.00E+00	1.00E-16	5.21 (4.23-6.20)	0.00E+00	0.00E+00			
SYTL3	2	3.37 (1.92-4.82)	5.10E-06	2.29E-02	3.31 (1.90-4.73)	4.43E-06	2.06E-02	1.08 (-0.49-2.66)	1.77E-01	1.00E+00
TANC2	4	3.07 (2.05-4.08)	3.36E-09	1.51E-05	2.81 (1.82-3.81)	3.10E-08	1.44E-04	3.04 (1.34-4.74)	4.67E-04	1.00E+00
TAS1R2	2	3.80 (2.32-5.28)	4.81E-07	2.16E-03	4.41 (2.95-5.88)	3.49E-09	1.62E-05			
TAS1R3	4	4.06 (3.00-5.13)	7.22E-14	3.24E-10	4.56 (3.52-5.61)	0.00E+00	4.93E-14	2.11 (0.80-3.43)	1.65E-03	1.00E+00

TAT	2	3.37 (1.92-4.83)	5.21E-06	2.34E-02	3.50 (2.08-4.92)	1.43E-06	6.67E-03			
TBC1D30	2	5.81 (3.85-7.78)	6.43E-09	2.89E-05	6.61 (4.65-8.57)	4.08E-11	1.90E-07	3.03 (0.63-5.43)	1.34E-02	1.00E+00
TCHH	7	1.71 (0.96-2.46)	7.71E-06	3.46E-02	2.12 (1.37-2.87)	2.78E-08	1.29E-04	2.67 (1.53-3.82)	5.07E-06	1.72E-02
TCOF1	5	1.91 (1.01-2.80)	3.09E-05	1.39E-01	2.45 (1.56-3.35)	7.88E-08	3.66E-04	2.87 (1.43-4.31)	9.36E-05	3.17E-01
TECPR1	12	3.39 (2.79-3.99)	0.00E+00	0.00E+00	3.04 (2.46-3.62)	0.00E+00	0.00E+00			
TFEB	2	5.81 (3.85-7.77)	6.46E-09	2.90E-05	6.20 (4.41-8.00)	1.16E-11	5.39E-08			
TGS1	3	3.30 (2.12-4.48)	4.15E-08	1.86E-04	2.96 (1.81-4.11)	4.33E-07	2.01E-03	2.74 (0.95-4.53)	2.68E-03	1.00E+00
THAP8	2	4.11 (2.60-5.63)	9.96E-08	4.47E-04	3.30 (1.88-4.72)	5.05E-06	2.35E-02	3.04 (0.63-5.44)	1.34E-02	1.00E+00
TIMM17B	2	4.65 (3.00-6.30)	3.09E-08	1.39E-04	4.11 (2.64-5.58)	4.01E-08	1.86E-04			
TJP2	2	4.71 (3.11-6.32)	8.28E-09	3.72E-05	5.22 (3.67-6.78)	4.28E-11	1.99E-07			
TMEM187	2	3.44 (1.96-4.91)	4.77E-06	2.14E-02	4.07 (2.61-5.54)	4.89E-08	2.28E-04	3.04 (0.63-5.44)	1.34E-02	1.00E+00
TMEM41A	15	2.58 (2.05-3.10)	0.00E+00	0.00E+00	2.57 (2.05-3.09)	0.00E+00	0.00E+00	1.21 (0.62-1.80)	5.50E-05	1.86E-01
TMTC1	4	2.72 (1.71-3.74)	1.30E-07	5.85E-04	3.42 (2.41-4.43)	3.33E-11	1.55E-07	1.54 (0.35-2.73)	1.11E-02	1.00E+00
TNFRSF10A	13	2.57 (2.01-3.13)	0.00E+00	9.00E-16	2.54 (1.99-3.10)	0.00E+00	1.00E-15	1.38 (0.74-2.03)	2.48E-05	8.40E-02
TNRC6C	2	3.87 (2.38-5.35)	3.33E-07	1.50E-03	4.66 (3.18-6.15)	7.46E-10	3.47E-06			
TNXB	5	2.55 (1.66-3.45)	2.26E-08	1.02E-04	2.46 (1.57-3.35)	5.25E-08	2.44E-04	2.16 (0.97-3.34)	3.80E-04	1.00E+00
TOM1	2	4.43 (2.87-5.98)	2.30E-08	1.03E-04	5.22 (3.67-6.78)	4.28E-11	1.99E-07			
TOPBP1	2	5.81 (3.85-7.78)	6.44E-09	2.89E-05	5.22 (3.67-6.78)	4.33E-11	2.01E-07			
TRANK1	4	2.28 (1.28-3.28)	7.35E-06	3.30E-02	2.71 (1.71-3.70)	9.09E-08	4.23E-04	1.65 (0.44-2.85)	7.36E-03	1.00E+00
TRHR	5	2.64 (1.74-3.55)	1.08E-08	4.83E-05	2.70 (1.80-3.60)	3.52E-09	1.64E-05	1.48 (0.42-2.53)	5.99E-03	1.00E+00
TRPM1	6	1.38 (0.58-2.19)	7.84E-04	1.00E+00	1.85 (1.05-2.66)	6.71E-06	3.12E-02	1.09 (0.18-2.00)	1.94E-02	1.00E+00
TRPM2	3	2.00 (0.85-3.15)	6.40E-04	1.00E+00	2.63 (1.48-3.78)	6.96E-06	3.23E-02	1.49 (0.14-2.85)	3.11E-02	1.00E+00
TRPM3	2	5.41 (3.62-7.21)	3.41E-09	1.53E-05	5.36 (3.78-6.94)	2.72E-11	1.26E-07	3.04 (0.63-5.44)	1.34E-02	1.00E+00
TRPM8	3	5.53 (4.03-7.03)	5.15E-13	2.31E-09	3.74 (2.57-4.91)	3.39E-10	1.58E-06	2.75 (0.95-4.54)	2.68E-03	1.00E+00
TSHZ2	3	2.36 (1.20-3.51)	6.11E-05	2.74E-01	2.98 (1.83-4.13)	3.76E-07	1.75E-03	1.36 (0.03-2.69)	4.56E-02	1.00E+00
TTLL8	7	1.64 (0.89-2.39)	1.76E-05	7.89E-02	1.77 (1.03-2.52)	3.30E-06	1.54E-02	0.82 (0.00-1.64)	5.08E-02	1.00E+00
TTN	28	1.73 (1.36-2.11)	0.00E+00	5.00E-16	1.53 (1.16-1.91)	7.00E-16	3.36E-12	1.51 (1.07-1.96)	2.87E-11	9.72E-08
TXNRD2	3	2.47 (1.31-3.63)	2.80E-05	1.26E-01	2.98 (1.83-4.13)	3.79E-07	1.76E-03	2.75 (0.95-4.54)	2.68E-03	1.00E+00
ULK4	2	6.51 (4.10-8.91)	1.11E-07	4.99E-04	5.36 (3.78-6.93)	2.59E-11	1.20E-07			
UNC13C	28	2.99 (2.59-3.39)	0.00E+00	0.00E+00	2.88 (2.49-3.27)	0.00E+00	0.00E+00	2.44 (1.90-2.98)	0.00E+00	2.80E-15
UNC13D	2	3.29 (1.84-4.74)	8.31E-06	3.73E-02	3.84 (2.41-5.28)	1.57E-07	7.29E-04	0.32 (-1.16-1.80)	6.71E-01	1.00E+00

USH1C	2	4.56 (2.98-6.13)	1.41E-08	6.34E-05	5.22 (3.66-6.77)	4.60E-11	2.14E-07	3.03 (0.63-5.43)	1.34E-02	1.00E+00
USH2A	12	1.54 (0.97-2.12)	1.33E-07	5.98E-04	1.67 (1.10-2.24)	1.04E-08	4.82E-05	1.36 (0.69-2.03)	6.28E-05	2.13E-01
USHBP1	2	3.51 (2.05-4.97)	2.34E-06	1.05E-02	3.94 (2.50-5.37)	7.89E-08	3.67E-04			
USP28	2	3.07 (1.64-4.51)	2.67E-05	1.20E-01	3.72 (2.29-5.15)	3.27E-07	1.52E-03	3.03 (0.63-5.43)	1.34E-02	1.00E+00
USP34	2	6.51 (4.10-8.91)	1.11E-07	4.99E-04	6.20 (4.41-8.00)	1.15E-11	5.36E-08			
UTP20	7	1.98 (1.23-2.74)	2.47E-07	1.11E-03	1.92 (1.17-2.67)	4.91E-07	2.28E-03	2.09 (1.10-3.08)	3.53E-05	1.20E-01
UTRN	4	3.77 (2.72-4.82)	1.63E-12	7.31E-09	4.36 (3.33-5.40)	1.00E-16	6.72E-13	2.12 (0.80-3.44)	1.64E-03	1.00E+00
VEZF1	4	2.24 (1.24-3.24)	1.06E-05	4.74E-02	2.66 (1.67-3.65)	1.44E-07	6.72E-04	2.63 (1.13-4.12)	5.96E-04	1.00E+00
VSIG8	5	3.60 (2.67-4.54)	4.07E-14	1.83E-10	3.37 (2.46-4.27)	3.02E-13	1.41E-09			
WDR64	2	4.43 (2.87-5.98)	2.31E-08	1.04E-04	4.41 (2.95-5.88)	3.51E-09	1.63E-05			
WDR72	2	4.43 (2.87-5.98)	2.30E-08	1.03E-04	4.82 (3.32-6.32)	3.08E-10	1.43E-06			
WDR87	3	4.96 (3.61-6.32)	6.89E-13	3.10E-09	4.87 (3.64-6.10)	8.20E-15	3.79E-11			
WFIKKN2	6	1.73 (0.92-2.54)	2.90E-05	1.30E-01	2.10 (1.29-2.91)	3.53E-07	1.64E-03	2.19 (1.09-3.28)	8.86E-05	3.00E-01
WNK2	43	3.09 (2.76-3.42)	0.00E+00	0.00E+00	2.86 (2.54-3.18)	0.00E+00	0.00E+00			
WNT10A	3	2.34 (1.19-3.49)	7.05E-05	3.17E-01	2.64 (1.49-3.78)	6.62E-06	3.08E-02	1.49 (0.14-2.85)	3.11E-02	1.00E+00
WWC2	2	5.12 (3.42-6.82)	3.61E-09	1.62E-05	5.00 (3.48-6.52)	1.19E-10	5.52E-07			
XPO4	2	6.51 (4.10-8.91)	1.11E-07	4.99E-04	7.30 (4.90-9.71)	2.56E-09	1.19E-05			
YIPF3	2	3.67 (2.20-5.14)	9.52E-07	4.27E-03	3.97 (2.53-5.41)	6.23E-08	2.90E-04	3.03 (0.63-5.43)	1.34E-02	1.00E+00
YLPM1	3	5.30 (3.87-6.73)	4.27E-13	1.92E-09	5.63 (4.30-6.96)	1.00E-16	5.06E-13	3.44 (1.17-5.70)	2.94E-03	1.00E+00
ZC3HAV1	2	2.98 (1.55-4.41)	4.42E-05	1.98E-01	3.56 (2.14-4.99)	8.97E-07	4.17E-03	3.03 (0.63-5.43)	1.34E-02	1.00E+00
ZFP1	5	1.95 (1.05-2.84)	2.11E-05	9.47E-02	2.27 (1.38-3.17)	6.12E-07	2.84E-03	1.01 (0.02-2.01)	4.59E-02	1.00E+00
ZNF124	3	2.60 (1.44-3.77)	1.14E-05	5.13E-02	3.28 (2.12-4.44)	3.12E-08	1.45E-04	2.35 (0.74-3.95)	4.21E-03	1.00E+00
ZNF132	2	3.40 (1.95-4.85)	4.59E-06	2.06E-02	3.31 (1.89-4.72)	4.78E-06	2.22E-02			
ZNF142	5	3.47 (2.55-4.39)	1.44E-13	6.48E-10	2.38 (1.50-3.27)	1.32E-07	6.12E-04	3.26 (1.61-4.90)	1.01E-04	3.44E-01
ZNF212	2	5.80 (3.84-7.77)	7.05E-09	3.17E-05	4.99 (3.46-6.51)	1.44E-10	6.70E-07			
ZNF234	2	6.51 (4.10-8.91)	1.11E-07	4.99E-04	7.30 (4.90-9.71)	2.56E-09	1.19E-05			
ZNF337	2	2.74 (1.32-4.16)	1.60E-04	7.21E-01	3.22 (1.80-4.63)	8.15E-06	3.79E-02	2.34 (0.37-4.30)	1.96E-02	1.00E+00
ZNF407	2	4.02 (2.52-5.52)	1.50E-07	6.74E-04	4.47 (3.00-5.94)	2.45E-09	1.14E-05			
ZNF451	3	4.35 (3.09-5.60)	1.28E-11	5.73E-08	4.53 (3.33-5.73)	1.57E-13	7.32E-10	2.74 (0.95-4.53)	2.68E-03	1.00E+00
ZNF644	2	3.08 (1.64-4.51)	2.72E-05	1.22E-01	3.40 (1.98-4.82)	2.79E-06	1.30E-02			
ZNF679	2	2.62 (1.20-4.04)	3.04E-04	1.00E+00	3.32 (1.90-4.74)	4.51E-06	2.10E-02	1.65 (-0.06-3.35)	5.80E-02	1.00E+00

ZNF680	2	3.62 (2.15-5.09)	1.35E-06	6.05E-03	3.62 (2.19-5.05)	6.72E-07	3.13E-03			
ZNF777	4	2.34 (1.33-3.34)	5.13E-06	2.31E-02	2.91 (1.91-3.91)	1.25E-08	5.81E-05	1.79 (0.55-3.03)	4.56E-03	1.00E+00
ZNF781	2	3.80 (2.32-5.29)	4.93E-07	2.21E-03	4.48 (3.00-5.95)	2.54E-09	1.18E-05	2.34 (0.38-4.31)	1.95E-02	1.00E+00
ZNF786	4	3.22 (2.20-4.25)	7.13E-10	3.20E-06	3.72 (2.70-4.74)	7.23E-13	3.36E-09	1.79 (0.55-3.03)	4.56E-03	1.00E+00
ZNF862	2	4.20 (2.68-5.72)	6.14E-08	2.76E-04	4.82 (3.32-6.32)	3.09E-10	1.43E-06			
ZPR1	11	1.91 (1.30-2.51)	5.30E-10	2.38E-06	2.27 (1.67-2.87)	1.06E-13	4.91E-10	1.97 (1.20-2.74)	5.50E-07	1.86E-03

Gene	N Variants	logOR (CI) NFE	pvalue	corrected	logOR (CI) ALL	pvalue	corrected	logOR (CI) Swedish	pvalue	corrected
ABCA13	8	1.25 (0.55-1.95)	4.76E-04	1.00E+00	1.70 (1.00-2.40)	1.93E-06	9.94E-03	1.17 (0.43-1.91)	1.83E-03	1.00E+00
ABCA5	4	2.84 (1.85-3.84)	2.21E-08	1.10E-04	3.35 (2.35-4.34)	4.01E-11	2.06E-07	2.70 (1.50-3.91)	1.13E-05	4.85E-02
ABCC10	3	6.18 (4.78-7.57)	0.00E+00	1.77E-14	3.65 (2.50-4.80)	5.08E-10	2.61E-06	4.49 (2.23-6.76)	1.02E-04	4.38E-01
ABCC6	3	4.44 (3.25-5.63)	2.36E-13	1.18E-09	4.34 (3.18-5.50)	2.37E-13	1.21E-09			
ABCC8	2				8.36 (5.95-10.77)	9.79E-12	5.03E-08			
ABCG2	3	2.37 (1.22-3.52)	5.45E-05	2.72E-01	2.75 (1.60-3.90)	2.62E-06	1.34E-02	3.12 (1.61-4.62)	5.09E-05	2.18E-01
ABI3BP	7	1.88 (1.13-2.63)	9.60E-07	4.79E-03	2.16 (1.41-2.91)	1.78E-08	9.16E-05	2.78 (1.86-3.71)	3.81E-09	1.63E-05
ACAN	3	3.20 (2.04-4.35)	6.34E-08	3.17E-04	3.44 (2.29-4.60)	4.65E-09	2.39E-05	2.20 (0.90-3.50)	9.39E-04	1.00E+00
ACOT6	2	2.58 (1.17-3.98)	3.23E-04	1.00E+00	3.22 (1.82-4.63)	6.77E-06	3.48E-02	2.70 (1.00-4.41)	1.91E-03	1.00E+00
ACSM6	2	3.90 (2.47-5.33)	9.22E-08	4.61E-04	3.33 (1.92-4.74)	3.41E-06	1.75E-02	3.40 (1.43-5.36)	7.18E-04	1.00E+00
ADAM20	2	5.62 (4.04-7.20)	3.25E-12	1.63E-08	6.41 (4.83-8.00)	1.80E-15	9.22E-12			
ADAM8	2	2.62 (1.21-4.03)	2.59E-04	1.00E+00	3.21 (1.80-4.61)	7.64E-06	3.93E-02	3.40 (1.43-5.36)	7.18E-04	1.00E+00
ADAMTS16	6	1.75 (0.94-2.57)	2.45E-05	1.22E-01	2.30 (1.49-3.11)	3.03E-08	1.56E-04	1.59 (0.71-2.46)	3.77E-04	1.00E+00
ADAMTSL2	2	2.90 (1.48-4.31)	6.38E-05	3.19E-01	3.56 (2.14-4.97)	8.88E-07	4.56E-03	3.41 (1.43-5.39)	7.15E-04	1.00E+00
ADAT2	2				8.36 (5.95-10.77)	9.81E-12	5.04E-08			
ADGRB1	3	6.18 (4.78-7.57)	0.00E+00	1.77E-14	6.82 (5.46-8.18)	0.00E+00	0.00E+00	3.11 (1.60-4.61)	5.09E-05	2.18E-01
ADGRV1	8	1.60 (0.90-2.30)	8.03E-06	4.01E-02	1.92 (1.22-2.62)	8.02E-08	4.12E-04	1.13 (0.40-1.87)	2.57E-03	1.00E+00
ADH1B	6	1.80 (0.99-2.61)	1.36E-05	6.81E-02	1.89 (1.08-2.70)	4.83E-06	2.48E-02	2.06 (1.15-2.96)	8.73E-06	3.73E-02
AEBP2	2	3.63 (2.21-5.05)	5.76E-07	2.88E-03	3.44 (2.03-4.84)	1.66E-06	8.52E-03	2.14 (0.56-3.72)	7.92E-03	1.00E+00
AHNAK	8	1.50 (0.80-2.20)	2.62E-05	1.31E-01	1.67 (0.98-2.37)	2.71E-06	1.39E-02	1.08 (0.35-1.81)	3.92E-03	1.00E+00
AIRE	3	3.53 (2.37-4.70)	2.70E-09	1.35E-05	2.93 (1.79-4.08)	5.60E-07	2.87E-03	1.66 (0.43-2.90)	8.47E-03	1.00E+00

AK9	3	4.31 (3.12-5.49)	9.57E-13	4.78E-09	4.25 (3.10-5.41)	6.05E-13	3.11E-09	4.49 (2.23-6.76)	1.02E-04	4.38E-01
AKAP17A	6	2.10 (1.28-2.91)	4.72E-07	2.36E-03	2.11 (1.29-2.92)	3.86E-07	1.98E-03	1.67 (0.79-2.55)	1.96E-04	8.38E-01
AKAP9	2	2.79 (1.38-4.20)	1.01E-04	5.07E-01	3.48 (2.08-4.89)	1.20E-06	6.17E-03	2.70 (1.00-4.41)	1.91E-03	1.00E+00
AKT2	2				5.65 (4.17-7.14)	8.64E-14	4.44E-10			
ALKBH1	2	5.17 (3.65-6.68)	2.41E-11	1.21E-07	5.14 (3.69-6.59)	3.66E-12	1.88E-08			
ALMS1	6	1.84 (1.04-2.65)	7.85E-06	3.92E-02	2.21 (1.40-3.02)	8.08E-08	4.15E-04	1.93 (1.03-2.82)	2.34E-05	1.00E-01
ALPK3	5	2.23 (1.34-3.12)	9.00E-07	4.49E-03	2.39 (1.50-3.27)	1.32E-07	6.78E-04	1.17 (0.24-2.11)	1.34E-02	1.00E+00
ALS2	5	2.69 (1.80-3.58)	3.21E-09	1.60E-05	2.51 (1.62-3.40)	2.98E-08	1.53E-04	1.45 (0.50-2.39)	2.64E-03	1.00E+00
ANK2	3	2.58 (1.43-3.73)	1.12E-05	5.61E-02	3.03 (1.88-4.18)	2.41E-07	1.24E-03	1.55 (0.32-2.78)	1.33E-02	1.00E+00
ANKRD31	3	5.89 (4.55-7.22)	0.00E+00	2.55E-14	6.69 (5.35-8.02)	0.00E+00	0.00E+00			
ANO9	2	6.18 (4.47-7.88)	1.27E-12	6.32E-09	6.42 (4.83-8.00)	1.80E-15	9.18E-12	4.09 (1.68-6.50)	8.68E-04	1.00E+00
APC	2	6.87 (4.90-8.84)	7.63E-12	3.81E-08	7.26 (5.46-9.06)	2.40E-15	1.22E-11			
APOB	10	1.28 (0.65-1.90)	6.48E-05	3.23E-01	1.74 (1.12-2.37)	5.02E-08	2.58E-04	1.41 (0.74-2.07)	3.49E-05	1.49E-01
ARHGEF4	3	5.20 (3.96-6.44)	2.00E-16	1.12E-12	4.71 (3.54-5.87)	3.00E-15	1.53E-11			
ARID1B	2	5.08 (3.57-6.59)	3.91E-11	1.95E-07	4.78 (3.34-6.21)	6.60E-11	3.39E-07			
ARSI	2	3.21 (1.78-4.63)	1.01E-05	5.02E-02	3.81 (2.39-5.23)	1.46E-07	7.52E-04	4.10 (1.69-6.52)	8.58E-04	1.00E+00
ASIC4	3	3.91 (2.74-5.08)	5.57E-11	2.78E-07	3.67 (2.52-4.82)	3.91E-10	2.01E-06			
ATP13A1	3	2.66 (1.51-3.81)	5.98E-06	2.99E-02	3.27 (2.12-4.42)	2.54E-08	1.30E-04	2.01 (0.74-3.29)	1.99E-03	1.00E+00
ATP2C2	3	2.53 (1.38-3.68)	1.55E-05	7.72E-02	2.68 (1.53-3.82)	4.44E-06	2.28E-02	2.70 (1.31-4.10)	1.44E-04	6.14E-01
ATP7B	13	3.16 (2.59-3.73)	0.00E+00	0.00E+00	3.10 (2.54-3.67)	0.00E+00	0.00E+00	2.96 (2.26-3.67)	2.00E-16	8.50E-13
ATR	3	4.04 (2.86-5.21)	1.50E-11	7.50E-08	3.43 (2.28-4.58)	4.86E-09	2.50E-05	3.11 (1.60-4.61)	5.09E-05	2.18E-01
AVIL	3	3.08 (1.93-4.23)	1.65E-07	8.25E-04	3.35 (2.21-4.50)	1.01E-08	5.17E-05	3.80 (2.01-5.60)	3.30E-05	1.41E-01
AXDND1	2	3.00 (1.58-4.42)	3.45E-05	1.72E-01	3.25 (1.84-4.67)	6.71E-06	3.45E-02	3.41 (1.43-5.39)	7.15E-04	1.00E+00
BACH1	2	3.57 (2.15-5.00)	8.28E-07	4.13E-03	4.06 (2.64-5.47)	1.91E-08	9.79E-05			
BAHCC1	4	2.05 (1.05-3.04)	5.22E-05	2.61E-01	2.38 (1.39-3.37)	2.45E-06	1.26E-02	2.14 (1.02-3.26)	1.73E-04	7.41E-01
BDP1	2	7.56 (5.16-9.97)	7.28E-10	3.63E-06	8.36 (5.95-10.77)	9.90E-12	5.09E-08			
BEND7	2	2.89 (1.48-4.30)	5.81E-05	2.90E-01	3.37 (1.96-4.78)	2.61E-06	1.34E-02	3.40 (1.43-5.36)	7.18E-04	1.00E+00
BMP1	2	6.47 (4.67-8.26)	1.78E-12	8.89E-09	7.26 (5.46-9.06)	2.40E-15	1.22E-11			
BRCA2	4	3.34 (2.34-4.34)	6.55E-11	3.27E-07	3.49 (2.50-4.49)	5.88E-12	3.02E-08	2.07 (0.96-3.18)	2.51E-04	1.00E+00
C10orf90	4	3.13 (2.12-4.13)	9.40E-10	4.70E-06	3.29 (2.29-4.29)	9.42E-11	4.84E-07	2.71 (1.50-3.92)	1.13E-05	4.85E-02
C16orf96	9	1.32 (0.66-1.98)	9.24E-05	4.62E-01	1.58 (0.92-2.24)	2.78E-06	1.43E-02	1.25 (0.55-1.95)	4.42E-04	1.00E+00

C3orf56	4	2.68 (1.69-3.67)	1.29E-07	6.43E-04	3.25 (2.26-4.24)	1.40E-10	7.17E-07	2.48 (1.31-3.64)	3.09E-05	1.32E-01
C6	5	1.97 (1.09-2.86)	1.31E-05	6.57E-02	2.27 (1.38-3.16)	5.25E-07	2.70E-03	1.71 (0.75-2.67)	4.93E-04	1.00E+00
C6orf141	2	4.81 (3.32-6.30)	2.56E-10	1.28E-06	4.88 (3.43-6.33)	3.93E-11	2.02E-07	2.49 (0.83-4.15)	3.22E-03	1.00E+00
CA9	2	3.79 (2.35-5.23)	2.49E-07	1.24E-03	3.40 (1.98-4.81)	2.62E-06	1.35E-02	2.72 (1.00-4.43)	1.91E-03	1.00E+00
CABIN1	5	1.82 (0.94-2.71)	5.40E-05	2.70E-01	2.12 (1.24-3.01)	2.51E-06	1.29E-02	2.11 (1.12-3.11)	3.24E-05	1.39E-01
CACNA1A	4	2.16 (1.17-3.16)	2.03E-05	1.01E-01	2.24 (1.25-3.23)	9.64E-06	4.95E-02	1.95 (0.85-3.05)	4.97E-04	1.00E+00
CACNA1E	21	3.97 (3.52-4.42)	0.00E+00	0.00E+00	2.89 (2.45-3.33)	0.00E+00	0.00E+00	3.77 (3.10-4.44)	0.00E+00	0.00E+00
CALCRL	2	2.55 (1.14-3.97)	4.09E-04	1.00E+00	3.20 (1.79-4.62)	8.98E-06	4.61E-02	2.31 (0.69-3.93)	5.17E-03	1.00E+00
CAMSAP2	7	2.01 (1.25-2.76)	1.78E-07	8.91E-04	2.25 (1.49-3.00)	4.90E-09	2.52E-05	2.02 (1.18-2.85)	2.32E-06	9.92E-03
CARD10	2	3.87 (2.44-5.30)	1.10E-07	5.50E-04	4.53 (3.11-5.96)	4.69E-10	2.41E-06	2.14 (0.56-3.72)	7.92E-03	1.00E+00
CASD1	3	2.80 (1.65-3.95)	1.84E-06	9.21E-03	3.41 (2.27-4.56)	5.60E-09	2.88E-05	2.41 (1.08-3.75)	3.91E-04	1.00E+00
CBFA2T2	3	2.22 (1.07-3.37)	1.55E-04	7.74E-01	2.67 (1.52-3.81)	5.35E-06	2.75E-02	3.12 (1.61-4.62)	5.09E-05	2.18E-01
CCDC141	4	3.39 (2.39-4.39)	3.40E-11	1.70E-07	3.45 (2.46-4.45)	9.81E-12	5.04E-08	3.17 (1.85-4.49)	2.49E-06	1.06E-02
CCDC146	3	2.26 (1.10-3.43)	1.35E-04	6.74E-01	2.75 (1.59-3.91)	3.37E-06	1.73E-02	1.25 (0.03-2.47)	4.40E-02	1.00E+00
CCDC168	15	1.36 (0.85-1.88)	1.68E-07	8.41E-04	1.67 (1.16-2.18)	1.39E-10	7.15E-07	0.95 (0.42-1.48)	4.51E-04	1.00E+00
CCDC33	2	3.36 (1.94-4.78)	3.37E-06	1.68E-02	3.56 (2.15-4.96)	7.32E-07	3.76E-03	4.09 (1.68-6.50)	8.68E-04	1.00E+00
CCDC42	2	6.18 (4.47-7.88)	1.27E-12	6.32E-09	3.95 (2.54-5.37)	4.13E-08	2.12E-04	3.40 (1.43-5.36)	7.18E-04	1.00E+00
CCNT1	3	3.30 (2.12-4.47)	3.54E-08	1.77E-04	3.33 (2.17-4.50)	2.06E-08	1.06E-04	2.44 (1.09-3.79)	3.92E-04	1.00E+00
CCP110	2	3.29 (1.87-4.70)	5.32E-06	2.66E-02	3.71 (2.30-5.12)	2.53E-07	1.30E-03	2.48 (0.83-4.13)	3.22E-03	1.00E+00
CCR1	2	3.42 (1.99-4.85)	2.69E-06	1.35E-02	4.04 (2.62-5.47)	2.63E-08	1.35E-04	2.72 (1.00-4.43)	1.91E-03	1.00E+00
CD109	6	1.84 (1.03-2.65)	8.24E-06	4.11E-02	2.36 (1.56-3.17)	1.02E-08	5.26E-05	1.79 (0.90-2.67)	7.35E-05	3.14E-01
CD226	2	2.81 (1.39-4.22)	1.05E-04	5.25E-01	3.40 (1.98-4.81)	2.56E-06	1.31E-02	4.10 (1.69-6.52)	8.58E-04	1.00E+00
CD79A	2	2.73 (1.31-4.15)	1.58E-04	7.90E-01	3.20 (1.78-4.61)	9.37E-06	4.81E-02	4.10 (1.69-6.52)	8.58E-04	1.00E+00
CDHR5	2	3.14 (1.73-4.56)	1.29E-05	6.44E-02	3.48 (2.07-4.88)	1.26E-06	6.48E-03	4.09 (1.68-6.50)	8.68E-04	1.00E+00
CELSR1	4	2.41 (1.41-3.40)	2.13E-06	1.06E-02	2.80 (1.80-3.79)	3.47E-08	1.78E-04	2.48 (1.32-3.65)	3.09E-05	1.32E-01
CELSR2	6	2.40 (1.59-3.22)	7.15E-09	3.57E-05	2.15 (1.34-2.96)	2.11E-07	1.08E-03	2.01 (1.11-2.92)	1.23E-05	5.25E-02
CEP131	3	5.14 (3.90-6.37)	4.00E-16	1.82E-12	5.88 (4.65-7.11)	0.00E+00	0.00E+00	3.11 (1.60-4.61)	5.09E-05	2.18E-01
CEP250	5	1.65 (0.76-2.54)	2.68E-04	1.00E+00	2.03 (1.14-2.92)	7.56E-06	3.89E-02	1.20 (0.27-2.14)	1.18E-02	1.00E+00
CFAP65	3	4.83 (3.62-6.04)	5.20E-15	2.58E-11	4.07 (2.92-5.23)	4.78E-12	2.46E-08	2.70 (1.31-4.10)	1.44E-04	6.14E-01
CFAP92	5	2.46 (1.55-3.38)	1.36E-07	6.78E-04	2.93 (2.02-3.84)	3.31E-10	1.70E-06	2.66 (1.58-3.75)	1.54E-06	6.61E-03
CHAF1B	2	4.62 (3.15-6.09)	6.73E-10	3.36E-06	4.16 (2.74-5.57)	8.92E-09	4.58E-05	4.09 (1.68-6.50)	8.68E-04	1.00E+00

CHKA	2	2.91 (1.50-4.32)	5.26E-05	2.63E-01	3.25 (1.84-4.65)	5.84E-06	3.00E-02	1.89 (0.35-3.43)	1.63E-02	1.00E+00
CLMN	2	4.52 (3.06-5.98)	1.35E-09	6.77E-06	5.02 (3.57-6.46)	9.92E-12	5.10E-08	3.40 (1.43-5.36)	7.18E-04	1.00E+00
CMTM3	3	2.84 (1.68-3.99)	1.43E-06	7.12E-03	3.42 (2.27-4.57)	5.93E-09	3.04E-05	1.86 (0.60-3.12)	3.79E-03	1.00E+00
CMYA5	6	2.14 (1.33-2.95)	2.25E-07	1.12E-03	2.60 (1.80-3.41)	2.72E-10	1.40E-06	2.41 (1.47-3.36)	5.31E-07	2.27E-03
CNGB1	2	5.48 (3.93-7.04)	5.35E-12	2.67E-08	5.59 (4.11-7.07)	1.34E-13	6.89E-10			
CNTNAP4	2	3.87 (2.44-5.30)	1.10E-07	5.50E-04	4.53 (3.11-5.96)	4.71E-10	2.42E-06	2.99 (1.19-4.79)	1.11E-03	1.00E+00
COL12A1	8	1.66 (0.96-2.37)	3.73E-06	1.86E-02	1.79 (1.09-2.50)	6.11E-07	3.14E-03	1.48 (0.72-2.23)	1.18E-04	5.06E-01
COL14A1	5	2.08 (1.19-2.96)	4.43E-06	2.21E-02	2.35 (1.46-3.24)	1.96E-07	1.01E-03	2.01 (1.02-2.99)	6.57E-05	2.81E-01
COL16A1	6	2.99 (2.17-3.81)	7.63E-13	3.81E-09	2.42 (1.61-3.23)	5.12E-09	2.63E-05	3.00 (1.96-4.04)	1.63E-08	6.97E-05
CORO1A	4	3.38 (2.36-4.40)	9.74E-11	4.87E-07	3.78 (2.76-4.80)	3.68E-13	1.89E-09	2.52 (1.33-3.71)	3.10E-05	1.33E-01
CPA3	3	3.91 (2.74-5.08)	5.57E-11	2.78E-07	4.56 (3.40-5.73)	1.66E-14	8.55E-11	2.88 (1.45-4.32)	8.46E-05	3.62E-01
CPD	3	2.14 (0.98-3.30)	3.09E-04	1.00E+00	2.63 (1.47-3.79)	8.95E-06	4.60E-02	1.47 (0.24-2.70)	1.96E-02	1.00E+00
CPXM1	6	1.55 (0.74-2.37)	1.73E-04	8.63E-01	1.95 (1.14-2.76)	2.42E-06	1.24E-02	1.73 (0.85-2.61)	1.22E-04	5.23E-01
CROCC	10	1.89 (1.27-2.52)	3.29E-09	1.64E-05	2.31 (1.68-2.93)	5.43E-13	2.79E-09	2.23 (1.52-2.95)	9.15E-10	3.91E-06
CRYBG2	7	1.49 (0.74-2.24)	1.06E-04	5.27E-01	1.81 (1.06-2.56)	2.38E-06	1.22E-02	1.61 (0.80-2.42)	9.64E-05	4.12E-01
CRYBG3	9	1.31 (0.65-1.97)	9.91E-05	4.95E-01	1.65 (0.99-2.32)	9.03E-07	4.64E-03	1.64 (0.93-2.36)	6.42E-06	2.75E-02
CSMD2	5	2.32 (1.42-3.21)	3.56E-07	1.78E-03	2.75 (1.86-3.64)	1.40E-09	7.17E-06	2.37 (1.34-3.41)	6.35E-06	2.72E-02
CUBN	5	1.97 (1.08-2.85)	1.37E-05	6.85E-02	2.40 (1.52-3.29)	1.03E-07	5.32E-04	1.60 (0.65-2.55)	9.89E-04	1.00E+00
CYLD	3	4.07 (2.88-5.26)	2.14E-11	1.07E-07	3.67 (2.50-4.84)	7.11E-10	3.65E-06	3.83 (2.02-5.64)	3.22E-05	1.38E-01
CYP26A1	2	2.86 (1.44-4.28)	7.84E-05	3.92E-01	3.54 (2.12-4.96)	9.90E-07	5.09E-03	2.15 (0.56-3.75)	7.92E-03	1.00E+00
DAAM2	6	1.62 (0.81-2.43)	8.93E-05	4.46E-01	2.08 (1.27-2.89)	4.75E-07	2.44E-03	2.01 (1.11-2.91)	1.23E-05	5.25E-02
DBX2	3	2.39 (1.24-3.54)	4.78E-05	2.39E-01	2.97 (1.82-4.12)	3.98E-07	2.05E-03	2.01 (0.74-3.29)	1.99E-03	1.00E+00
DCAF4	3	3.17 (2.02-4.33)	7.05E-08	3.52E-04	2.66 (1.52-3.81)	5.04E-06	2.59E-02	1.93 (0.66-3.19)	2.78E-03	1.00E+00
DCLK2	2	5.08 (3.57-6.59)	3.92E-11	1.96E-07	5.47 (4.00-6.94)	3.11E-13	1.60E-09			
DDN	2	3.45 (2.03-4.87)	1.85E-06	9.25E-03	4.10 (2.68-5.51)	1.40E-08	7.21E-05	4.09 (1.68-6.50)	8.68E-04	1.00E+00
DDX50	2	3.65 (2.23-5.08)	4.96E-07	2.48E-03	4.23 (2.82-5.65)	4.89E-09	2.51E-05	4.09 (1.68-6.50)	8.68E-04	1.00E+00
DDX60L	6	3.10 (2.28-3.91)	1.05E-13	5.26E-10	3.37 (2.56-4.19)	4.00E-16	2.13E-12	2.79 (1.79-3.79)	4.54E-08	1.94E-04
DENND5A	2	6.46 (4.67-8.26)	1.79E-12	8.92E-09	7.26 (5.46-9.06)	2.40E-15	1.24E-11	4.09 (1.68-6.50)	8.68E-04	1.00E+00
DHRS2	2	3.44 (2.02-4.85)	2.05E-06	1.02E-02	3.42 (2.01-4.83)	1.88E-06	9.65E-03	1.78 (0.26-3.31)	2.21E-02	1.00E+00
DHX16	3	2.78 (1.63-3.94)	2.27E-06	1.14E-02	2.61 (1.47-3.76)	8.11E-06	4.16E-02	2.89 (1.45-4.33)	8.46E-05	3.62E-01
DHX37	9	3.66 (2.97-4.35)	0.00E+00	0.00E+00	3.35 (2.67-4.03)	0.00E+00	0.00E+00	3.16 (2.28-4.04)	2.23E-12	9.54E-09

DIDO1	3	3.38 (2.23-4.54)	9.92E-09	4.96E-05	3.76 (2.60-4.91)	1.61E-10	8.29E-07	4.49 (2.23-6.76)	1.02E-04	4.38E-01
DIS3L2	2	3.02 (1.61-4.43)	2.74E-05	1.37E-01	3.61 (2.21-5.02)	4.88E-07	2.51E-03	2.99 (1.19-4.79)	1.11E-03	1.00E+00
DISP2	3	2.50 (1.35-3.65)	2.06E-05	1.03E-01	2.73 (1.59-3.88)	3.06E-06	1.57E-02	2.89 (1.45-4.33)	8.46E-05	3.62E-01
DIXDC1	2	3.98 (2.55-5.41)	5.32E-08	2.66E-04	3.56 (2.15-4.96)	7.33E-07	3.76E-03	2.70 (1.00-4.41)	1.91E-03	1.00E+00
DLL1	2				6.55 (4.94-8.16)	1.50E-15	7.48E-12			
DMXL1	2	5.00 (3.50-6.50)	6.24E-11	3.12E-07	5.79 (4.30-7.29)	3.45E-14	1.77E-10	4.09 (1.68-6.50)	8.68E-04	1.00E+00
DNA2	2	5.77 (4.16-7.38)	2.07E-12	1.04E-08	5.88 (4.37-7.38)	2.11E-14	1.08E-10	4.09 (1.68-6.50)	8.68E-04	1.00E+00
DNAH14	7	2.87 (2.11-3.62)	9.05E-14	4.52E-10	3.25 (2.50-4.00)	0.00E+00	1.25E-13	2.25 (1.40-3.11)	2.55E-07	1.09E-03
DNAH17	12	3.05 (2.47-3.63)	0.00E+00	0.00E+00	2.48 (1.91-3.06)	0.00E+00	1.35E-13	1.56 (0.94-2.17)	7.37E-07	3.15E-03
DNAH5	3	2.38 (1.23-3.53)	4.70E-05	2.35E-01	2.80 (1.65-3.94)	1.68E-06	8.62E-03	1.55 (0.32-2.77)	1.34E-02	1.00E+00
DNAH6	5	1.57 (0.68-2.46)	5.16E-04	1.00E+00	2.03 (1.14-2.91)	7.49E-06	3.85E-02	2.23 (1.22-3.25)	1.49E-05	6.38E-02
DNAH8	5	2.06 (1.18-2.95)	5.09E-06	2.54E-02	2.21 (1.33-3.10)	9.40E-07	4.83E-03	1.36 (0.43-2.30)	4.39E-03	1.00E+00
DNAH9	2	4.13 (2.69-5.57)	1.91E-08	9.55E-05	4.32 (2.90-5.74)	2.57E-09	1.32E-05			
DNAI1	2	3.56 (2.14-4.98)	9.34E-07	4.67E-03	4.00 (2.59-5.42)	2.84E-08	1.46E-04	3.40 (1.43-5.36)	7.18E-04	1.00E+00
DNAJB8	6	6.25 (5.23-7.28)	0.00E+00	0.00E+00	6.01 (5.11-6.91)	0.00E+00	0.00E+00			
DNAJC1	2	6.18 (4.47-7.88)	1.27E-12	6.36E-09	3.73 (2.32-5.13)	2.21E-07	1.14E-03			
DNAJC22	2	2.83 (1.41-4.25)	9.10E-05	4.54E-01	3.46 (2.04-4.87)	1.75E-06	8.99E-03	3.00 (1.20-4.81)	1.11E-03	1.00E+00
DNASE1	3	4.64 (3.44-5.84)	3.41E-14	1.70E-10	2.98 (1.84-4.13)	3.33E-07	1.71E-03	3.80 (2.01-5.60)	3.30E-05	1.41E-01
DOCK7	2	4.39 (2.93-5.84)	3.27E-09	1.63E-05	4.41 (2.99-5.83)	1.24E-09	6.40E-06	4.09 (1.68-6.50)	8.68E-04	1.00E+00
DOK2	2	2.97 (1.56-4.38)	3.70E-05	1.85E-01	3.62 (2.22-5.03)	4.54E-07	2.33E-03	1.60 (0.09-3.11)	3.72E-02	1.00E+00
DOK7	2	4.20 (2.75-5.64)	1.20E-08	5.99E-05	4.49 (3.06-5.91)	6.54E-10	3.36E-06	3.40 (1.43-5.36)	7.18E-04	1.00E+00
DOP1B	8	1.53 (0.83-2.23)	1.85E-05	9.23E-02	1.86 (1.16-2.57)	1.85E-07	9.49E-04	1.76 (1.00-2.53)	6.07E-06	2.59E-02
DOT1L	2	2.76 (1.35-4.17)	1.22E-04	6.09E-01	3.27 (1.86-4.67)	5.15E-06	2.65E-02	1.78 (0.26-3.31)	2.21E-02	1.00E+00
DPYSL4	2	5.48 (3.93-7.04)	5.35E-12	2.67E-08	6.06 (4.53-7.59)	7.70E-15	3.94E-11	3.40 (1.43-5.36)	7.18E-04	1.00E+00
DSCAML1	3	3.40 (2.25-4.56)	8.17E-09	4.08E-05	2.94 (1.79-4.08)	4.85E-07	2.49E-03	1.93 (0.66-3.19)	2.78E-03	1.00E+00
DST	5	2.32 (1.43-3.21)	3.06E-07	1.53E-03	2.30 (1.42-3.19)	3.33E-07	1.71E-03	1.96 (0.98-2.94)	9.13E-05	3.91E-01
DYNC2H1	4	2.47 (1.47-3.46)	1.19E-06	5.94E-03	3.02 (2.03-4.01)	2.62E-09	1.35E-05	1.79 (0.71-2.87)	1.21E-03	1.00E+00
DYNLT2B	7	3.56 (2.79-4.33)	0.00E+00	5.00E-16	3.10 (2.34-3.86)	1.10E-15	5.49E-12	1.96 (1.12-2.79)	4.39E-06	1.88E-02
EEF1AKMT4-	3	2.69 (1.53-3.86)	5.98E-06	2.99E-02	3.24 (2.07-4.40)	5.03E-08	2.59E-04	3.43 (1.81-5.04)	3.39E-05	1.45E-01
EGF	3	3.68 (2.52-4.84)	5.64E-10	2.82E-06	4.24 (3.09-5.40)	6.84E-13	3.51E-09	2.30 (0.98-3.61)	6.16E-04	1.00E+00
EHBP1L1	4	3.18 (2.18-4.19)	5.68E-10	2.84E-06	2.87 (1.87-3.87)	1.74E-08	8.92E-05	3.19 (1.86-4.51)	2.48E-06	1.06E-02

ENPP6	2	6.87 (4.90-8.84)	7.63E-12	3.81E-08	6.42 (4.83-8.00)	1.80E-15	9.19E-12			
ENTPD7	3	3.39 (2.23-4.55)	1.05E-08	5.23E-05	3.45 (2.30-4.60)	4.43E-09	2.28E-05	2.89 (1.45-4.33)	8.46E-05	3.62E-01
EPHA10	5	2.24 (1.36-3.13)	7.30E-07	3.65E-03	2.38 (1.49-3.26)	1.45E-07	7.43E-04	2.11 (1.12-3.11)	3.24E-05	1.39E-01
EPHB4	2	2.99 (1.57-4.41)	3.64E-05	1.82E-01	3.48 (2.06-4.89)	1.50E-06	7.73E-03	2.02 (0.45-3.59)	1.16E-02	1.00E+00
ERV3-1	2	3.98 (2.55-5.41)	5.33E-08	2.66E-04	4.27 (2.85-5.68)	3.81E-09	1.96E-05			
ESAM	2	3.97 (2.52-5.41)	7.13E-08	3.56E-04	3.51 (2.10-4.93)	1.17E-06	6.01E-03	2.15 (0.56-3.75)	7.92E-03	1.00E+00
ESF1	2	5.00 (3.50-6.50)	6.19E-11	3.09E-07	5.72 (4.23-7.21)	5.47E-14	2.81E-10	3.40 (1.43-5.36)	7.18E-04	1.00E+00
ETV5	2	3.98 (2.55-5.42)	5.37E-08	2.68E-04	4.37 (2.95-5.79)	1.76E-09	9.06E-06	4.09 (1.68-6.50)	8.68E-04	1.00E+00
EXOC3L2	2	4.07 (2.63-5.50)	2.92E-08	1.46E-04	4.75 (3.32-6.18)	8.22E-11	4.22E-07	2.99 (1.19-4.79)	1.11E-03	1.00E+00
EXOSC3	3	4.67 (3.47-5.87)	2.38E-14	1.19E-10	3.62 (2.47-4.77)	6.46E-10	3.32E-06			
F10	2	3.45 (2.03-4.87)	1.95E-06	9.75E-03	3.35 (1.95-4.76)	3.01E-06	1.55E-02	3.40 (1.43-5.36)	7.18E-04	1.00E+00
F2	2	6.87 (4.90-8.84)	7.65E-12	3.82E-08	6.57 (4.96-8.18)	1.20E-15	6.40E-12	4.09 (1.68-6.50)	8.68E-04	1.00E+00
FAM135A	3	4.04 (2.87-5.22)	1.63E-11	8.14E-08	4.50 (3.33-5.66)	4.36E-14	2.24E-10	2.01 (0.74-3.29)	1.99E-03	1.00E+00
FAM53A	4	3.21 (2.21-4.21)	3.24E-10	1.62E-06	2.98 (1.99-3.97)	3.85E-09	1.98E-05	1.84 (0.75-2.92)	9.14E-04	1.00E+00
FAM71B	6	1.65 (0.83-2.47)	7.90E-05	3.95E-01	2.02 (1.20-2.83)	1.45E-06	7.45E-03	1.40 (0.53-2.27)	1.60E-03	1.00E+00
FAM81A	2	6.87 (4.90-8.84)	7.64E-12	3.82E-08	7.26 (5.46-9.06)	2.40E-15	1.23E-11			
FAT2	9	1.64 (0.97-2.30)	1.29E-06	6.44E-03	1.99 (1.33-2.65)	3.85E-09	1.98E-05	1.61 (0.89-2.32)	9.84E-06	4.21E-02
FBLN2	4	3.98 (2.97-5.00)	1.53E-14	7.66E-11	4.31 (3.31-5.32)	0.00E+00	2.28E-13	3.40 (2.01-4.79)	1.71E-06	7.33E-03
FBN1	5	5.18 (4.22-6.14)	0.00E+00	0.00E+00	4.08 (3.19-4.98)	0.00E+00	1.90E-15	3.62 (2.30-4.94)	7.60E-08	3.25E-04
FBP1	3	4.02 (2.85-5.19)	1.82E-11	9.09E-08	4.72 (3.55-5.89)	2.40E-15	1.24E-11	3.80 (2.01-5.60)	3.30E-05	1.41E-01
FBRS	3	3.04 (1.89-4.19)	2.33E-07	1.17E-03	3.11 (1.96-4.26)	1.06E-07	5.42E-04	3.11 (1.60-4.61)	5.09E-05	2.18E-01
FBXO40	2	5.62 (4.04-7.20)	3.25E-12	1.62E-08	5.18 (3.73-6.64)	2.67E-12	1.37E-08	2.70 (1.00-4.41)	1.91E-03	1.00E+00
FGD5	2	7.56 (5.16-9.97)	7.26E-10	3.63E-06	8.36 (5.95-10.77)	9.81E-12	5.04E-08			
FKTN	5	1.96 (1.05-2.87)	2.58E-05	1.29E-01	2.24 (1.33-3.15)	1.50E-06	7.71E-03	2.29 (1.25-3.32)	1.49E-05	6.38E-02
FLG	11	1.31 (0.71-1.91)	1.70E-05	8.49E-02	1.68 (1.09-2.28)	3.18E-08	1.64E-04	1.09 (0.47-1.71)	6.28E-04	1.00E+00
FLG2	3	2.67 (1.51-3.82)	5.79E-06	2.89E-02	3.04 (1.89-4.19)	2.23E-07	1.15E-03	2.55 (1.19-3.92)	2.40E-04	1.00E+00
FNDC1	7	1.67 (0.91-2.42)	1.56E-05	7.78E-02	2.11 (1.36-2.87)	4.19E-08	2.15E-04	1.67 (0.85-2.48)	6.18E-05	2.64E-01
FNDC7	9	1.94 (1.27-2.62)	1.97E-08	9.83E-05	2.32 (1.64-3.00)	1.95E-11	1.00E-07	1.66 (0.94-2.39)	7.60E-06	3.25E-02
FOXK2	4	2.05 (1.05-3.04)	5.45E-05	2.72E-01	2.36 (1.36-3.35)	3.23E-06	1.66E-02	1.53 (0.46-2.59)	4.89E-03	1.00E+00
FREM1	3	2.96 (1.81-4.11)	4.75E-07	2.37E-03	3.41 (2.26-4.56)	5.86E-09	3.01E-05			
FREM2	6	2.51 (1.70-3.33)	1.33E-09	6.63E-06	2.86 (2.05-3.67)	4.43E-12	2.27E-08	2.55 (1.59-3.51)	2.07E-07	8.83E-04

FREM3	3	2.14 (0.99-3.28)	2.51E-04	1.00E+00	2.58 (1.44-3.72)	9.69E-06	4.98E-02	1.40 (0.19-2.61)	2.38E-02	1.00E+00
FSIP2	10	1.48 (0.85-2.10)	3.96E-06	1.98E-02	1.74 (1.11-2.37)	5.16E-08	2.65E-04	1.44 (0.77-2.10)	2.51E-05	1.07E-01
GAK	2	5.08 (3.57-6.59)	3.90E-11	1.95E-07	5.32 (3.86-6.78)	9.61E-13	4.94E-09	2.14 (0.56-3.72)	7.92E-03	1.00E+00
GARRE1	2	7.56 (5.15-9.97)	7.34E-10	3.67E-06	8.36 (5.95-10.76)	9.92E-12	5.10E-08	3.40 (1.43-5.36)	7.18E-04	1.00E+00
GJB6	4	2.04 (1.04-3.04)	6.13E-05	3.06E-01	2.38 (1.38-3.38)	2.91E-06	1.50E-02	1.61 (0.54-2.69)	3.20E-03	1.00E+00
GLCCI1	2	2.96 (1.55-4.37)	3.99E-05	2.00E-01	3.31 (1.91-4.72)	3.79E-06	1.94E-02	2.14 (0.56-3.72)	7.92E-03	1.00E+00
GNAT2	4	2.14 (1.14-3.14)	2.75E-05	1.37E-01	2.62 (1.62-3.62)	2.71E-07	1.39E-03	1.66 (0.58-2.73)	2.55E-03	1.00E+00
GOLGA6L2	2	2.98 (1.56-4.41)	3.97E-05	1.98E-01	3.37 (1.95-4.78)	3.22E-06	1.65E-02			
GOLPH3L	2	3.04 (1.63-4.45)	2.39E-05	1.20E-01	3.61 (2.20-5.02)	5.14E-07	2.64E-03	1.52 (0.02-3.02)	4.67E-02	1.00E+00
GPRIN3	2	5.77 (4.16-7.38)	2.07E-12	1.03E-08	5.96 (4.45-7.48)	1.28E-14	6.56E-11			
GRIK1	2	2.70 (1.30-4.11)	1.66E-04	8.30E-01	3.28 (1.88-4.69)	4.58E-06	2.36E-02	2.30 (0.69-3.91)	5.17E-03	1.00E+00
GRK4	2	3.93 (2.49-5.36)	7.71E-08	3.85E-04	4.28 (2.86-5.70)	3.32E-09	1.71E-05			
GSX2	2	7.56 (5.16-9.97)	7.29E-10	3.64E-06	8.36 (5.95-10.77)	9.84E-12	5.06E-08	3.40 (1.43-5.36)	7.18E-04	1.00E+00
GTPBP6	2	3.61 (2.18-5.04)	7.93E-07	3.96E-03	4.33 (2.90-5.76)	2.91E-09	1.50E-05	3.00 (1.20-4.81)	1.11E-03	1.00E+00
H1-2	2	2.67 (1.26-4.07)	2.04E-04	1.00E+00	3.27 (1.87-4.68)	4.95E-06	2.54E-02	2.99 (1.19-4.79)	1.11E-03	1.00E+00
HEATR1	7	2.18 (1.43-2.93)	1.33E-08	6.66E-05	2.24 (1.49-2.99)	4.57E-09	2.35E-05	1.79 (0.97-2.61)	1.85E-05	7.89E-02
HECTD4	4	3.14 (2.15-4.14)	6.83E-10	3.41E-06	3.23 (2.24-4.22)	1.81E-10	9.28E-07	2.99 (1.72-4.26)	4.01E-06	1.72E-02
HELZ2	3	2.61 (1.46-3.76)	8.52E-06	4.26E-02	3.08 (1.93-4.22)	1.42E-07	7.32E-04	2.41 (1.08-3.75)	3.91E-04	1.00E+00
HERC1	3	2.57 (1.43-3.72)	1.10E-05	5.48E-02	3.01 (1.87-4.16)	2.53E-07	1.30E-03	3.11 (1.60-4.61)	5.09E-05	2.18E-01
HERC2	3	2.39 (1.24-3.53)	4.44E-05	2.22E-01	2.80 (1.65-3.94)	1.63E-06	8.37E-03	2.10 (0.81-3.38)	1.39E-03	1.00E+00
HHIPL1	2	3.63 (2.21-5.05)	5.69E-07	2.84E-03	3.87 (2.46-5.28)	7.62E-08	3.91E-04	3.40 (1.43-5.36)	7.18E-04	1.00E+00
HIVEP2	2	3.73 (2.29-5.16)	3.58E-07	1.79E-03	4.33 (2.90-5.76)	2.92E-09	1.50E-05			
HIVEP3	3	5.33 (4.08-6.58)	1.00E-16	4.15E-13	5.63 (4.42-6.84)	0.00E+00	4.00E-16	4.49 (2.23-6.76)	1.02E-04	4.38E-01
HK3	8	1.52 (0.82-2.23)	2.27E-05	1.13E-01	1.65 (0.95-2.36)	4.19E-06	2.15E-02	1.82 (1.05-2.59)	3.57E-06	1.53E-02
HLA-DQA2	2	5.24 (3.70-6.78)	2.37E-11	1.19E-07	4.94 (3.49-6.39)	2.60E-11	1.33E-07	0.79 (-0.66-2.25)	2.85E-01	1.00E+00
HLA-DRB1	2	6.44 (4.46-8.41)	1.67E-10	8.37E-07	5.90 (4.31-7.48)	3.67E-13	1.89E-09	3.00 (1.20-4.81)	1.11E-03	1.00E+00
HMCN2	8	2.04 (1.34-2.74)	1.10E-08	5.50E-05	2.53 (1.83-3.23)	1.32E-12	6.76E-09	2.38 (1.57-3.20)	9.46E-09	4.05E-05
HOXC6	7	5.36 (4.53-6.19)	0.00E+00	0.00E+00	5.92 (5.11-6.74)	0.00E+00	0.00E+00	5.35 (3.25-7.45)	5.76E-07	2.46E-03
HOXD13	2	3.71 (2.29-5.14)	3.27E-07	1.64E-03	3.49 (2.08-4.89)	1.19E-06	6.13E-03	2.14 (0.56-3.72)	7.92E-03	1.00E+00
IFNW1	5	2.51 (1.61-3.40)	4.60E-08	2.30E-04	2.15 (1.26-3.05)	2.42E-06	1.24E-02	1.93 (0.95-2.92)	1.24E-04	5.31E-01
IGDCC3	2	5.95 (4.31-7.60)	1.45E-12	7.23E-09	6.16 (4.62-7.71)	4.60E-15	2.37E-11			

IGDCC4	7	1.59 (0.84-2.34)	3.45E-05	1.72E-01	2.02 (1.27-2.77)	1.45E-07	7.44E-04	1.46 (0.65-2.26)	3.71E-04	1.00E+00
IGSF11	2	3.34 (1.92-4.77)	4.34E-06	2.17E-02	3.86 (2.44-5.29)	9.96E-08	5.11E-04	3.00 (1.20-4.81)	1.11E-03	1.00E+00
IGSF9B	3	2.67 (1.52-3.82)	5.20E-06	2.60E-02	2.99 (1.85-4.14)	2.99E-07	1.53E-03	2.10 (0.81-3.38)	1.39E-03	1.00E+00
IL17RA	3	2.92 (1.77-4.08)	6.31E-07	3.15E-03	3.31 (2.16-4.45)	1.61E-08	8.27E-05	2.55 (1.19-3.91)	2.40E-04	1.00E+00
IL24	2				8.36 (5.95-10.77)	9.79E-12	5.03E-08			
INSRR	6	2.75 (1.94-3.57)	3.10E-11	1.55E-07	3.23 (2.42-4.04)	5.70E-15	2.93E-11	2.35 (1.42-3.29)	8.31E-07	3.56E-03
IPO5	2	3.56 (2.14-4.98)	9.34E-07	4.67E-03	4.04 (2.63-5.46)	2.11E-08	1.08E-04	2.99 (1.19-4.79)	1.11E-03	1.00E+00
IQCE	3	2.55 (1.41-3.70)	1.29E-05	6.47E-02	2.77 (1.62-3.91)	2.15E-06	1.10E-02	1.85 (0.60-3.11)	3.79E-03	1.00E+00
IRS1	2	5.62 (4.04-7.20)	3.26E-12	1.63E-08	5.47 (4.00-6.94)	3.10E-13	1.59E-09			
ITGA7	2	3.04 (1.63-4.45)	2.40E-05	1.20E-01	3.61 (2.20-5.01)	5.16E-07	2.65E-03	3.40 (1.43-5.36)	7.18E-04	1.00E+00
ITGAD	3	2.96 (1.79-4.13)	6.81E-07	3.40E-03	3.51 (2.35-4.68)	3.55E-09	1.83E-05			
ITGB4	3	3.06 (1.90-4.21)	2.02E-07	1.01E-03	2.69 (1.55-3.84)	3.90E-06	2.01E-02	2.30 (0.98-3.61)	6.16E-04	1.00E+00
ITPRID2	4	3.73 (2.71-4.74)	6.09E-13	3.04E-09	3.65 (2.64-4.65)	1.03E-12	5.27E-09	2.85 (1.61-4.09)	6.72E-06	2.88E-02
IVD	2	2.53 (1.13-3.94)	4.12E-04	1.00E+00	3.24 (1.83-4.64)	6.27E-06	3.22E-02	1.25 (-0.22-2.73)	9.63E-02	1.00E+00
JPH2	2	3.38 (1.95-4.81)	3.51E-06	1.75E-02	3.31 (1.90-4.73)	4.53E-06	2.33E-02	1.46 (-0.04-2.96)	5.70E-02	1.00E+00
KATNAL2	2	5.62 (4.04-7.20)	3.25E-12	1.62E-08	6.16 (4.62-7.71)	4.60E-15	2.37E-11	2.70 (1.00-4.41)	1.91E-03	1.00E+00
KATNIP	2	5.62 (4.04-7.20)	3.25E-12	1.62E-08	6.41 (4.83-8.00)	1.80E-15	9.24E-12	3.40 (1.43-5.36)	7.18E-04	1.00E+00
KCNA7	2	6.17 (4.47-7.88)	1.31E-12	6.53E-09	6.97 (5.26-8.68)	1.20E-15	5.98E-12	4.09 (1.68-6.50)	8.68E-04	1.00E+00
KCNJ14	3	4.39 (3.19-5.59)	8.16E-13	4.07E-09	3.37 (2.21-4.53)	1.42E-08	7.30E-05	2.44 (1.09-3.79)	3.92E-04	1.00E+00
KDM5A	4	3.29 (2.29-4.29)	1.12E-10	5.59E-07	3.56 (2.57-4.56)	2.25E-12	1.15E-08	3.17 (1.85-4.49)	2.49E-06	1.06E-02
KHDC3L	2	3.77 (2.33-5.21)	2.68E-07	1.34E-03	4.33 (2.90-5.76)	2.91E-09	1.50E-05	1.61 (0.10-3.13)	3.71E-02	1.00E+00
KIAA1614	2	4.10 (2.66-5.54)	2.36E-08	1.18E-04	4.70 (3.27-6.13)	1.25E-10	6.41E-07			
KIAA2026	4	1.84 (0.85-2.83)	2.72E-04	1.00E+00	2.37 (1.38-3.36)	2.58E-06	1.32E-02	1.95 (0.85-3.04)	4.97E-04	1.00E+00
KIDINS220	2	3.00 (1.59-4.41)	3.08E-05	1.54E-01	3.32 (1.92-4.73)	3.56E-06	1.83E-02			
KIF17	2	3.02 (1.61-4.43)	2.72E-05	1.36E-01	3.49 (2.09-4.90)	1.15E-06	5.89E-03	2.99 (1.19-4.79)	1.11E-03	1.00E+00
KIF27	2	2.62 (1.20-4.04)	2.87E-04	1.00E+00	3.24 (1.83-4.66)	6.99E-06	3.59E-02	2.72 (1.00-4.43)	1.91E-03	1.00E+00
KMT2C	7	1.83 (1.07-2.58)	1.89E-06	9.45E-03	2.19 (1.44-2.94)	1.10E-08	5.67E-05	1.88 (1.05-2.70)	8.05E-06	3.44E-02
KRT2	3	2.29 (1.14-3.44)	9.68E-05	4.84E-01	2.68 (1.53-3.82)	4.91E-06	2.52E-02	2.42 (1.08-3.76)	3.91E-04	1.00E+00
KRT35	2	2.77 (1.36-4.17)	1.17E-04	5.84E-01	3.31 (1.90-4.72)	3.89E-06	2.00E-02	2.01 (0.45-3.57)	1.16E-02	1.00E+00
KRTAP5-7	2	6.00 (4.29-7.70)	5.51E-12	2.75E-08	6.26 (4.68-7.84)	8.40E-15	4.33E-11			
LAMA3	4				6.66 (5.51-7.81)	0.00E+00	0.00E+00			

LAMA5	11	1.30 (0.71-1.90)	1.87E-05	9.35E-02	1.56 (0.97-2.16)	2.78E-07	1.43E-03	1.06 (0.44-1.69)	8.40E-04	1.00E+00
LAMC2	7	2.32 (1.56-3.07)	1.53E-09	7.63E-06	2.66 (1.91-3.41)	3.59E-12	1.84E-08	2.51 (1.62-3.40)	2.80E-08	1.20E-04
LARP4B	2	4.62 (3.15-6.09)	6.73E-10	3.36E-06	5.23 (3.77-6.68)	1.92E-12	9.89E-09	4.09 (1.68-6.50)	8.68E-04	1.00E+00
LHCGR	3	2.82 (1.67-3.98)	1.64E-06	8.21E-03	3.24 (2.09-4.39)	3.33E-08	1.71E-04	3.81 (2.01-5.61)	3.28E-05	1.40E-01
LHX2	3	5.36 (4.09-6.64)	2.00E-16	1.00E-12	4.55 (3.37-5.74)	4.65E-14	2.39E-10	4.52 (2.25-6.80)	9.81E-05	4.19E-01
LIMCH1	3	4.18 (3.01-5.36)	3.31E-12	1.65E-08	3.25 (2.10-4.39)	2.87E-08	1.47E-04			
LRBA	3	2.49 (1.33-3.65)	2.76E-05	1.38E-01	3.07 (1.91-4.24)	2.20E-07	1.13E-03	2.12 (0.82-3.42)	1.38E-03	1.00E+00
LRP2	9	1.49 (0.83-2.15)	9.21E-06	4.60E-02	1.56 (0.90-2.22)	3.52E-06	1.81E-02	1.18 (0.49-1.88)	8.25E-04	1.00E+00
LRRC30	2	5.38 (3.83-6.93)	1.05E-11	5.23E-08	3.25 (1.84-4.67)	6.69E-06	3.44E-02	2.02 (0.45-3.59)	1.16E-02	1.00E+00
LRRC41	3	5.14 (3.90-6.37)	4.00E-16	1.81E-12	5.88 (4.65-7.11)	0.00E+00	0.00E+00	3.40 (1.79-5.00)	3.43E-05	1.47E-01
LRRC9	2	3.00 (1.59-4.41)	3.09E-05	1.54E-01	3.66 (2.25-5.07)	3.55E-07	1.82E-03	2.70 (1.00-4.41)	1.91E-03	1.00E+00
LRRIQ1	6	2.10 (1.29-2.92)	4.44E-07	2.22E-03	2.07 (1.26-2.89)	5.81E-07	2.99E-03	2.11 (1.19-3.02)	6.12E-06	2.62E-02
LRRN4	2	5.00 (3.50-6.50)	6.19E-11	3.09E-07	5.59 (4.11-7.07)	1.34E-13	6.88E-10	4.09 (1.68-6.50)	8.68E-04	1.00E+00
LUC7L	2	6.47 (4.67-8.26)	1.78E-12	8.88E-09	6.97 (5.27-8.68)	1.10E-15	5.72E-12	4.09 (1.68-6.50)	8.68E-04	1.00E+00
MACF1	7	1.95 (1.20-2.70)	3.51E-07	1.75E-03	2.09 (1.34-2.83)	4.59E-08	2.36E-04	1.97 (1.14-2.80)	3.23E-06	1.38E-02
MANSC1	2	3.06 (1.65-4.47)	2.10E-05	1.05E-01	3.75 (2.35-5.16)	1.79E-07	9.19E-04	2.30 (0.69-3.91)	5.17E-03	1.00E+00
MAP3K13	2	4.73 (3.26-6.21)	3.25E-10	1.62E-06	4.78 (3.34-6.21)	6.58E-11	3.38E-07			
MAPK3	4	3.40 (2.38-4.43)	7.43E-11	3.71E-07	3.80 (2.78-4.81)	2.98E-13	1.53E-09	2.52 (1.33-3.71)	3.10E-05	1.33E-01
MAPK8IP2	2	2.76 (1.35-4.17)	1.22E-04	6.12E-01	3.35 (1.94-4.75)	3.00E-06	1.54E-02	2.99 (1.19-4.79)	1.11E-03	1.00E+00
MAPKAPK3	4	6.39 (5.12-7.67)	0.00E+00	0.00E+00	5.88 (4.80-6.96)	0.00E+00	0.00E+00			
MAST4	3	6.58 (5.08-8.09)	0.00E+00	4.63E-14	5.33 (4.14-6.53)	0.00E+00	1.01E-14	3.80 (2.01-5.60)	3.30E-05	1.41E-01
MCM3AP	7	1.96 (1.21-2.71)	3.20E-07	1.60E-03	1.87 (1.12-2.62)	1.06E-06	5.46E-03	1.17 (0.38-1.95)	3.66E-03	1.00E+00
MCM5	2	5.77 (4.16-7.38)	2.06E-12	1.03E-08	5.88 (4.37-7.38)	2.09E-14	1.07E-10	4.09 (1.68-6.50)	8.68E-04	1.00E+00
MCUR1	2	3.78 (2.35-5.21)	2.12E-07	1.06E-03	3.70 (2.30-5.11)	2.57E-07	1.32E-03	3.40 (1.43-5.36)	7.18E-04	1.00E+00
MEGF6	8	1.40 (0.69-2.10)	9.59E-05	4.79E-01	1.83 (1.13-2.53)	3.02E-07	1.55E-03	1.54 (0.79-2.30)	5.68E-05	2.43E-01
MEI1	3	4.51 (3.32-5.71)	1.42E-13	7.07E-10	5.11 (3.92-6.30)	0.00E+00	1.61E-13	3.81 (2.01-5.61)	3.28E-05	1.40E-01
MEX3B	2	3.26 (1.84-4.67)	6.31E-06	3.15E-02	3.79 (2.38-5.20)	1.42E-07	7.32E-04			
MFHAS1	2	5.37 (3.83-6.91)	8.89E-12	4.44E-08	4.04 (2.63-5.46)	2.11E-08	1.08E-04	2.48 (0.83-4.13)	3.22E-03	1.00E+00
MGAM	3	3.11 (1.96-4.27)	1.24E-07	6.19E-04	3.75 (2.60-4.90)	1.78E-10	9.16E-07			
MLIP	2	6.46 (4.67-8.26)	1.78E-12	8.90E-09	6.97 (5.27-8.68)	1.10E-15	5.75E-12			
MOCOS	2	3.09 (1.67-4.50)	1.83E-05	9.16E-02	3.47 (2.06-4.88)	1.33E-06	6.82E-03	2.70 (1.00-4.41)	1.91E-03	1.00E+00

MOV10L1	2	3.05 (1.64-4.46)	2.24E-05	1.12E-01	3.59 (2.18-5.00)	5.81E-07	2.98E-03	1.60 (0.09-3.11)	3.72E-02	1.00E+00
MPO	2	3.03 (1.62-4.44)	2.56E-05	1.28E-01	3.51 (2.10-4.92)	1.02E-06	5.25E-03	1.45 (-0.04-2.94)	5.73E-02	1.00E+00
MRTFA	3	4.33 (3.15-5.51)	7.49E-13	3.74E-09	4.85 (3.68-6.02)	5.00E-16	2.72E-12	3.80 (2.01-5.60)	3.30E-05	1.41E-01
MSS51	3	2.54 (1.38-3.71)	1.90E-05	9.49E-02	2.88 (1.72-4.05)	1.15E-06	5.90E-03	1.38 (0.15-2.61)	2.79E-02	1.00E+00
MTUS1	12	1.15 (0.58-1.72)	7.72E-05	3.86E-01	1.54 (0.97-2.11)	1.21E-07	6.22E-04	1.17 (0.57-1.77)	1.36E-04	5.82E-01
MUC16	20	1.55 (1.11-2.00)	6.59E-12	3.29E-08	1.39 (0.95-1.84)	6.83E-10	3.51E-06	1.17 (0.70-1.63)	8.43E-07	3.61E-03
MUC4	11	2.67 (2.07-3.28)	0.00E+00	1.47E-14	2.23 (1.63-2.82)	2.89E-13	1.49E-09	2.80 (2.06-3.54)	1.21E-13	5.18E-10
MUC5B	9	1.79 (1.13-2.45)	1.18E-07	5.90E-04	2.12 (1.45-2.78)	3.77E-10	1.94E-06	1.91 (1.18-2.64)	2.97E-07	1.27E-03
MVP	2	6.47 (4.67-8.26)	1.78E-12	8.87E-09	5.03 (3.58-6.47)	8.88E-12	4.56E-08			
MYCBP2	2				6.16 (4.62-7.70)	4.80E-15	2.45E-11			
MYH3	5	1.57 (0.68-2.46)	5.29E-04	1.00E+00	2.09 (1.20-2.98)	4.04E-06	2.08E-02	1.27 (0.33-2.21)	7.91E-03	1.00E+00
MYO15A	5	2.40 (1.51-3.29)	1.17E-07	5.85E-04	2.68 (1.80-3.57)	2.92E-09	1.50E-05	1.60 (0.65-2.55)	9.89E-04	1.00E+00
MYO15B	9	1.54 (0.88-2.20)	4.71E-06	2.35E-02	1.95 (1.29-2.61)	7.52E-09	3.86E-05	1.86 (1.13-2.58)	5.32E-07	2.28E-03
MYO18B	2	7.56 (5.16-9.97)	7.26E-10	3.63E-06	6.75 (5.10-8.40)	1.00E-15	5.16E-12			
MYO5B	2	3.01 (1.60-4.42)	2.90E-05	1.45E-01	3.33 (1.92-4.74)	3.40E-06	1.75E-02	1.38 (-0.11-2.86)	6.91E-02	1.00E+00
MYO7A	6	2.05 (1.24-2.87)	7.29E-07	3.64E-03	2.45 (1.64-3.26)	3.28E-09	1.68E-05	1.53 (0.66-2.40)	5.66E-04	1.00E+00
N4BP2	2	3.20 (1.77-4.62)	1.09E-05	5.42E-02	3.76 (2.34-5.18)	2.11E-07	1.08E-03	1.70 (0.17-3.23)	2.90E-02	1.00E+00
NACA	3	2.88 (1.73-4.03)	9.30E-07	4.65E-03	3.39 (2.25-4.54)	6.89E-09	3.54E-05	1.93 (0.66-3.19)	2.78E-03	1.00E+00
NAP1L3	6	3.53 (2.66-4.39)	1.40E-15	7.07E-12	3.38 (2.53-4.23)	6.40E-15	3.27E-11	4.17 (2.76-5.57)	6.82E-09	2.92E-05
NAV2	5	3.25 (2.36-4.15)	1.01E-12	5.06E-09	3.51 (2.62-4.40)	1.03E-14	5.31E-11	3.40 (2.15-4.64)	8.87E-08	3.79E-04
NCF4	2	3.39 (1.97-4.81)	2.77E-06	1.39E-02	3.47 (2.06-4.88)	1.33E-06	6.83E-03	2.99 (1.19-4.79)	1.11E-03	1.00E+00
NCOA3	2	5.15 (3.63-6.67)	2.79E-11	1.39E-07	4.17 (2.75-5.59)	8.00E-09	4.11E-05	2.99 (1.19-4.79)	1.11E-03	1.00E+00
NCOR2	3	6.36 (4.92-7.80)	0.00E+00	2.20E-14	6.46 (5.17-7.76)	0.00E+00	0.00E+00	3.80 (2.01-5.60)	3.30E-05	1.41E-01
NEB	8	2.26 (1.55-2.96)	2.90E-10	1.45E-06	2.48 (1.78-3.18)	4.04E-12	2.07E-08	2.04 (1.26-2.82)	3.18E-07	1.36E-03
NEIL2	2	4.01 (2.57-5.44)	4.38E-08	2.19E-04	4.11 (2.70-5.53)	1.25E-08	6.40E-05	3.40 (1.43-5.36)	7.18E-04	1.00E+00
NIBAN3	2	4.07 (2.63-5.50)	2.92E-08	1.46E-04	4.67 (3.24-6.10)	1.52E-10	7.83E-07	2.99 (1.19-4.79)	1.11E-03	1.00E+00
NIN	6	2.16 (1.35-2.97)	1.65E-07	8.26E-04	2.02 (1.21-2.83)	9.17E-07	4.71E-03	2.05 (1.15-2.95)	8.75E-06	3.74E-02
NINL	3	2.91 (1.76-4.06)	7.02E-07	3.51E-03	3.32 (2.17-4.47)	1.42E-08	7.29E-05	1.66 (0.42-2.89)	8.50E-03	1.00E+00
NLRP5	3	3.64 (2.48-4.80)	8.43E-10	4.21E-06	4.15 (2.99-5.31)	2.01E-12	1.03E-08	2.70 (1.31-4.10)	1.44E-04	6.14E-01
NME8	2	2.84 (1.43-4.24)	7.92E-05	3.96E-01	3.44 (2.03-4.85)	1.62E-06	8.31E-03	1.60 (0.09-3.11)	3.72E-02	1.00E+00
NMUR2	2	2.71 (1.30-4.12)	1.60E-04	7.97E-01	3.25 (1.84-4.65)	5.85E-06	3.01E-02	1.52 (0.02-3.02)	4.67E-02	1.00E+00

NOC4L	2	4.27 (2.82-5.71)	7.30E-09	3.65E-05	3.41 (2.01-4.82)	1.97E-06	1.01E-02	2.48 (0.83-4.13)	3.22E-03	1.00E+00
NOL8	3	4.14 (2.96-5.32)	5.20E-12	2.60E-08	4.56 (3.40-5.73)	1.67E-14	8.56E-11	2.19 (0.89-3.49)	9.39E-04	1.00E+00
NOX4	2	5.00 (3.50-6.50)	6.23E-11	3.11E-07	4.18 (2.77-5.60)	7.19E-09	3.69E-05	4.09 (1.68-6.50)	8.68E-04	1.00E+00
NR1I2	2	5.77 (4.16-7.38)	2.06E-12	1.03E-08	6.57 (4.96-8.18)	1.20E-15	6.32E-12			
NRG2	2	4.43 (2.97-5.88)	2.46E-09	1.23E-05	4.75 (3.32-6.18)	8.25E-11	4.24E-07	3.40 (1.43-5.36)	7.18E-04	1.00E+00
NRP2	4	2.64 (1.64-3.64)	2.09E-07	1.04E-03	2.52 (1.53-3.51)	6.62E-07	3.40E-03	2.01 (0.91-3.12)	3.57E-04	1.00E+00
NT5E	2	3.14 (1.73-4.56)	1.29E-05	6.42E-02	3.24 (1.84-4.65)	6.05E-06	3.11E-02	2.70 (1.00-4.41)	1.91E-03	1.00E+00
NTNG2	4	2.09 (1.09-3.09)	4.09E-05	2.04E-01	2.35 (1.35-3.35)	3.80E-06	1.95E-02	1.66 (0.58-2.73)	2.55E-03	1.00E+00
NUAK2	2	4.52 (3.06-5.98)	1.31E-09	6.56E-06	4.04 (2.63-5.46)	2.11E-08	1.08E-04	2.01 (0.45-3.57)	1.16E-02	1.00E+00
NUDT19	2	3.29 (1.87-4.70)	5.35E-06	2.67E-02	3.81 (2.39-5.22)	1.24E-07	6.36E-04	4.09 (1.68-6.50)	8.68E-04	1.00E+00
NUP210	2	3.82 (2.38-5.26)	1.97E-07	9.84E-04	3.42 (2.00-4.84)	2.23E-06	1.14E-02	3.00 (1.20-4.81)	1.11E-03	1.00E+00
OR10J5	2	2.92 (1.51-4.33)	4.93E-05	2.46E-01	3.38 (1.98-4.79)	2.39E-06	1.23E-02	2.99 (1.19-4.79)	1.11E-03	1.00E+00
OR10X1	6	2.26 (1.45-3.08)	5.40E-08	2.70E-04	2.83 (2.02-3.65)	9.60E-12	4.93E-08	1.28 (0.42-2.14)	3.41E-03	1.00E+00
OR14A2	3	2.37 (1.22-3.52)	5.57E-05	2.78E-01	2.77 (1.62-3.92)	2.32E-06	1.19E-02	1.24 (0.03-2.45)	4.45E-02	1.00E+00
OR2A5	3	7.98 (5.71-10.25)	5.64E-12	2.82E-08	6.83 (5.46-8.19)	0.00E+00	0.00E+00			
OR4A16	2	4.79 (3.31-6.27)	2.31E-10	1.15E-06	4.89 (3.45-6.33)	2.71E-11	1.39E-07	2.70 (1.00-4.41)	1.91E-03	1.00E+00
OR4C11	2	3.36 (1.93-4.79)	3.98E-06	1.99E-02	4.04 (2.61-5.46)	2.90E-08	1.49E-04	3.00 (1.20-4.81)	1.11E-03	1.00E+00
OR6P1	4	1.73 (0.74-2.73)	6.13E-04	1.00E+00	2.26 (1.27-3.25)	7.92E-06	4.07E-02	1.14 (0.10-2.18)	3.11E-02	1.00E+00
OTOGL	5	2.32 (1.43-3.21)	3.10E-07	1.55E-03	2.24 (1.35-3.12)	7.38E-07	3.79E-03	1.67 (0.71-2.63)	6.29E-04	1.00E+00
OVOL2	2	4.16 (2.72-5.60)	1.51E-08	7.54E-05	3.85 (2.44-5.26)	8.97E-08	4.61E-04			
P2RY6	2	3.17 (1.75-4.59)	1.26E-05	6.29E-02	3.73 (2.31-5.15)	2.59E-07	1.33E-03	3.41 (1.43-5.39)	7.15E-04	1.00E+00
PAK5	3	5.83 (4.50-7.17)	0.00E+00	6.32E-14	5.12 (3.93-6.31)	0.00E+00	1.67E-13	4.50 (2.23-6.77)	1.01E-04	4.33E-01
PAM	2	4.39 (2.93-5.84)	3.26E-09	1.63E-05	4.22 (2.80-5.63)	5.58E-09	2.87E-05			
PCDH10	2	4.49 (3.02-5.96)	2.15E-09	1.07E-05	4.01 (2.58-5.43)	3.56E-08	1.83E-04			
PCM1	5	4.04 (3.13-4.95)	0.00E+00	1.49E-14	3.84 (2.95-4.73)	0.00E+00	1.78E-13	3.91 (2.47-5.34)	9.60E-08	4.11E-04
PDIA4	4	2.33 (1.34-3.33)	4.04E-06	2.02E-02	2.70 (1.71-3.69)	9.15E-08	4.70E-04	2.70 (1.50-3.91)	1.13E-05	4.85E-02
PDPR	5	2.15 (1.26-3.04)	2.27E-06	1.14E-02	2.61 (1.72-3.50)	8.73E-09	4.49E-05	1.30 (0.36-2.23)	6.87E-03	1.00E+00
PDZD2	3	2.35 (1.21-3.50)	5.65E-05	2.82E-01	2.67 (1.53-3.81)	4.77E-06	2.45E-02	1.50 (0.27-2.72)	1.64E-02	1.00E+00
PER1	2	4.47 (3.01-5.93)	1.81E-09	9.06E-06	4.99 (3.55-6.43)	1.19E-11	6.13E-08	3.40 (1.43-5.36)	7.18E-04	1.00E+00
PHKA2	5	1.68 (0.79-2.57)	2.17E-04	1.00E+00	2.02 (1.13-2.91)	8.95E-06	4.60E-02	1.51 (0.56-2.46)	1.81E-03	1.00E+00
PHLDA1	2	4.44 (2.98-5.91)	2.78E-09	1.39E-05	5.01 (3.55-6.46)	1.44E-11	7.41E-08			

PHLDB3	3	2.16 (1.01-3.30)	2.23E-04	1.00E+00	2.70 (1.55-3.84)	3.81E-06	1.96E-02	2.10 (0.81-3.38)	1.39E-03	1.00E+00
PHYHD1	3	3.22 (2.05-4.39)	7.20E-08	3.60E-04	3.29 (2.12-4.45)	3.17E-08	1.63E-04	2.22 (0.90-3.53)	9.38E-04	1.00E+00
PIEZO1	10	1.96 (1.33-2.58)	1.02E-09	5.09E-06	1.95 (1.33-2.58)	9.92E-10	5.10E-06	1.54 (0.87-2.21)	7.11E-06	3.04E-02
PIEZO2	3	3.18 (2.03-4.34)	6.55E-08	3.27E-04	3.43 (2.28-4.58)	4.64E-09	2.38E-05	2.70 (1.31-4.10)	1.44E-04	6.14E-01
PIGR	3	3.93 (2.76-5.11)	5.02E-11	2.51E-07	4.47 (3.30-5.64)	5.99E-14	3.08E-10	2.55 (1.19-3.92)	2.40E-04	1.00E+00
PIK3C2G	4	2.70 (1.70-3.70)	1.32E-07	6.59E-04	2.63 (1.64-3.63)	2.29E-07	1.18E-03	2.49 (1.32-3.66)	3.09E-05	1.32E-01
PIK3R4	4	2.95 (1.95-3.94)	6.78E-09	3.39E-05	2.68 (1.69-3.68)	1.09E-07	5.58E-04	2.84 (1.60-4.07)	6.73E-06	2.88E-02
PIKFYVE	3	2.97 (1.82-4.12)	4.23E-07	2.11E-03	3.49 (2.34-4.64)	2.53E-09	1.30E-05	2.70 (1.31-4.10)	1.44E-04	6.14E-01
PITRM1	6	1.64 (0.83-2.46)	7.63E-05	3.81E-01	1.97 (1.16-2.78)	2.09E-06	1.07E-02	1.73 (0.85-2.62)	1.22E-04	5.20E-01
PKD1L1	4	2.28 (1.28-3.27)	7.13E-06	3.56E-02	2.67 (1.67-3.66)	1.43E-07	7.36E-04	2.30 (1.16-3.44)	7.68E-05	3.28E-01
PKD1L2	4	2.30 (1.31-3.30)	5.77E-06	2.88E-02	2.61 (1.61-3.60)	2.70E-07	1.39E-03	1.74 (0.66-2.82)	1.57E-03	1.00E+00
PKDREJ	8	2.00 (1.30-2.70)	2.34E-08	1.17E-04	2.34 (1.64-3.04)	6.16E-11	3.16E-07	1.92 (1.15-2.70)	1.15E-06	4.90E-03
PKHD1	5	2.38 (1.49-3.27)	1.53E-07	7.66E-04	2.79 (1.90-3.67)	7.10E-10	3.65E-06	2.37 (1.34-3.39)	6.35E-06	2.72E-02
PLCH1	5	1.99 (1.09-2.88)	1.25E-05	6.25E-02	2.39 (1.50-3.28)	1.42E-07	7.30E-04	2.45 (1.41-3.49)	4.03E-06	1.72E-02
PLEC	9	1.39 (0.73-2.05)	3.57E-05	1.78E-01	1.60 (0.94-2.26)	1.85E-06	9.48E-03	1.29 (0.59-1.98)	2.99E-04	1.00E+00
PLEKHH1	3	2.72 (1.57-3.86)	3.59E-06	1.79E-02	3.21 (2.07-4.36)	3.98E-08	2.05E-04	1.50 (0.27-2.72)	1.64E-02	1.00E+00
PLIN4	2				7.62 (5.65-9.59)	3.18E-14	1.63E-10			
PLXNA4	7	1.47 (0.72-2.22)	1.22E-04	6.11E-01	1.93 (1.18-2.68)	4.30E-07	2.21E-03	1.63 (0.82-2.44)	7.83E-05	3.35E-01
PLXNB2	4	2.12 (1.12-3.11)	3.03E-05	1.52E-01	2.55 (1.56-3.55)	4.55E-07	2.34E-03	2.99 (1.72-4.27)	4.01E-06	1.71E-02
PM20D1	6	1.63 (0.81-2.45)	1.01E-04	5.05E-01	1.95 (1.13-2.77)	3.03E-06	1.55E-02	1.47 (0.60-2.34)	9.66E-04	1.00E+00
PMFBP1	3	3.86 (2.69-5.03)	9.33E-11	4.66E-07	4.43 (3.27-5.60)	7.39E-14	3.80E-10			
PNPLA1	2	3.29 (1.87-4.70)	5.33E-06	2.66E-02	3.75 (2.34-5.15)	1.92E-07	9.88E-04	2.70 (1.00-4.41)	1.91E-03	1.00E+00
POLG	8	1.64 (0.94-2.35)	5.46E-06	2.73E-02	1.91 (1.20-2.62)	1.21E-07	6.24E-04	1.27 (0.52-2.02)	8.52E-04	1.00E+00
POLI	3	2.60 (1.45-3.75)	9.19E-06	4.59E-02	2.90 (1.75-4.04)	6.94E-07	3.57E-03	2.70 (1.31-4.10)	1.44E-04	6.14E-01
POLK	3	2.45 (1.30-3.60)	2.95E-05	1.47E-01	2.98 (1.83-4.13)	3.65E-07	1.88E-03	2.10 (0.81-3.39)	1.39E-03	1.00E+00
POLQ	5	2.63 (1.74-3.52)	6.55E-09	3.27E-05	2.31 (1.43-3.20)	2.98E-07	1.53E-03	1.39 (0.45-2.33)	3.73E-03	1.00E+00
POR	3	2.33 (1.18-3.48)	7.40E-05	3.70E-01	2.68 (1.53-3.82)	4.90E-06	2.52E-02	2.01 (0.74-3.29)	1.99E-03	1.00E+00
POU4F1	3	2.46 (1.31-3.60)	2.72E-05	1.36E-01	2.61 (1.46-3.75)	7.90E-06	4.06E-02	2.01 (0.73-3.28)	1.99E-03	1.00E+00
PRAM1	2	6.19 (4.47-7.90)	1.51E-12	7.52E-09	3.65 (2.23-5.07)	4.59E-07	2.36E-03			
PRDM2	3	2.62 (1.47-3.76)	7.95E-06	3.97E-02	3.07 (1.93-4.22)	1.49E-07	7.65E-04	3.40 (1.79-5.00)	3.43E-05	1.47E-01
PRR12	4	1.91 (0.92-2.90)	1.61E-04	8.02E-01	2.37 (1.38-3.36)	2.60E-06	1.34E-02	1.48 (0.43-2.54)	5.96E-03	1.00E+00

PRR14	2	6.87 (4.90-8.84)	7.70E-12	3.85E-08	7.67 (5.70-9.63)	2.21E-14	1.13E-10			
PRUNE2	7	1.54 (0.79-2.29)	5.74E-05	2.87E-01	1.89 (1.14-2.64)	7.33E-07	3.76E-03	1.26 (0.47-2.05)	1.74E-03	1.00E+00
PSD2	2	5.08 (3.57-6.59)	3.89E-11	1.95E-07	5.72 (4.23-7.21)	5.44E-14	2.79E-10			
PSD3	4	3.35 (2.34-4.35)	6.28E-11	3.14E-07	3.92 (2.92-4.92)	1.60E-14	8.21E-11	2.39 (1.23-3.54)	4.93E-05	2.11E-01
PSMD8	8	2.50 (1.79-3.20)	5.07E-12	2.53E-08	2.35 (1.65-3.06)	6.31E-11	3.24E-07	1.23 (0.49-1.98)	1.12E-03	1.00E+00
PSMF1	3	2.84 (1.69-3.99)	1.31E-06	6.56E-03	3.34 (2.20-4.49)	1.11E-08	5.68E-05	3.11 (1.60-4.61)	5.09E-05	2.18E-01
PTPRC	2	2.70 (1.30-4.11)	1.66E-04	8.29E-01	3.38 (1.97-4.78)	2.49E-06	1.28E-02	2.48 (0.83-4.13)	3.22E-03	1.00E+00
PTPRG	2	5.26 (3.73-6.79)	1.47E-11	7.36E-08	5.96 (4.45-7.48)	1.27E-14	6.53E-11			
PTPRJ	3	5.67 (4.37-6.96)	0.00E+00	5.68E-14	6.37 (5.08-7.65)	0.00E+00	0.00E+00			
PTPRZ1	2	3.95 (2.52-5.39)	6.45E-08	3.22E-04	4.28 (2.86-5.70)	3.36E-09	1.72E-05	2.48 (0.83-4.13)	3.22E-03	1.00E+00
RAB3GAP2	2	5.95 (4.31-7.60)	1.45E-12	7.24E-09	6.75 (5.10-8.40)	1.00E-15	5.15E-12	4.09 (1.68-6.50)	8.68E-04	1.00E+00
RAD21	2	4.34 (2.89-5.79)	4.31E-09	2.15E-05	3.61 (2.20-5.01)	5.16E-07	2.65E-03			
RAD21L1	3	2.18 (1.03-3.32)	2.08E-04	1.00E+00	2.68 (1.53-3.83)	4.72E-06	2.43E-02	1.24 (0.03-2.45)	4.45E-02	1.00E+00
RASGRP2	2	5.95 (4.31-7.60)	1.45E-12	7.25E-09	4.28 (2.86-5.70)	3.33E-09	1.71E-05	3.40 (1.43-5.36)	7.18E-04	1.00E+00
RC3H2	2	5.48 (3.93-7.04)	5.33E-12	2.66E-08	3.51 (2.10-4.92)	1.02E-06	5.23E-03	2.70 (1.00-4.41)	1.91E-03	1.00E+00
REV3L	2	7.56 (5.16-9.97)	7.25E-10	3.62E-06	4.72 (3.29-6.16)	1.01E-10	5.20E-07			
RHOJ	2	3.03 (1.61-4.46)	2.86E-05	1.43E-01	3.34 (1.92-4.75)	3.80E-06	1.95E-02	3.41 (1.43-5.39)	7.15E-04	1.00E+00
RIF1	4	2.31 (1.32-3.30)	4.95E-06	2.47E-02	2.78 (1.79-3.77)	3.70E-08	1.90E-04	2.14 (1.02-3.26)	1.73E-04	7.41E-01
RNF123	3	4.26 (3.08-5.44)	1.59E-12	7.96E-09	3.86 (2.71-5.01)	5.15E-11	2.64E-07	3.80 (2.01-5.60)	3.30E-05	1.41E-01
RNF169	4	2.30 (1.30-3.30)	6.70E-06	3.35E-02	2.51 (1.51-3.51)	8.17E-07	4.20E-03	1.90 (0.81-3.00)	6.77E-04	1.00E+00
RP1L1	8	1.94 (1.24-2.64)	5.99E-08	2.99E-04	2.27 (1.57-2.97)	2.02E-10	1.04E-06	1.69 (0.93-2.45)	1.27E-05	5.43E-02
RPRD2	3	2.71 (1.56-3.86)	3.75E-06	1.87E-02	3.28 (2.14-4.43)	1.99E-08	1.02E-04	2.10 (0.81-3.38)	1.39E-03	1.00E+00
RUSC2	2	3.48 (2.06-4.90)	1.56E-06	7.81E-03	4.13 (2.71-5.55)	1.09E-08	5.62E-05			
RUVBL2	2	5.62 (4.04-7.20)	3.27E-12	1.63E-08	3.71 (2.31-5.12)	2.39E-07	1.23E-03	4.09 (1.68-6.50)	8.68E-04	1.00E+00
SAMD9	5	2.09 (1.20-2.98)	4.04E-06	2.02E-02	2.45 (1.56-3.34)	6.17E-08	3.17E-04	2.06 (1.07-3.06)	4.65E-05	1.99E-01
SAP130	2	7.56 (5.16-9.97)	7.25E-10	3.62E-06	7.67 (5.70-9.63)	2.19E-14	1.12E-10	4.09 (1.68-6.50)	8.68E-04	1.00E+00
SBF1	2	5.00 (3.50-6.50)	6.19E-11	3.09E-07	5.18 (3.73-6.64)	2.66E-12	1.37E-08			
SCN10A	3	2.27 (1.13-3.42)	1.02E-04	5.12E-01	2.72 (1.57-3.86)	3.21E-06	1.65E-02	1.66 (0.42-2.89)	8.50E-03	1.00E+00
SCN7A	4	2.18 (1.19-3.18)	1.70E-05	8.51E-02	2.49 (1.50-3.48)	8.89E-07	4.57E-03	2.15 (1.03-3.27)	1.73E-04	7.40E-01
SCRIB	2	3.40 (1.99-4.82)	2.51E-06	1.26E-02	3.99 (2.58-5.40)	3.12E-08	1.60E-04	4.09 (1.68-6.50)	8.68E-04	1.00E+00
SCUBE2	2	3.98 (2.55-5.41)	5.32E-08	2.66E-04	4.60 (3.17-6.03)	2.73E-10	1.40E-06	3.40 (1.43-5.36)	7.18E-04	1.00E+00

SDK2	6	2.91 (2.09-3.72)	3.18E-12	1.59E-08	3.19 (2.38-4.01)	1.53E-14	7.86E-11	3.25 (2.15-4.35)	6.51E-09	2.79E-05
SEMA3F	2	5.97 (4.31-7.63)	1.68E-12	8.39E-09	4.91 (3.46-6.36)	3.08E-11	1.58E-07	3.41 (1.43-5.39)	7.15E-04	1.00E+00
SEMA3G	4	2.48 (1.48-3.48)	1.20E-06	5.97E-03	2.45 (1.46-3.45)	1.43E-06	7.33E-03	1.49 (0.43-2.56)	5.90E-03	1.00E+00
SEPTIN8	2	6.89 (4.91-8.86)	8.24E-12	4.11E-08	6.77 (5.11-8.42)	1.20E-15	6.28E-12			
SETD1A	3	2.43 (1.28-3.58)	3.29E-05	1.64E-01	2.99 (1.84-4.13)	3.11E-07	1.60E-03	3.40 (1.79-5.00)	3.43E-05	1.47E-01
SETD2	3	3.53 (2.37-4.69)	2.53E-09	1.26E-05	3.82 (2.67-4.97)	8.31E-11	4.27E-07			
SFPQ	3	3.84 (2.67-5.01)	1.12E-10	5.61E-07	4.42 (3.26-5.58)	8.84E-14	4.54E-10	2.41 (1.08-3.75)	3.91E-04	1.00E+00
SGSM1	2	4.23 (2.79-5.68)	9.39E-09	4.69E-05	4.90 (3.46-6.33)	2.57E-11	1.32E-07	4.09 (1.68-6.50)	8.68E-04	1.00E+00
SHANK1	5	3.21 (2.31-4.10)	2.23E-12	1.11E-08	3.43 (2.54-4.32)	4.33E-14	2.22E-10	2.52 (1.47-3.57)	2.50E-06	1.07E-02
SIPA1L2	2	3.67 (2.23-5.10)	5.40E-07	2.70E-03	4.23 (2.80-5.66)	6.32E-09	3.25E-05	3.41 (1.43-5.39)	7.15E-04	1.00E+00
SIPA1L3	5	2.05 (1.16-2.95)	7.23E-06	3.61E-02	2.15 (1.26-3.05)	2.47E-06	1.27E-02	1.93 (0.95-2.92)	1.24E-04	5.31E-01
SLC12A9	2	3.50 (2.07-4.93)	1.60E-06	7.98E-03	3.76 (2.34-5.18)	2.12E-07	1.09E-03	1.90 (0.35-3.45)	1.62E-02	1.00E+00
SLC17A1	2	3.04 (1.62-4.47)	2.69E-05	1.34E-01	3.43 (2.02-4.85)	2.03E-06	1.04E-02	2.02 (0.45-3.59)	1.16E-02	1.00E+00
SLC22A3	2	2.84 (1.44-4.25)	7.56E-05	3.77E-01	3.42 (2.01-4.82)	1.89E-06	9.69E-03	1.45 (-0.04-2.94)	5.73E-02	1.00E+00
SLC27A5	6	1.77 (0.95-2.58)	1.99E-05	9.95E-02	2.05 (1.24-2.87)	6.77E-07	3.48E-03	1.58 (0.71-2.45)	3.79E-04	1.00E+00
SLC2A10	2	6.47 (4.67-8.26)	1.78E-12	8.88E-09	6.75 (5.10-8.40)	1.00E-15	5.12E-12	4.09 (1.68-6.50)	8.68E-04	1.00E+00
SLC46A3	2	7.56 (5.16-9.97)	7.24E-10	3.62E-06	8.36 (5.95-10.77)	9.78E-12	5.02E-08			
SLC49A3	3	4.68 (3.47-5.88)	2.76E-14	1.38E-10	4.26 (3.10-5.42)	6.92E-13	3.56E-09			
SLC9A4	3	2.55 (1.40-3.70)	1.43E-05	7.13E-02	3.15 (2.00-4.30)	7.86E-08	4.04E-04	2.10 (0.81-3.39)	1.39E-03	1.00E+00
SLIT2	2	6.87 (4.90-8.84)	7.63E-12	3.81E-08	7.26 (5.47-9.06)	2.40E-15	1.22E-11	4.09 (1.68-6.50)	8.68E-04	1.00E+00
SMARCAL1	4	2.43 (1.43-3.42)	1.79E-06	8.94E-03	2.87 (1.87-3.86)	1.59E-08	8.15E-05	2.08 (0.97-3.19)	2.51E-04	1.00E+00
SMG6	2	3.10 (1.68-4.52)	1.93E-05	9.65E-02	3.60 (2.19-5.02)	6.31E-07	3.24E-03	1.80 (0.26-3.33)	2.20E-02	1.00E+00
SMIM32	2	4.26 (2.77-5.75)	2.10E-08	1.05E-04	3.91 (2.48-5.35)	9.01E-08	4.63E-04			
SNTB1	2	5.37 (3.83-6.91)	8.89E-12	4.44E-08	5.59 (4.11-7.07)	1.34E-13	6.87E-10			
SOGA1	2	4.87 (3.38-6.37)	1.71E-10	8.52E-07	3.35 (1.94-4.77)	3.49E-06	1.79E-02	2.72 (1.00-4.43)	1.91E-03	1.00E+00
SORBS2	2	5.37 (3.83-6.91)	8.89E-12	4.44E-08	4.06 (2.64-5.47)	1.91E-08	9.80E-05			
SPARC	2	3.45 (2.03-4.87)	1.84E-06	9.18E-03	4.20 (2.78-5.62)	6.25E-09	3.21E-05			
SPINK5	5	2.14 (1.25-3.03)	2.56E-06	1.28E-02	2.55 (1.66-3.44)	2.06E-08	1.06E-04	3.07 (1.91-4.22)	1.98E-07	8.46E-04
SPTA1	9	1.29 (0.63-1.95)	1.26E-04	6.32E-01	1.62 (0.96-2.28)	1.62E-06	8.34E-03	1.36 (0.66-2.06)	1.45E-04	6.18E-01
SPTB	2	4.47 (3.01-5.92)	1.88E-09	9.39E-06	3.87 (2.45-5.28)	8.01E-08	4.12E-04			
SPTBN5	8	1.91 (1.20-2.61)	1.07E-07	5.32E-04	2.39 (1.69-3.10)	2.57E-11	1.32E-07	2.30 (1.50-3.11)	2.24E-08	9.59E-05

SPTLC2	4	2.73 (1.71-3.75)	1.44E-07	7.18E-04	2.64 (1.63-3.65)	3.32E-07	1.70E-03	1.87 (0.77-2.98)	9.01E-04	1.00E+00
SRGAP3	9	3.15 (2.48-3.82)	0.00E+00	2.00E-16	3.66 (2.99-4.33)	0.00E+00	0.00E+00	2.90 (2.06-3.73)	9.85E-12	4.21E-08
ST18	2	2.65 (1.24-4.06)	2.21E-04	1.00E+00	3.22 (1.81-4.62)	7.04E-06	3.62E-02	2.48 (0.83-4.13)	3.22E-03	1.00E+00
ST7L	3	5.40 (4.14-6.67)	0.00E+00	2.49E-13	5.51 (4.31-6.71)	0.00E+00	1.50E-15	4.49 (2.23-6.76)	1.02E-04	4.38E-01
STAB1	9	3.16 (2.49-3.82)	0.00E+00	1.00E-16	3.55 (2.88-4.21)	0.00E+00	0.00E+00	2.10 (1.36-2.84)	3.06E-08	1.31E-04
STARD9	8	1.75 (1.05-2.45)	9.11E-07	4.55E-03	2.08 (1.38-2.78)	5.61E-09	2.88E-05	1.67 (0.91-2.42)	1.60E-05	6.85E-02
SVIL	2	6.46 (4.67-8.26)	1.79E-12	8.92E-09	6.75 (5.10-8.40)	1.00E-15	5.19E-12			
SYNE2	15	1.50 (0.99-2.01)	8.44E-09	4.22E-05	1.86 (1.35-2.37)	1.05E-12	5.41E-09	1.17 (0.64-1.71)	1.85E-05	7.90E-02
SYNE3	2	5.74 (4.13-7.36)	3.48E-12	1.74E-08	5.85 (4.34-7.37)	3.88E-14	2.00E-10			
SYNE4	2	5.63 (4.04-7.22)	3.82E-12	1.91E-08	4.53 (3.09-5.96)	6.48E-10	3.33E-06	4.10 (1.69-6.52)	8.58E-04	1.00E+00
SYNPO	2	3.76 (2.33-5.18)	2.45E-07	1.22E-03	4.13 (2.71-5.54)	1.12E-08	5.75E-05	2.99 (1.19-4.79)	1.11E-03	1.00E+00
SYTL2	2	2.78 (1.38-4.19)	1.06E-04	5.31E-01	3.29 (1.89-4.70)	4.39E-06	2.25E-02	2.30 (0.69-3.91)	5.17E-03	1.00E+00
SZT2	4	1.95 (0.95-2.94)	1.19E-04	5.96E-01	2.36 (1.37-3.35)	3.05E-06	1.56E-02	1.56 (0.50-2.62)	3.99E-03	1.00E+00
TAF6L	5	1.97 (1.07-2.86)	1.71E-05	8.53E-02	2.29 (1.40-3.19)	5.11E-07	2.63E-03	1.62 (0.66-2.58)	9.75E-04	1.00E+00
TARBP1	2	2.97 (1.56-4.38)	3.66E-05	1.83E-01	3.47 (2.06-4.88)	1.34E-06	6.86E-03	2.70 (1.00-4.41)	1.91E-03	1.00E+00
TBC1D32	2	6.87 (4.90-8.84)	7.65E-12	3.82E-08	5.65 (4.17-7.14)	8.67E-14	4.45E-10			
TBL1X	2	7.33 (4.92-9.75)	2.57E-09	1.28E-05	8.07 (5.66-10.49)	5.42E-11	2.78E-07			
TCHH	5	1.84 (0.96-2.73)	4.59E-05	2.29E-01	2.00 (1.12-2.89)	8.90E-06	4.57E-02	1.60 (0.65-2.55)	9.89E-04	1.00E+00
TDRD6	8	1.51 (0.81-2.22)	2.57E-05	1.29E-01	1.89 (1.19-2.60)	1.37E-07	7.05E-04	1.32 (0.58-2.07)	4.98E-04	1.00E+00
TEDC1	2	4.20 (2.75-5.64)	1.20E-08	5.98E-05	4.13 (2.71-5.54)	1.12E-08	5.73E-05	2.70 (1.00-4.41)	1.91E-03	1.00E+00
TEP1	4	3.04 (2.04-4.03)	2.46E-09	1.23E-05	3.32 (2.32-4.31)	6.02E-11	3.09E-07	2.48 (1.31-3.64)	3.09E-05	1.32E-01
TERT	3	2.21 (1.06-3.36)	1.65E-04	8.26E-01	2.65 (1.50-3.79)	6.26E-06	3.22E-02	2.55 (1.19-3.92)	2.40E-04	1.00E+00
TESK2	2	4.39 (2.93-5.84)	3.26E-09	1.63E-05	3.73 (2.32-5.14)	2.21E-07	1.14E-03	3.40 (1.43-5.36)	7.18E-04	1.00E+00
TET3	2	7.56 (5.16-9.97)	7.25E-10	3.62E-06	7.67 (5.70-9.64)	2.18E-14	1.12E-10			
TEX37	2	3.10 (1.68-4.52)	1.94E-05	9.67E-02	3.21 (1.79-4.62)	8.70E-06	4.47E-02	2.72 (1.00-4.43)	1.91E-03	1.00E+00
TG	3	3.27 (2.11-4.42)	2.94E-08	1.47E-04	3.78 (2.62-4.93)	1.30E-10	6.70E-07	3.11 (1.60-4.61)	5.09E-05	2.18E-01
THBS1	2	4.20 (2.75-5.64)	1.20E-08	5.98E-05	4.89 (3.46-6.33)	2.57E-11	1.32E-07	2.99 (1.19-4.79)	1.11E-03	1.00E+00
THEG	2	5.62 (4.04-7.20)	3.25E-12	1.62E-08	5.47 (4.00-6.94)	3.10E-13	1.59E-09	3.40 (1.43-5.36)	7.18E-04	1.00E+00
TLN2	7	1.89 (1.14-2.64)	8.22E-07	4.10E-03	2.17 (1.42-2.92)	1.30E-08	6.68E-05	1.58 (0.77-2.38)	1.20E-04	5.13E-01
TLR2	5	2.42 (1.53-3.31)	9.31E-08	4.65E-04	2.71 (1.82-3.59)	2.15E-09	1.10E-05	1.39 (0.45-2.33)	3.73E-03	1.00E+00
TLR5	9	1.21 (0.55-1.88)	3.22E-04	1.00E+00	1.51 (0.85-2.17)	7.61E-06	3.91E-02	1.61 (0.89-2.32)	9.84E-06	4.21E-02

TMC8	2	6.47 (4.67-8.26)	1.78E-12	8.88E-09	5.37 (3.90-6.83)	6.67E-13	3.43E-09			
TMEM41A	8	3.01 (2.29-3.72)	2.00E-16	7.60E-13	3.00 (2.29-3.71)	1.00E-16	5.96E-13	1.64 (0.88-2.41)	2.44E-05	1.04E-01
TMEM64	2	5.16 (3.64-6.67)	2.62E-11	1.31E-07	5.36 (3.89-6.82)	7.37E-13	3.79E-09	2.01 (0.45-3.57)	1.16E-02	1.00E+00
TMEM71	2	2.73 (1.33-4.14)	1.40E-04	6.97E-01	3.30 (1.90-4.71)	4.04E-06	2.07E-02	2.99 (1.19-4.79)	1.11E-03	1.00E+00
TMEM8B	4	1.99 (1.00-2.98)	8.63E-05	4.31E-01	2.49 (1.50-3.48)	8.73E-07	4.49E-03	2.48 (1.32-3.65)	3.09E-05	1.32E-01
TMEM94	2	5.17 (3.65-6.68)	2.42E-11	1.21E-07	5.42 (3.95-6.88)	4.57E-13	2.35E-09	4.09 (1.68-6.50)	8.68E-04	1.00E+00
TNFRSF10A	6	4.48 (3.62-5.34)	0.00E+00	0.00E+00	4.11 (3.28-4.94)	0.00E+00	0.00E+00	2.73 (1.73-3.73)	7.62E-08	3.26E-04
TNFRSF21	3	2.42 (1.25-3.58)	4.72E-05	2.36E-01	2.97 (1.81-4.14)	5.29E-07	2.72E-03	3.14 (1.62-4.65)	5.07E-05	2.17E-01
TNRC6C	2	4.67 (3.20-6.14)	4.71E-10	2.35E-06	4.90 (3.46-6.33)	2.57E-11	1.32E-07	4.09 (1.68-6.50)	8.68E-04	1.00E+00
TOPBP1	2	3.31 (1.90-4.73)	4.45E-06	2.22E-02	3.72 (2.31-5.13)	2.37E-07	1.22E-03	2.48 (0.83-4.13)	3.22E-03	1.00E+00
TRAM2	2	2.72 (1.31-4.13)	1.52E-04	7.60E-01	3.20 (1.79-4.60)	8.14E-06	4.18E-02	1.89 (0.35-3.43)	1.63E-02	1.00E+00
TRERF1	2	4.31 (2.86-5.75)	5.66E-09	2.83E-05	4.83 (3.40-6.27)	4.19E-11	2.15E-07	3.40 (1.43-5.36)	7.18E-04	1.00E+00
TRIM21	2	3.47 (2.05-4.89)	1.65E-06	8.25E-03	4.10 (2.68-5.51)	1.39E-08	7.16E-05	4.09 (1.68-6.50)	8.68E-04	1.00E+00
TRIM26	3	5.06 (3.83-6.29)	8.00E-16	3.75E-12	5.11 (3.92-6.29)	0.00E+00	1.42E-13			
TRIM28	2	3.07 (1.66-4.49)	1.97E-05	9.83E-02	3.73 (2.32-5.13)	2.21E-07	1.14E-03	2.14 (0.56-3.72)	7.92E-03	1.00E+00
TRPM2	3	4.92 (3.71-6.14)	2.20E-15	1.12E-11	5.30 (4.11-6.49)	0.00E+00	1.44E-14	3.80 (2.01-5.60)	3.30E-05	1.41E-01
TRPM8	2	6.18 (4.47-7.88)	1.27E-12	6.32E-09	4.39 (2.97-5.81)	1.43E-09	7.37E-06	3.40 (1.43-5.36)	7.18E-04	1.00E+00
TRPV1	4	2.51 (1.52-3.51)	7.51E-07	3.75E-03	2.97 (1.98-3.97)	4.57E-09	2.35E-05	1.65 (0.58-2.72)	2.56E-03	1.00E+00
TTC6	7	2.90 (2.14-3.65)	5.62E-14	2.81E-10	2.73 (1.98-3.48)	1.07E-12	5.49E-09	2.95 (2.00-3.90)	1.37E-09	5.84E-06
TTLL11	5	2.66 (1.76-3.56)	7.04E-09	3.52E-05	2.89 (1.99-3.79)	2.62E-10	1.34E-06	2.03 (1.03-3.02)	6.53E-05	2.79E-01
TTN	38	1.48 (1.16-1.80)	0.00E+00	1.00E-15	1.59 (1.27-1.91)	0.00E+00	0.00E+00	1.20 (0.86-1.54)	3.16E-12	1.35E-08
TUBGCP6	2	2.96 (1.55-4.37)	3.92E-05	1.96E-01	3.45 (2.04-4.85)	1.54E-06	7.93E-03	2.14 (0.56-3.72)	7.92E-03	1.00E+00
UNC79	4	2.50 (1.51-3.50)	7.89E-07	3.94E-03	2.97 (1.98-3.97)	4.12E-09	2.12E-05	2.70 (1.50-3.91)	1.13E-05	4.85E-02
USP48	2	3.33 (1.91-4.75)	4.06E-06	2.03E-02	3.52 (2.11-4.92)	9.68E-07	4.97E-03	2.70 (1.00-4.41)	1.91E-03	1.00E+00
VPS13C	3	2.49 (1.33-3.64)	2.32E-05	1.16E-01	2.77 (1.62-3.92)	2.22E-06	1.14E-02	1.86 (0.60-3.12)	3.79E-03	1.00E+00
VSIG10	2	2.68 (1.27-4.09)	1.89E-04	9.44E-01	3.22 (1.81-4.62)	7.12E-06	3.66E-02	2.30 (0.69-3.91)	5.17E-03	1.00E+00
VWA5B1	4	5.21 (4.14-6.29)	0.00E+00	0.00E+00	5.20 (4.18-6.23)	0.00E+00	0.00E+00	3.40 (2.00-4.79)	1.72E-06	7.36E-03
VWDE	3	2.38 (1.23-3.53)	4.90E-05	2.45E-01	2.62 (1.47-3.77)	7.78E-06	4.00E-02	2.55 (1.19-3.92)	2.40E-04	1.00E+00
WAPL	2	7.56 (5.16-9.97)	7.25E-10	3.62E-06	8.36 (5.95-10.77)	9.77E-12	5.02E-08			
WDHD1	9	2.75 (2.08-3.43)	1.40E-15	6.89E-12	2.63 (1.96-3.30)	1.47E-14	7.55E-11	2.40 (1.63-3.18)	1.28E-09	5.46E-06
WDR36	7	1.42 (0.67-2.17)	2.12E-04	1.00E+00	1.73 (0.99-2.48)	5.73E-06	2.94E-02	1.45 (0.65-2.25)	3.73E-04	1.00E+00

WDR87	2	7.56 (5.16-9.97)	7.24E-10	3.62E-06	7.26 (5.47-9.06)	2.40E-15	1.22E-11			
WIPF3	2	4.23 (2.79-5.68)	9.40E-09	4.70E-05	4.67 (3.24-6.10)	1.54E-10	7.89E-07			
WNK2	18	2.74 (2.26-3.21)	0.00E+00	0.00E+00	2.94 (2.47-3.41)	0.00E+00	0.00E+00	3.21 (2.58-3.84)	0.00E+00	0.00E+00
WNT9B	2	2.67 (1.26-4.08)	1.97E-04	9.85E-01	3.18 (1.78-4.59)	8.86E-06	4.55E-02	4.09 (1.68-6.50)	8.68E-04	1.00E+00
XIRP2	6	1.59 (0.78-2.39)	1.20E-04	6.01E-01	1.99 (1.19-2.80)	1.30E-06	6.69E-03	1.11 (0.26-1.95)	1.04E-02	1.00E+00
YTHDC1	3	2.44 (1.29-3.59)	3.23E-05	1.62E-01	2.69 (1.54-3.84)	4.28E-06	2.20E-02	1.79 (0.54-3.04)	5.05E-03	1.00E+00
ZBED9	2	6.87 (4.90-8.84)	7.64E-12	3.82E-08	3.26 (1.86-4.66)	5.38E-06	2.77E-02			
ZBTB21	2	4.34 (2.89-5.79)	4.32E-09	2.16E-05	3.91 (2.49-5.32)	5.94E-08	3.05E-04			
ZC3H18	2	3.73 (2.29-5.16)	3.63E-07	1.81E-03	3.76 (2.34-5.18)	2.15E-07	1.10E-03	1.80 (0.26-3.33)	2.20E-02	1.00E+00
ZC3H3	3	4.04 (2.86-5.21)	1.49E-11	7.45E-08	4.18 (3.02-5.34)	1.40E-12	7.21E-09	4.49 (2.23-6.76)	1.02E-04	4.38E-01
ZC3H4	2	7.56 (5.16-9.97)	7.25E-10	3.62E-06	6.42 (4.83-8.00)	1.80E-15	9.21E-12			
ZFHX3	12	1.64 (1.07-2.21)	1.92E-08	9.61E-05	1.61 (1.04-2.18)	3.48E-08	1.79E-04	1.02 (0.42-1.61)	8.28E-04	1.00E+00
ZFP30	2	5.01 (3.51-6.52)	7.23E-11	3.61E-07	5.54 (4.06-7.03)	2.55E-13	1.31E-09	2.31 (0.69-3.93)	5.17E-03	1.00E+00
ZNF212	3	3.29 (2.14-4.45)	2.39E-08	1.19E-04	3.72 (2.57-4.87)	2.36E-10	1.21E-06	4.49 (2.23-6.76)	1.02E-04	4.38E-01
ZNF24	2	3.40 (1.99-4.82)	2.51E-06	1.25E-02	3.97 (2.55-5.38)	3.78E-08	1.94E-04	2.14 (0.56-3.72)	7.92E-03	1.00E+00
ZNF266	4	2.88 (1.88-3.87)	1.66E-08	8.31E-05	3.16 (2.17-4.16)	4.69E-10	2.41E-06	4.09 (2.39-5.80)	2.46E-06	1.05E-02
ZNF324	2	3.76 (2.33-5.18)	2.44E-07	1.22E-03	4.16 (2.74-5.57)	8.90E-09	4.57E-05	2.48 (0.83-4.13)	3.22E-03	1.00E+00
ZNF334	2	3.92 (2.49-5.36)	7.83E-08	3.91E-04	4.21 (2.80-5.63)	5.68E-09	2.92E-05			
ZNF474	2	6.18 (4.47-7.88)	1.27E-12	6.32E-09	6.06 (4.53-7.59)	7.70E-15	3.95E-11			
ZNF609	4	2.51 (1.52-3.51)	7.04E-07	3.51E-03	2.44 (1.45-3.43)	1.33E-06	6.83E-03	1.52 (0.46-2.58)	4.90E-03	1.00E+00
ZNF614	4	1.88 (0.89-2.88)	2.03E-04	1.00E+00	2.34 (1.35-3.34)	3.67E-06	1.88E-02	1.45 (0.39-2.51)	7.15E-03	1.00E+00
ZNF653	2	4.67 (3.20-6.14)	4.72E-10	2.36E-06	4.34 (2.91-5.76)	2.21E-09	1.14E-05			
ZNF775	2	2.95 (1.54-4.36)	4.18E-05	2.09E-01	3.54 (2.13-4.95)	8.24E-07	4.24E-03	3.40 (1.43-5.36)	7.18E-04	1.00E+00
ZNF813	2				8.38 (5.96-10.79)	1.01E-11	5.20E-08	4.10 (1.69-6.52)	8.58E-04	1.00E+00
ZNF860	2				7.67 (5.70-9.64)	2.18E-14	1.12E-10			
ZP4	2	3.87 (2.44-5.30)	1.10E-07	5.49E-04	4.47 (3.04-5.89)	7.74E-10	3.98E-06			
ZPR1	5	6.05 (4.98-7.11)	0.00E+00	0.00E+00	5.84 (4.88-6.79)	0.00E+00	0.00E+00			

Supplementary table 2c. Swedish replication cohort (JAGUAR). Genes with enrichment in LoF variants in tinnitus patients (N=147) and control (N=151) cohorts. Only genes with significant enrichment when compared with gnomAD database are shown. Odds ratio (OR) were calculated for the allelic frequencies reported in Non-Finish European (NFE) from gnomAD, all individuals form gnomAD and Swegen. If no LoF variants was reported in NFE or Swegen cohorts, OR cannot be calculated and cells are empty. Multiple hypothesis testing correction for each pvalue have been addressed by False Discovery Rate (FDR) approach for the total number of genes and total number of variants for each comparison.

Gene	N Variants	logOR (CI) NFE	pvalue	pvalue corrected	logOR (CI) ALL	pvalue	pvalue corrected	logOR (CI) Swedish	pvalue	pvalue corrected
ABCA13	2	4.30 (2.69-5.90)	1.47E-07	7.52E-05	4.94 (3.37-6.51)	7.61E-10	3.98E-07	2.61 (0.21-5.02)	3.30E-02	1.00E+00
ANKRD20A4F	5	2.36 (1.45-3.27)	3.38E-07	1.72E-04	2.76 (1.86-3.66)	1.82E-09	9.51E-07	1.24 (0.16-2.32)	2.49E-02	1.00E+00
ANO7	9	1.22 (0.56-1.89)	3.01E-04	1.54E-01	1.38 (0.72-2.05)	4.02E-05	2.10E-02	0.41 (-0.32-1.13)	2.73E-01	1.00E+00
CCDC9B	3	1.86 (0.71-3.01)	1.54E-03	7.86E-01	2.49 (1.35-3.64)	2.04E-05	1.07E-02	0.82 (-0.49-2.13)	2.19E-01	1.00E+00
CCNH	2	6.09 (3.69-8.49)	6.75E-07	3.44E-04	6.89 (4.48-9.29)	1.92E-08	1.01E-05	2.61 (0.21-5.02)	3.30E-02	1.00E+00
CFTR	2	3.04 (1.59-4.50)	4.02E-05	2.05E-02	3.33 (1.90-4.76)	4.81E-06	2.52E-03	1.23 (-0.47-2.93)	1.57E-01	1.00E+00
CHDH	2	4.01 (2.46-5.57)	4.19E-07	2.14E-04	4.69 (3.15-6.23)	2.18E-09	1.14E-06	1.92 (-0.04-3.89)	5.49E-02	1.00E+00
CHRNA5	2	4.99 (3.20-6.78)	4.81E-08	2.45E-05	5.09 (3.49-6.69)	4.76E-10	2.49E-07			
CLEC4C	3	2.03 (0.88-3.18)	5.67E-04	2.89E-01	2.63 (1.48-3.78)	7.51E-06	3.93E-03	0.94 (-0.39-2.27)	1.66E-01	1.00E+00
CRYBG2	3	2.01 (0.85-3.17)	6.54E-04	3.33E-01	2.47 (1.32-3.62)	2.62E-05	1.37E-02	0.82 (-0.49-2.14)	2.18E-01	1.00E+00
DUOX1	4	2.40 (1.39-3.41)	3.36E-06	1.71E-03	2.68 (1.67-3.68)	1.61E-07	8.43E-05	1.37 (0.13-2.60)	2.99E-02	1.00E+00
FUT10	2	4.71 (3.00-6.41)	5.92E-08	3.02E-05	5.10 (3.49-6.70)	4.75E-10	2.49E-07	2.62 (0.21-5.02)	3.29E-02	1.00E+00
GNA14	2	4.99 (3.20-6.79)	4.81E-08	2.45E-05	4.81 (3.26-6.36)	1.31E-09	6.84E-07	1.01 (-0.64-2.65)	2.31E-01	1.00E+00
GPLD1	6	1.62 (0.81-2.44)	9.83E-05	5.01E-02	1.66 (0.85-2.48)	6.08E-05	3.18E-02	0.38 (-0.51-1.27)	3.99E-01	1.00E+00
HRNR	2	3.69 (2.18-5.20)	1.72E-06	8.79E-04	3.93 (2.47-5.39)	1.33E-07	6.96E-05			
HSD3B1	4	1.86 (0.86-2.85)	2.65E-04	1.35E-01	2.41 (1.42-3.40)	2.02E-06	1.06E-03			
ITPRID1	2	2.79 (1.35-4.23)	1.42E-04	7.25E-02	3.22 (1.80-4.65)	9.14E-06	4.78E-03			
KIAA0040	4	1.58 (0.58-2.57)	1.85E-03	9.43E-01	2.25 (1.26-3.25)	8.72E-06	4.56E-03	2.62 (0.92-4.32)	2.55E-03	1.00E+00
MANSC1	4	2.28 (1.28-3.29)	8.35E-06	4.26E-03	2.98 (1.97-3.98)	5.97E-09	3.12E-06	1.52 (0.25-2.79)	1.90E-02	1.00E+00
MFSD4B	2	2.28 (0.86-3.71)	1.63E-03	8.30E-01	2.92 (1.50-4.33)	5.39E-05	2.82E-02	1.23 (-0.47-2.93)	1.57E-01	1.00E+00
MROH2A	2	4.48 (2.84-6.12)	8.95E-08	4.56E-05	4.69 (3.15-6.22)	2.11E-09	1.10E-06			
NUP214	8	1.64 (0.93-2.35)	6.39E-06	3.26E-03	1.66 (0.96-2.37)	3.93E-06	2.05E-03	0.97 (0.15-1.79)	2.11E-02	1.00E+00
OR6C76	2	2.43 (1.00-3.85)	8.46E-04	4.32E-01	3.02 (1.60-4.44)	3.09E-05	1.62E-02	0.82 (-0.78-2.43)	3.15E-01	1.00E+00
OSMR	2	4.30 (2.69-5.90)	1.48E-07	7.53E-05	4.94 (3.37-6.51)	7.63E-10	3.99E-07	2.61 (0.21-5.02)	3.30E-02	1.00E+00

PKHD1L1	2	2.72 (1.29-4.16)	2.03E-04	1.03E-01	3.42 (1.99-4.85)	2.81E-06	1.47E-03	2.61 (0.21-5.02)	3.30E-02	1.00E+00
POMGNT1	2	2.83 (1.39-4.28)	1.19E-04	6.05E-02	3.00 (1.58-4.42)	3.47E-05	1.81E-02	1.92 (-0.04-3.89)	5.49E-02	1.00E+00
PPP2R1B	3	2.74 (1.56-3.91)	5.01E-06	2.55E-03	3.41 (2.23-4.58)	1.20E-08	6.30E-06	1.93 (0.32-3.53)	1.87E-02	1.00E+00
PTCH2	3	5.80 (4.01-7.59)	2.15E-10	1.10E-07	6.60 (4.81-8.39)	5.10E-13	2.67E-10	1.92 (0.32-3.52)	1.88E-02	1.00E+00
RAB25	2	2.45 (1.03-3.88)	7.49E-04	3.82E-01	3.04 (1.62-4.46)	2.74E-05	1.43E-02	1.01 (-0.64-2.65)	2.31E-01	1.00E+00
REXO5	3	2.06 (0.90-3.21)	4.94E-04	2.52E-01	2.70 (1.55-3.86)	4.49E-06	2.35E-03	1.64 (0.14-3.14)	3.26E-02	1.00E+00
SLC16A8	8	1.07 (0.36-1.78)	3.03E-03	1.00E+00	1.64 (0.94-2.35)	5.09E-06	2.66E-03	1.12 (0.28-1.97)	8.83E-03	1.00E+00
SLC6A18	2	3.79 (2.27-5.31)	1.06E-06	5.41E-04	4.11 (2.64-5.58)	4.40E-08	2.30E-05			
SREK1IP1	4	6.76 (4.56-8.95)	1.59E-09	8.13E-07	6.15 (4.76-7.54)	0.00E+00	2.20E-15			
TNFRSF10A	2	2.76 (1.32-4.20)	1.71E-04	8.74E-02	3.45 (2.02-4.89)	2.36E-06	1.23E-03	0.31 (-1.21-1.83)	6.90E-01	1.00E+00
TRIM64	3	3.93 (2.14-5.73)	1.71E-05	8.71E-03	3.53 (2.10-4.97)	1.43E-06	7.48E-04	2.33 (0.54-4.13)	1.08E-02	1.00E+00
ZNF343	7	1.82 (1.06-2.58)	2.58E-06	1.31E-03	2.32 (1.56-3.07)	2.02E-09	1.05E-06	1.69 (0.69-2.68)	9.04E-04	3.66E-01
ZNF470	2	4.14 (2.57-5.72)	2.54E-07	1.29E-04	4.00 (2.53-5.46)	9.05E-08	4.74E-05			
ZNF83	2	4.70 (3.00-6.40)	5.87E-08	2.99E-05	4.94 (3.36-6.51)	7.70E-10	4.02E-07	0.82 (-0.78-2.42)	3.16E-01	1.00E+00

Gene	N Variants	logOR (CI) NFE	pvalue	corrected	logOR (CI) ALL	pvalue	corrected	logOR (CI) Swedish	pvalue	corrected
ADGRD2	2	2.84 (1.40-4.29)	1.14E-04	5.79E-02	3.36 (1.93-4.79)	4.15E-06	2.19E-03			
ANKRD20A4P	5	2.33 (1.43-3.24)	4.58E-07	2.32E-04	2.73 (1.83-3.63)	2.60E-09	1.37E-06	1.21 (0.13-2.29)	2.83E-02	1.00E+00
ATIC	2	2.42 (1.00-3.85)	8.51E-04	4.32E-01	3.03 (1.61-4.45)	2.82E-05	1.48E-02			
BAIAP3	2	4.68 (2.98-6.38)	7.03E-08	3.56E-05	5.48 (3.77-7.18)	2.81E-10	1.48E-07	1.90 (-0.07-3.86)	5.84E-02	1.00E+00
CAPN3	2	3.76 (2.24-5.28)	1.26E-06	6.39E-04	3.68 (2.24-5.13)	5.93E-07	3.13E-04	1.89 (-0.07-3.86)	5.86E-02	1.00E+00
CCDC66	9	1.49 (0.83-2.16)	1.00E-05	5.08E-03	1.83 (1.17-2.49)	5.49E-08	2.89E-05	0.80 (0.04-1.55)	3.91E-02	1.00E+00
CLEC4M	5	1.18 (0.29-2.07)	9.15E-03	1.00E+00	1.80 (0.91-2.69)	7.15E-05	3.77E-02	1.43 (0.31-2.56)	1.25E-02	1.00E+00
FAM227B	4	2.11 (1.10-3.11)	3.94E-05	2.00E-02	2.56 (1.56-3.56)	5.08E-07	2.68E-04	2.19 (0.69-3.69)	4.26E-03	1.00E+00
HHLA2	6	1.50 (0.68-2.32)	3.20E-04	1.62E-01	1.94 (1.12-2.75)	3.16E-06	1.66E-03	0.86 (-0.08-1.80)	7.24E-02	1.00E+00
HRNR	2	3.66 (2.15-5.17)	2.05E-06	1.04E-03	3.91 (2.44-5.37)	1.62E-07	8.53E-05			
ICAM3	2	4.96 (3.17-6.75)	5.60E-08	2.84E-05	5.25 (3.61-6.89)	3.70E-10	1.95E-07			
LRRC66	2	2.97 (1.52-4.42)	5.93E-05	3.00E-02	3.72 (2.28-5.17)	4.60E-07	2.42E-04			
MUC4	4	1.92 (0.92-2.91)	1.66E-04	8.40E-02	2.33 (1.33-3.32)	4.38E-06	2.31E-03	3.28 (1.09-5.47)	3.36E-03	1.00E+00
MYOM2	2	3.98 (2.43-5.53)	4.91E-07	2.49E-04	4.55 (3.03-6.08)	4.30E-09	2.27E-06			

OC90	2	3.50 (2.01-4.99)	4.38E-06	2.22E-03	4.09 (2.61-5.56)	5.56E-08	2.93E-05	2.59 (0.19-4.99)	3.47E-02	1.00E+00
OR3A1	3	2.34 (1.18-3.51)	7.93E-05	4.02E-02	2.99 (1.83-4.15)	4.43E-07	2.33E-04	1.61 (0.11-3.11)	3.55E-02	1.00E+00
OR52E8	3	3.21 (2.01-4.41)	1.54E-07	7.83E-05	3.87 (2.68-5.07)	2.06E-10	1.08E-07	1.61 (0.11-3.11)	3.55E-02	1.00E+00
OSGEPL1	5	1.34 (0.45-2.23)	3.18E-03	1.00E+00	1.88 (0.99-2.77)	3.61E-05	1.90E-02	2.13 (0.81-3.45)	1.58E-03	6.33E-01
PCDHAC1	2				6.86 (4.46-9.26)	2.18E-08	1.15E-05			
PIPOX	2	5.37 (3.41-7.33)	8.14E-08	4.13E-05	6.17 (4.20-8.13)	7.25E-10	3.82E-07			
PRPH	7	1.57 (0.81-2.32)	5.04E-05	2.55E-02	1.73 (0.98-2.49)	6.62E-06	3.49E-03	0.80 (-0.06-1.67)	6.79E-02	1.00E+00
RAB44	5	2.51 (1.60-3.42)	5.91E-08	3.00E-05	2.58 (1.68-3.47)	1.70E-08	8.96E-06	1.43 (0.31-2.56)	1.25E-02	1.00E+00
RSRP1	4	3.62 (2.56-4.69)	2.60E-11	1.32E-08	4.30 (3.24-5.35)	1.50E-15	7.82E-13	1.90 (0.51-3.28)	7.44E-03	1.00E+00
SCNN1A	2	2.48 (1.05-3.91)	6.66E-04	3.38E-01	2.82 (1.40-4.23)	9.44E-05	4.98E-02	1.20 (-0.50-2.90)	1.66E-01	1.00E+00
SHISAL2B	3	3.34 (2.13-4.55)	6.17E-08	3.13E-05	3.38 (2.21-4.55)	1.56E-08	8.24E-06	0.69 (-0.60-1.99)	2.96E-01	1.00E+00
SPATA4	3	2.89 (1.71-4.07)	1.69E-06	8.59E-04	3.04 (1.87-4.20)	3.00E-07	1.58E-04	1.90 (0.29-3.50)	2.04E-02	1.00E+00
SREK1IP1	2	6.03 (3.63-8.43)	8.78E-07	4.45E-04	5.43 (3.72-7.13)	4.07E-10	2.15E-07			
TEKT3	2	2.35 (0.93-3.77)	1.22E-03	6.17E-01	2.93 (1.51-4.35)	5.14E-05	2.71E-02	1.20 (-0.50-2.90)	1.66E-01	1.00E+00
TOGARAM2	5	1.70 (0.81-2.59)	1.96E-04	9.96E-02	1.95 (1.06-2.84)	1.79E-05	9.41E-03	1.21 (0.13-2.29)	2.83E-02	1.00E+00
TRPM4	4	2.22 (1.21-3.23)	1.57E-05	7.96E-03	2.68 (1.68-3.69)	1.49E-07	7.86E-05	1.68 (0.36-3.00)	1.27E-02	1.00E+00
VPS13C	2				5.47 (3.77-7.17)	2.78E-10	1.47E-07			
WFIKKN2	2				6.86 (4.46-9.27)	2.18E-08	1.15E-05			
ZNF599	18	1.01 (0.54-1.48)	2.28E-05	1.16E-02	1.26 (0.79-1.73)	1.27E-07	6.69E-05	0.73 (0.20-1.26)	7.34E-03	1.00E+00

Supplementary table 2d. Swedish replication cohort (JAGUAR). Genes with enrichment in missense variants in tinnitus patients (N=147) and controls (N=151) cohorts.Odds ratio (OR) were calculated for the allelic frequencies reported in Non-Finish European (NFE) from gnomAD, all individuals form gnomAD and Swegen. If no LoF variants was reported in NFE or Swegen cohorts, OR cannot be calculated and cells are empty. Multiple hypothesis testing correction for each pvalue have been addressed by False Discovery Rate (FDR) approach for the total number of genes and total number of variants for each comparison.

Gene	N Variants	logOR (CI) NFE	pvalue	pvalue corrected	logOR (CI) ALL	pvalue	pvalue corrected	logOR (CI) Swedish	pvalue	pvalue corrected
ACP3	2	3.79 (2.27-5.31)	1.06E-06	2.20E-03	4.49 (2.98-6.00)	5.56E-09	1.17E-05	1.23 (-0.47-2.93)	1.57E-01	1.00E+00
ALDH1B1	2	3.89 (2.36-5.43)	6.77E-07	1.41E-03	3.08 (1.66-4.50)	2.13E-05	4.49E-02	1.92 (-0.04-3.89)	5.49E-02	1.00E+00
ANKRD27	2	3.17 (1.70-4.63)	2.24E-05	4.66E-02	3.75 (2.30-5.21)	4.04E-07	8.53E-04	1.92 (-0.04-3.89)	5.49E-02	1.00E+00
ARAP1	7	1.77 (1.01-2.52)	4.24E-06	8.81E-03	2.30 (1.55-3.06)	1.84E-09	3.89E-06	1.23 (0.32-2.14)	8.08E-03	1.00E+00
ARHGEF17	4	2.59 (1.58-3.60)	5.06E-07	1.05E-03	3.29 (2.28-4.30)	1.64E-10	3.47E-07	1.70 (0.38-3.01)	1.15E-02	1.00E+00
ARIH1	3	2.39 (1.22-3.55)	5.89E-05	1.22E-01	2.65 (1.50-3.80)	6.67E-06	1.41E-02	3.03 (0.76-5.29)	8.88E-03	1.00E+00
ARMC7	2	4.99 (3.20-6.79)	4.80E-08	9.97E-05	4.59 (3.06-6.11)	3.54E-09	7.48E-06	1.92 (-0.04-3.89)	5.49E-02	1.00E+00
ARMCX4	3	3.85 (2.57-5.13)	3.85E-09	8.00E-06	4.21 (2.98-5.45)	2.59E-11	5.46E-08	1.41 (-0.02-2.85)	5.37E-02	1.00E+00
ARRDC5	3	2.62 (1.46-3.79)	1.09E-05	2.26E-02	3.13 (1.97-4.29)	1.21E-07	2.55E-04	1.41 (-0.02-2.84)	5.39E-02	1.00E+00
ATP1A4	7	2.22 (1.46-2.99)	1.21E-08	2.52E-05	2.45 (1.69-3.20)	2.58E-10	5.45E-07	1.48 (0.53-2.44)	2.34E-03	1.00E+00
B3GLCT	2				6.89 (4.49-9.29)	1.93E-08	4.07E-05			
BCAR1	2	2.45 (1.03-3.88)	7.48E-04	1.00E+00	3.13 (1.71-4.55)	1.63E-05	3.45E-02	1.92 (-0.04-3.89)	5.49E-02	1.00E+00
BMPER	3	2.22 (1.06-3.38)	1.73E-04	3.60E-01	2.87 (1.72-4.03)	1.08E-06	2.28E-03	1.63 (0.13-3.13)	3.27E-02	1.00E+00
BOD1L1	8	2.30 (1.59-3.01)	2.31E-10	4.81E-07	2.10 (1.40-2.80)	4.37E-09	9.22E-06	1.52 (0.62-2.41)	9.13E-04	1.00E+00
BRCA1	5	1.81 (0.92-2.70)	6.66E-05	1.38E-01	2.11 (1.22-3.00)	3.09E-06	6.52E-03	2.43 (1.00-3.87)	8.75E-04	1.00E+00
C19orf12	6	1.48 (0.66-2.29)	3.95E-04	8.21E-01	2.09 (1.28-2.91)	4.94E-07	1.04E-03	1.78 (0.68-2.88)	1.47E-03	1.00E+00
C7orf26	2	4.99 (3.20-6.79)	4.80E-08	9.98E-05	5.79 (4.00-7.58)	2.45E-10	5.18E-07	2.62 (0.21-5.02)	3.29E-02	1.00E+00
CAPN15	11	2.21 (1.60-2.81)	9.31E-13	1.94E-09	2.63 (2.03-3.23)	0.00E+00	2.39E-14	1.38 (0.63-2.12)	2.90E-04	5.14E-01
CCDC168	7	1.34 (0.59-2.10)	4.58E-04	9.51E-01	1.72 (0.97-2.47)	7.17E-06	1.51E-02	1.16 (0.26-2.06)	1.14E-02	1.00E+00
CCDC88B	2	4.01 (2.46-5.56)	4.11E-07	8.55E-04	4.81 (3.25-6.36)	1.27E-09	2.69E-06	1.92 (-0.04-3.88)	5.51E-02	1.00E+00
CD34	4	1.88 (0.88-2.88)	2.24E-04	4.65E-01	2.49 (1.50-3.49)	9.07E-07	1.92E-03	1.36 (0.13-2.59)	3.01E-02	1.00E+00
CEACAM20	5	1.41 (0.52-2.29)	1.93E-03	1.00E+00	2.00 (1.11-2.89)	9.80E-06	2.07E-02	0.82 (-0.19-1.84)	1.12E-01	1.00E+00
CEP131	2	4.14 (2.57-5.72)	2.48E-07	5.16E-04	4.69 (3.15-6.22)	2.12E-09	4.47E-06			
CHST4	2	5.40 (3.44-7.36)	7.07E-08	1.47E-04	5.79 (4.00-7.58)	2.45E-10	5.18E-07			

CLCN1	2	3.89 (2.36-5.43)	6.83E-07	1.42E-03	3.55 (2.11-4.99)	1.31E-06	2.77E-03	1.92 (-0.04-3.89)	5.49E-02	1.00E+00
CLIC2	5	1.89 (0.99-2.80)	3.72E-05	7.73E-02	2.06 (1.16-2.95)	6.36E-06	1.34E-02	1.24 (0.16-2.32)	2.49E-02	1.00E+00
CLSTN1	2	4.99 (3.20-6.78)	4.77E-08	9.91E-05	5.28 (3.63-6.92)	3.02E-10	6.39E-07			
CPA5	3	3.79 (2.55-5.04)	2.36E-09	4.91E-06	4.21 (2.99-5.42)	1.02E-11	2.15E-08	1.23 (-0.16-2.62)	8.28E-02	1.00E+00
CRAMP1	4	1.68 (0.68-2.68)	9.94E-04	1.00E+00	2.17 (1.17-3.16)	1.98E-05	4.19E-02	0.91 (-0.24-2.06)	1.20E-01	1.00E+00
CSMD2	4	3.08 (2.04-4.11)	5.26E-09	1.09E-05	2.21 (1.22-3.21)	1.34E-05	2.83E-02	1.52 (0.25-2.79)	1.89E-02	1.00E+00
CYB5R2	2	4.71 (3.00-6.41)	5.91E-08	1.23E-04	5.28 (3.64-6.92)	3.07E-10	6.49E-07			
DCAF8L1	5	2.14 (1.24-3.05)	3.57E-06	7.43E-03	2.56 (1.66-3.46)	2.52E-08	5.31E-05	1.59 (0.44-2.75)	6.78E-03	1.00E+00
DGLUCY	3	2.14 (0.98-3.30)	2.90E-04	6.03E-01	2.71 (1.56-3.87)	4.14E-06	8.74E-03	0.62 (-0.66-1.91)	3.41E-01	1.00E+00
DNAH11	3	2.16 (1.01-3.32)	2.45E-04	5.09E-01	2.49 (1.34-3.64)	2.17E-05	4.57E-02	1.23 (-0.16-2.62)	8.31E-02	1.00E+00
DOCK11	7	2.02 (1.25-2.78)	2.45E-07	5.10E-04	2.59 (1.83-3.35)	2.94E-11	6.20E-08	2.50 (1.26-3.73)	7.28E-05	1.29E-01
DTNB	5	2.05 (1.15-2.95)	7.61E-06	1.58E-02	2.68 (1.78-3.58)	4.79E-09	1.01E-05	1.34 (0.24-2.44)	1.68E-02	1.00E+00
EEA1	3	2.76 (1.59-3.94)	4.22E-06	8.77E-03	2.50 (1.35-3.65)	2.06E-05	4.35E-02	2.33 (0.54-4.13)	1.08E-02	1.00E+00
EIF2AK4	2	6.09 (3.69-8.49)	6.76E-07	1.40E-03	6.89 (4.48-9.29)	1.93E-08	4.07E-05			
ELK3	4	1.98 (0.98-2.98)	1.10E-04	2.29E-01	2.60 (1.60-3.60)	3.55E-07	7.49E-04	1.23 (0.03-2.44)	4.50E-02	1.00E+00
ENPP7	7	1.47 (0.72-2.22)	1.30E-04	2.70E-01	1.87 (1.12-2.62)	9.99E-07	2.11E-03	1.16 (0.26-2.06)	1.14E-02	1.00E+00
ENTPD7	3	6.50 (4.23-8.77)	1.88E-08	3.91E-05	5.10 (3.79-6.41)	2.51E-14	5.30E-11	1.41 (-0.02-2.85)	5.37E-02	1.00E+00
EPHX1	2	4.48 (2.84-6.12)	8.98E-08	1.87E-04	4.25 (2.76-5.73)	2.04E-08	4.32E-05	2.61 (0.21-5.02)	3.30E-02	1.00E+00
ERCC2	3	2.73 (1.56-3.91)	4.93E-06	1.03E-02	2.74 (1.59-3.89)	3.18E-06	6.72E-03			
ETV1	2	6.09 (3.69-8.50)	6.75E-07	1.40E-03	6.20 (4.23-8.16)	6.19E-10	1.31E-06			
EVPL	3	2.80 (1.62-3.97)	3.19E-06	6.64E-03	3.19 (2.03-4.35)	7.45E-08	1.57E-04	1.63 (0.13-3.13)	3.28E-02	1.00E+00
FAIM2	4	1.87 (0.87-2.87)	2.51E-04	5.22E-01	2.43 (1.43-3.43)	1.84E-06	3.90E-03	1.12 (-0.07-2.30)	6.48E-02	1.00E+00
FBXL8	2	2.99 (1.54-4.45)	5.30E-05	1.10E-01	3.39 (1.95-4.82)	3.57E-06	7.53E-03	1.92 (-0.04-3.89)	5.49E-02	1.00E+00
FGF4	4	1.62 (0.62-2.62)	1.47E-03	1.00E+00	2.22 (1.22-3.21)	1.28E-05	2.70E-02	1.37 (0.13-2.60)	2.99E-02	1.00E+00
FOXK2	4	4.39 (3.24-5.54)	7.02E-14	1.46E-10	3.17 (2.16-4.18)	7.68E-10	1.62E-06	1.01 (-0.16-2.18)	8.97E-02	1.00E+00
GABRA6	5	1.57 (0.67-2.46)	5.82E-04	1.00E+00	2.04 (1.14-2.93)	7.49E-06	1.58E-02	1.24 (0.16-2.32)	2.49E-02	1.00E+00
GMPPB	3	2.46 (1.29-3.62)	3.66E-05	7.61E-02	2.97 (1.81-4.13)	5.28E-07	1.11E-03	0.82 (-0.49-2.14)	2.18E-01	1.00E+00
GPR63	4	2.05 (1.05-3.06)	6.05E-05	1.26E-01	2.60 (1.60-3.60)	3.32E-07	7.02E-04	1.12 (-0.07-2.30)	6.48E-02	1.00E+00
GUCY2C	2	3.15 (1.69-4.61)	2.44E-05	5.08E-02	3.89 (2.43-5.35)	1.68E-07	3.55E-04	0.82 (-0.78-2.43)	3.15E-01	1.00E+00
H1-1	2	4.48 (2.84-6.12)	8.95E-08	1.86E-04	4.69 (3.15-6.22)	2.12E-09	4.47E-06			
HCRTR1	3	2.04 (0.89-3.20)	5.32E-04	1.00E+00	2.62 (1.47-3.78)	8.17E-06	1.73E-02	1.93 (0.32-3.53)	1.87E-02	1.00E+00

HEATR1	5	2.00 (1.10-2.89)	1.22E-05	2.53E-02	2.31 (1.42-3.20)	3.52E-07	7.43E-04	2.44 (1.00-3.87)	8.70E-04	1.00E+00
HS3ST6	6	1.91 (1.10-2.73)	4.31E-06	8.97E-03	1.93 (1.12-2.74)	3.06E-06	6.47E-03	1.23 (0.25-2.22)	1.42E-02	1.00E+00
IGFN1	7	4.07 (3.23-4.90)	0.00E+00	0.00E+00	2.55 (1.79-3.31)	4.81E-11	1.02E-07	1.69 (0.69-2.68)	9.04E-04	1.00E+00
ISLR	2	3.60 (2.10-5.10)	2.47E-06	5.13E-03	4.05 (2.58-5.52)	6.23E-08	1.32E-04	2.61 (0.21-5.02)	3.30E-02	1.00E+00
ISM2	2				5.79 (4.00-7.58)	2.48E-10	5.24E-07	2.62 (0.21-5.02)	3.29E-02	1.00E+00
JCAD	3	3.50 (2.28-4.72)	1.70E-08	3.53E-05	3.40 (2.23-4.57)	1.17E-08	2.47E-05	0.82 (-0.49-2.13)	2.19E-01	1.00E+00
KISS1R	2	4.70 (3.00-6.41)	5.98E-08	1.24E-04	4.58 (3.06-6.11)	3.60E-09	7.61E-06			
KL	6	2.26 (1.44-3.09)	7.94E-08	1.65E-04	2.40 (1.58-3.21)	8.95E-09	1.89E-05	1.78 (0.68-2.88)	1.47E-03	1.00E+00
KMT2D	2	3.25 (1.79-4.72)	1.38E-05	2.88E-02	3.39 (1.96-4.82)	3.41E-06	7.21E-03			
KMT5A	2	4.93 (3.14-6.72)	7.01E-08	1.46E-04	4.51 (2.99-6.03)	6.32E-09	1.34E-05			
KNDC1	7	1.14 (0.40-1.89)	2.75E-03	1.00E+00	1.65 (0.90-2.39)	1.60E-05	3.39E-02	1.23 (0.32-2.14)	8.08E-03	1.00E+00
LAMA5	9	1.70 (1.04-2.36)	5.12E-07	1.06E-03	2.27 (1.61-2.93)	1.81E-11	3.81E-08	1.08 (0.29-1.86)	7.14E-03	1.00E+00
LARGE2	4	1.86 (0.86-2.85)	2.64E-04	5.49E-01	2.42 (1.42-3.41)	1.91E-06	4.03E-03	1.11 (-0.07-2.29)	6.52E-02	1.00E+00
LONRF3	2	4.24 (2.59-5.88)	4.34E-07	9.02E-04	4.99 (3.34-6.63)	2.77E-09	5.84E-06			
LRIG1	6	1.92 (1.11-2.73)	3.82E-06	7.95E-03	2.05 (1.24-2.86)	6.91E-07	1.46E-03	1.07 (0.12-2.03)	2.81E-02	1.00E+00
LRP10	5	2.96 (2.04-3.88)	3.08E-10	6.40E-07	2.71 (1.82-3.61)	3.02E-09	6.37E-06	1.46 (0.34-2.58)	1.09E-02	1.00E+00
LRP8	6	2.93 (2.10-3.77)	6.72E-12	1.40E-08	2.42 (1.60-3.23)	5.96E-09	1.26E-05	3.72 (1.60-5.84)	5.81E-04	1.00E+00
LRWD1	2	4.30 (2.70-5.90)	1.50E-07	3.11E-04	4.81 (3.26-6.36)	1.30E-09	2.75E-06	0.67 (-0.91-2.24)	4.06E-01	1.00E+00
LTBP1	6	3.27 (2.42-4.13)	5.91E-14	1.23E-10	3.41 (2.58-4.25)	9.00E-16	1.88E-12	1.33 (0.32-2.33)	9.50E-03	1.00E+00
LUZP4	3	2.45 (1.27-3.62)	4.48E-05	9.32E-02	3.05 (1.88-4.22)	3.11E-07	6.56E-04			
LY75	18	1.56 (1.09-2.03)	8.62E-11	1.79E-07	1.93 (1.46-2.40)	9.00E-16	2.01E-12	2.26 (1.54-2.98)	6.34E-10	1.12E-06
MAF	2				6.84 (4.44-9.25)	2.39E-08	5.06E-05			
MAGEC1	7	2.88 (2.09-3.66)	5.78E-13	1.20E-09	2.99 (2.22-3.76)	1.94E-14	4.10E-11	1.68 (0.68-2.67)	9.25E-04	1.00E+00
MAP3K4	8	1.58 (0.88-2.29)	1.10E-05	2.29E-02	2.04 (1.33-2.74)	1.35E-08	2.86E-05	2.62 (1.42-3.83)	1.93E-05	3.43E-02
MBD5	6	1.66 (0.84-2.47)	6.49E-05	1.35E-01	1.99 (1.18-2.80)	1.54E-06	3.25E-03	0.88 (-0.05-1.82)	6.44E-02	1.00E+00
MCPH1	2	2.87 (1.43-4.32)	9.73E-05	2.02E-01	3.25 (1.83-4.68)	7.92E-06	1.67E-02			
MED12	3	3.26 (2.04-4.47)	1.65E-07	3.43E-04	3.94 (2.73-5.16)	1.99E-10	4.21E-07	2.33 (0.54-4.13)	1.08E-02	1.00E+00
MGA	2	5.40 (3.43-7.36)	7.02E-08	1.46E-04	6.19 (4.23-8.15)	6.14E-10	1.30E-06			
MMP17	13	1.66 (1.11-2.22)	4.00E-09	8.32E-06	2.12 (1.57-2.68)	4.34E-14	9.18E-11	2.19 (1.36-3.01)	2.11E-07	3.74E-04
MPHOSPH9	6	2.39 (1.56-3.22)	1.50E-08	3.12E-05	2.51 (1.69-3.33)	1.80E-09	3.80E-06	2.34 (1.07-3.61)	3.06E-04	5.43E-01
MUC16	16	0.79 (0.30-1.29)	1.65E-03	1.00E+00	1.23 (0.73-1.72)	1.09E-06	2.31E-03	0.74 (0.18-1.30)	9.64E-03	1.00E+00

NAGA	4	1.76 (0.76-2.76)	5.34E-04	1.00E+00	2.28 (1.29-3.28)	6.59E-06	1.39E-02	2.21 (0.71-3.71)	3.85E-03	1.00E+00
NAV2	2	2.54 (1.11-3.97)	5.08E-04	1.00E+00	3.28 (1.85-4.71)	6.79E-06	1.43E-02			
NBEAL2	6	2.42 (1.60-3.25)	8.43E-09	1.75E-05	2.87 (2.05-3.69)	6.04E-12	1.27E-08	0.94 (0.00-1.89)	4.99E-02	1.00E+00
NINL	2	4.30 (2.69-5.90)	1.48E-07	3.07E-04	4.49 (2.98-6.00)	5.55E-09	1.17E-05	2.61 (0.21-5.02)	3.30E-02	1.00E+00
NKD2	4	1.93 (0.93-2.93)	1.50E-04	3.11E-01	2.37 (1.38-3.37)	2.92E-06	6.16E-03	0.82 (-0.31-1.96)	1.55E-01	1.00E+00
NLRP3	3	2.07 (0.91-3.23)	4.59E-04	9.54E-01	2.71 (1.56-3.87)	4.13E-06	8.72E-03	1.64 (0.14-3.14)	3.26E-02	1.00E+00
NOC2L	3	2.41 (1.24-3.57)	5.21E-05	1.08E-01	2.80 (1.64-3.95)	2.12E-06	4.47E-03			
NRDE2	4	3.05 (2.02-4.09)	6.65E-09	1.38E-05	2.23 (1.24-3.23)	1.13E-05	2.38E-02	1.93 (0.54-3.32)	6.58E-03	1.00E+00
NTRK1	3	5.40 (3.79-7.00)	4.37E-11	9.08E-08	5.21 (3.87-6.54)	1.83E-14	3.86E-11	1.64 (0.14-3.14)	3.26E-02	1.00E+00
OR4D5	5	2.61 (1.71-3.52)	1.60E-08	3.32E-05	2.60 (1.70-3.49)	1.18E-08	2.48E-05	1.92 (0.68-3.17)	2.40E-03	1.00E+00
OR8U1	5	1.58 (0.69-2.47)	5.25E-04	1.00E+00	2.00 (1.11-2.89)	1.05E-05	2.22E-02	1.14 (0.08-2.20)	3.57E-02	1.00E+00
PALD1	3	5.81 (4.01-7.60)	2.17E-10	4.52E-07	6.20 (4.59-7.80)	3.64E-14	7.70E-11	2.33 (0.54-4.13)	1.08E-02	1.00E+00
PAPPA	4	1.96 (0.96-2.96)	1.25E-04	2.59E-01	2.49 (1.49-3.49)	1.04E-06	2.19E-03	1.52 (0.25-2.79)	1.89E-02	1.00E+00
PAPSS1	4	1.92 (0.93-2.92)	1.59E-04	3.30E-01	2.36 (1.37-3.35)	3.24E-06	6.84E-03	1.23 (0.03-2.43)	4.53E-02	1.00E+00
PCBP1	6	4.64 (3.66-5.61)	0.00E+00	0.00E+00	2.98 (2.15-3.80)	1.43E-12	3.03E-09	2.63 (1.24-4.02)	2.12E-04	3.76E-01
PCDHA1	5	2.84 (1.93-3.76)	1.22E-09	2.54E-06	2.11 (1.22-3.00)	3.37E-06	7.11E-03	2.44 (1.01-3.88)	8.56E-04	1.00E+00
PCK1	10	1.53 (0.90-2.16)	2.05E-06	4.27E-03	2.14 (1.51-2.77)	2.41E-11	5.08E-08	0.89 (0.17-1.62)	1.57E-02	1.00E+00
PEG3	4	2.89 (1.87-3.92)	2.94E-08	6.11E-05	2.79 (1.79-3.78)	4.68E-08	9.89E-05	1.23 (0.03-2.43)	4.53E-02	1.00E+00
PELP1	10	1.88 (1.25-2.52)	5.05E-09	1.05E-05	1.76 (1.13-2.38)	3.88E-08	8.20E-05	2.15 (1.22-3.08)	6.23E-06	1.10E-02
PKNOX2	3	2.81 (1.63-3.99)	2.96E-06	6.15E-03	3.19 (2.02-4.35)	8.23E-08	1.74E-04	1.64 (0.14-3.14)	3.26E-02	1.00E+00
PLXNB1	3	2.57 (1.40-3.74)	1.69E-05	3.52E-02	3.08 (1.92-4.24)	2.08E-07	4.40E-04	0.94 (-0.39-2.28)	1.66E-01	1.00E+00
POLH	6	1.95 (1.13-2.76)	3.03E-06	6.30E-03	2.26 (1.44-3.07)	5.11E-08	1.08E-04	1.32 (0.32-2.32)	9.64E-03	1.00E+00
PRODH2	2	6.09 (3.69-8.50)	6.76E-07	1.40E-03	6.20 (4.23-8.16)	6.20E-10	1.31E-06			
PRRG3	3	6.24 (3.98-8.51)	6.66E-08	1.38E-04	6.29 (4.49-8.08)	6.32E-12	1.34E-08			
PRRT3	3	4.89 (3.45-6.32)	2.39E-11	4.97E-08	4.52 (3.28-5.76)	7.55E-13	1.59E-09			
PTCH1	4	3.46 (2.41-4.51)	1.25E-10	2.60E-07	2.39 (1.39-3.39)	2.66E-06	5.61E-03	1.52 (0.25-2.79)	1.89E-02	1.00E+00
PTPRU	2	5.40 (3.43-7.36)	7.03E-08	1.46E-04	5.09 (3.49-6.70)	4.65E-10	9.81E-07			
PURA	5	3.20 (2.27-4.13)	1.43E-11	2.97E-08	3.24 (2.34-4.15)	2.08E-12	4.39E-09	3.54 (1.39-5.68)	1.26E-03	1.00E+00
PYGM	6	1.49 (0.68-2.30)	3.30E-04	6.87E-01	1.88 (1.08-2.69)	5.05E-06	1.07E-02	0.88 (-0.05-1.82)	6.44E-02	1.00E+00
QRSL1	4	2.13 (1.12-3.13)	3.35E-05	6.97E-02	2.51 (1.51-3.51)	8.71E-07	1.84E-03	1.12 (-0.07-2.30)	6.48E-02	1.00E+00
RAB12	2	5.38 (3.42-7.34)	7.90E-08	1.64E-04	5.48 (3.78-7.18)	2.72E-10	5.74E-07			
RAB38	5	1.49 (0.60-2.38)	1.05E-03	1.00E+00	1.98 (1.09-2.87)	1.22E-05	2.57E-02	1.34 (0.24-2.43)	1.70E-02	1.00E+00
RAB3D	3	2.61 (1.44-3.78)	1.27E-05	2.65E-02	2.62 (1.47-3.78)	8.16E-06	1.72E-02	3.03 (0.76-5.29)	8.88E-03	1.00E+00

RALY	2	3.70 (2.18-5.22)	1.88E-06	3.91E-03	3.79 (2.34-5.25)	3.24E-07	6.84E-04	1.51 (-0.28-3.31)	9.75E-02	1.00E+00
RFX7	6	1.68 (0.86-2.49)	5.35E-05	1.11E-01	1.91 (1.10-2.72)	3.89E-06	8.22E-03	2.11 (0.92-3.30)	5.15E-04	9.12E-01
RP1L1	4	4.49 (3.32-5.65)	4.32E-14	8.98E-11	4.95 (3.83-6.07)	0.00E+00	8.00E-15	1.52 (0.25-2.79)	1.89E-02	1.00E+00
SCAF1	2	2.87 (1.42-4.31)	9.79E-05	2.04E-01	3.51 (2.08-4.95)	1.59E-06	3.35E-03	1.51 (-0.28-3.31)	9.75E-02	1.00E+00
SELP	2	2.59 (1.16-4.03)	3.85E-04	8.01E-01	3.27 (1.85-4.70)	6.92E-06	1.46E-02	2.62 (0.21-5.02)	3.29E-02	1.00E+00
SELPLG	5	3.23 (2.30-4.17)	1.06E-11	2.19E-08	2.78 (1.88-3.68)	1.35E-09	2.86E-06	2.16 (0.84-3.48)	1.38E-03	1.00E+00
SH3BP2	2	3.89 (2.36-5.42)	6.73E-07	1.40E-03	3.10 (1.68-4.52)	1.88E-05	3.98E-02			
SLC12A9	3	2.42 (1.26-3.59)	4.65E-05	9.66E-02	2.68 (1.53-3.84)	5.26E-06	1.11E-02	0.82 (-0.49-2.14)	2.18E-01	1.00E+00
SLC15A5	3	3.36 (2.16-4.57)	4.87E-08	1.01E-04	2.51 (1.36-3.66)	1.95E-05	4.12E-02	1.64 (0.14-3.14)	3.26E-02	1.00E+00
SLC24A2	2	6.09 (3.69-8.49)	6.79E-07	1.41E-03	5.79 (3.99-7.58)	2.50E-10	5.28E-07			
SLC26A1	6	3.93 (3.04-4.82)	0.00E+00	1.00E-14	3.94 (3.10-4.79)	0.00E+00	1.00E-16	3.02 (1.42-4.62)	2.17E-04	3.85E-01
SLC30A4	4	2.63 (1.62-3.65)	3.90E-07	8.11E-04	3.03 (2.03-4.04)	3.50E-09	7.40E-06	1.71 (0.38-3.03)	1.14E-02	1.00E+00
SLC45A4	5	1.89 (0.99-2.78)	3.77E-05	7.85E-02	2.15 (1.26-3.04)	2.28E-06	4.81E-03	1.46 (0.34-2.58)	1.09E-02	1.00E+00
SLC4A4	5	1.93 (1.03-2.83)	2.55E-05	5.29E-02	2.48 (1.58-3.37)	5.77E-08	1.22E-04	1.24 (0.16-2.32)	2.49E-02	1.00E+00
SNAI3	2	3.61 (2.11-5.11)	2.50E-06	5.20E-03	4.32 (2.83-5.82)	1.39E-08	2.93E-05	2.62 (0.21-5.02)	3.29E-02	1.00E+00
SORCS2	5	5.19 (4.00-6.38)	0.00E+00	2.97E-14	5.47 (4.39-6.55)	0.00E+00	0.00E+00			
SOX9	2	3.68 (2.18-5.19)	1.71E-06	3.55E-03	3.19 (1.76-4.61)	1.13E-05	2.38E-02			
SPNS3	5	1.74 (0.85-2.63)	1.30E-04	2.70E-01	2.26 (1.38-3.15)	5.91E-07	1.25E-03	1.74 (0.55-2.93)	4.12E-03	1.00E+00
SPOPL	7	1.50 (0.74-2.25)	1.08E-04	2.25E-01	1.90 (1.15-2.66)	7.72E-07	1.63E-03	1.69 (0.69-2.68)	9.04E-04	1.00E+00
STARD9	3	2.43 (1.27-3.60)	4.10E-05	8.51E-02	3.07 (1.91-4.23)	2.03E-07	4.30E-04	0.94 (-0.39-2.27)	1.66E-01	1.00E+00
SYNE3	5	1.47 (0.58-2.36)	1.25E-03	1.00E+00	1.93 (1.04-2.82)	2.08E-05	4.40E-02	1.46 (0.34-2.58)	1.09E-02	1.00E+00
TAF6	4	3.53 (2.47-4.59)	6.15E-11	1.28E-07	2.73 (1.73-3.73)	9.35E-08	1.98E-04	1.71 (0.38-3.03)	1.14E-02	1.00E+00
TCP11L1	2	3.05 (1.59-4.50)	4.04E-05	8.40E-02	3.46 (2.02-4.89)	2.35E-06	4.96E-03	2.62 (0.21-5.02)	3.29E-02	1.00E+00
THBS1	2	3.61 (2.11-5.11)	2.50E-06	5.19E-03	3.84 (2.39-5.30)	2.24E-07	4.73E-04	2.62 (0.21-5.02)	3.29E-02	1.00E+00
TMEM38A	2	3.89 (2.36-5.43)	6.76E-07	1.40E-03	4.59 (3.06-6.11)	3.54E-09	7.47E-06			
TMIE	4	2.65 (1.63-3.67)	3.36E-07	6.99E-04	3.06 (2.05-4.06)	2.76E-09	5.83E-06	1.12 (-0.07-2.30)	6.48E-02	1.00E+00
TRIM35	7	2.14 (1.38-2.91)	3.73E-08	7.76E-05	1.92 (1.16-2.67)	6.56E-07	1.39E-03	1.48 (0.53-2.44)	2.34E-03	1.00E+00
TRIM42	2	3.05 (1.59-4.50)	4.06E-05	8.44E-02	3.75 (2.30-5.20)	3.88E-07	8.19E-04	1.52 (-0.28-3.31)	9.73E-02	1.00E+00
TRIM55	5	2.59 (1.68-3.50)	2.49E-08	5.19E-05	3.06 (2.16-3.96)	2.99E-11	6.31E-08	1.24 (0.16-2.32)	2.49E-02	1.00E+00
TRPM2	4	1.78 (0.78-2.78)	4.87E-04	1.00E+00	2.15 (1.15-3.14)	2.30E-05	4.87E-02	1.71 (0.38-3.03)	1.14E-02	1.00E+00
TTYH2	2	2.53 (1.11-3.96)	5.05E-04	1.00E+00	3.17 (1.75-4.59)	1.22E-05	2.59E-02	1.92 (-0.04-3.88)	5.51E-02	1.00E+00
TUBA8	2	3.15 (1.69-4.61)	2.44E-05	5.08E-02	3.75 (2.30-5.20)	3.89E-07	8.22E-04	1.92 (-0.04-3.89)	5.49E-02	1.00E+00
UGT3A2	2	2.57 (1.13-4.00)	4.41E-04	9.18E-01	3.23 (1.80-4.65)	9.25E-06	1.95E-02	1.52 (-0.28-3.31)	9.73E-02	1.00E+00

UMODL1	5	1.57 (0.68-2.46)	5.24E-04	1.00E+00	1.92 (1.03-2.81)	2.18E-05	4.61E-02	0.89 (-0.13-1.92)	8.78E-02	1.00E+00
VAV2	5	2.46 (1.55-3.36)	9.86E-08	2.05E-04	2.65 (1.75-3.54)	6.15E-09	1.30E-05	1.13 (0.07-2.20)	3.60E-02	1.00E+00
ZFHX3	9	1.91 (1.24-2.57)	1.83E-08	3.81E-05	2.32 (1.65-2.98)	6.99E-12	1.48E-08	1.48 (0.64-2.32)	5.48E-04	9.72E-01
ZIC2	2	4.29 (2.68-5.89)	1.57E-07	3.26E-04	4.67 (3.14-6.21)	2.35E-09	4.97E-06			
ZNF214	8	1.81 (1.10-2.52)	5.15E-07	1.07E-03	1.87 (1.16-2.57)	1.93E-07	4.08E-04	2.62 (1.42-3.83)	1.93E-05	3.43E-02

Gene	N Variants	logOR (CI) NFE	pvalue	pvalue	logOR (CI) ALL	pvalue	pvalue	logOR (CI) Swedish	pvalue	pvalue
ABCA7	3	1.96 (0.80-3.11)	8.79E-04	1.00E+00	2.52 (1.37-3.67)	1.72E-05	3.81E-02	1.38 (-0.05-2.82)	5.86E-02	1.00E+00
ACTL8	7	1.27 (0.52-2.02)	9.44E-04	1.00E+00	1.68 (0.94-2.43)	1.05E-05	2.32E-02	0.85 (-0.02-1.71)	5.49E-02	1.00E+00
ADGRD2	3	2.41 (1.25-3.58)	5.01E-05	1.08E-01	2.89 (1.73-4.05)	1.02E-06	2.25E-03			
ADGRG4	2	2.98 (1.51-4.45)	6.88E-05	1.49E-01	3.51 (2.06-4.97)	2.19E-06	4.85E-03			
AGFG2	3	2.45 (1.28-3.61)	3.95E-05	8.55E-02	3.14 (1.98-4.31)	1.21E-07	2.69E-04	2.30 (0.51-4.10)	1.18E-02	1.00E+00
AGL	5	2.25 (1.35-3.16)	1.00E-06	2.16E-03	2.72 (1.83-3.62)	2.70E-09	5.99E-06	1.11 (0.05-2.18)	4.03E-02	1.00E+00
AHNAK2	4	1.86 (0.86-2.86)	2.64E-04	5.70E-01	2.28 (1.28-3.27)	7.09E-06	1.57E-02	1.20 (0.00-2.41)	5.03E-02	1.00E+00
ANKRD27	2	3.14 (1.68-4.61)	2.63E-05	5.69E-02	3.73 (2.28-5.18)	4.88E-07	1.08E-03	1.90 (-0.07-3.86)	5.84E-02	1.00E+00
AP4M1	2	6.07 (3.66-8.47)	7.55E-07	1.63E-03	6.86 (4.46-9.27)	2.18E-08	4.83E-05	2.59 (0.19-4.99)	3.47E-02	1.00E+00
APOE	5	1.93 (1.03-2.82)	2.60E-05	5.62E-02	2.16 (1.26-3.05)	2.16E-06	4.78E-03	1.11 (0.05-2.18)	4.03E-02	1.00E+00
ARHGEF40	2	3.17 (1.71-4.64)	2.17E-05	4.70E-02	3.87 (2.41-5.32)	2.02E-07	4.47E-04	2.59 (0.19-4.99)	3.47E-02	1.00E+00
ARID1A	2	5.36 (3.40-7.33)	8.53E-08	1.85E-04	5.75 (3.96-7.55)	3.15E-10	6.96E-07	1.90 (-0.07-3.86)	5.84E-02	1.00E+00
ARRDC5	4	2.88 (1.86-3.91)	3.18E-08	6.87E-05	3.39 (2.38-4.41)	4.98E-11	1.10E-07	1.67 (0.35-2.99)	1.29E-02	1.00E+00
ATP6V1B1	2	3.02 (1.57-4.47)	4.73E-05	1.02E-01	3.57 (2.13-5.01)	1.23E-06	2.72E-03	1.90 (-0.07-3.86)	5.84E-02	1.00E+00
BCR	5	1.39 (0.50-2.28)	2.14E-03	1.00E+00	1.96 (1.07-2.84)	1.46E-05	3.23E-02	0.73 (-0.28-1.74)	1.55E-01	1.00E+00
BPIFB3	2	6.06 (3.66-8.47)	7.57E-07	1.64E-03	6.86 (4.46-9.26)	2.19E-08	4.85E-05	1.90 (-0.07-3.86)	5.84E-02	1.00E+00
BRCC3	4	2.69 (1.66-3.72)	2.92E-07	6.32E-04	2.90 (1.89-3.91)	1.87E-08	4.14E-05	2.60 (0.89-4.30)	2.79E-03	1.00E+00
BSN	11	1.19 (0.59-1.79)	9.30E-05	2.01E-01	1.63 (1.03-2.23)	8.61E-08	1.90E-04	1.73 (0.93-2.54)	2.52E-05	4.66E-02
C1orf68	2	2.81 (1.36-4.25)	1.38E-04	2.98E-01	3.30 (1.88-4.73)	5.85E-06	1.30E-02	1.20 (-0.50-2.90)	1.66E-01	1.00E+00
C3	2	3.67 (2.16-5.18)	1.97E-06	4.27E-03	3.40 (1.96-4.83)	3.41E-06	7.55E-03	1.90 (-0.07-3.86)	5.84E-02	1.00E+00
C4orf19	4	1.73 (0.73-2.73)	6.80E-04	1.00E+00	2.39 (1.39-3.39)	2.65E-06	5.87E-03	1.90 (0.51-3.29)	7.38E-03	1.00E+00
CABP1	2				6.13 (4.17-8.10)	9.10E-10	2.01E-06			
CALB1	4	2.98 (1.95-4.01)	1.43E-08	3.08E-05	3.40 (2.39-4.42)	5.37E-11	1.19E-07	1.09 (-0.10-2.27)	7.16E-02	1.00E+00
CCDC191	9	1.39 (0.72-2.05)	4.16E-05	9.00E-02	1.87 (1.21-2.53)	2.90E-08	6.42E-05	1.46 (0.62-2.30)	6.82E-04	1.00E+00

CDH3	3	3.70 (2.46-4.94)	4.70E-09	1.02E-05	3.77 (2.59-4.96)	4.67E-10	1.03E-06	1.20 (-0.19-2.60)	8.98E-02	1.00E+00
CDKN1C	2	3.05 (1.59-4.51)	4.31E-05	9.32E-02	3.36 (1.92-4.79)	4.63E-06	1.02E-02	1.49 (-0.30-3.28)	1.03E-01	1.00E+00
CEACAM20	5	1.38 (0.49-2.27)	2.35E-03	1.00E+00	1.97 (1.09-2.86)	1.29E-05	2.85E-02	0.80 (-0.22-1.81)	1.24E-01	1.00E+00
COL14A1	2	6.06 (3.66-8.47)	7.56E-07	1.64E-03	4.38 (2.88-5.88)	1.10E-08	2.44E-05	1.90 (-0.07-3.86)	5.84E-02	1.00E+00
CRELD2	2	3.22 (1.75-4.69)	1.73E-05	3.74E-02	3.89 (2.43-5.35)	1.83E-07	4.04E-04	0.80 (-0.81-2.40)	3.31E-01	1.00E+00
CTBS	2	5.37 (3.41-7.33)	8.23E-08	1.78E-04	5.47 (3.77-7.18)	2.84E-10	6.29E-07			
CTNND2	3	5.05 (3.55-6.55)	4.20E-11	9.09E-08	4.74 (3.47-6.01)	2.54E-13	5.62E-10			
CTR9	3	2.35 (1.18-3.51)	7.85E-05	1.70E-01	2.67 (1.51-3.82)	6.00E-06	1.33E-02	0.69 (-0.60-1.99)	2.96E-01	1.00E+00
CUBN	3	2.71 (1.54-3.89)	6.19E-06	1.34E-02	3.32 (2.15-4.49)	2.65E-08	5.86E-05	1.90 (0.29-3.50)	2.04E-02	1.00E+00
CXCR5	3	3.48 (2.26-4.70)	2.24E-08	4.84E-05	4.09 (2.89-5.30)	2.87E-11	6.35E-08	1.39 (-0.05-2.82)	5.84E-02	1.00E+00
DAAM2	5	1.87 (0.97-2.77)	4.35E-05	9.41E-02	2.15 (1.26-3.04)	2.34E-06	5.18E-03	1.43 (0.31-2.56)	1.25E-02	1.00E+00
DHRS2	3	2.47 (1.30-3.63)	3.49E-05	7.56E-02	2.95 (1.79-4.11)	6.02E-07	1.33E-03	0.80 (-0.51-2.11)	2.34E-01	1.00E+00
DTNB	4	1.80 (0.80-2.80)	4.21E-04	9.10E-01	2.42 (1.43-3.42)	1.91E-06	4.23E-03	1.09 (-0.10-2.27)	7.16E-02	1.00E+00
EML6	6	1.52 (0.71-2.33)	2.40E-04	5.19E-01	1.83 (1.02-2.64)	8.77E-06	1.94E-02	1.05 (0.09-2.01)	3.23E-02	1.00E+00
EP400	9	1.28 (0.62-1.94)	1.52E-04	3.29E-01	1.56 (0.91-2.22)	3.26E-06	7.22E-03	1.39 (0.56-2.21)	1.05E-03	1.00E+00
EVPL	6	1.96 (1.14-2.78)	2.55E-06	5.52E-03	2.42 (1.60-3.23)	5.50E-09	1.22E-05	1.29 (0.29-2.29)	1.13E-02	1.00E+00
FAIM2	4	1.84 (0.84-2.84)	3.08E-04	6.67E-01	2.40 (1.40-3.40)	2.39E-06	5.29E-03	1.09 (-0.10-2.27)	7.16E-02	1.00E+00
FAT1	2	2.77 (1.33-4.20)	1.65E-04	3.56E-01	3.36 (1.93-4.79)	4.04E-06	8.94E-03	2.59 (0.18-4.99)	3.48E-02	1.00E+00
FAT2	7	1.53 (0.78-2.28)	6.82E-05	1.48E-01	1.99 (1.24-2.74)	2.00E-07	4.43E-04	1.90 (0.85-2.95)	3.98E-04	7.37E-01
FCRL6	2	3.43 (1.94-4.91)	6.23E-06	1.35E-02	3.34 (1.90-4.77)	4.90E-06	1.08E-02	0.39 (-1.15-1.93)	6.20E-01	1.00E+00
FLCN	5	1.62 (0.73-2.52)	3.77E-04	8.15E-01	1.97 (1.08-2.86)	1.51E-05	3.34E-02	1.72 (0.53-2.91)	4.67E-03	1.00E+00
GABBR2	2				6.81 (4.41-9.22)	2.74E-08	6.07E-05			
GFI1B	4	1.67 (0.67-2.67)	1.06E-03	1.00E+00	2.23 (1.23-3.22)	1.16E-05	2.57E-02	1.34 (0.11-2.58)	3.33E-02	1.00E+00
GMPR	5	2.01 (1.11-2.90)	1.23E-05	2.65E-02	2.54 (1.64-3.43)	2.71E-08	6.00E-05	2.42 (0.98-3.85)	9.77E-04	1.00E+00
GPKOW	4	5.42 (3.92-6.92)	1.44E-12	3.12E-09	5.88 (4.49-7.27)	1.00E-16	2.54E-13	3.29 (1.09-5.48)	3.31E-03	1.00E+00
HELZ2	10	2.09 (1.46-2.72)	9.96E-11	2.15E-07	2.49 (1.86-3.12)	8.60E-15	1.91E-11	1.80 (0.94-2.66)	3.91E-05	7.23E-02
HIPK4	3	2.60 (1.43-3.77)	1.35E-05	2.93E-02	3.14 (1.98-4.31)	1.21E-07	2.67E-04	1.61 (0.11-3.11)	3.55E-02	1.00E+00
HOXA11	2	2.84 (1.40-4.28)	1.13E-04	2.46E-01	3.07 (1.65-4.49)	2.21E-05	4.90E-02	1.89 (-0.07-3.86)	5.86E-02	1.00E+00
HSPA12B	2	2.63 (1.20-4.06)	3.26E-04	7.05E-01	3.12 (1.70-4.55)	1.68E-05	3.73E-02	0.64 (-0.94-2.22)	4.26E-01	1.00E+00
IDH2	6	2.18 (1.36-3.00)	2.02E-07	4.36E-04	2.53 (1.71-3.34)	1.23E-09	2.72E-06	1.61 (0.55-2.67)	2.95E-03	1.00E+00
IFNA14	2	2.73 (1.29-4.17)	1.98E-04	4.29E-01	3.37 (1.93-4.80)	4.09E-06	9.05E-03	1.20 (-0.50-2.90)	1.66E-01	1.00E+00
IRGC	3				7.27 (5.00-9.54)	3.20E-10	7.07E-07			
IVD	4	1.97 (0.96-2.97)	1.20E-04	2.60E-01	2.69 (1.68-3.69)	1.46E-07	3.24E-04	0.98 (-0.18-2.15)	9.86E-02	1.00E+00

KCNH6	3	2.83 (1.65-4.01)	2.49E-06	5.40E-03	3.14 (1.98-4.30)	1.19E-07	2.62E-04	1.38 (-0.05-2.82)	5.86E-02	1.00E+00
KIAA1549	4	4.13 (3.01-5.24)	4.52E-13	9.77E-10	4.79 (3.69-5.89)	0.00E+00	3.48E-14	2.60 (0.89-4.30)	2.79E-03	1.00E+00
KNDC1	7	1.12 (0.37-1.87)	3.44E-03	1.00E+00	1.62 (0.87-2.37)	2.19E-05	4.85E-02	1.21 (0.29-2.12)	9.53E-03	1.00E+00
LAMA5	12	1.31 (0.73-1.88)	7.90E-06	1.71E-02	1.84 (1.27-2.41)	2.81E-10	6.22E-07	1.25 (0.55-1.95)	4.88E-04	9.04E-01
LLGL2	6	1.50 (0.69-2.31)	2.91E-04	6.30E-01	1.77 (0.96-2.58)	1.88E-05	4.16E-02	1.61 (0.55-2.67)	2.95E-03	1.00E+00
LRP10	4	2.70 (1.68-3.72)	2.03E-07	4.38E-04	2.46 (1.46-3.46)	1.35E-06	2.99E-03	1.21 (0.00-2.41)	4.99E-02	1.00E+00
LRP8	3	4.16 (2.87-5.46)	2.99E-10	6.47E-07	4.08 (2.88-5.29)	3.16E-11	7.00E-08			
LRTM1	3	3.34 (2.13-4.54)	6.26E-08	1.35E-04	3.74 (2.56-4.93)	6.26E-10	1.39E-06	1.20 (-0.19-2.60)	8.98E-02	1.00E+00
MAP3K4	5	4.14 (3.14-5.14)	5.00E-16	1.03E-12	4.48 (3.52-5.43)	0.00E+00	1.00E-16			
MCM2	2	3.23 (1.76-4.70)	1.64E-05	3.54E-02	3.97 (2.51-5.44)	1.08E-07	2.40E-04	2.59 (0.19-4.99)	3.47E-02	1.00E+00
MDN1	7	1.83 (1.07-2.58)	2.14E-06	4.62E-03	1.85 (1.10-2.60)	1.36E-06	3.02E-03	1.14 (0.23-2.04)	1.35E-02	1.00E+00
MECOM	3	3.18 (1.98-4.38)	2.03E-07	4.39E-04	3.74 (2.56-4.93)	6.10E-10	1.35E-06	2.30 (0.51-4.10)	1.18E-02	1.00E+00
MEGF8	3	2.88 (1.70-4.07)	1.67E-06	3.61E-03	3.53 (2.35-4.70)	3.86E-09	8.53E-06	2.99 (0.73-5.26)	9.57E-03	1.00E+00
MMP17	14	1.79 (1.26-2.33)	4.64E-11	1.00E-07	2.25 (1.72-2.78)	1.00E-16	2.55E-13	2.46 (1.59-3.33)	3.11E-08	5.76E-05
MTUS2	4	1.66 (0.66-2.66)	1.11E-03	1.00E+00	2.27 (1.27-3.27)	7.92E-06	1.75E-02	0.98 (-0.18-2.15)	9.86E-02	1.00E+00
MUC12	6	1.32 (0.51-2.13)	1.39E-03	1.00E+00	1.79 (0.98-2.59)	1.45E-05	3.22E-02	1.12 (0.15-2.09)	2.34E-02	1.00E+00
MUC4	11	1.44 (0.83-2.05)	3.51E-06	7.60E-03	1.54 (0.94-2.14)	5.15E-07	1.14E-03	1.90 (1.06-2.74)	8.99E-06	1.66E-02
MYBBP1A	3	2.59 (1.42-3.76)	1.37E-05	2.96E-02	2.88 (1.72-4.03)	1.06E-06	2.34E-03	1.38 (-0.05-2.82)	5.86E-02	1.00E+00
NFXL1	5	1.43 (0.54-2.32)	1.68E-03	1.00E+00	1.98 (1.09-2.87)	1.30E-05	2.88E-02	1.43 (0.31-2.56)	1.25E-02	1.00E+00
NKD1	2	4.45 (2.81-6.10)	1.09E-07	2.35E-04	4.66 (3.13-6.20)	2.68E-09	5.93E-06			
NOL6	2	4.12 (2.54-5.69)	2.95E-07	6.39E-04	3.56 (2.12-5.00)	1.21E-06	2.68E-03	2.59 (0.18-4.99)	3.48E-02	1.00E+00
NPAS3	3	3.22 (2.01-4.42)	1.52E-07	3.30E-04	2.64 (1.48-3.79)	7.51E-06	1.66E-02	1.20 (-0.19-2.60)	8.98E-02	1.00E+00
NPHS1	5	1.43 (0.54-2.33)	1.63E-03	1.00E+00	2.03 (1.14-2.92)	7.69E-06	1.70E-02	1.21 (0.13-2.29)	2.83E-02	1.00E+00
NRXN2	5	1.61 (0.71-2.50)	4.24E-04	9.16E-01	2.11 (1.22-3.00)	3.39E-06	7.50E-03	1.11 (0.05-2.18)	4.03E-02	1.00E+00
NUP210	2	3.43 (1.94-4.91)	6.24E-06	1.35E-02	3.68 (2.24-5.13)	6.04E-07	1.34E-03	0.98 (-0.67-2.62)	2.44E-01	1.00E+00
OR2H2	4	2.43 (1.42-3.44)	2.45E-06	5.30E-03	2.67 (1.67-3.67)	1.71E-07	3.79E-04	1.50 (0.22-2.77)	2.11E-02	1.00E+00
OR2T11	3	2.53 (1.36-3.70)	2.26E-05	4.89E-02	2.91 (1.75-4.07)	8.87E-07	1.96E-03	1.39 (-0.05-2.82)	5.84E-02	1.00E+00
OTOG	8	1.16 (0.46-1.86)	1.18E-03	1.00E+00	1.69 (0.99-2.39)	2.12E-06	4.69E-03	1.20 (0.35-2.05)	5.63E-03	1.00E+00
OTUD4	2				5.76 (3.97-7.56)	2.97E-10	6.58E-07	1.90 (-0.07-3.86)	5.84E-02	1.00E+00
PAPPA	2	2.97 (1.52-4.43)	5.97E-05	1.29E-01	3.49 (2.06-4.93)	1.89E-06	4.18E-03	2.59 (0.19-4.99)	3.47E-02	1.00E+00
PDZD2	3	3.21 (2.01-4.41)	1.49E-07	3.23E-04	4.01 (2.81-5.21)	5.45E-11	1.21E-07	1.38 (-0.05-2.82)	5.86E-02	1.00E+00
PGAP6	2	3.75 (2.23-5.27)	1.39E-06	3.01E-03	4.36 (2.86-5.86)	1.26E-08	2.79E-05			
PGPEP1	3	3.91 (2.65-5.17)	1.22E-09	2.64E-06	4.18 (2.97-5.39)	1.37E-11	3.03E-08	3.00 (0.73-5.27)	9.51E-03	1.00E+00

PHKA1	4	2.69 (1.66-3.72)	3.00E-07	6.49E-04	2.95 (1.93-3.96)	1.18E-08	2.61E-05	0.89 (-0.26-2.04)	1.31E-01	1.00E+00
PON1	4	1.62 (0.62-2.62)	1.46E-03	1.00E+00	2.24 (1.25-3.24)	1.03E-05	2.28E-02	1.50 (0.22-2.77)	2.11E-02	1.00E+00
PSMB11	2	3.67 (2.16-5.18)	1.97E-06	4.26E-03	3.60 (2.16-5.05)	9.79E-07	2.17E-03			
PYGM	3	2.13 (0.97-3.29)	3.19E-04	6.90E-01	2.52 (1.37-3.68)	1.74E-05	3.85E-02	1.20 (-0.19-2.60)	8.98E-02	1.00E+00
RAB12	2	5.35 (3.39-7.31)	9.16E-08	1.98E-04	5.45 (3.75-7.15)	3.32E-10	7.34E-07			
RAG1	4	2.01 (1.01-3.01)	8.65E-05	1.87E-01	2.21 (1.22-3.21)	1.32E-05	2.92E-02	1.09 (-0.10-2.27)	7.16E-02	1.00E+00
RBPJL	2	3.12 (1.66-4.58)	2.86E-05	6.19E-02	3.34 (1.90-4.77)	4.88E-06	1.08E-02	1.90 (-0.07-3.86)	5.84E-02	1.00E+00
RGMA	2	3.76 (2.24-5.28)	1.28E-06	2.77E-03	3.43 (1.99-4.86)	2.83E-06	6.27E-03	2.59 (0.19-4.99)	3.47E-02	1.00E+00
RP1L1	6	1.36 (0.55-2.17)	9.94E-04	1.00E+00	1.91 (1.10-2.72)	3.75E-06	8.31E-03	1.61 (0.55-2.67)	2.97E-03	1.00E+00
RPTOR	5	1.31 (0.42-2.20)	3.90E-03	1.00E+00	1.95 (1.06-2.84)	1.77E-05	3.91E-02	1.21 (0.13-2.29)	2.83E-02	1.00E+00
RTP5	3	3.01 (1.82-4.20)	7.15E-07	1.55E-03	3.63 (2.45-4.81)	1.64E-09	3.63E-06	1.20 (-0.19-2.60)	8.98E-02	1.00E+00
RYR1	7	1.25 (0.50-2.00)	1.06E-03	1.00E+00	1.75 (1.00-2.49)	4.59E-06	1.02E-02	1.01 (0.12-1.89)	2.53E-02	1.00E+00
RYR3	11	1.44 (0.84-2.04)	2.66E-06	5.75E-03	1.99 (1.39-2.59)	6.78E-11	1.50E-07	1.35 (0.61-2.10)	3.81E-04	7.04E-01
SIPA1L1	4	1.69 (0.69-2.69)	9.28E-04	1.00E+00	2.26 (1.26-3.25)	8.94E-06	1.98E-02	0.89 (-0.26-2.04)	1.31E-01	1.00E+00
SLC25A48	4	2.08 (1.08-3.09)	4.90E-05	1.06E-01	2.76 (1.75-3.76)	7.04E-08	1.56E-04	1.21 (0.00-2.41)	4.99E-02	1.00E+00
SLC26A6	3	2.52 (1.35-3.69)	2.37E-05	5.13E-02	2.83 (1.67-3.98)	1.67E-06	3.69E-03	2.30 (0.51-4.10)	1.18E-02	1.00E+00
SMYD1	5	2.90 (1.98-3.82)	6.68E-10	1.44E-06	3.17 (2.27-4.08)	6.47E-12	1.43E-08	1.43 (0.31-2.56)	1.25E-02	1.00E+00
SORCS2	2	4.24 (2.63-5.84)	2.25E-07	4.88E-04	4.52 (2.99-6.04)	6.07E-09	1.34E-05			
SORL1	6	1.50 (0.69-2.31)	3.01E-04	6.51E-01	2.03 (1.22-2.84)	9.13E-07	2.02E-03	1.05 (0.09-2.01)	3.22E-02	1.00E+00
SOX13	4	2.04 (1.04-3.05)	6.54E-05	1.42E-01	2.56 (1.56-3.56)	5.00E-07	1.11E-03	1.34 (0.11-2.58)	3.33E-02	1.00E+00
SPG11	4	1.81 (0.81-2.81)	3.79E-04	8.21E-01	2.24 (1.25-3.24)	1.02E-05	2.26E-02	1.21 (0.00-2.41)	4.99E-02	1.00E+00
STAB1	6	1.38 (0.57-2.19)	8.78E-04	1.00E+00	1.88 (1.07-2.69)	5.45E-06	1.21E-02	0.98 (0.03-1.93)	4.32E-02	1.00E+00
STARD9	6	2.43 (1.60-3.25)	7.82E-09	1.69E-05	2.10 (1.29-2.91)	3.72E-07	8.24E-04	1.90 (0.76-3.03)	1.05E-03	1.00E+00
STXBP5	9	1.64 (0.97-2.31)	1.59E-06	3.44E-03	2.20 (1.53-2.87)	1.15E-10	2.55E-07	2.51 (1.41-3.61)	8.01E-06	1.48E-02
TEX33	3	2.36 (1.20-3.53)	7.07E-05	1.53E-01	3.02 (1.86-4.18)	3.39E-07	7.51E-04	1.20 (-0.19-2.60)	8.98E-02	1.00E+00
TMEM151B	3	4.49 (3.13-5.85)	8.87E-11	1.92E-07	5.28 (3.92-6.64)	2.44E-14	5.41E-11			
TMPRSS7	2	4.27 (2.67-5.88)	1.79E-07	3.87E-04	4.78 (3.23-6.34)	1.61E-09	3.56E-06			
TNS3	3	1.91 (0.75-3.06)	1.20E-03	1.00E+00	2.51 (1.36-3.66)	1.97E-05	4.35E-02	1.05 (-0.31-2.41)	1.30E-01	1.00E+00
TRANK1	3	5.09 (3.59-6.59)	3.10E-11	6.71E-08	5.88 (4.38-7.39)	1.56E-14	3.46E-11	3.00 (0.73-5.27)	9.51E-03	1.00E+00
TRIM36	4	1.86 (0.86-2.86)	2.60E-04	5.63E-01	2.15 (1.16-3.14)	2.14E-05	4.73E-02	2.18 (0.68-3.68)	4.30E-03	1.00E+00
TRIM50	4	2.17 (1.16-3.17)	2.41E-05	5.22E-02	2.86 (1.86-3.86)	2.37E-08	5.24E-05	1.68 (0.36-3.00)	1.27E-02	1.00E+00
TTN	22	0.85 (0.42-1.27)	8.42E-05	1.82E-01	1.03 (0.61-1.45)	1.43E-06	3.16E-03	1.00 (0.50-1.49)	8.53E-05	1.58E-01
TTYH3	3	3.04 (1.85-4.23)	5.64E-07	1.22E-03	2.82 (1.66-3.97)	1.84E-06	4.06E-03	2.30 (0.51-4.10)	1.18E-02	1.00E+00

UBAC1	5	2.05 (1.15-2.94)	7.47E-06	1.62E-02	2.59 (1.69-3.48)	1.33E-08	2.94E-05	1.31 (0.21-2.41)	1.93E-02	1.00E+00
UPF3B	2	4.02 (2.42-5.63)	8.90E-07	1.93E-03	3.21 (1.77-4.65)	1.21E-05	2.68E-02	1.20 (-0.50-2.90)	1.66E-01	1.00E+00
USP19	4	2.09 (1.09-3.09)	4.55E-05	9.84E-02	2.36 (1.36-3.36)	3.45E-06	7.63E-03	2.19 (0.69-3.69)	4.26E-03	1.00E+00
USP49	4	3.51 (2.45-4.57)	8.54E-11	1.85E-07	3.90 (2.86-4.93)	1.58E-13	3.49E-10			
VWA5B1	5	1.74 (0.85-2.64)	1.26E-04	2.72E-01	2.08 (1.19-2.97)	4.28E-06	9.48E-03	1.31 (0.21-2.41)	1.93E-02	1.00E+00
WNT9B	4	1.83 (0.83-2.83)	3.23E-04	7.00E-01	2.35 (1.36-3.34)	3.56E-06	7.87E-03	3.28 (1.09-5.48)	3.34E-03	1.00E+00
XRN2	5	2.19 (1.28-3.09)	2.03E-06	4.38E-03	1.97 (1.08-2.86)	1.43E-05	3.16E-02	1.72 (0.53-2.91)	4.67E-03	1.00E+00
ZC3H12C	3	5.37 (3.77-6.98)	5.16E-11	1.12E-07	5.48 (4.09-6.87)	1.15E-14	2.55E-11	1.90 (0.29-3.50)	2.04E-02	1.00E+00
ZCCHC14	2	4.97 (3.17-6.76)	5.65E-08	1.22E-04	4.30 (2.80-5.79)	1.69E-08	3.73E-05	1.49 (-0.30-3.28)	1.03E-01	1.00E+00
ZMYM2	6	1.36 (0.55-2.18)	1.05E-03	1.00E+00	1.86 (1.05-2.67)	7.30E-06	1.62E-02	1.30 (0.30-2.30)	1.11E-02	1.00E+00
ZNF266	2	3.98 (2.42-5.53)	5.12E-07	1.11E-03	3.59 (2.15-5.03)	1.02E-06	2.25E-03	2.59 (0.18-4.99)	3.48E-02	1.00E+00
ZNF385B	2	2.70 (1.26-4.13)	2.35E-04	5.09E-01	3.34 (1.91-4.77)	4.87E-06	1.08E-02			
ZNF516	2	6.06 (3.66-8.46)	7.58E-07	1.64E-03	3.39 (1.96-4.82)	3.37E-06	7.45E-03	2.59 (0.18-4.99)	3.48E-02	1.00E+00
ZNF692	3	2.78 (1.61-3.96)	3.67E-06	7.95E-03	3.34 (2.17-4.51)	2.24E-08	4.95E-05	2.30 (0.51-4.10)	1.18E-02	1.00E+00
ZNRF3	4	1.98 (0.97-2.98)	1.13E-04	2.45E-01	2.53 (1.53-3.53)	6.94E-07	1.54E-03	1.50 (0.22-2.77)	2.11E-02	1.00E+00
ZXDC	4	1.93 (0.92-2.93)	1.65E-04	3.58E-01	2.49 (1.49-3.49)	1.03E-06	2.27E-03	1.68 (0.36-3.00)	1.27E-02	1.00E+00

Supplementary table 3. Burden of LoF variants in human genes found in the SEVTIN cohort. LOEUF values for genes with LoF variants were annnotated for each gene according to gnomAD. NFE: non-finnish Europeans from gnomAD; ALL: total population from gnomAD: Swedish: SweGen population. Total number of variants including LoF, missense and synonymous per gen and subgroup is also noted. Multiple hypothesis testing correction for each pvalue have been addressed by False Discovery Rate (FDR) approach for the total number of genes and total number of variants for each comparison. **Genes whose variants discovered in TIGER cohort have not been reported in SweGen have been removed.**

Genes have been ranked by gene constraint measured by LOEUF. Top genes are highly intolerant to LoF variants.

Gene	Locus	Variants	logOR (CI) NFE	pvalue	pvalue corrected	logOR (CI) ALL	pvalue	pvalue corrected	logOR (CI) Swedish	pvalue	pvalue corrected	LOEUF	Protein name (HGNC)
TUT4	1p32.3	8	2.91 (2.38-3.45)	0	0	2.89 (2.36-3.42)	0	0	1.67 (1.11-2.24)	6.94E-09	1.82E-06	0.11	Terminal Uridylyltransferase 4
FAM135A	6q13	7	2.79 (1.62-3.96)	2.82E-06	1.02E-03	2.75 (1.59-3.91)	2.82E-06	1.35E-03	3.43 (1.81-5.04)	3.39E-05	8.88E-03	0.27	Family with sequence similarity 135 member A
KIAA1109	4q27	14	3.67 (2.48-4.85)	1.34E-09	4.85E-07	3.58 (2.41-4.75)	1.96E-09	7.43E-07	3.14 (1.62-4.65)	5.07E-05	1.33E-02	0.41	Transmembrane Protein KIAA1109
DNAH7	2q32.3	23	2.68 (1.53-3.84)	5.07E-06	1.84E-03	2.84 (1.69-3.98)	1.31E-06	4.95E-04	3.81 (2.01-5.61)	3.28E-05	8.60E-03	0.84	Dynein axonemal heavy chain 7
TMEM232	5q22.1	8	4.91 (3.68-6.14)	5.40E-15	1.98E-12	4.31 (3.13-5.48)	7.06E-13	2.68E-10	3.43 (1.81-5.04)	3.39E-05	8.88E-03	1.08	Transmembrane Protein 232
TMEM41A	3q27.2	5	3.44 (2.71-4.17)	0	0	3.17 (2.45-3.89)	0	2E-15	1.73 (0.95-2.50)	1.22E-05	3.20E-03	1.17	Transmembrane Protein 41A
ATP7B	13q14.3	17	3.73 (3.22-4.24)	0	0	3.34 (2.84-3.84)	0	0	3.41 (2.73-4.09)	0	0	1.19	ATPase copper transporting beta
DYNLT2B	3q29	6	3.13 (2.30-3.96)	1.37E-13	4.98E-11	2.71 (1.89-3.53)	1.02E-10	3.88E-08	1.99 (1.09-2.90)	1.69E-05	4.43E-03	1.49	Dynein light chain Tctex-type 2B

Supplementary table 4. Enrichment analysis for LoF variants found in TIGER cohort. Only genes significatively enriched are found in this table. LOEUF values for LoF variant tolerance are noted for each gene according to gnomAD. NFE: non-finnish Europeans from gnomAD; ALL: total population from gnomAD: Swedish: SweGen population. Total number of variants including LoF, missense and synonymous per gene and subgroup is also noted. Genes whose variants discovered in TIGER cohort have not been detected in SweGen have been removed. Multiple hypothesis testing correction for each pvalue have been addressed by False Discovery Rate (FDR) approach for the total number of genes and total number of variants for each comparison.

Genes have been ranked by constraint measured by LOEUF. Top genes are highly intolerant to LoF variants.

Gene	Locus	Variants	logOR (CI) NFE	pvalue	pvalue corrected	logOR (CI) ALL	pvalue	pvalue corrected	logOR (CI) Swedish	pvalue	pvalue corrected	LOEUF	Protein name (HGNC)
KDM4A	1p34.2-p34.1	6	3.58 (2.76-4.40)	<10E-15	0	3.98 (3.15-4.82)	<10E-15	0	3.41 (2.27-4.55)	4.60E-09	1.25E-06	0.16	Lysine Demethylase 4A
CYLD	16q12.1	9	3.35 (2.77-3.94)	<10E-15	0	3.62 (3.03-4.21)	<10E-15	0	3.83 (2.93-4.73)	1.00E-16	0.00E+00	0.2	CYLD Lysine 63 Deubiquitinase
LHX2	9q33.3	9	4.40 (3.72-5.09)	<10E-15	0	5.21 (4.47-5.94)	<10E-15	0	4.52 (3.21-5.84)	1.51E-11	1.08E-07	0.22	LIM Homeobox 2
PRDM2	1p36.21	28	3.39 (2.93-3.85)	<10E-15	0	3.50 (3.04-3.97)	<10E-15	0	2.98 (2.41-3.56)	<10E-15	0	0.22	PR/SET Domain 2
TMEM132D	12q24.33	13	4.09 (3.53-4.66)	<10E-15	0	4.64 (4.05-5.23)	<10E-15	0	2.86 (2.17-3.55)	5.00E-16	6.30E-11	0.23	Transmembrane Protein 132D
EEPD1	7p14.2	8	2.08 (1.41-2.75)	8.86E-11	5.14E-05	2.23 (1.55-2.90)	8.86E-11	6.47E-04	2.22 (1.46-2.98)	1.00E-08	4.17E-03	0.58	Endonuclease/Exonuclease/Phosphatase Family Domain Containing 1
CEP131	17q25.3	4	4.15 (3.12-5.19)	<10E-15	0	4.80 (3.69-5.90)	<10E-15	0	4.13 (2.42-5.85)	2.32E-06	0.00E+00	0.78	Centrosomal Protein 131
COLGALT1	19p13.11	9	2.86 (2.05-3.68)	5.17E-14	0.00E+00	3.16 (2.34-3.99)	5.17E-14	0.00E+00	2.26 (1.33-3.18)	1.94E-06	1.95E-13	0.88	Collagen Beta(1-O)Galactosyltransferase 1
NAP1L3	Xq21.32	3	3.88 (3.16-4.61)	<10E-15	0	4.10 (3.36-4.84)	<10E-15	0	3.57 (2.54-4.60)	1.02E-11	3.46E-08	0.9	Nucleosome Assembly Protein 1 Like 3
SLCO4A1	20q13.33	13	3.98 (3.47-4.49)	<10E-15	0	4.28 (3.75-4.80)	<10E-15	0	4.02 (3.19-4.84)	<10E-15	0	1	Solute Carrier Organic Anion Transporter Family Member 4A1
SYNE3	14q32.13	18	6.00 (5.24-6.77)	<10E-15	0	6.11 (5.26-6.96)	<10E-15	0	3.70 (2.63-4.76)	1.03E-11	1.74E-06	1.08	Spectrin Repeat Containing Nuclear Envelope Family Member 3
ANKRD27	19q13.11	13	4.03 (3.50-4.56)	<10E-15	0	4.49 (3.94-5.04)	<10E-15	0	5.47 (3.99-6.95)	4.66E-13	3.33E-05	1.1	Ankyrin Repeat Domain 27
GPAT3	4q21.23	8	3.16 (2.65-3.66)	<10E-15	0	3.27 (2.75-3.78)	<10E-15	0	2.13 (1.56-2.70)	1.70E-13	1.60E-15	1.11	Glycerol-3-Phosphate Acyltransferase 3
PIK3R6	17p13.1	7	2.83 (2.25-3.41)	<10E-15	1.2222E-11	2.96 (2.38-3.54)	<10E-15	4.9797E-10	3.01 (2.27-3.75)	1.40E-15	2.22E-05	1.46	Phosphoinositide-3-Kinase Regulatory Subunit 6
ENTPD2	9q34.3	11	2.46 (1.83-3.09)	3.57E-14	0.00E+00	2.44 (1.81-3.07)	3.57E-14	0.00E+00	1.44 (0.77-2.11)	2.48E-05	1.25E-12	1.47	Ectonucleoside Triphosphate Diphosphohydrolase 2
TNFRSF10A	8p21.3	7	3.89 (3.06-4.72)	<10E-15	0	4.22 (3.37-5.07)	<10E-15	0	2.73 (1.73-3.73)	7.62E-08	1.47E-04	1.56	TNF Receptor Superfamily Member 10a
OR5M9	11q12.1	9	3.20 (2.63-3.76)	<10E-15	0	3.39 (2.82-3.96)	<10E-15	0	3.47 (2.75-4.19)	<10E-15	0	1.62	Olfactory Receptor Family 5 Subfamily M Member 9

Supplementary table 5. Missense variants found in candidate genes for tinnitus extreme phenotype ANK2, AKAP9 and TSC2 (Amanat et al., 2021).

Variant (ANK2)	Consequence	Location	Exon	Frequency	Max.Freq.P op.	Male (n)	Female (n)	CADD Phred
rs149645600	missense_variant	protein_codin	38/46	0.003	gnomAD_NFE	0	1	22.9
rs141191319	missense_variant	protein_codin	38/46	0.004	gnomAD_NFE	2	2	18.80
rs753223319	missense_variant	protein_codin	38/46	0.0003265	gnomAD_EAS	0	1	7,961
rs764914059	missense_variant	protein_codin	38/46	8.95E-03	gnomAD_NFE	1	0	10.46
rs145895389	missense_variant	protein_codin	38/46	0.00302	gnomAD_NFE	1	0	4,351
rs36210417	missense_variant	protein_codin	38/46	0.02757	gnomAD_ASJ	3	2	24.9
rs121912706	missense_variant	protein_codin	45/46	0.002	gnomAD_NFE	1	0	27.7
rs45454496	missense_variant	protein_codin	45/46	0.01837	gnomAD_ASJ	0	1	23.3

Variant (AKAP9)	Consequence	Location	Exon	Frequency	Max.Freq.P op.	Male (n)	Female (n)	CADD Phred
rs137853994	missense_variant	chr16:205339	4/42	0.002213	gnomAD_EAS	1	0	26.4
rs1800725	missense_variant	chr16:206079	11/42	0.02716	gnomAD_ASJ	1	2	26.5
rs374936223	missense_variant	chr16:206199	12/42	0.0002326	gnomAD_EAS	0	1	24.7
rs397515223	missense_variant	chr16:207181	19/42	0.001326	gnomAD_FIN	0	1	24.7
rs137854410	missense_variant	chr16:207904	27/42	0.0008	gnomAD_AFR	1	0	25.1
rs45517419	missense_variant	chr16:208854	42/42	0.005455	gnomAD_FIN	0	2	0.102

Variant (TSC2)	Consequence	Feature	Exon	Frequency	op.	Male (n)	Female (n)	CADD Phred
rs35669569	missense_variant	protein_codin	2/50	0.01423	gnomAD_FIN	2	4	5,917
rs146305558	missense_variant	protein_codin	19/50	0.001	gnomAD_EAS	1	0	25.8
rs144875383	missense_variant	protein_codin	33/50	0.003	gnomAD_NFE	0	1	8,536
CHR7:92098212	se_variant&splice_region_	protein_codin	43/50	,	,	0	1	32
rs34327395	missense_variant	protein_codin	44/50	0.0139	gnomAD_NFE	1	0	18.96

Supplementary table 6. Genes with significant variant enrichment replicated between TIGER and independant swedish JAGUAR cohort. LoF-enriched genes replicated in the exome replication cohort are summarised in the upper table (A). Missense-enriched genes replicated in the exome replication cohort are summarised in the second table (B). If no variant for a gene was called for SEVTIN (N Variants = 0), info for that gene will appear in blank as enrichment can not be estimated.

A		TIGER						
**Gene**	**LOEUF**	**N Variants (TIGER)**	**logOR (CI) NFE**	**pvalue**	**logOR (CI) ALL**	**pvalue**	**logOR (CI) Swedish**	**pvalue**
PTCH2	0.82	2	5.81 (3.85-7.78)	6.43E-09	6.61 (4.65-8.57)	4.07E-11	1.93 (0.14-3.72)	3.46E-02
RAB25	1.55	2	2.87 (1.44-4.30)	8.14E-05	3.46 (2.04-4.88)	1.89E-06	1.42 (-0.22-3.07)	8.99E-02
TNFRSF10A	1.56	13	3.36 (2.79-3.93)	0.00E+00	3.13 (2.57-3.69)	0.00E+00	2.02 (1.31-2.74)	3.27E-08

B		TIGER						
**Gene**	**LOEUF**	**N Variants (TIGER)**	**logOR (CI) NFE**	**pvalue**	**logOR (CI) ALL**	**pvalue**	**logOR (CI) Swedish**	**pvalue**
PTCH1	0.07	5	2.57 (1.67-3.47)	1.89E-08	2.29 (1.41-3.18)	3.82E-07	1.31 (0.28-2.33)	1.23E-02
HEATR1	0.14	8	0.77 (0.07-1.47)	3.11E-02	0.93 (0.23-1.62)	9.26E-03	0.70 (-0.06-1.46)	7.00E-02
ZFHX3	0.14	22	0.99 (0.57-1.41)	3.98E-06	0.92 (0.49-1.34)	1.98E-05	0.42 (-0.03-0.87)	6.93E-02
NAV2	0.25	3	1.16 (0.01-2.30)	4.72E-02	1.78 (0.64-2.92)	2.22E-03	1.24 (-0.07-2.55)	6.37E-02
ARHGEF17	0.29	5	1.68 (0.80-2.57)	1.99E-04	2.20 (1.32-3.09)	1.02E-06	2.34 (1.10-3.58)	2.23E-04
LAMA5	0.31	11	0.79 (0.20-1.39)	9.15E-03	0.94 (0.35-1.54)	1.88E-03	0.84 (0.19-1.50)	1.17E-02
NBEAL2	0.32	3	2.14 (0.99-3.29)	2.61E-04	2.59 (1.45-3.73)	9.21E-06	0.66 (-0.57-1.90)	2.94E-01
PTPRU	0.34	2	2.95 (1.52-4.38)	5.17E-05	3.69 (2.27-5.12)	3.92E-07	3.03 (0.63-5.43)	1.34E-02
PLXNB1	0.39	2	3.51 (2.05-4.97)	2.34E-06	3.97 (2.53-5.41)	6.24E-08		
FOXK2	0.58	7	1.48 (0.72-2.23)	1.17E-04	1.79 (1.05-2.54)	2.61E-06	1.52 (0.62-2.41)	8.70E-04
STARD9	0.63	5	1.48 (0.72-2.23) (	1.53E-02	1.31 (0.43-2.20)	3.46E-03	0.90 (-0.08-1.88)	7.08E-02
CCDC168	0.68	12	1.26 (0.68-1.83)	1.71E-05	1.47 (0.90-2.04)	4.79E-07	0.60 (-0.01-1.22)	5.51E-02
EVPL	0.75	34	5.16 (4.75-5.58)	0.00E+00	4.55 (4.18-4.91)	0.00E+00	4.51 (3.47-5.55)	0.00E+00
MUC16	0.76	23	1.43 (1.01-1.84)	1.27E-11	1.38 (0.97-1.79)	5.26E-11	1.41 (0.93-1.90)	1.06E-08
CEP131	0.78	3	5.39 (3.60-7.18)	3.69E-09	4.80 (3.30-6.30)	3.53E-10	3.03 (0.63-5.43)	1.34E-02
PYGM	0.87	7	1.50 (0.75-2.25)	8.60E-05	1.69 (0.94-2.44)	9.69E-06	0.85 (0.03-1.68)	4.29E-02
SYNE3	1.08	5	5.62 (4.43-6.81)	0.00E+00	5.21 (4.23-6.20)	0.00E+00		
NINL	1.09	7	2.02 (1.27-2.77)	1.39E-07	2.56 (1.81-3.31)	2.28E-11	1.15 (0.30-2.00)	7.99E-03
TRPM2	1.11	3	2.00 (0.85-3.15)	6.40E-04	2.63 (1.48-3.78)	6.96E-06	1.49 (0.14-2.85)	3.11E-02
SLC12A9	1.16	2	-0.03 (-1.42-1.37)	9.71E-01	0.51 (-0.89-1.90)	4.76E-01	-0.34 (-1.78-1.09)	6.40E-01
NAGA	1.3	3	3.97 (2.75-5.19)	1.92E-10	4.67 (3.45-5.88)	4.91E-14	3.44 (1.17-5.70)	2.93E-03
LRWD1	1.31	2	4.72 (3.11-6.33)	8.55E-09	5.23 (3.67-6.78)	4.50E-11	1.09 (-0.49-2.67)	1.77E-01
RP1L1	1.95	4	0.63 (-0.36-1.62)	2.10E-01	0.90 (-0.08-1.89)	7.19E-02	1.33 (0.18-2.47)	2.35E-02
CSMD2	not available	5	3.10 (2.19-4.02)	2.36E-11	3.19 (2.29-4.08)	3.12E-12		

A		SEVTIN						
**Gene**	**LOEUF**	**N Variants (SEVTIN)**	**logOR (CI) NFE**	**pvalue**	**logOR (CI) ALL**	**pvalue**	**logOR (CI) Swedish**	**pvalue**
PTCH2	0.82	0						
RAB25	1.55	0						
TNFRSF10A	1.56	6	4.22 (3.37-5.07)	0.00E+00	3.89 (3.06-4.72)	0.00E+00	2.73 (1.73-3.73)	7.62E-08

B		SEVTIN						
**Gene**	**LOEUF**	**N Variants (SEVTIN)**	**logOR (CI) NFE**	**pvalue**	**logOR (CI) ALL**	**pvalue**	**logOR (CI) Swedish**	**pvalue**
PTCH1	0.07	2	3.30 (1.88-4.71)	4.93E-06	2.67 (1.26-4.07)	1.92E-04	2.14 (0.56-3.72)	7.92E-03
HEATR1	0.14	7	2.18 (1.43-2.93)	1.33E-08	2.24 (1.49-2.99)	4.57E-09	1.79 (0.97-2.61)	1.85E-05
ZFHX3	0.14	12	1.64 (1.07-2.21)	1.92E-08	1.61 (1.04-2.18)	3.48E-08	1.02 (0.42-1.61)	8.28E-04
NAV2	0.25	5	3.25 (2.36-4.15)	1.01E-12	3.51 (2.62-4.40)	1.03E-14	3.40 (2.15-4.64)	8.87E-08
ARHGEF17	0.29	2	1.51 (0.10-2.92)	3.57E-02	1.66 (0.26-3.07)	2.05E-02	1.39 (-0.11-2.88)	6.88E-02
LAMA5	0.31	11	1.30 (0.71-1.90)	1.87E-05	1.56 (0.97-2.16)	2.78E-07	1.06 (0.44-1.69)	8.40E-04
NBEAL2	0.32	0						
PTPRU	0.34	0						
PLXNB1	0.39	4	1.73 (0.72-2.75)	7.95E-04	2.04 (1.03-3.05)	7.61E-05	1.60 (0.51-2.68)	3.90E-03
FOXK2	0.58	4	2.05 (1.05-3.04)	5.45E-05	2.36 (1.36-3.35)	3.23E-06	1.53 (0.46-2.59)	4.89E-03
STARD9	0.63	8	1.75 (1.05-2.45)	9.11E-07	2.08 (1.38-2.78)	5.61E-09	1.67 (0.91-2.42)	1.60E-05
CCDC168	0.68	15	1.36 (0.85-1.88)	1.68E-07	1.67 (1.16-2.18)	1.39E-10	0.95 (0.42-1.48)	4.51E-04
EVPL	0.75	4	1.73 (0.74-2.72)	6.46E-04	1.94 (0.95-2.93)	1.25E-04	1.95 (0.85-3.05)	4.97E-04
MUC16	0.76	20	1.55 (1.11-2.00)	6.59E-12	1.39 (0.95-1.84)	6.83E-10	1.17 (0.70-1.63)	8.43E-07
CEP131	0.78	3	5.14 (3.90-6.37)	4.00E-16	5.88 (4.65-7.11)	0.00E+00	3.11 (1.60-4.61)	5.09E-05
PYGM	0.87	2	1.87 (0.46-3.28)	9.38E-03	1.92 (0.51-3.33)	7.48E-03	1.10 (-0.38-2.57)	1.44E-01
SYNE3	1.08	2	5.74 (4.13-7.36)	3.48E-12	5.85 (4.34-7.37)	3.88E-14		
NINL	1.09	3	2.91 (1.76-4.06)	7.02E-07	3.32 (2.17-4.47)	1.42E-08	1.66 (0.42-2.89)	8.50E-03
TRPM2	1.11	3	4.92 (3.71-6.14)	2.20E-15	5.30 (4.11-6.49)	0.00E+00	3.80 (2.01-5.60)	3.30E-05
SLC12A9	1.16	2	3.50 (2.07-4.93)	1.60E-06	3.76 (2.34-5.18)	2.12E-07	1.90 (0.35-3.45)	1.62E-02
NAGA	1.3	0						
LRWD1	1.31	0						
RP1L1	1.95	8	1.94 (1.24-2.64)	5.99E-08	2.27 (1.57-2.97)	2.02E-10	1.69 (0.93-2.45)	1.27E-05
CSMD2	not available	5	2.32 (1.42-3.21)	3.56E-07	2.75 (1.86-3.64)	1.40E-09	2.37 (1.34-3.41)	6.35E-06

A		JAGUAR					
**Gene**	**LOEUF**	**N Variants (Replicatio**	**logOR (CI) NFE**	**pvalue**	**logOR (CI) ALL**	**pvalue**	**logOR (CI) Swedish**	**pvalue**
PTCH2	0.82	3	5.80 (4.01-7.59)	2.15E-10	6.60 (4.81-8.39)	5.10E-13	1.92 (0.32-3.52)	1.88E-02
RAB25	1.55	2	2.45 (1.03-3.88)	7.49E-04	3.04 (1.62-4.46)	2.74E-05	1.01 (-0.64-2.65)	2.31E-01
TNFRSF10A	1.56	2	2.76 (1.32-4.20)	1.71E-04	3.45 (2.02-4.89)	2.36E-06	0.31 (-1.21-1.83)	6.90E-01

B		JAGUAR						
**Gene**	**LOEUF**	**N Variants (Replicatio**	**logOR (CI) NFE**	**pvalue**	**logOR (CI) ALL**	**pvalue**	**logOR (CI) Swedish**	**pvalue**
PTCH1	0.07	4	3.46 (2.41-4.51)	1.25E-10	2.39 (1.39-3.39)	2.66E-06	1.52 (0.25-2.79)	1.89E-02
HEATR1	0.14	5	2.00 (1.10-2.89)	1.22E-05	2.31 (1.42-3.20)	3.52E-07	2.44 (1.00-3.87)	8.70E-04
ZFHX3	0.14	9	1.91 (1.24-2.57)	1.83E-08	2.32 (1.65-2.98)	6.99E-12	1.48 (0.64-2.32)	5.48E-04
NAV2	0.25	2	2.54 (1.11-3.97)	5.08E-04	3.28 (1.85-4.71)	6.79E-06		
ARHGEF17	0.29	4	2.59 (1.58-3.60)	5.06E-07	3.29 (2.28-4.30)	1.64E-10	1.70 (0.38-3.01)	1.15E-02
LAMA5	0.31	9	1.70 (1.04-2.36)	5.12E-07	2.27 (1.61-2.93)	1.81E-11	1.08 (0.29-1.86)	7.14E-03
NBEAL2	0.32	6	2.42 (1.60-3.25)	8.43E-09	2.87 (2.05-3.69)	6.04E-12	0.94 (0.00-1.89)	4.99E-02
PTPRU	0.34	2	5.40 (3.43-7.36)	7.03E-08	5.09 (3.49-6.70)	4.65E-10		
PLXNB1	0.39	3	2.57 (1.40-3.74)	1.69E-05	3.08 (1.92-4.24)	2.08E-07	0.94 (-0.39-2.28)	1.66E-01
FOXK2	0.58	4	4.39 (3.24-5.54)	7.02E-14	3.17 (2.16-4.18)	7.68E-10	1.01 (-0.16-2.18)	8.97E-02
STARD9	0.63	3	2.43 (1.27-3.60)	4.10E-05	3.07 (1.91-4.23)	2.03E-07	0.94 (-0.39-2.27)	1.66E-01
CCDC168	0.68	7	1.34 (0.59-2.10)	4.58E-04	1.72 (0.97-2.47)	7.17E-06	1.16 (0.26-2.06)	1.14E-02
EVPL	0.75	3	2.80 (1.62-3.97)	3.19E-06	3.19 (2.03-4.35)	7.45E-08	1.63 (0.13-3.13)	3.28E-02
MUC16	0.76	16	0.79 (0.30-1.29)	1.65E-03	1.23 (0.73-1.72)	1.09E-06	0.74 (0.18-1.30)	9.64E-03
CEP131	0.78	2	4.14 (2.57-5.72)	2.48E-07	4.69 (3.15-6.22)	2.12E-09		
PYGM	0.87	6	1.49 (0.68-2.30)	3.30E-04	1.88 (1.08-2.69)	5.05E-06	0.88 (-0.05-1.82)	6.44E-02
SYNE3	1.08	5	1.47 (0.58-2.36)	1.25E-03	1.93 (1.04-2.82)	2.08E-05	1.46 (0.34-2.58)	1.09E-02
NINL	1.09	2	4.30 (2.69-5.90)	1.48E-07	4.49 (2.98-6.00)	5.55E-09	2.61 (0.21-5.02)	3.30E-02
TRPM2	1.11	4	1.78 (0.78-2.78)	4.87E-04	2.15 (1.15-3.14)	2.30E-05	1.71 (0.38-3.03)	1.14E-02
SLC12A9	1.16	3	2.42 (1.26-3.59)	4.65E-05	2.68 (1.53-3.84)	5.26E-06	0.82 (-0.49-2.14)	2.18E-01
NAGA	1.3	4	1.76 (0.76-2.76)	5.34E-04	2.28 (1.29-3.28)	6.59E-06	2.21 (0.71-3.71)	3.85E-03
LRWD1	1.31	2	4.30 (2.70-5.90)	1.50E-07	4.81 (3.26-6.36)	1.30E-09	0.67 (-0.91-2.24)	4.06E-01
RP1L1	1.95	4	4.49 (3.32-5.65)	4.32E-14	4.95 (3.83-6.07)	0.00E+00	1.52 (0.25-2.79)	1.89E-02
CSMD2	not available	4	3.08 (2.04-4.11)	5.26E-09	2.21 (1.22-3.21)	1.34E-05	1.52 (0.25-2.79)	1.89E-02

Supplementary table 7. Candidate likely pathogenic SV calls found in the TIGER cohort and SweGen reference dataset (ACMG score = 4, likely pathogenic; ACME score = 5, pathogenic). In blue, candidate likely pathogenic LSV annotated in a high constraint region using gnomAD pLI > 0.9.

SV call	Length	TIGER (Frq)	SweGen (Frq)	OR	pvalue	Ranking criteria	gnomAD	ACMG	Genes overlapping
1_1445966_1497748_DEL_1	51782	0.0103	0.004	2.58	3.71E-01	1A (+0.00);2A (cf P_loss_source, +1.00);3A (2 genes, +0.00);5F (+0.00)	7.27E-09	5	ATAD3B;ATAD3C
1_16037276_16060630_DEL_1	23289	0.0412	0.015	2.78	8.01E-02	1A (+0.00);2A (cf P_loss_source, +1.00);3A (2 genes, +0.00);5F (+0.00)	1.15E-02	5	CLCNKB;FAM131C
10_86062_97355_DEL_1	11293	0.0103	0.009	1.15	6.05E-01	1A (+0.00);2A (cf P_loss_source, +1.00);3A (0 gene, +0.00);5F (+0.00)		5	
13_36047653_36051585_DEL_1	3932	0.0103	0.002	5.17	2.43E-01	1A (+0.00);2A (cf P_loss_source, +1.00);3A (1 gene, +0.00);5F (+0.00)	1.00E+00	5	DCLK1
19_17331876_17339761_DUP_1	7804	0.0825	0.058	1.44	3.74E-01	1A (+0.00);2A (cf P_gain_source, +1.00);3A (2 genes, +0.00);5F (+0.00)	9.56E-01	5	ANO8;GTPBP3
22_24236524_24237988_DEL_1	1464	0.0103	0.002	5.17	2.43E-01	1A (+0.00);2A (cf P_loss_source, +1.00);3A (1 gene, +0.00);5F (+0.00)	2.16E-11	5	GGT5
3_9993722_10047700_DUP_1	53928	0.0619	0.115	0.52	1.39E-01	1A (+0.00);2A (cf P_gain_source, +1.00);3A (4 genes, +0.00);5F (+0.00)	2.86E-01	5	CIDECP1;EMC3;EMC3-AS1;FANCD2
3_9994134_10047917_DEL_1	53587	0.1959	0.219	0.88	7.17E-01	1A (+0.00);2A (cf P_loss_source, +1.00);3A (4 genes, +0.00);5F (+0.00)	2.86E-01	5	CIDECP1;EMC3;EMC3-AS1;FANCD2
4_68580890_68676412_DEL_1	95440	0.1649	0.201	0.80	5.28E-01	1A (+0.00);2A (cf P_loss_source, +1.00);3A (1 gene, +0.00);5F (+0.00)	2.29E-21	5	UGT2B15
4_106170668_106178063_DEL_1	7395	0.0103	0.002	5.17	2.43E-01	1A (+0.00);2A (cf P_loss_source, +1.00);3A (1 gene, +0.00);5F (+0.00)	2.09E-15	5	TBCK
6_32810807_32885394_DEL_1	74561	0.0928	0.163	0.55	9.25E-02	1A (+0.00);2A (cf P_loss_source, +1.00);3A (6 genes, +0.00);5F (+0.00)	2.18E-02	5	HLA-DOB;PSMB8;PSMB8-AS1;PSMB9;TAP1;TAP2
7_142454546_142473462_DEL_1	18916	0.0103	0.024	0.43	7.20E-01	1A (+0.00);2A (cf P_loss_source, +1.00);3A (1 gene, +0.00);5F (+0.00)		5	TCAF2
7_150603940_150682167_DEL_1	78227	0.3402	0.413	0.79	2.62E-01	1A (+0.00);2A (cf P_loss_source, +1.00);3A (1 gene, +0.00);5F (+0.00)	1.42E-01	5	GIMAP6
8_142877802_142915909_DEL_1	37945	0.5361	0.486	1.14	4.33E-01	1A (+0.00);2A (cf P_loss_source, +1.00);3A (2 genes, +0.00);5F (+0.00)	8.25E-07	5	CYP11B1;CYP11B2
X_6826751_6910749_DEL_1	83998	0.0206	0.007	2.96	1.86E-01	1A (+0.00);2A (cf P_loss_source, +1.00);3A (0 gene, +0.00);5F (+0.00)		5	
1_16049908_16059762_DEL_1	9741	0.3711	0.517	0.65	2.45E-02	1A (+0.00);2D-4 (CLCNKB, +0.90);3A (2 genes, +0.00);5F (+0.00)	1.15E-02	4	CLCNKB;FAM131C
1_108190708_108194677_DEL_1	3921	0.3918	0.405	0.96	9.25E-01	1A (+0.00);2C-1 (SLC25A24, +0.90);3A (1 gene, +0.00);5F (+0.00)	3.35E-12	4	SLC25A24
10_94737567_94799352_DEL_1	61785	0.0103	0.003	3.45	3.10E-01	1A (+0.00);2C-1 (CYP2C19, +0.90);3A (1 gene, +0.00);5F (+0.00)	6.49E-20	4	CYP2C19
11_763165_764482_DEL_1	1317	0.0103	0.04	0.25	2.58E-01	1A (+0.00);2E-1 (TALDO1, +0.90);3A (1 gene, +0.00);5F (+0.00)	2.52E-06	4	TALDO1
11_78476711_78486359_DEL_1	9648	0.0103	0.003	3.45	3.10E-01	1A (+0.00);2E-1 (NARS2, +0.90);3A (1 gene, +0.00);5F (+0.00)	7.11E-08	4	NARS2
11_95847898_95849672_DEL_1	1774	0.0309	0.023	1.35	4.96E-01	1A (+0.00);2E-1 (MTMR2, +0.90);3A (1 gene, +0.00);5F (+0.00)	3.04E-07	4	MTMR2
11_95850748_95857550_DEL_1	6802	0.0309	0.023	1.35	4.96E-01	1A (+0.00);2E-1 (MTMR2, +0.90);3A (1 gene, +0.00);5F (+0.00)	3.04E-07	4	MTMR2
11_95858631_95861990_DEL_1	3359	0.0309	0.023	1.35	4.96E-01	1A (+0.00);2E-1 (MTMR2, +0.90);3A (1 gene, +0.00);5F (+0.00)	3.04E-07	4	MTMR2
11_95865674_95887609_DEL_1	21935	0.0103	0.016	0.64	1.00E+00	1A (+0.00);2E-1 (MTMR2, +0.90);3A (1 gene, +0.00);5F (+0.00)	3.04E-07	4	MTMR2
12_56236054_56284834_DEL_1	48780	0.0103	0.005	2.07	4.27E-01	1A (+0.00);2D-4 (SLC39A5, +0.90);3A (4 genes, +0.00);5F (+0.00)	1.00E+00	4	ANKRD52;COQ10A;CS;SLC39A5
15_58404787_58495121_DEL_1	90334	0.0103	0.001	10.33	1.69E-01	1A (+0.00);2C-1 (LIPC, +0.90);3A (2 genes, +0.00);5F (+0.00)	3.28E-07	4	LIPC;LIPC-AS1
19_40849890_40881764_DEL_1	31857	0.0206	0.023	0.90	1.00E+00	1A (+0.00);2C-1 (CYP2A6, +0.90);3A (2 genes, +0.00);5F (+0.00)	5.40E-09	4	CYP2A6;CYP2A7
2_178432293_178441472_DEL_1	9179	0.0103	0.001	10.33	1.69E-01	1A (+0.00);2E-1 (PRKRA, +0.90);3A (2 genes, +0.00);5F (+0.00)	4.17E-01	4	CHROMR;PRKRA
2_178436407_178443151_DEL_1	6718	0.0206	0.047	0.43	3.13E-01	1A (+0.00);2E-1 (PRKRA, +0.90);3A (2 genes, +0.00);5F (+0.00)	4.17E-01	4	CHROMR;PRKRA
2_178443492_178450151_DEL_1	6659	0.0103	0.029	0.35	5.12E-01	1A (+0.00);2E-1 (PRKRA, +0.90);3A (1 gene, +0.00);5F (+0.00)	4.17E-01	4	PRKRA
2_178447584_178450239_DEL_1	2655	0.1031	0.219	0.44	9.47E-03	1A (+0.00);2E-1 (PRKRA, +0.90);3A (1 gene, +0.00);5F (+0.00)	4.17E-01	4	PRKRA
2_190309088_190318358_DEL_1	9270	0.0103	0.003	3.45	3.10E-01	1A (+0.00);2E-1 (HIBCH, +0.90);3A (1 gene, +0.00);5F (+0.00)	2.58E-13	4	HIBCH
2_227330844_227332416_DEL_1	1572	0.0412	0.073	0.56	3.10E-01	1A (+0.00);2E-1 (MFF, +0.90);3A (1 gene, +0.00);5F (+0.00)	6.54E-02	4	MFF
6_3156593_3180222_DEL_1	23542	0.1856	0.025	8.06	4.28E-09	1A (+0.00);2C-1 (TUBB2A, +0.90);3A (1 gene, +0.00);5F (+0.00)	9.35E-01	4	TUBB2A
6_10467776_10530962_DEL_1	63186	0.0103	0.003	3.45	3.10E-01	1A (+0.00);2C-1 (GCNT2, +0.90);3A (1 gene, +0.00);5F (+0.00)	2.41E-05	4	GCNT2
7_142749957_142760873_DEL_1	10883	0.0206	0.081	0.25	2.95E-02	1A (+0.00);2D-4 (PRSS1, +0.90);3A (2 genes, +0.00);5F (+0.00)	4.14E-11	4	PRSS1;TCAF2
9_133433471_133434930_DEL_1	1459	0.0103	0.001	10.33	1.69E-01	1A (+0.00);2E-1 (ADAMTS13, +0.90);3A (1 gene, +0.00);5F (+0.00)	5.14E-15	4	ADAMTS13

Supplementary table 8. Candidate ultrarare large structural variants according to ACMG adapted criteria, quality filtering of SV length > 1Kb and SV length < 1Mb, and high constraint ratios (gnomAD pLI >0.9).

SV call	Length	Gene	TIGER (Frq)	SweGen (Frq)	OR	pvalue	gnomAD pLI	Ranking criteria	ACMG_class
13_36047653_36051585_DEL_1	3932	DCLK1	0.0103	0.002	5.18	0.24	1.00	1A (+0.00);2A (cf P_loss_source, +1.00);3A (1 gene, +0.00);5F (+0.00)	5
12_56236054_56284834_DEL_1	48780	ANKRD52;COQ10A;	0.0103	0.005	2.07	0.43	1.00	1A (+0.00);2D-4 (SLC39A5, +0.90);3A (4 genes, +0.00);5F (+0.00)	4
X_119923026_119930956_DUP_1	7930	NKAP	0.0103	0.008	1.29	0.57	1.00	1A (+0.00);2I-1 (NKAP, +0.45);3A (1 gene, +0.00);5F (+0.00)	3
10_34198170_34200550_DEL_1	2380	PARD3	0.0103	0.004	2.59	0.37	0.96	1A (+0.00);2H (PARD3, +0.15);3A (1 gene, +0.00);5F (+0.00)	3
10_113139007_113140800_DEL_1	1793	TCF7L2	0.0103	0.001	10.36	1.70E-01	1.00	1A (+0.00);2H (TCF7L2, +0.15);3A (1 gene, +0.00);5F (+0.00)	3
11_19617912_19620833_DEL_1	2921	NAV2	0.0103	0.001	10.36	0.17	1.00	1A (+0.00);2H (NAV2, +0.15);3A (1 gene, +0.00);5F (+0.00)	3
13_73783123_73786122_DEL_1	2999	KLF12	0.0103	0.001	10.36	0.17	0.98	1A (+0.00);2H (KLF12, +0.15);3A (1 gene, +0.00);5F (+0.00)	3
14_21234245_21269297_DEL_1	35052	HNRNPC	0.0103	0.013	0.79	1.00	0.98	1A (+0.00);2H (HNRNPC, +0.15);3A (1 gene, +0.00);5F (+0.00)	3
14_63488583_63497617_DEL_1	9034	PPP2R5E	0.0103	0.008	1.29	0.57	1.00	1A (+0.00);2H (PPP2R5E, +0.15);3A (1 gene, +0.00);5F (+0.00)	3
14_78692800_78699300_DEL_1	6500	NRXN3	0.0103	0.008	1.29	0.57	1.00	1A (+0.00);2H (NRXN3, +0.15);3A (1 gene, +0.00);5F (+0.00)	3
14_89360867_89362702_DEL_1	1835	FOXN3	0.0103	0.002	5.18	0.24	0.93	1A (+0.00);2H (FOXN3, +0.15);3A (1 gene, +0.00);5F (+0.00)	3
14_102178129_102179890_DEL_1	1761	WDR20	0.0103	0.008	1.29	0.57	0.92	1A (+0.00);2H (WDR20, +0.15);3A (1 gene, +0.00);5F (+0.00)	3
15_47314413_47357152_DEL_1	42739	SEMA6D	0.0103	0.013	0.79	1.00	0.94	1A (+0.00);2H (SEMA6D, +0.15);3A (1 gene, +0.00);5F (+0.00)	3
15_82634688_82645538_DEL_1	10850	CPEB1	0.0103	0.005	2.07	0.43	1.00	1A (+0.00);2H (CPEB1, +0.15);3A (1 gene, +0.00);5F (+0.00)	3
16_6271341_6275228_DEL_1	3887	RBFOX1	0.0103	0.003	3.46	0.31	0.95	1A (+0.00);2H (RBFOX1, +0.15);3A (1 gene, +0.00);5F (+0.00)	3
16_67053286_67054823_DEL_1	1537	CBFB	0.0103	0.011	0.94	1.00	0.96	1A (+0.00);2H (CBFB, +0.15);3A (1 gene, +0.00);5F (+0.00)	3
18_3635343_3636930_DEL_1	1587	DLGAP1	0.0103	0.001	10.36	0.17	1.00	1A (+0.00);2H (DLGAP1, +0.15);3A (1 gene, +0.00);5F (+0.00)	3
2_231105479_231106802_DEL_1	1323	PSMD1	0.0103	0.006	1.72	0.48	1.00	1A (+0.00);2H (PSMD1, +0.15);3A (1 gene, +0.00);5F (+0.00)	3
20_42228713_42230412_DEL_1	1699	PTPRT	0.0103	0.002	5.18	0.24	1.00	1A (+0.00);2H (PTPRT, +0.15);3A (1 gene, +0.00);5F (+0.00)	3
5_11567712_11569667_DEL_1	1955	CTNND2	0.0103	0.004	2.59	0.37	1.00	1A (+0.00);2H (CTNND2, +0.15);3A (1 gene, +0.00);5F (+0.00)	3
5_167653848_167661820_DEL_1	7972	TENM2	0.0103	0.011	0.94	1.00	1.00	1A (+0.00);2H (TENM2, +0.15);3A (1 gene, +0.00);5F (+0.00)	3
6_128111577_128116453_DEL_1	4876	PTPRK	0.0103	0.016	0.64	1.00	0.90	1A (+0.00);2H (PTPRK, +0.15);3A (1 gene, +0.00);5F (+0.00)	3
9_116660864_116662531_DEL_1	1667	ASTN2	0.0103	0.003	3.46	0.31	1.00	1A (+0.00);2H (ASTN2, +0.15);3A (1 gene, +0.00);5F (+0.00)	3
X_97351652_97353724_DEL_1	2072	DIAPH2	0.0103	0.031	0.33	0.35	0.99	1A (+0.00);2H (DIAPH2, +0.15);3A (1 gene, +0.00);5F (+0.00)	3
1_110610990_110613610_DEL_1	2620	KCNA2	0.0103	0.003	3.46	0.31	0.91	1A (+0.00);2C-2 (KCNA2,+0.00);3A (1 gene, +0.00);5F (+0.00)	3
10_321647_323353_DEL_1	1706	DIP2C	0.0103	0.003	3.46	0.31	1.00	1A (+0.00);3A (1 gene, +0.00);5F (+0.00)	3
10_14033603_14035226_DUP_1	1623	FRMD4A	0.0103	0.001	10.36	0.17	1.00	1A (+0.00);2L (FRMD4A, +0.00);3A (1 gene, +0.00);5F (+0.00)	3
10_79175456_79176701_DEL_1	1245	ZMIZ1	0.0103	0.024	0.42	0.72	1.00	1A (+0.00);2C-2 (ZMIZ1,+0.00);3A (1 gene, +0.00);5F (+0.00)	3
10_110025269_110037941_DEL_1	12672	ADD3	0.0103	0.002	5.18	0.24	1.00	1A (+0.00);2C-2 (ADD3,+0.00);3A (1 gene, +0.00);5F (+0.00)	3
10_115896440_115900552_DUP_1	4112	ATRNL1	0.0103	0.004	2.59	0.37	1.00	1A (+0.00);2L (ATRNL1, +0.00);3A (1 gene, +0.00);5F (+0.00)	3
11_67139803_67149814_DEL_1	10011	KDM2A	0.0103	0.013	0.79	1.00	1.00	1A (+0.00);3A (1 gene, +0.00);5F (+0.00)	3
11_88799086_88804149_DUP_1	5063	GRM5	0.0103	0.009	1.15	0.61	1.00	1A (+0.00);2L (GRM5, +0.00);3A (1 gene, +0.00);5F (+0.00)	3
11_88824722_88832936_DUP_1	8214	GRM5	0.0103	0.011	0.94	1.00	1.00	1A (+0.00);2L (GRM5, +0.00);3A (1 gene, +0.00);5F (+0.00)	3
11_96245972_96248381_DEL_1	2409	MAML2	0.0103	0.01	1.03	1.00	1.00	1A (+0.00);3A (1 gene, +0.00);5F (+0.00)	3
12_2418528_2424397_DUP_1	5869	CACNA1C	0.0103	0.003	3.46	0.31	1.00	1A (+0.00);2I-3 (CACNA1C, +0.00);3A (1 gene, +0.00);5F (+0.00)	3
12_108718521_108720165_DEL_1	1644	CORO1C	0.0103	0.001	10.36	0.17	1.00	1A (+0.00);3A (1 gene, +0.00);5F (+0.00)	3
14_66577693_66658869_DEL_1	81176	GPHN	0.0103	0.001	10.36	0.17	1.00	1A (+0.00);3A (1 gene, +0.00);5F (+0.00)	3
14_104783358_104786652_DEL_1	3294	AKT1	0.0103	0.002	5.18	0.24	0.98	1A (+0.00);3A (1 gene, +0.00);5F (+0.00)	3
16_6605897_6640406_DUP_1	34509	RBFOX1	0.0103	0.005	2.07	0.43	0.95	1A (+0.00);2L (RBFOX1, +0.00);3A (1 gene, +0.00);5F (+0.00)	3

16_88053092_88054415_DUP_1	1323	BANP	0.0103	0.01	1.03	1.00	0.99	1A (+0.00);2E (cf B_gain_source, +0.00);3A (1 gene, +0.00);5F (+0.00)	3
17_914183_915432_DUP_1	1249	NXN	0.0103	0.001	10.36	0.17	1.00	1A (+0.00);2I-3 (NXN, +0.00);3A (1 gene, +0.00);5F (+0.00)	3
17_79369382_79397607_DUP_1	28225	RBFOX3	0.0103	0.026	0.39	0.50	1.00	1A (+0.00);2E (cf B_gain_source, +0.00);3A (1 gene, +0.00);5F (+0.00)	3
2_50966572_50976263_DEL_1	9691	NRXN1	0.0103	0.001	10.36	0.17	1.00	1A (+0.00);3A (1 gene, +0.00);5F (+0.00)	3
2_51021094_51023511_DEL_1	2417	NRXN1	0.0103	0.003	3.46	0.31	1.00	1A (+0.00);3A (1 gene, +0.00);5F (+0.00)	3
2_74168098_74169458_DUP_1	1360	MOB1A	0.0103	0.004	2.59	0.37	0.95	1A (+0.00);2L (MOB1A, +0.00);3A (1 gene, +0.00);5F (+0.00)	3
2_128262860_128274185_DUP_1	11325	HS6ST1	0.0103	0.002	5.18	0.24	0.92	1A (+0.00);2L (HS6ST1, +0.00);3A (1 gene, +0.00);5F (+0.00)	3
2_202416475_202422056_DUP_1	5581	BMPR2	0.0103	0.004	2.59	0.37	1.00	1A (+0.00);2I-3 (BMPR2, +0.00);3A (1 gene, +0.00);5F (+0.00)	3
2_211486447_211551030_DEL_1	64583	ERBB4	0.0103	0.008	1.29	0.57	1.00	1A (+0.00);3A (1 gene, +0.00);5F (+0.00)	3
20_42228726_42230769_DUP_1	2043	PTPRT	0.0103	0.002	5.18	0.24	1.00	1A (+0.00);2L (PTPRT, +0.00);3A (1 gene, +0.00);5F (+0.00)	3
20_49195370_49202053_DUP_1	6683	STAU1	0.0103	0.013	0.79	1.00	1.00	1A (+0.00);2L (STAU1, +0.00);3A (1 gene, +0.00);5F (+0.00)	3
20_59721842_59740560_DEL_1	18718	PHACTR3	0.0103	0.006	1.72	0.48	1.00	1A (+0.00);3A (1 gene, +0.00);5F (+0.00)	3
20_62057737_62058887_DEL_1	1150	TAF4	0.0103	0.006	1.72	0.48	1.00	1A (+0.00);3A (1 gene, +0.00);5F (+0.00)	3
21_40499419_40537010_DUP_1	37591	DSCAM	0.0103	0.003	3.46	0.31	1.00	1A (+0.00);2I-3 (DSCAM, +0.00);3A (1 gene, +0.00);5F (+0.00)	3
21_40513978_40516885_DEL_1	2907	DSCAM	0.0103	0.004	2.59	0.37	1.00	1A (+0.00);3A (1 gene, +0.00);5F (+0.00)	3
22_36024517_36026041_DUP_1	1524	RBFOX2	0.0103	0.006	1.72	0.48	1.00	1A (+0.00);2L (RBFOX2, +0.00);3A (1 gene, +0.00);5F (+0.00)	3
22_41622725_41624782_DEL_1	2057	XRCC6	0.0103	0.001	10.36	0.17	1.00	1A (+0.00);3A (1 gene, +0.00);5F (+0.00)	3
3_12998990_13008224_DUP_1	9234	IQSEC1	0.0103	0.009	1.15	0.61	1.00	1A (+0.00);2I-3 (IQSEC1, +0.00);3A (1 gene, +0.00);5F (+0.00)	3
3_49990183_49995711_DEL_1	5528	RBM6	0.0103	0.001	10.36	0.17	1.00	1A (+0.00);3A (1 gene, +0.00);5F (+0.00)	3
3_77293156_77295949_DUP_1	2793	ROBO2	0.0103	0.003	3.46	0.31	1.00	1A (+0.00);2E (cf B_gain_source, +0.00);3A (1 gene, +0.00);5F (+0.00)	3
3_114569743_114572115_DEL_1	2372	ZBTB20	0.0103	0.01	1.03	1.00	0.97	1A (+0.00);2C-2 (ZBTB20,+0.00);3A (1 gene, +0.00);5F (+0.00)	3
3_114938062_114949740_DEL_1	11678	ZBTB20	0.0103	0.008	1.29	0.57	0.97	1A (+0.00);2C-2 (ZBTB20,+0.00);3A (1 gene, +0.00);5F (+0.00)	3
4_744879_747581_DEL_1	2702	PCGF3	0.0103	0.002	5.18	0.24	0.95	1A (+0.00);3A (1 gene, +0.00);5F (+0.00)	3
4_121812027_121818719_DUP_1	6692	CCNA2;EXOSC9	0.0103	0.002	5.18	0.24	1.00	1A (+0.00);2E (cf B_gain_source, +0.00);3A (2 genes, +0.00);5F (+0.00)	3
4_156914007_156915470_DEL_1	1463	PDGFC	0.0103	0.015	0.68	1.00	0.99	1A (+0.00);3A (1 gene, +0.00);5F (+0.00)	3
5_119121855_119122904_DEL_1	1049	DMXL1	0.0103	0.004	2.59	0.37	1.00	1A (+0.00);3A (1 gene, +0.00);5F (+0.00)	3
6_16390644_16400931_DUP_1	10287	ATXN1	0.0103	0.003	3.46	0.31	0.97	1A (+0.00);2I-3 (ATXN1, +0.00);3A (1 gene, +0.00);5F (+0.00)	3
6_16567648_16573310_DEL_1	5662	ATXN1	0.0103	0.002	5.18	0.24	0.97	1A (+0.00);2C-2 (ATXN1,+0.00);3A (1 gene, +0.00);5F (+0.00)	3
7_78804865_78807885_DEL_1	3020	MAGI2	0.0103	0.004	2.59	0.37	1.00	1A (+0.00);3A (1 gene, +0.00);5F (+0.00)	3
7_107083323_107089065_DEL_1	5742	PRKAR2B	0.0103	0.004	2.59	0.37	1.00	1A (+0.00);3A (1 gene, +0.00);5F (+0.00)	3
7_151808947_151813564_DEL_1	4617	LOC644090;PRKAG2	0.0103	0.004	2.59	0.37	1.00	1A (+0.00);2C-2 (PRKAG2,+0.00);3A (2 genes, +0.00);5F (+0.00)	3
7_151829313_151831180_DEL_1	1867	PRKAG2	0.0103	0.008	1.29	0.57	1.00	1A (+0.00);3A (1 gene, +0.00);5F (+0.00)	3
8_1247515_1248651_DEL_1	1136	DLGAP2	0.0103	0.066	0.15	0.02	1.00	1A (+0.00);3A (1 gene, +0.00);5F (+0.00)	3
8_1447566_1469121_DEL_1	21555	DLGAP2	0.0103	0.001	10.36	0.17	1.00	1A (+0.00);3A (1 gene, +0.00);5F (+0.00)	3
8_140841897_140843555_DEL_1	1658	PTK2	0.0103	0.005	2.07	0.43	1.00	1A (+0.00);3A (1 gene, +0.00);5F (+0.00)	3
9_10312842_10329193_DUP_1	16351	PTPRD	0.0103	0.01	1.03	1.00	1.00	1A (+0.00);2L (PTPRD, +0.00);3A (1 gene, +0.00);5F (+0.00)	3
9_14287364_14291421_DEL_1	4057	NFIB	0.0103	0.016	0.64	1.00	1.00	1A (+0.00);3A (1 gene, +0.00);5F (+0.00)	3
9_88394932_88399168_DEL_1	4236	SPIN1	0.0103	0.008	1.29	0.57	0.97	1A (+0.00);3A (1 gene, +0.00);5F (+0.00)	3
9_89429811_89432048_DUP_1	2237	SEMA4D	0.0103	0.001	10.36	0.17	0.97	1A (+0.00);2L (SEMA4D, +0.00);3A (1 gene, +0.00);5F (+0.00)	3
9_111562015_111563770_DUP_1	1755	PTGR1;ZNF483	0.0103	0.003	3.46	0.31	0.97	1A (+0.00);2L (PTGR1/ZNF483, +0.00);3A (2 genes, +0.00);5F (+0.00)	3
9_123022521_123023642_DEL_1	1121	RABGAP1	0.0103	0.003	3.46	0.31	1.00	1A (+0.00);3A (1 gene, +0.00);5F (+0.00)	3
X_44938177_44940271_DUP_1	2094	KDM6A	0.0103	0.003	3.46	0.31	1.00	1A (+0.00);2I-3 (KDM6A, +0.00);3A (1 gene, +0.00);5F (+0.00)	3
X_53387061_53392516_DEL_1	5455	SMC1A	0.0103	0.001	10.36	0.17	1.00	1A (+0.00);3A (1 gene, +0.00);5F (+0.00)	3

X_133878223_133879600_DEL_1	1377	GPC3	0.0103	0.002	5.18	0.24	1.00	1A (+0.00);3A (1 gene, +0.00);5F (+0.00)	3
X_134386897_134389034_DEL_1	2137	PHF6	0.0103	0.005	2.07	0.43	1.00	1A (+0.00);3A (1 gene, +0.00);5F (+0.00)	3

Supplementary table 9. Enrichment of long structural variants (LSV) on SEVTIN and TIGER cohorts. A significant burden of duplications and deletions was found in ultrarare variants on regions with high constraint in both cohorts. SEVTIN enrichment in ultrarare LSV is higher than the enrichment found in TIGER cohort.

Cohort	Type of SV		OR(CI)	pvalue
**TIGER**	DEL	Constraint	1.76 (1.16-2.71)	8.50E-03
		UR	1.46 (1.11-1.92)	3.11E-03
	DUP	Constraint	1.24 (1.18-1.31)	2.50E-15
		UR	1.77 (1.16-2.71)	4.27E-03
**SEVTIN**	DEL	Constraint	1.33 (1.22-1.45)	2.53E-10
		UR	5.21 (3.37-8.07)	7.03E-10
	DUP	Constraint	1.34 (1.09-1.64)	4.30E-03
		UR	5.65 (2.95-10.80)	1.45E-05

Supplementary table 10. Candidate CNV found in the TIGER cohort. CNVs found only in one sample and in SweGen callset were filtered. Pathogenicity score was calculated according to Riggs, *et al.* 2020 AMCG guidelines modifications for CNV.

VariantID	Type	TIGER (n)	TIGER (frq)	Classification	All protein coding genes
chr13_50493045_50503045_DEL	DEL	2	0.021	Uncertain significance	
chr18_20745966_20861206_DEL	DEL	2	0.021	Uncertain significance	
chr3_151795565_151830565_DEL	DEL	2	0.021	Benign	AADAC
chr7_144180809_144375811_DEL	DEL	2	0.021	Benign	CTAGE8, CTAGE4, ARHGEF35, OR2A7, OR2A42, OR2A1, ARHGEF5
chr9_39180937_39355941_DEL	DEL	6	0.062	Benign	CNTNAP3, SPATA31A1
chr9_40485968_40565970_DEL	DEL	2	0.021	Uncertain significance	
chrY_20052817_20192795_DEL	DEL	3	0.031	Uncertain significance	
chrY_20067814_20342893_DEL	DEL	5	0.052	Uncertain significance	

Supplementary figure 1. Correlation plot between microarray probes for each candidate gene for tinnitus. Rectangle clusters focus on major number of correlation pairs per probes. Non-significant correlation pairs after t-test calculation are crossed-out.

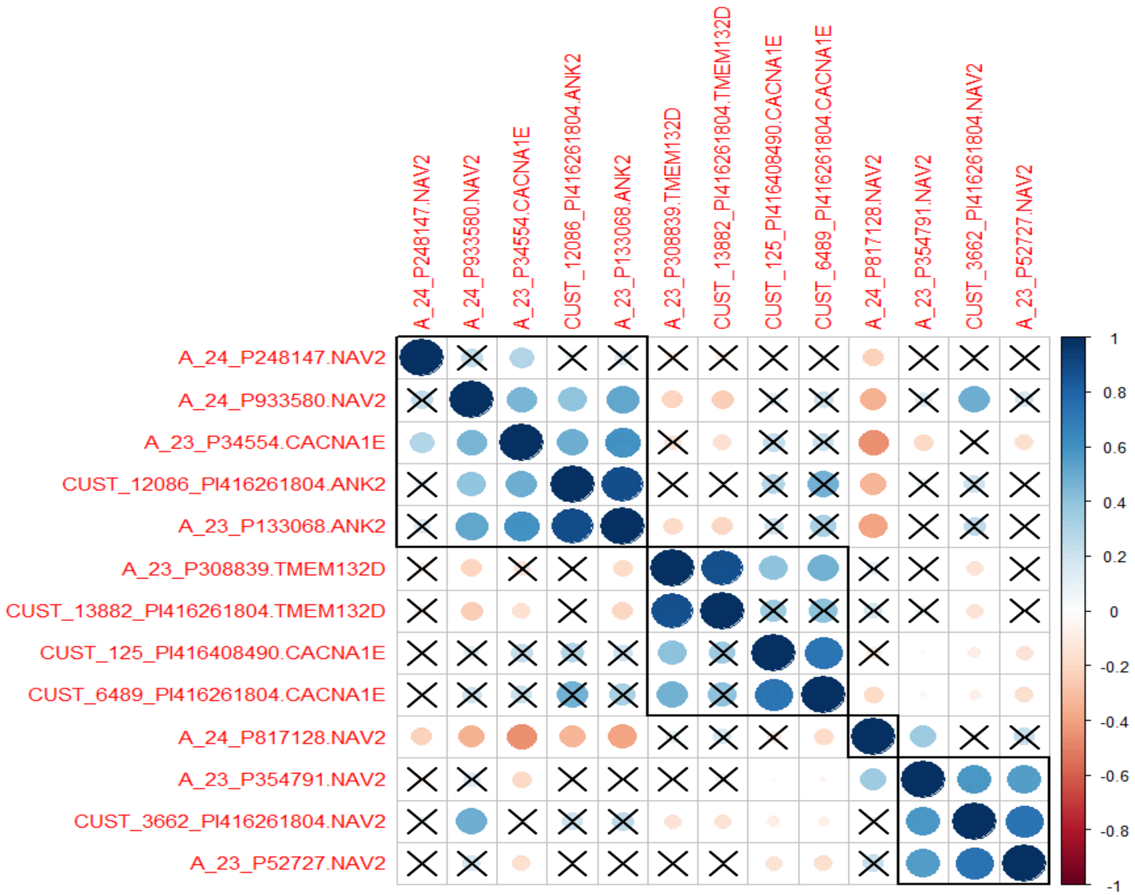

Supplementary figure 2. Scaled-log2 expression ratio for NAV2, ANK2, and CACNA1E RNAseq probes in H0351.2001 brain sample from Allen Human Brain Atlas. The human brain regions showing significant co-expression for the three genes include temporal lobe (superior temporal gyrus, middle temporal gyrus, inferior temporal gyrus, fusiform gyrus), frontal lobe (superior frontal gyrus), insula, hippocampal formation (dentate gyrus), and limbic system (cingulate gyrus). Color scale range from lower (blue) to high (red) expression measured by TPM values (scaled fragment counts derived using RSEM). Columns represents expression for each gene and brain structure: GRe-gyrus rectus; OrbGyri- orbital gyrus; orIFG-inferior frontal gyrus, orbital part; MFG-middle frontal gyrus; PCLa-s-paracentral lobule, anterior part, left, superior bank of gyrus; PCLa-i-paracentral lobule, anterior part, left, inferior bank of gyrus; PrG-precentral gyrus; SFG-m-superior frontal gyrus, left, medial bank of gyrus; SFG-l-superior frontal gyrus, left, lateral bank of gyrus; CgG-cingulate gyrus; PHG-parahippocampal gyrus; AnG-s-angular gyrus, left, superior bank of gyrus; AnG-i-angular gyrus, left, inferior bank of gyrus; SMG-i-supramarginal gyrus, left, inferior bank of gyrus; PoG-cs-postcentral gyrus, left, bank of the central sulcus; PCu-precuneus; SPL-superior parietal lobule; FuG-its-fusiform gyrus, left, bank of the its; ITG-inferior temporal gyrus; MTG-middle temporal gyrus; STG-superior temporal gyrus; GP-globus pallidus; CbCx-cerebellar cortex; Insula - Insula; Putamen - Putamen; str_V1 - Striatum; pest_V2 - pracentral lobule, olfactory area.

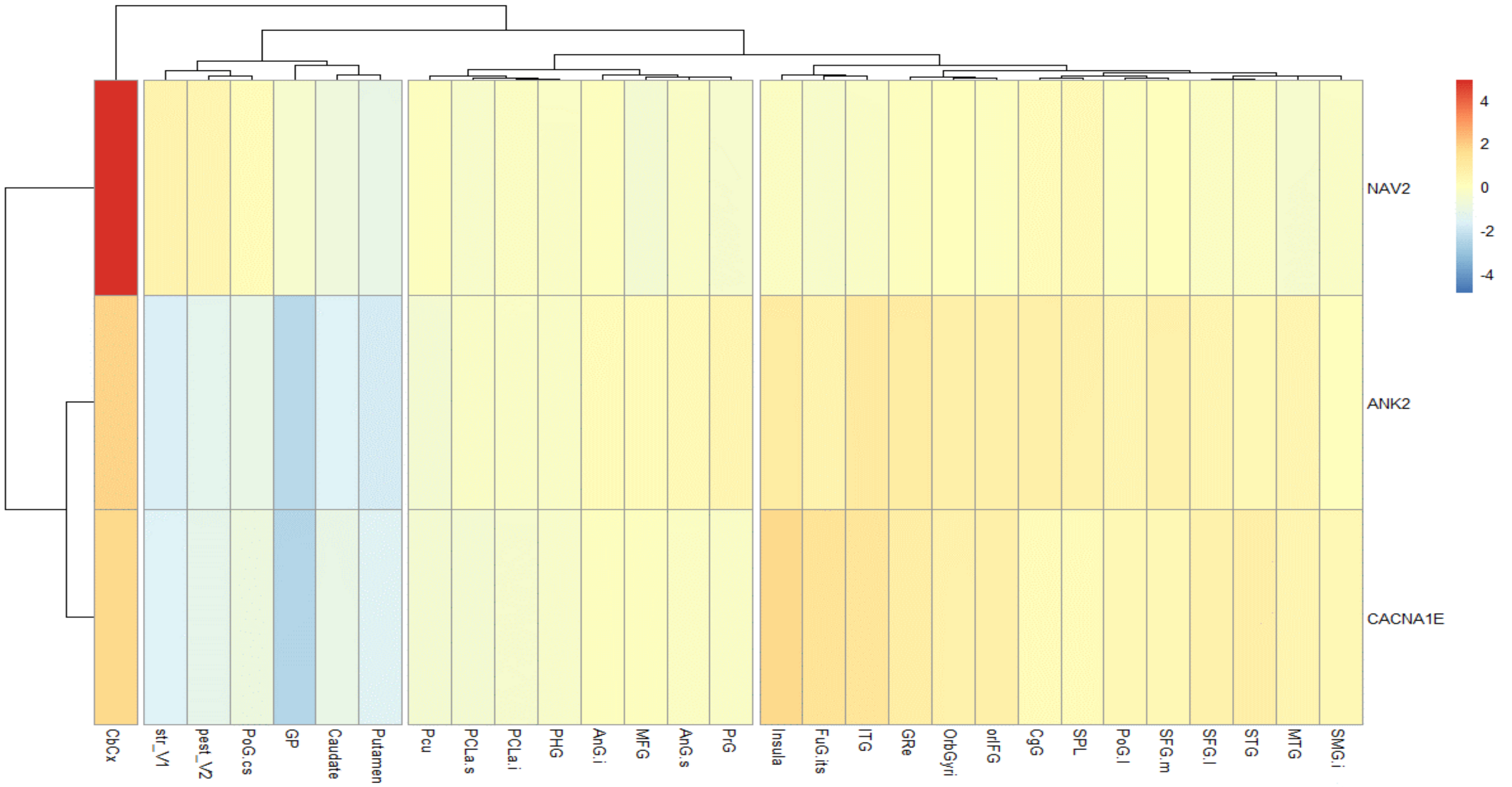

Supplement: Supplementary file 1 — Supplemental material [file 41525_2022_341_MOESM1_ESM.pdf]
