# Supplementary material for: Using coding and non-coding rare variants to target candidate genes in patients with severe tinnitus
Source: NPJ Genom Med. 2022 Nov 30;7:70. doi: 10.1038/s41525-022-00341-w (PMC9712652; doi:10.1038/s41525-022-00341-w)
Supplement: Supplementary file 2 — Nature Reporting Summary [file 41525_2022_341_MOESM2_ESM.pdf]

nature portfolio

Corresponding author(s): Alvaro Gallego-Martinez
Last updated by author(s): Sep 6, 2022

Reporting Summary

Nature Portfolio wishes to improve the reproducibility of the work that we publish. This form provides structure for consistency and transparency in reporting. For further information on Nature Portfolio policies, see our Editorial Policies and the Editorial Policy Checklist.

Statistics

For all statistical analyses, confirm that the following items are present in the figure legend, table legend, main text, or Methods section.

n/a	Confirmed	
☐	☒	The exact sample size (*n*) for each experimental group/condition, given as a discrete number and unit of measurement
☐	☒	A statement on whether measurements were taken from distinct samples or whether the same sample was measured repeatedly
☐	☒	The statistical test(s) used AND whether they are one- or two-sided<br>*Only common tests should be described solely by name; describe more complex techniques in the Methods section.*
☐	☒	A description of all covariates tested
☐	☒	A description of any assumptions or corrections, such as tests of normality and adjustment for multiple comparisons
☐	☒	A full description of the statistical parameters including central tendency (e.g. means) or other basic estimates (e.g. regression coefficient) AND variation (e.g. standard deviation) or associated estimates of uncertainty (e.g. confidence intervals)
☐	☒	For null hypothesis testing, the test statistic (e.g. *F*, *t*, *r*) with confidence intervals, effect sizes, degrees of freedom and *P* value noted<br>*Give P values as exact values whenever suitable.*
☐	☒	For Bayesian analysis, information on the choice of priors and Markov chain Monte Carlo settings
☐	☒	For hierarchical and complex designs, identification of the appropriate level for tests and full reporting of outcomes
☐	☒	Estimates of effect sizes (e.g. Cohen's *d*, Pearson's *r*), indicating how they were calculated

Our web collection on statistics for biologists contains articles on many of the points above.

Software and code

Policy information about availability of computer code

Data collection	A list of databases and tools used is provided:<br>Sarek Nextflow pipeline v. 2.6.1<br>Variant Effect Predictor v106<br>vcfanno v0.2.9<br>CADD v1.6<br>dbNSFP v. 4.1_a.<br>AnnotSV v3.0.6<br>gnomAD v2 SV GRCh38 liftover<br>gnomAD v3 GRCh38<br>CNVKit v.0.9.8
Data analysis	Main data analysis pipeline used is Sarek Nextflow pipeline, currently open published under Garcia et al, 2020 (doi: 10.12688/f1000research.16665.2). GitHub repository: https://github.com/nf-core/sarek . Tools used in this pipeline version and their respective versions are detailed in https://nf-co.re/sarek/2.6.1

For manuscripts utilizing custom algorithms or software that are central to the research but not yet described in published literature, software must be made available to editors and reviewers. We strongly encourage code deposition in a community repository (e.g. GitHub). See the Nature Portfolio guidelines for submitting code & software for further information.

Data

Policy information about availability of data

All manuscripts must include a data availability statement. This statement should provide the following information, where applicable:

- Accession codes, unique identifiers, or web links for publicly available datasets
- A description of any restrictions on data availability
- For clinical datasets or third party data, please ensure that the statement adheres to our policy

The datasets used and/or analyzed during the current study are available from the corresponding author on reasonable request.

Human research participants

Policy information about studies involving human research participants and Sex and Gender in Research.

Reporting on sex and gender	We followed 'Sex and Gender Equity in Research – SAGER – guidelines' to represent biological sex when collected. This data is detailed disaggregated in the manuscript. Sex-based analysis is performed in the manuscript. No gender-based analysis is performed.
Population characteristics	Study cohort is form swedish population with a different range of age, following tinnitus diagnosis according to tinnitus questionnaries.
Recruitment	Adult participants (> 18 years old) from LifeGene were recruited to the Swedish Tinnitus Outreach Project (STOP) and registered their interest on the STOP website (https://stop.ki.se), after which they received additional information and a consent form by mail. Involved persons answers a series of questionnaries detailed in the manuscript, selecting a subset of tinnitus cases with Tinnitus Functional Index > 48, chronic and constant tinnitus, for a total of 97 samples conforming TIGER cohort.
Ethics oversight	The project was approved by the local ethics committee "Regionala etikprövningsnämnden" in Stockholm (2015/2129-31/1). Written informed consent was obtained from all subjects.

Note that full information on the approval of the study protocol must also be provided in the manuscript.

Field-specific reporting

Please select the one below that is the best fit for your research. If you are not sure, read the appropriate sections before making your selection.

☒ Life sciences ☐ Behavioural & social sciences ☐ Ecological, evolutionary & environmental sciences

For a reference copy of the document with all sections, see nature.com/documents/nr-reporting-summary-flat.pdf

Life sciences study design

All studies must disclose on these points even when the disclosure is negative.

Sample size	Samples sizes were chosen according to clinical and psychometric criteria from a known registered cohort by LifeGene. We have selected a subgroup patients from TIGER according to clinical and psychometric criteria (THI score > 56 was considered a severe phenotype, SEVTIN cohort).
Data exclusions	We focus on likely-pathogenic to pathogenic variants, excluding non-pathogenic variants using Variant Effect Prediction (VEP) tool. Filtering of variants followed GATK recommended filtering and quality steps.
Replication	Replication was performed in a cohort of tinnitus cases exomes of the same swedish population (JAGUAR, n=148).
Randomization	No randomization was performed.
Blinding	Blinding was not relevant to this study as cases were totally anonymized and data was disaggregated.

Reporting for specific materials, systems and methods

We require information from authors about some types of materials, experimental systems and methods used in many studies. Here, indicate whether each material, system or method listed is relevant to your study. If you are not sure if a list item applies to your research, read the appropriate section before selecting a response.

Materials & experimental systems

n/a	Involved in the study
☒	☐ Antibodies
☒	☐ Eukaryotic cell lines
☒	☐ Palaeontology and archaeology
☒	☐ Animals and other organisms
☒	☐ Clinical data
☒	☐ Dual use research of concern

Methods

n/a	Involved in the study
☒	☐ ChIP-seq
☒	☐ Flow cytometry
☒	☐ MRI-based neuroimaging
